# Supplementary material for: Post-replicative initial expression of PAX6 during neuroectoderm differentiation
Source: EMBO J. 2025 Oct 21;44(23):7090–118. doi: 10.1038/s44318-025-00605-y (PMC12669799; doi:10.1038/s44318-025-00605-y)
Supplement: Supplementary file 2 — Appendix [file 44318_2025_605_MOESM2_ESM.pdf]

Appendix

Post-replicative initial expression of the cell fate regulator *PAX6* during neuroectoderm differentiation

Appendix Tables

Appendix Table S1. pp. 2-3

Appendix Figures

Appendix Figure S1. p. 4
Appendix Figure S2. p. 5
Appendix Figure S3. p. 6
Appendix Figure S4. pp. 7-8
Appendix Figure S5. p. 9

Appendix Table S1. Top 50 transcription factor binding sites of the *PAX6* 500 bp promoter analyzed by the JASPAR database (2022)

Chromosome	Start	End	Score	Orientation	Transcription factor
chr11	31811726	31811737	662	+	KLF15
chr11	31811493	31811511	659	–	ZNF454
chr11	31811726	31811738	652	–	PATZ1
chr11	31811727	31811741	639	+	EGR1
chr11	31811732	31811744	611	–	PATZ1
chr11	31811726	31811736	602	+	KLF5
chr11	31811726	31811737	602	+	KLF16
chr11	31811725	31811737	597	–	ZNF281
chr11	31811540	31811552	562	+	ZBTB14
chr11	31811732	31811752	557	+	RREB1
chr11	31811725	31811737	546	+	ZNF148
chr11	31811727	31811736	541	–	SP1
chr11	31811727	31811736	541	–	KLF1
chr11	31811727	31811736	541	–	SP2
chr11	31811727	31811736	541	–	SP4
chr11	31811727	31811736	541	–	KLF14
chr11	31811727	31811736	541	–	KLF12
chr11	31811727	31811736	541	–	KLF10
chr11	31811727	31811736	541	–	KLF7
chr11	31811316	31811327	539	+	POU5F1
chr11	31811736	31811747	534	+	KLF15
chr11	31811315	31811328	533	+	POU2F3
chr11	31811732	31811743	532	+	KLF15
chr11	31811737	31811751	530	+	WT1
chr11	31811613	31811625	529	–	ZBTB7B
chr11	31811725	31811738	528	+	SP3
chr11	31811738	31811758	528	+	RREB1
chr11	31811727	31811742	526	+	EGR3
chr11	31811613	31811625	520	–	ZBTB7C
chr11	31811726	31811737	520	+	KLF11
chr11	31811727	31811741	514	+	WT1
chr11	31811315	31811327	513	+	POU2F1
chr11	31811369	31811387	513	–	ZNF454
chr11	31811628	31811640	513	+	SP8
chr11	31811737	31811751	513	+	EGR1
chr11	31811367	31811381	512	+	ZFX

chr11	31811741	31811753	512	+	KLF4
chr11	31811726	31811738	509	+	SP9
chr11	31811628	31811639	504	+	KLF16
chr11	31811802	31811813	502	+	ZEB1
chr11	31811819	31811836	499	+	SPI1
chr11	31811525	31811543	498	–	ZNF816
chr11	31811737	31811746	494	–	SP2
chr11	31811743	31811752	494	–	KLF10
chr11	31811725	31811737	487	+	KLF4
chr11	31811533	31811547	486	+	ZNF610
chr11	31811355	31811368	482	+	POU2F3
chr11	31811735	31811747	482	–	ZNF281
chr11	31811496	31811506	481	+	PLAGL2
chr11	31811743	31811752	481	–	KLF1

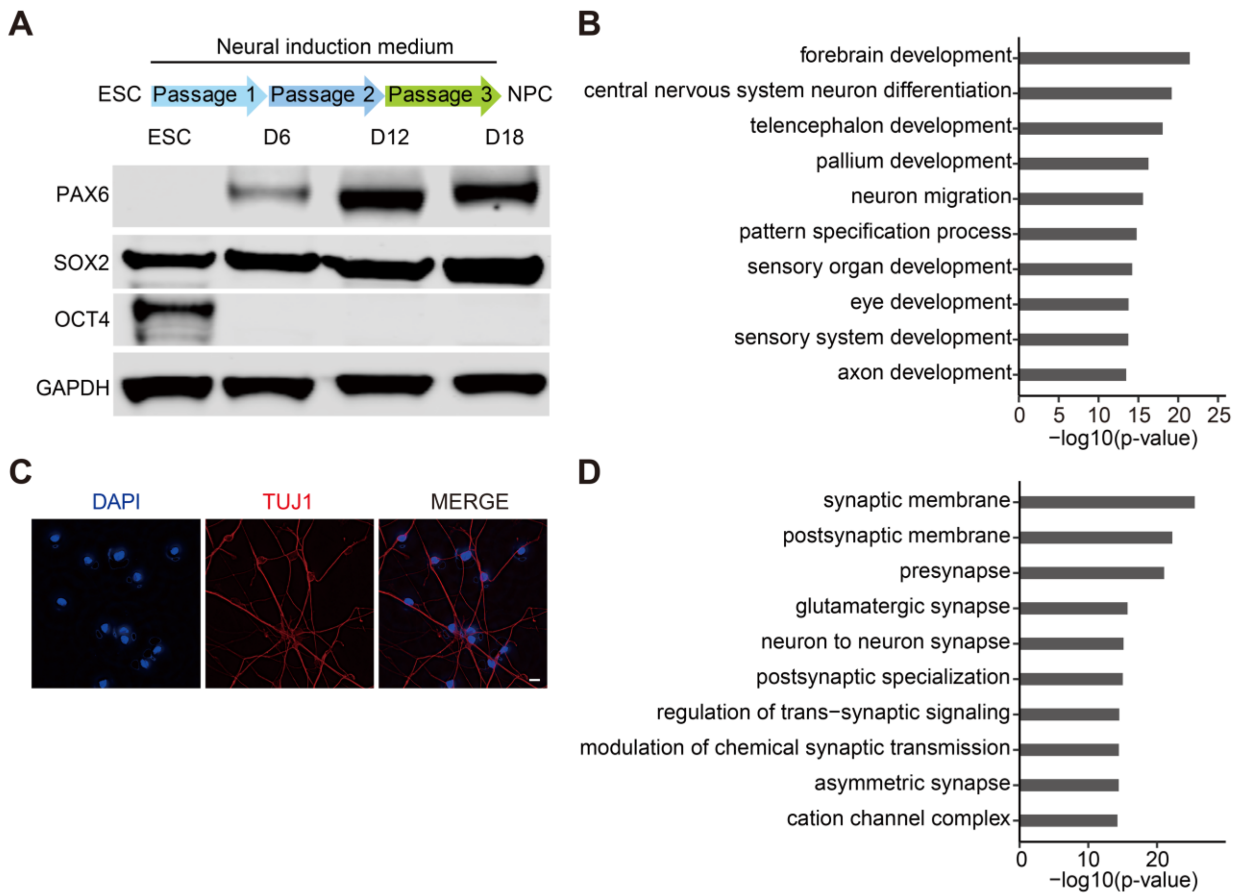

Appendix Figure S1. Establishment and characterization of the in vitro neural induction system from ESCs, related to Fig. 1.

(**A**) Western blot analysis of the protein levels of PAX6, SOX2, and OCT4 in ESCs and NPCs at day 6 (D6), day 12 (D12), and day 18 (D18) of neural induction. GAPDH was used as the loading control. (**B**) Gene Ontology (GO) enrichment analysis of upregulated genes in NPC_D18 compared to ESCs. Top 10 pathways are shown. (**C**) Image of forebrain neurons (differentiated from ESC-derived NPCs) stained with DAPI and antibody against the neuron marker TUJ1 (β-III Tubulin). Scale bar, 10 μm. (**D**) GO enrichment analysis of upregulated genes of NPC-derived neurons compared to NPC_D18. Top 10 pathways are shown. Statistical significance of GO enrichment analysis in (**B**) and (**D**) is determined using the hypergeometric test and Benjamini-Hochberg false discovery rate (FDR) correction, with a significance threshold of $p < 0.05$.

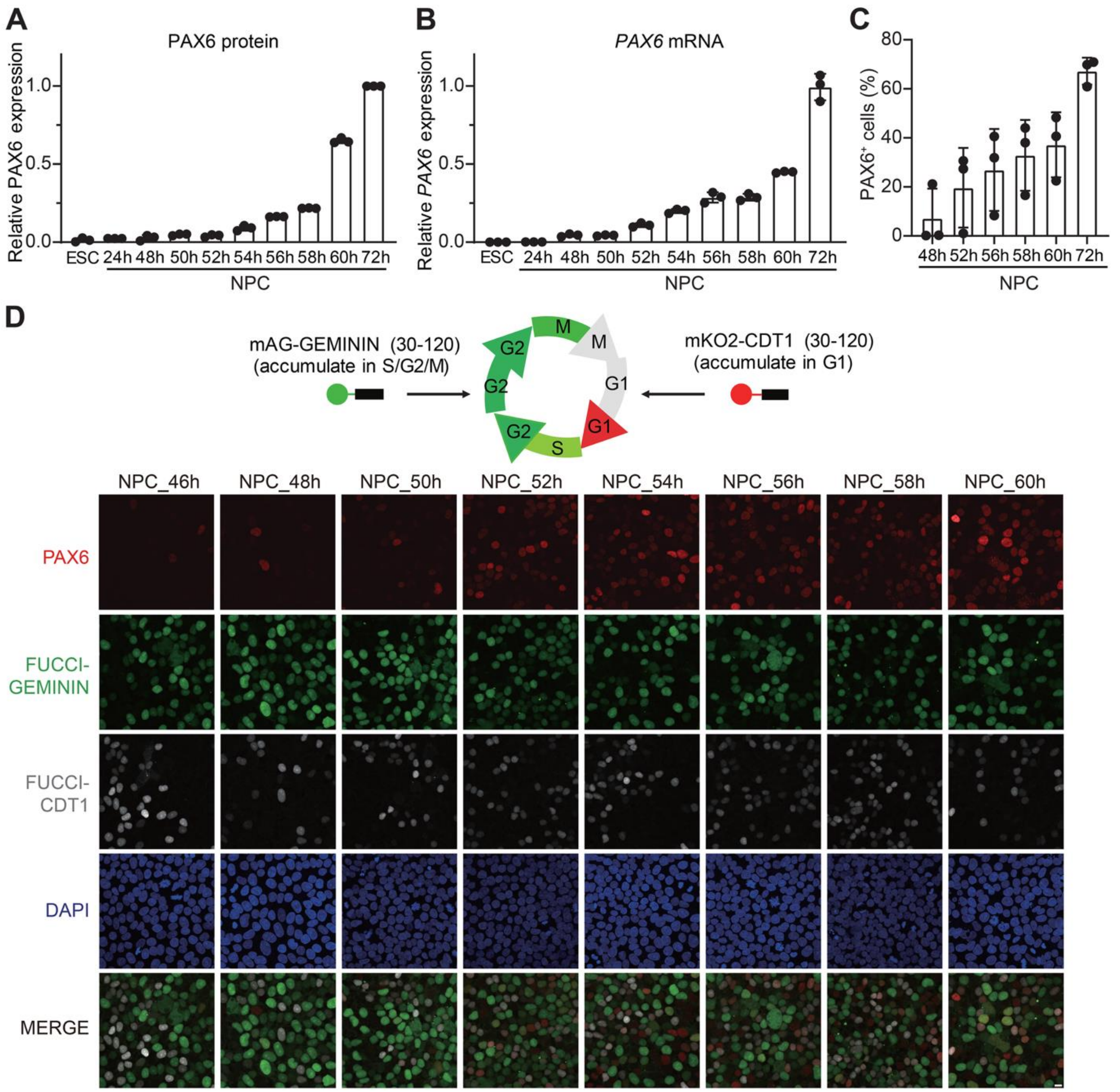

Appendix Figure S2. *PAX6* expression is activated between days 2 and 3 of neural induction, related to Fig. 2.

(**A**) Quantification of PAX6 protein levels (detected by Western blot) at different time points during ESC–NPC differentiation. Mean ± SD; n=3 independent experiments. (**B**) Quantification of *PAX6* mRNA levels (detected by qRT-PCR) at different time points during ESC–NPC differentiation. Mean ± SD; n=3 independent experiments. (**C**) Quantification of the percentage of $PAX6^{+}$ cells at different time points during ESC–NPC differentiation. Mean ± SD; n=3 independent experiments. (**D**) Schematic illustration of the FUCCI system (upper panel) and images of FUCCI ESCs at indicated times of neural induction stained with DAPI and the PAX6 antibody (lower panels). FUCCI signals (GEMININ, green; CDT1, white) indicate cell cycle status. Scale bar, 10 μm.

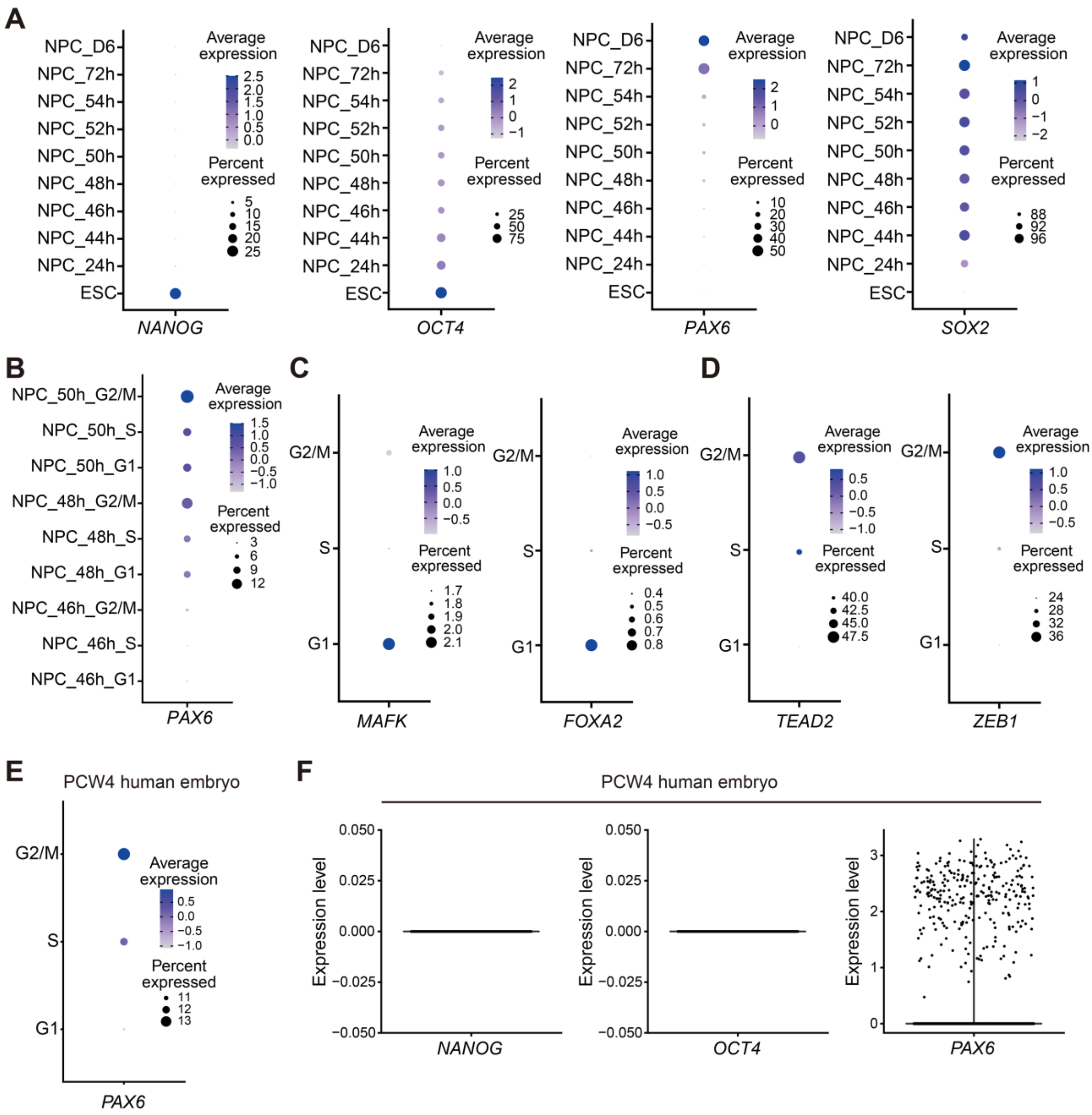

Appendix Figure S3. Single-cell analysis of gene expression from NPC_D2 to NPC_D3, related to Fig. 2.

(**A**) Dot blot showing the expression of *NANOG*, *OCT4*, *PAX6*, and *SOX2* from ESCs to NPC_D6 based on the single-cell RNA sequencing (scRNA-seq) data. (**B**) Dot blot showing the cell cycle distribution of *PAX6*-positive cells from NPC_46h to NPC_50h. (**C**, **D**) Dot blots showing two representative genes that are highly expressed in the G1 phase (**C**) or the G2 phase (**D**) based on the integrated scRNA-seq data. (**E**) Dot plot showing the cell cycle distribution of all *PAX6*$^{+}$ cells in publicly available scRNA-seq datasets of Carnegie stage 12 (CS12; around embryonic day 26–27 or PCW4) human embryonic tissues; n=1 embryo. (**F**) Violin plot showing the expression of *NANOG*, *OCT4*, and *PAX6* in scRNA-seq datasets in (**E**).

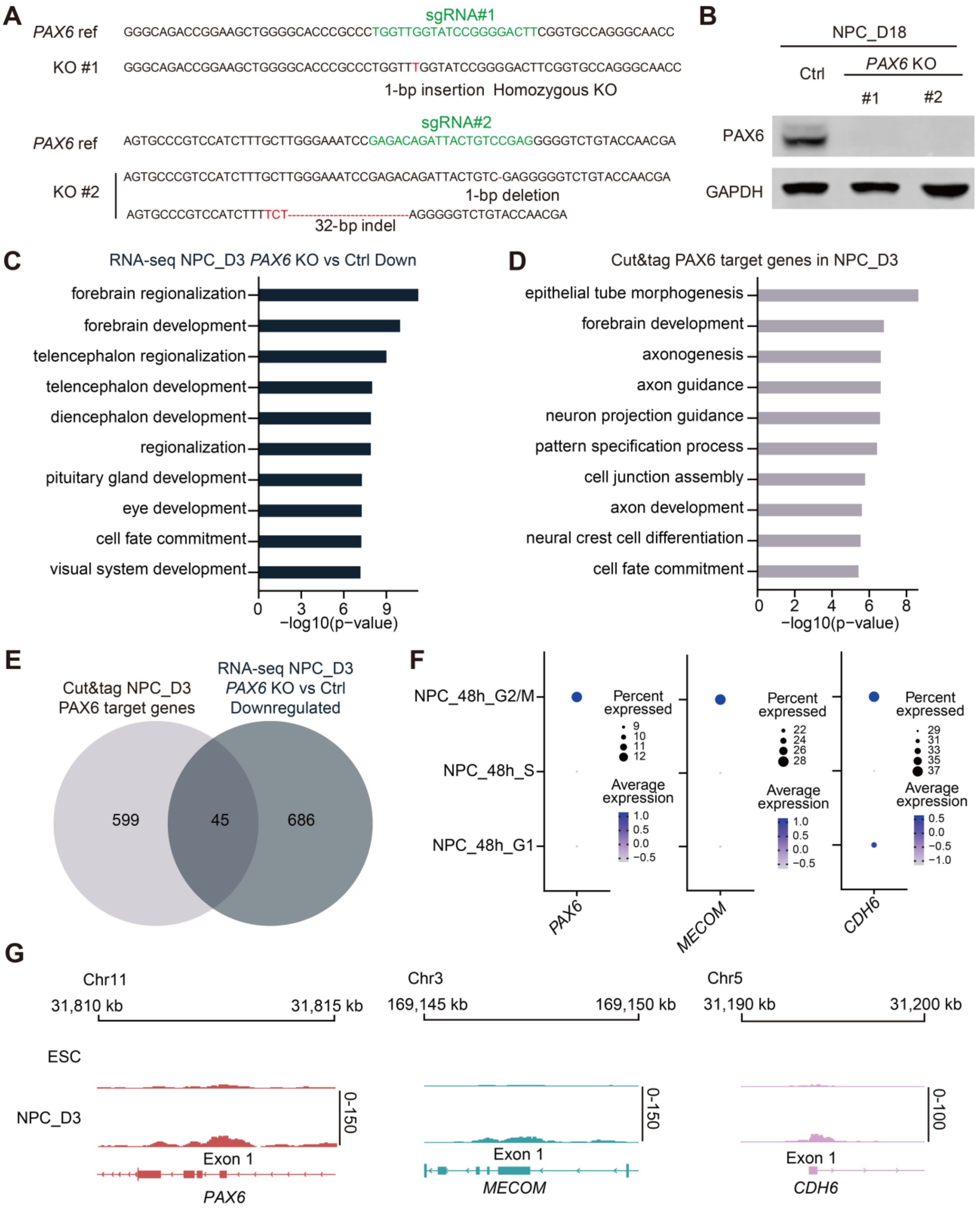

Appendix Figure S4. ***PAX6*** **promotes the expression of downstream genes during early neural induction, related to Fig. 2.**

(**A**) Sanger sequencing of the *PAX6* gene in two ESC knock out (KO) clones. The sgRNA sequence was highlighted in green, and indels in the genome were indicated in red. (**B**) Western blots showing the protein levels of PAX6 in WT and *PAX6* KO NPCs at day 18 of neural induction. (**C**) Top 10 enriched Gene Ontology (GO) pathways of downregulated genes in *PAX6* KO NPC_D3 compared to WT NPC_D3. (**D**) Top 10 enriched GO pathways of genes with PAX6-targeted peaks in NPC_D3 based on PAX6 CUT&Tag data analysis. (**E**) Venn diagram illustrating the overlap between genes downregulated in *PAX6* KO NPC_D3 in (**C**) and genes with PAX6 occupancy in (**D**). (**F**) Dot plot illustrating the expression of *PAX6*, *MECOM*, and *CDH6* across cell cycle phases at NPC_48h, based on scRNA-seq data. (**G**) CUT&Tag signal tracks of representative PAX6-bound genes in NPC_D3 samples. PAX6 CUT&Tag data from ESCs were included as a negative control. Statistical significance of GO enrichment analysis in (**C**) and (**D**) is determined using the hypergeometric test and Benjamini-Hochberg false discovery rate (FDR) correction, with a threshold of $p < 0.05$.

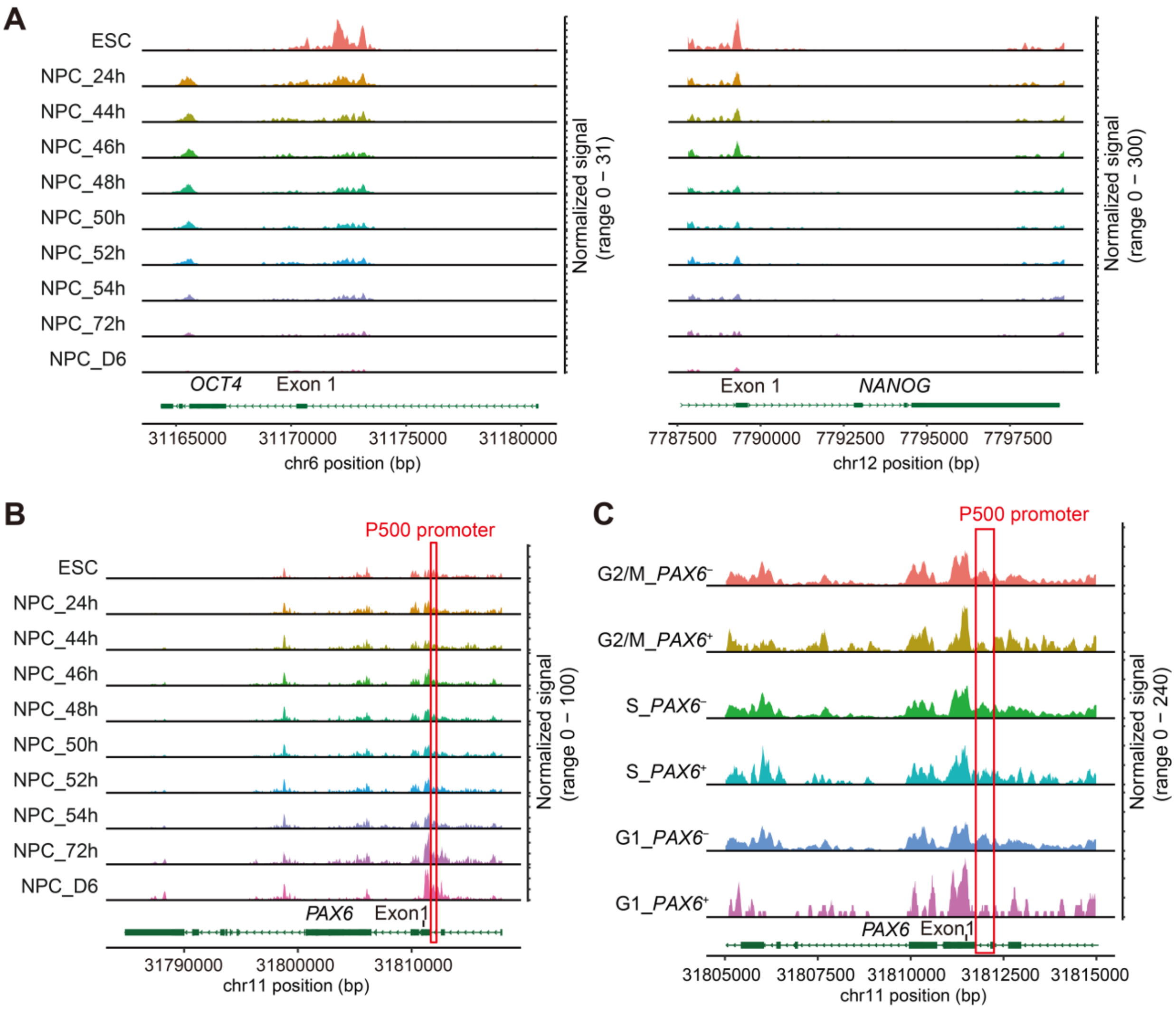

Appendix Figure S5. Chromatin accessibility of *PAX6* during early neural induction and across different cell cycle phases, related to Fig. 3.

(**A**, **B**) The chromatin accessibility of *OCT4* (**A**, left panel), *NANOG* (**A**, right panel), and *PAX6* (**B**) from ESCs to NPC_D6. Exon 1 of the three genes were labeled. (**C**) *PAX6* chromatin accessibility across G1, S, and G2/M phases in *PAX6*-positive and *PAX6*-negative cells. Peaks shown in each panel represented merged data from samples NPC_24 through NPC_52. The P500 *PAX6* promoter was highlighted.
