# Supplementary figures and images for: Post-replicative initial expression of PAX6 during neuroectoderm differentiation (part 1 of 2)
Source: EMBO J. 2025 Oct 21;44(23):7090–118. doi: 10.1038/s44318-025-00605-y (PMC12669799; doi:10.1038/s44318-025-00605-y)

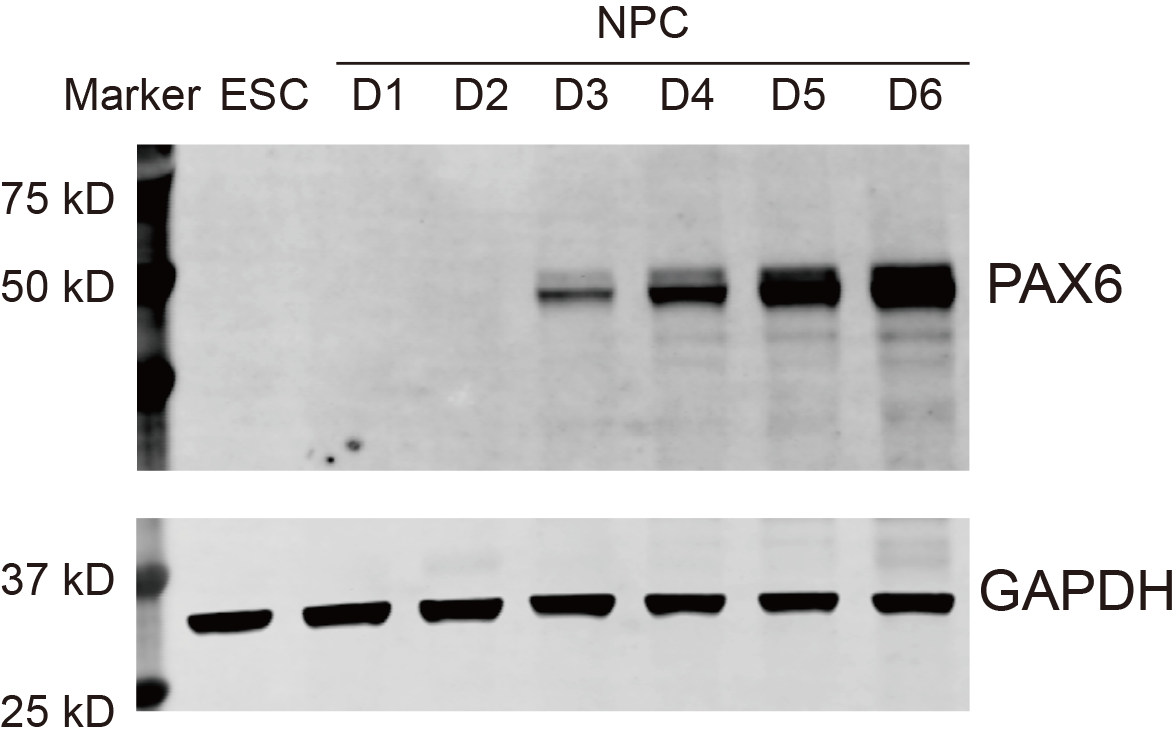

Supplement: Supplementary file 3 — Source data Fig. 1 [file 44318_2025_605_MOESM3_ESM.zip › Fig. 1/1A/Neural induction_ESC to D6.tif]

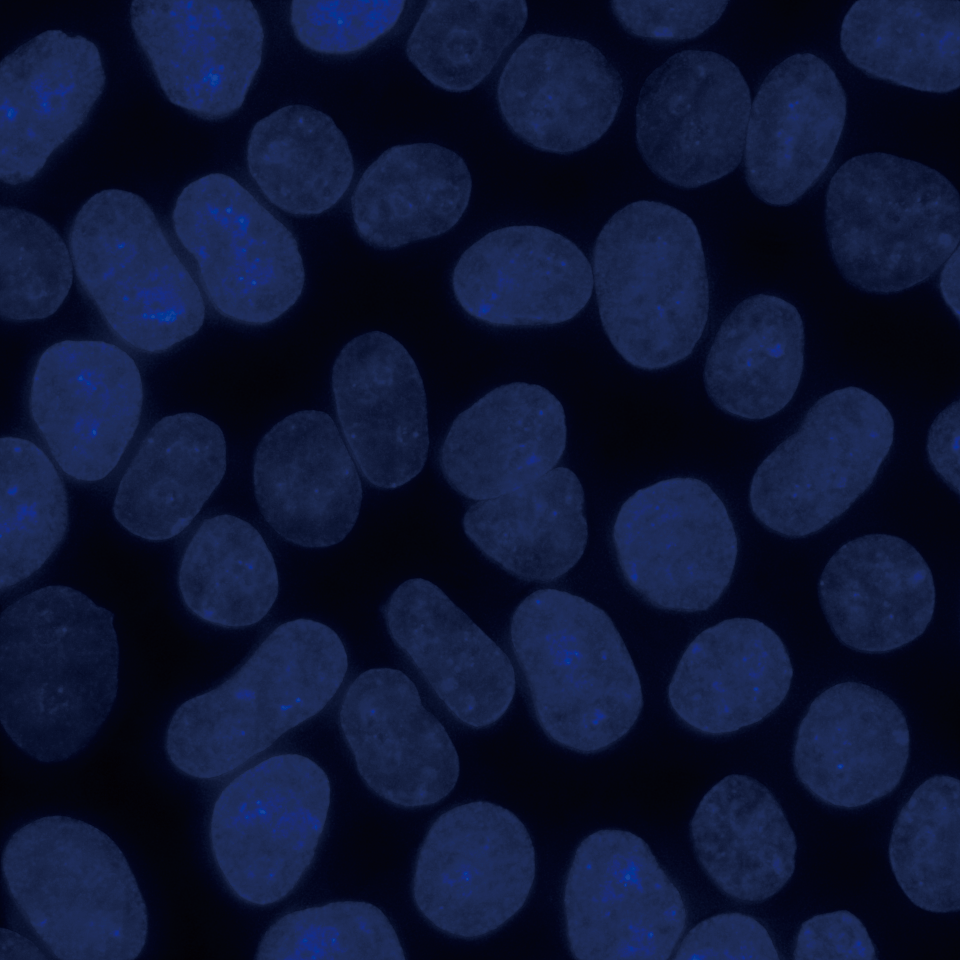

Supplement: Supplementary file 3 — Source data Fig. 1 [file 44318_2025_605_MOESM3_ESM.zip › Fig. 1/1B/DAPI/D2.tif]

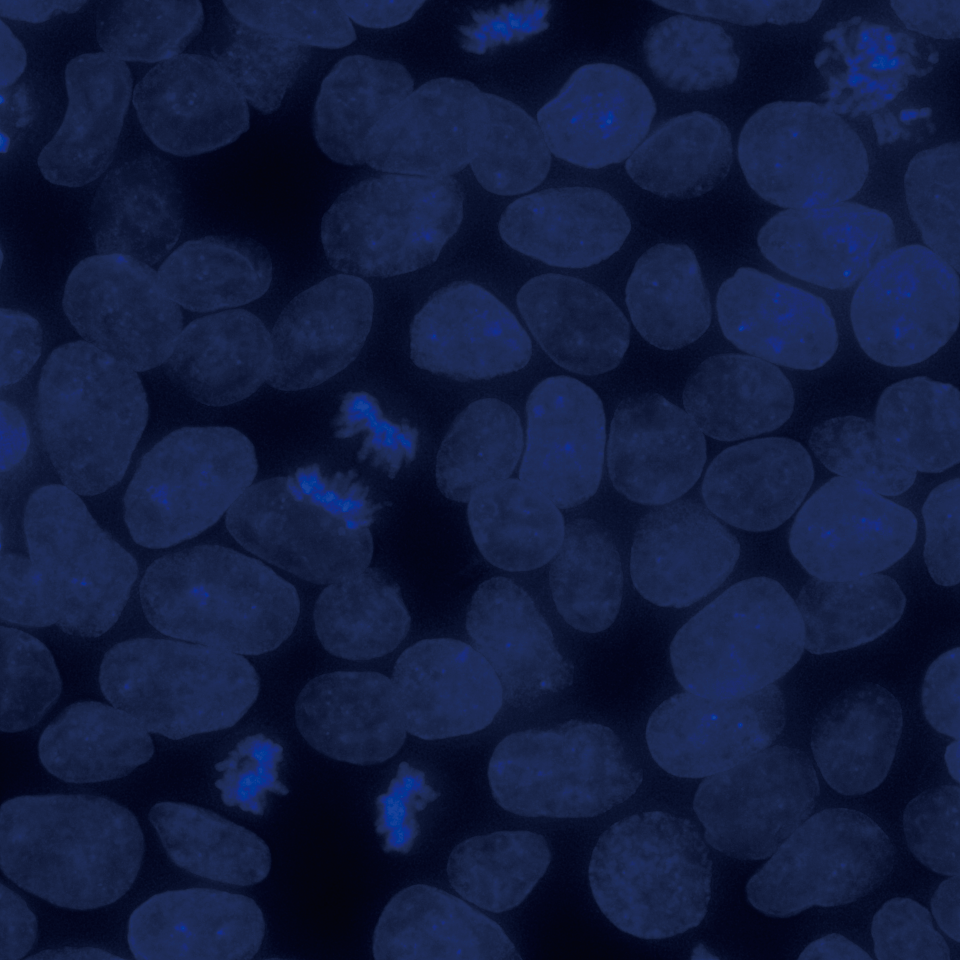

Supplement: Supplementary file 3 — Source data Fig. 1 [file 44318_2025_605_MOESM3_ESM.zip › Fig. 1/1B/DAPI/D3.tif]

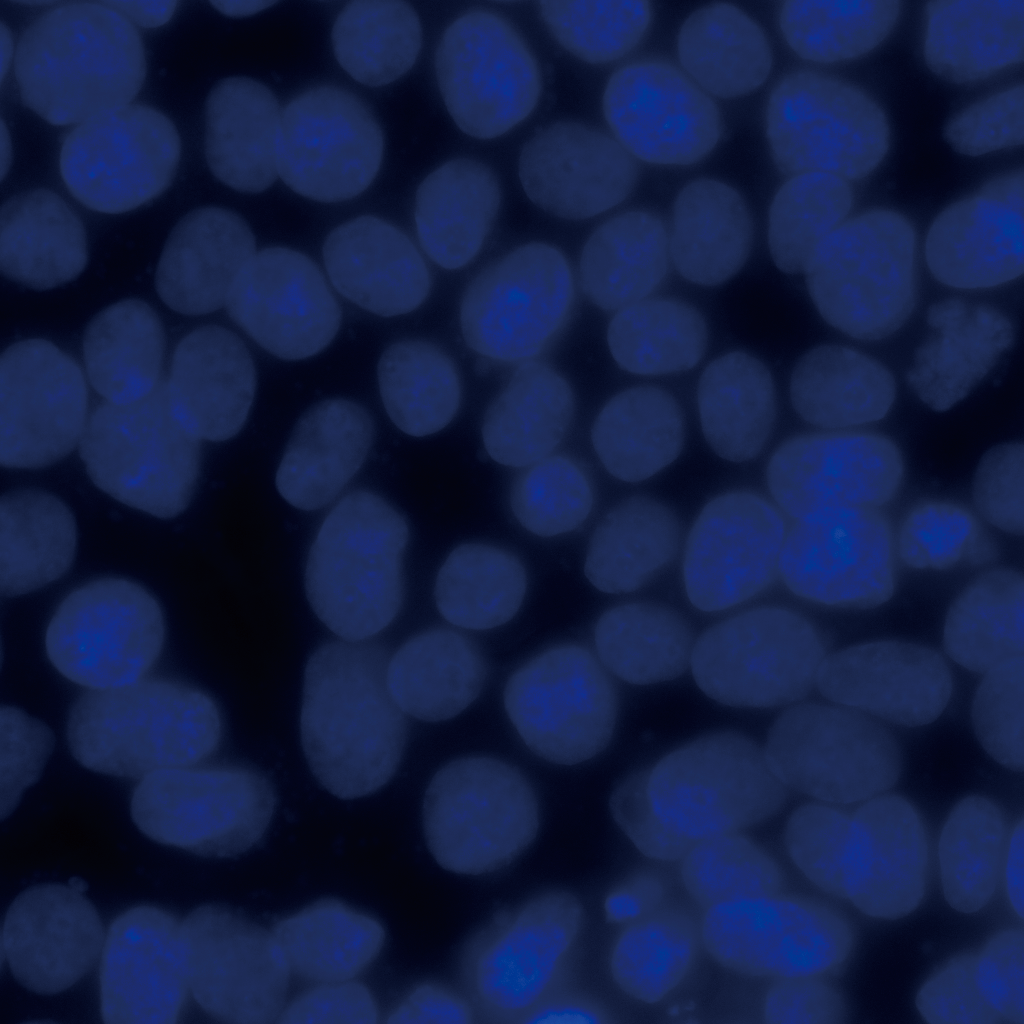

Supplement: Supplementary file 3 — Source data Fig. 1 [file 44318_2025_605_MOESM3_ESM.zip › Fig. 1/1B/DAPI/D4.tif]

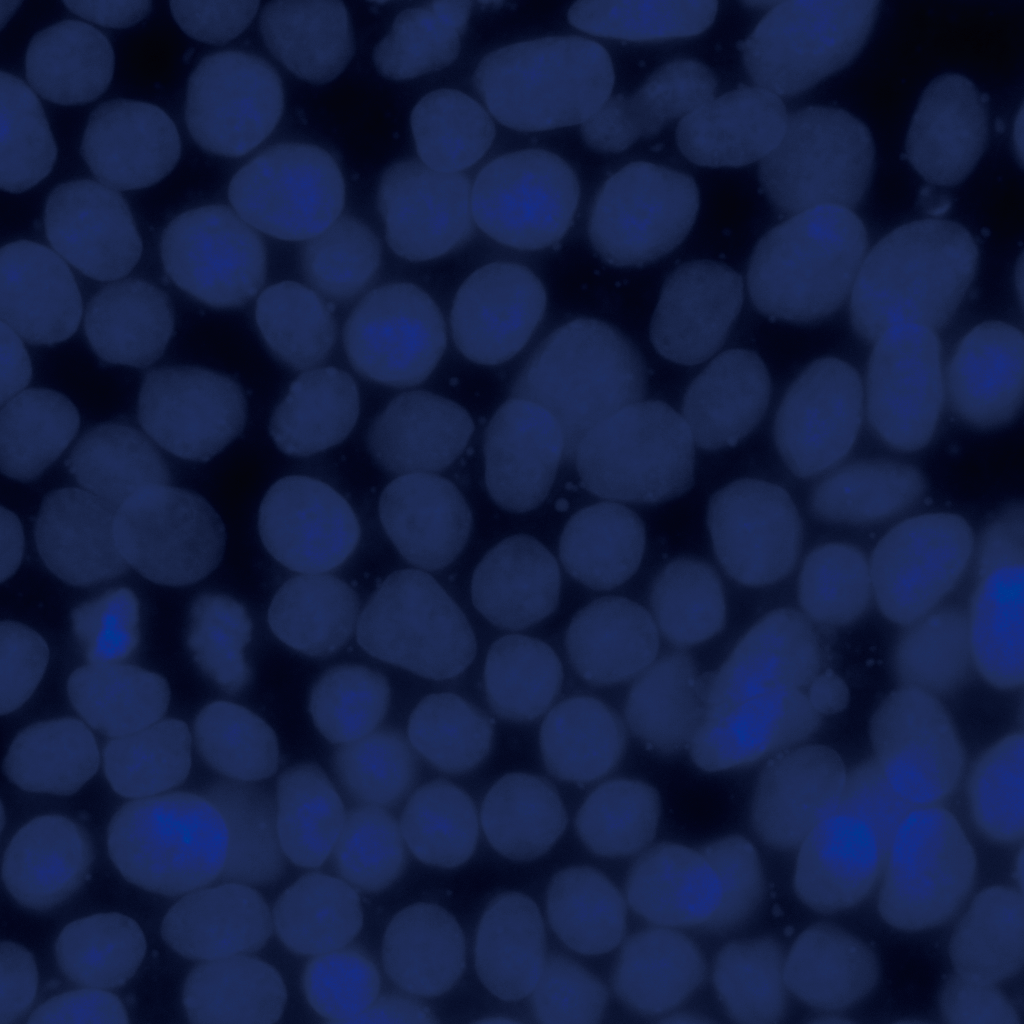

Supplement: Supplementary file 3 — Source data Fig. 1 [file 44318_2025_605_MOESM3_ESM.zip › Fig. 1/1B/DAPI/D5.tif]

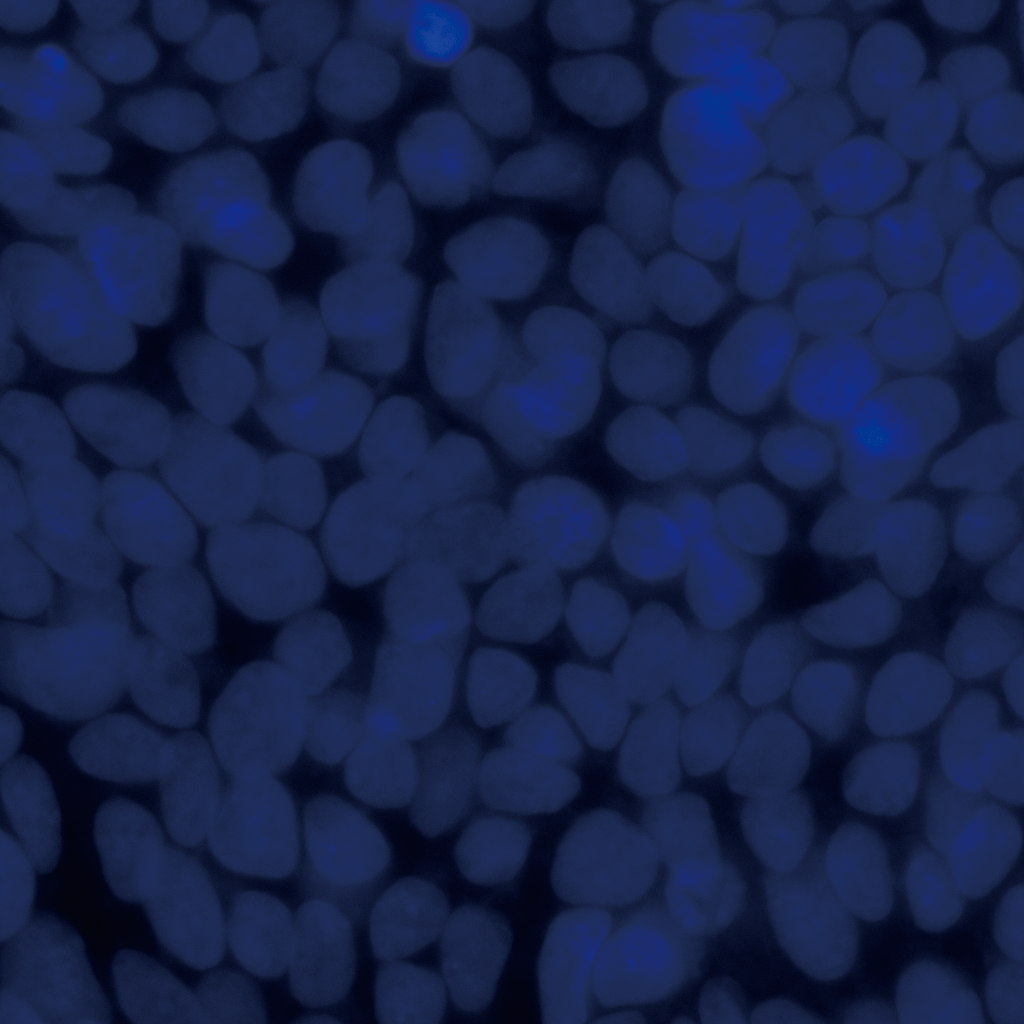

Supplement: Supplementary file 3 — Source data Fig. 1 [file 44318_2025_605_MOESM3_ESM.zip › Fig. 1/1B/DAPI/D6.tif]

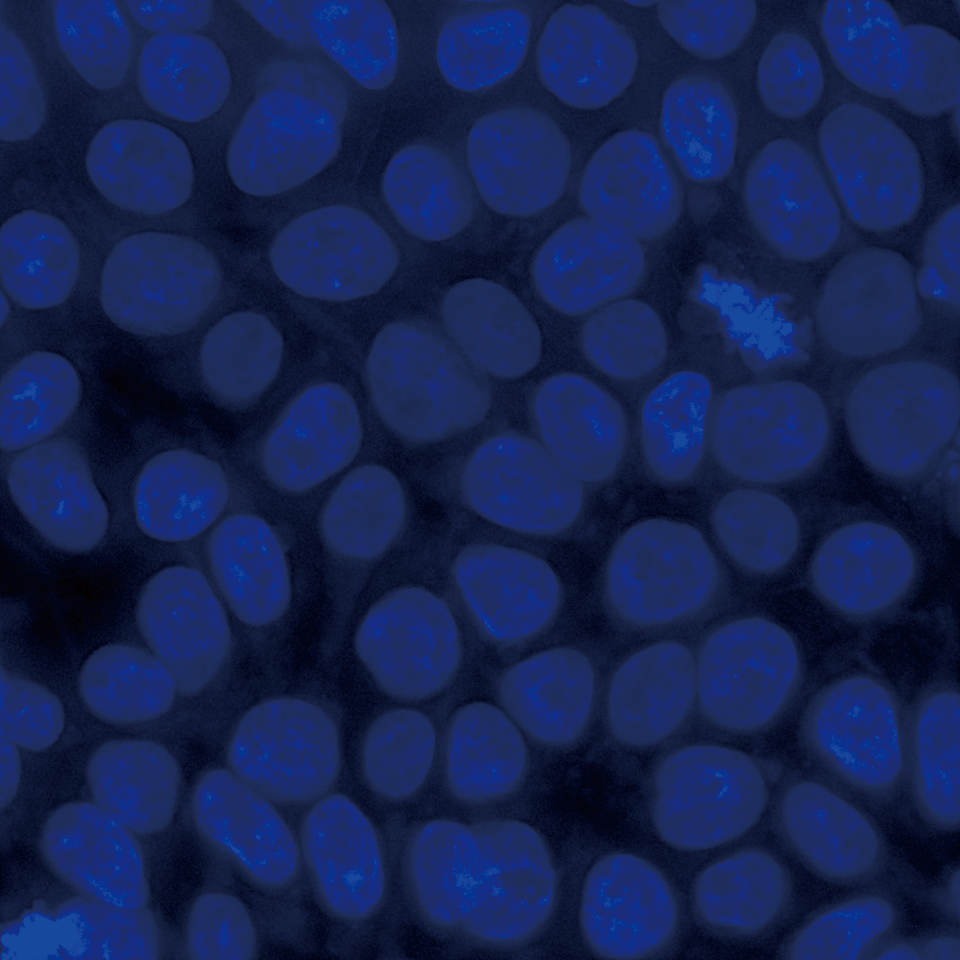

Supplement: Supplementary file 3 — Source data Fig. 1 [file 44318_2025_605_MOESM3_ESM.zip › Fig. 1/1B/DAPI/ESC.tif]

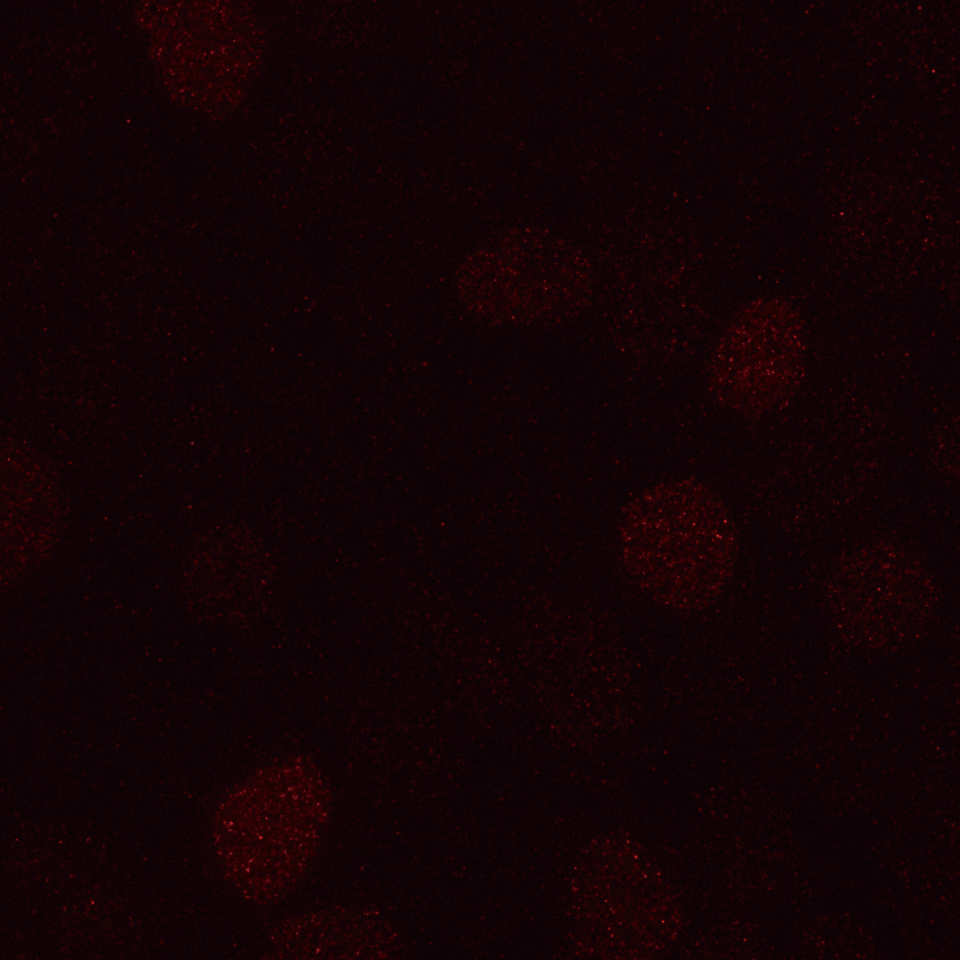

Supplement: Supplementary file 3 — Source data Fig. 1 [file 44318_2025_605_MOESM3_ESM.zip › Fig. 1/1B/PAX6/D2.tif]

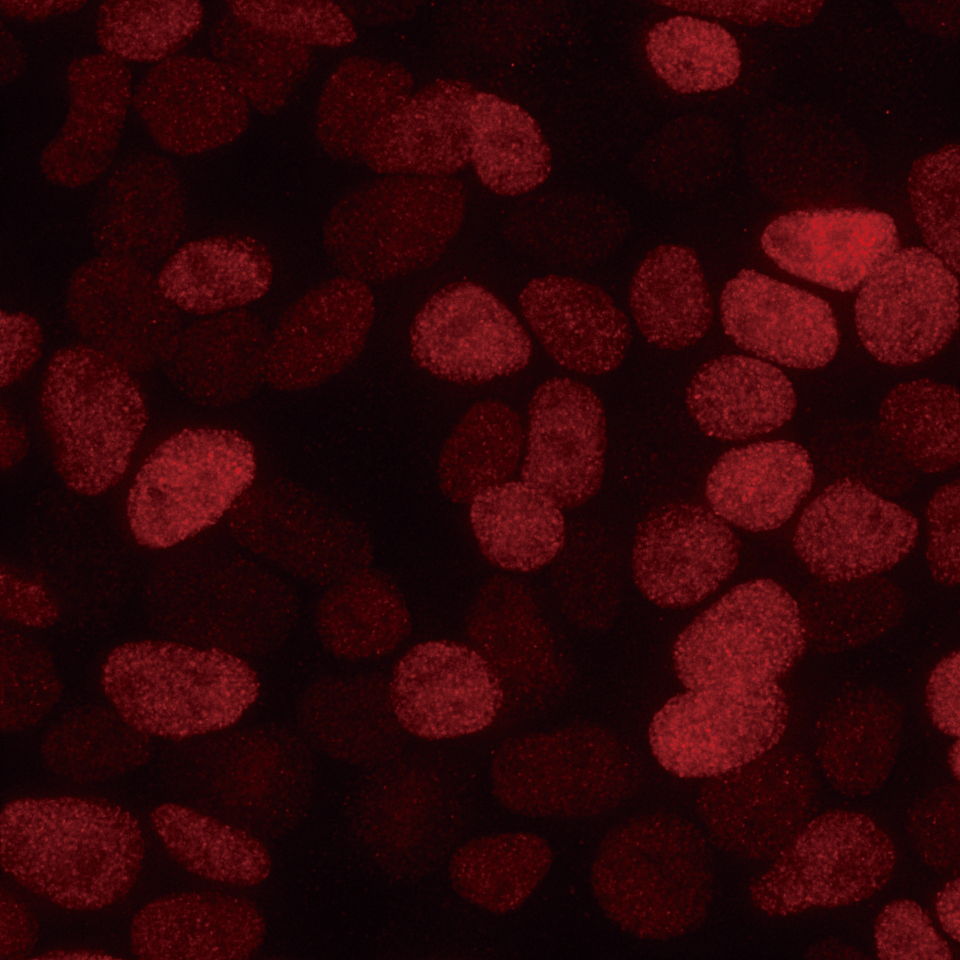

Supplement: Supplementary file 3 — Source data Fig. 1 [file 44318_2025_605_MOESM3_ESM.zip › Fig. 1/1B/PAX6/D3.tif]

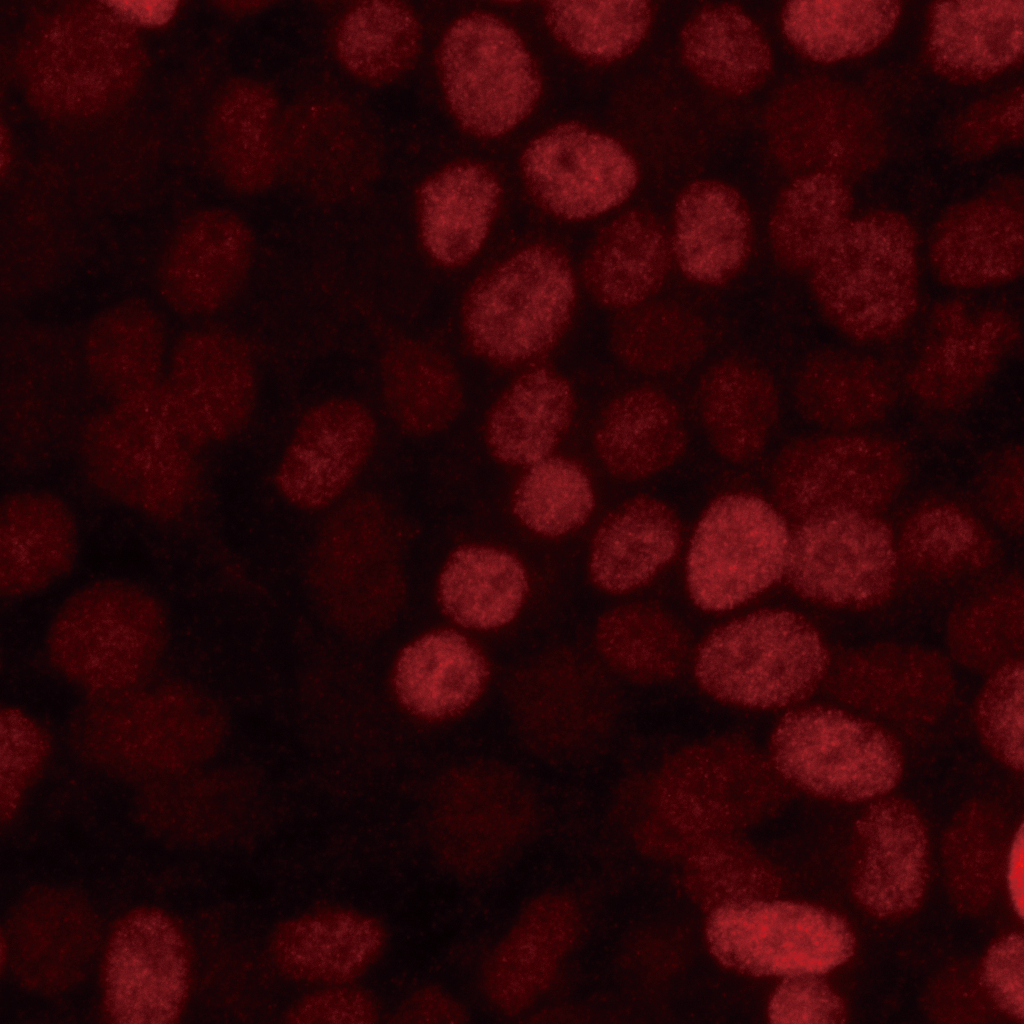

Supplement: Supplementary file 3 — Source data Fig. 1 [file 44318_2025_605_MOESM3_ESM.zip › Fig. 1/1B/PAX6/D4.tif]

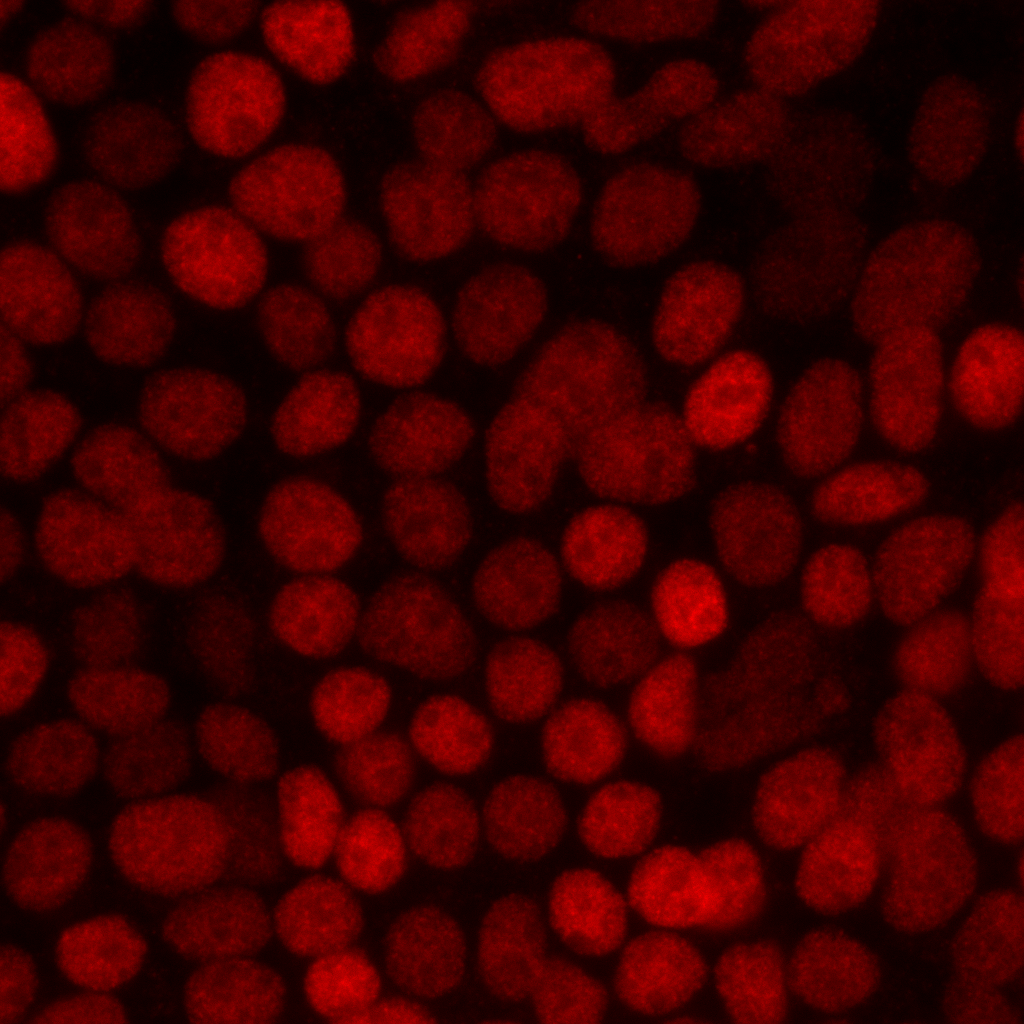

Supplement: Supplementary file 3 — Source data Fig. 1 [file 44318_2025_605_MOESM3_ESM.zip › Fig. 1/1B/PAX6/D5.tif]

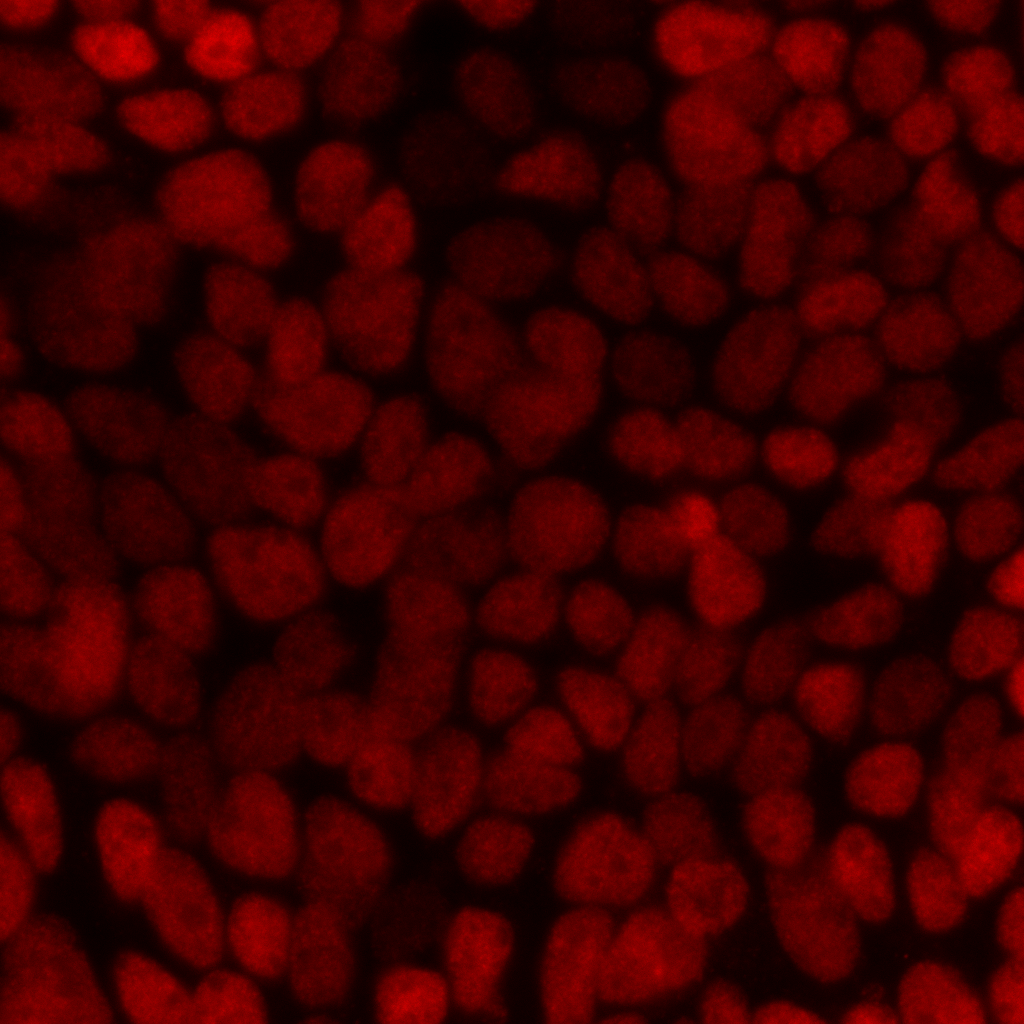

Supplement: Supplementary file 3 — Source data Fig. 1 [file 44318_2025_605_MOESM3_ESM.zip › Fig. 1/1B/PAX6/D6.tif]

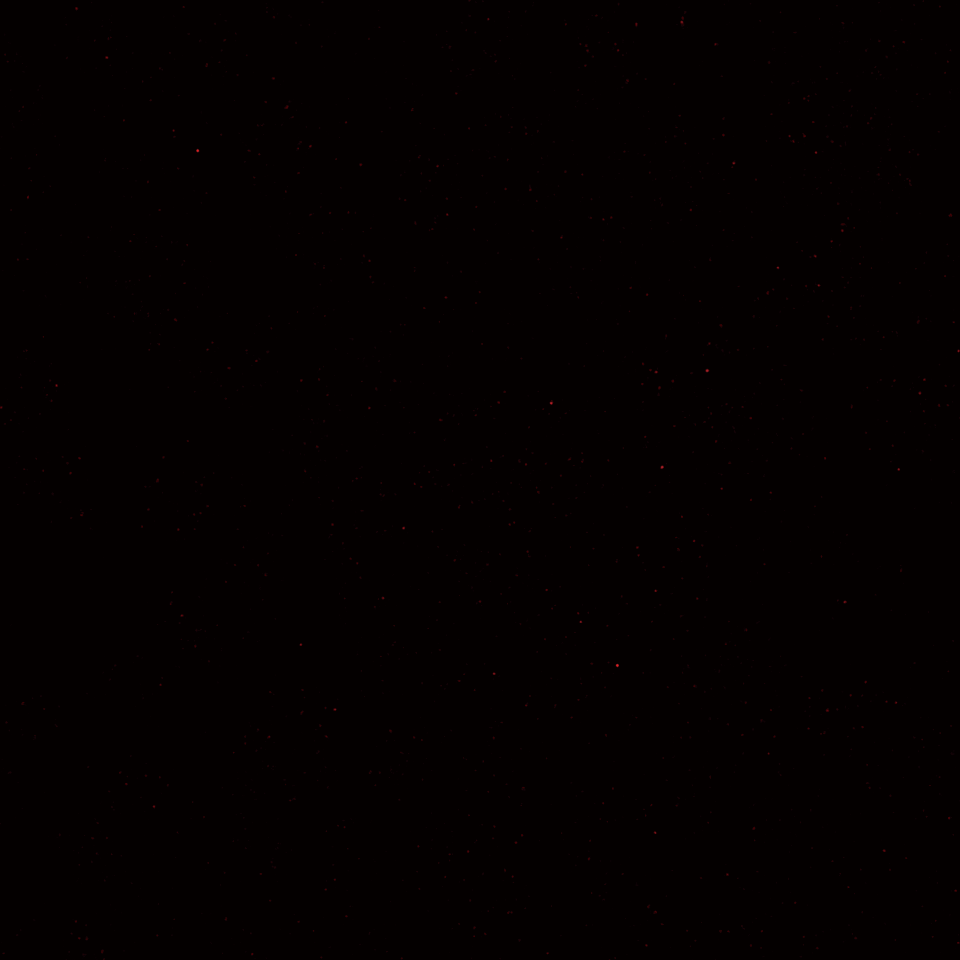

Supplement: Supplementary file 3 — Source data Fig. 1 [file 44318_2025_605_MOESM3_ESM.zip › Fig. 1/1B/PAX6/ESC.tif]

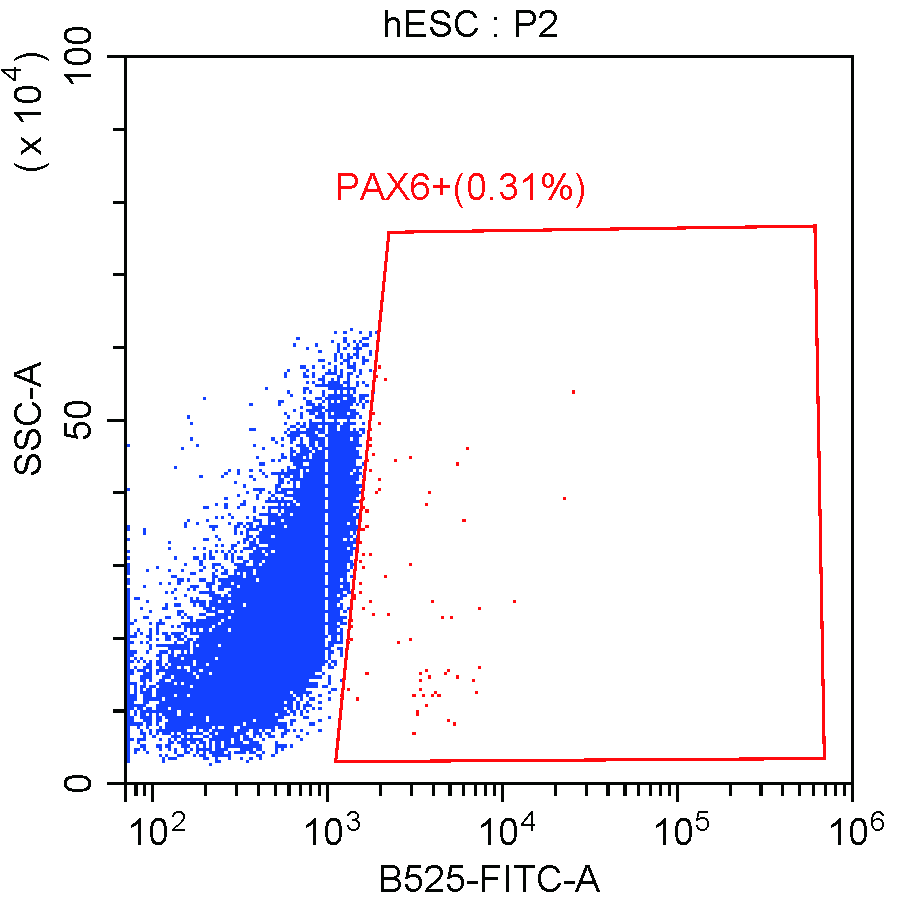

Supplement: Supplementary file 3 — Source data Fig. 1 [file 44318_2025_605_MOESM3_ESM.zip › Fig. 1/1C/Replication_1/ESC.tif]

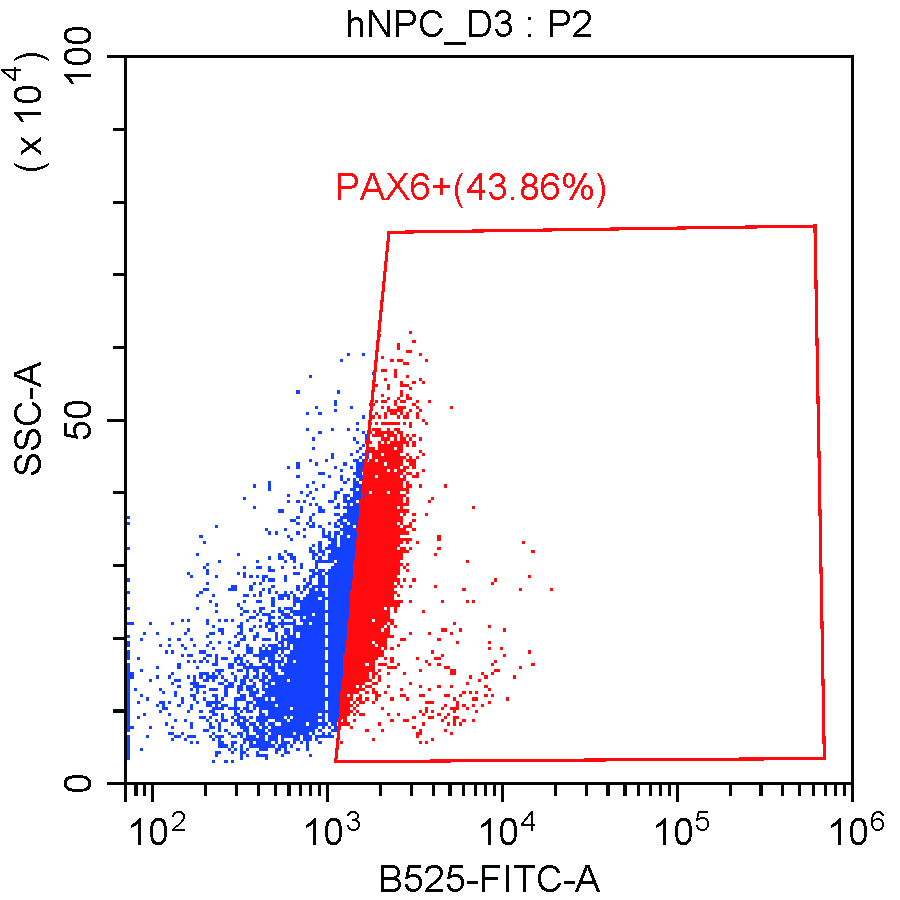

Supplement: Supplementary file 3 — Source data Fig. 1 [file 44318_2025_605_MOESM3_ESM.zip › Fig. 1/1C/Replication_1/hNPC_D3.tif]

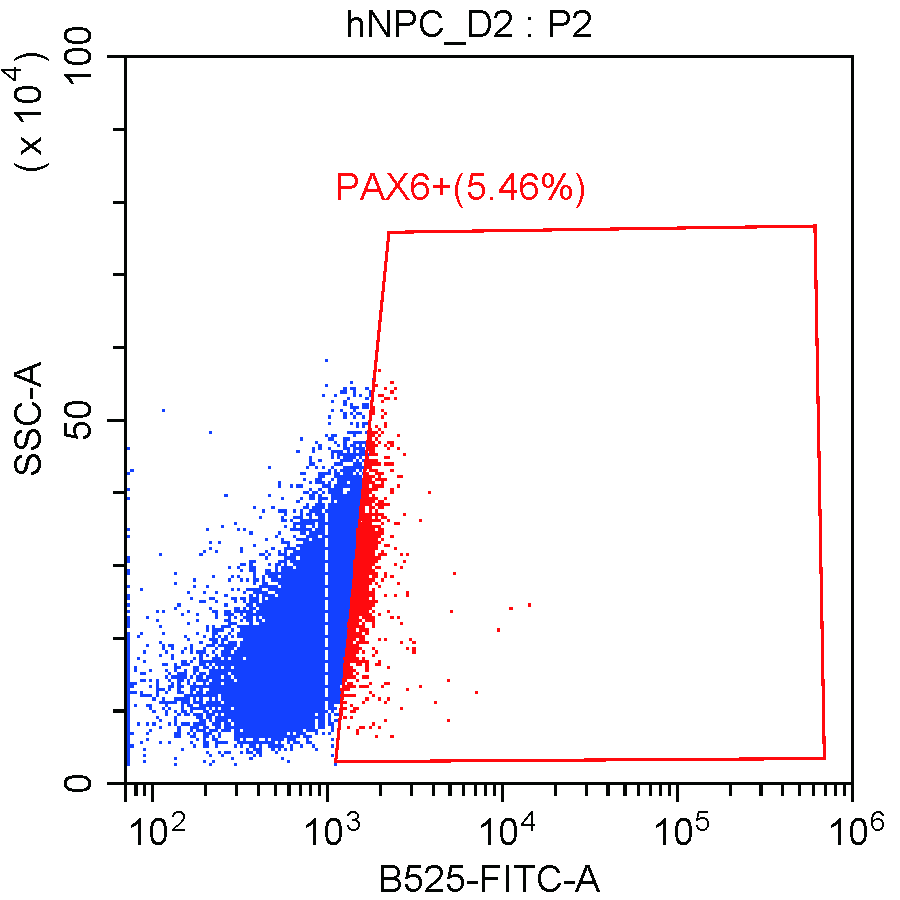

Supplement: Supplementary file 3 — Source data Fig. 1 [file 44318_2025_605_MOESM3_ESM.zip › Fig. 1/1C/Replication_1/NPC_D2.tif]

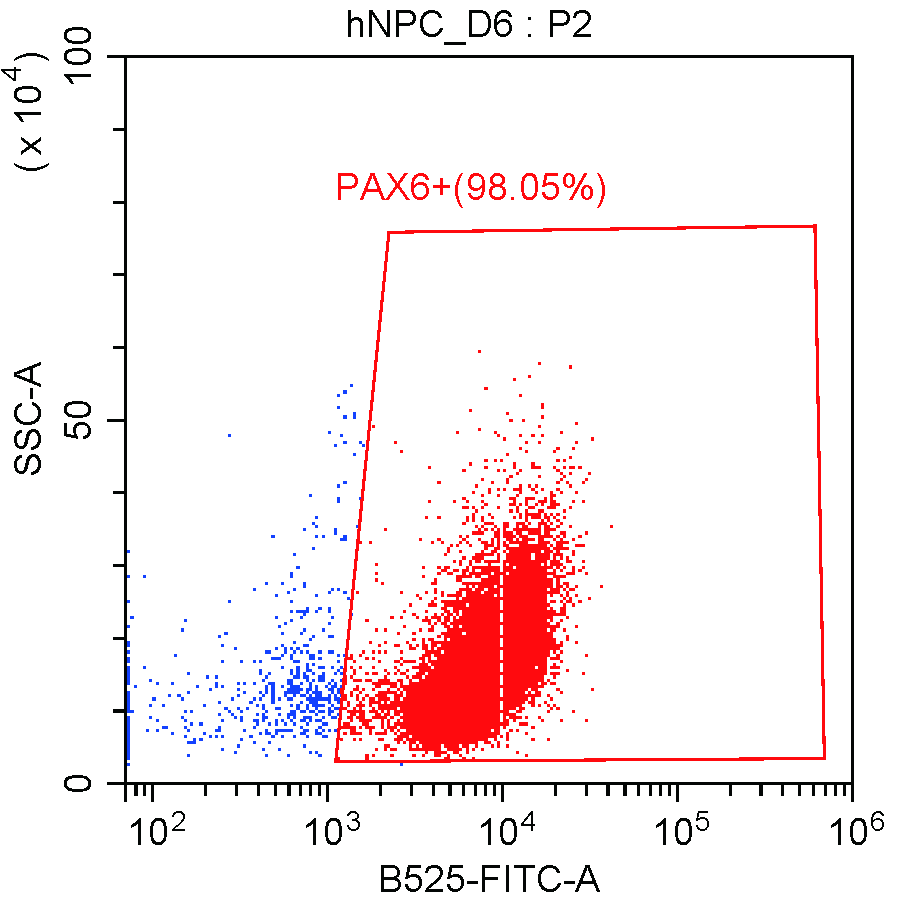

Supplement: Supplementary file 3 — Source data Fig. 1 [file 44318_2025_605_MOESM3_ESM.zip › Fig. 1/1C/Replication_1/NPC_D6.tif]

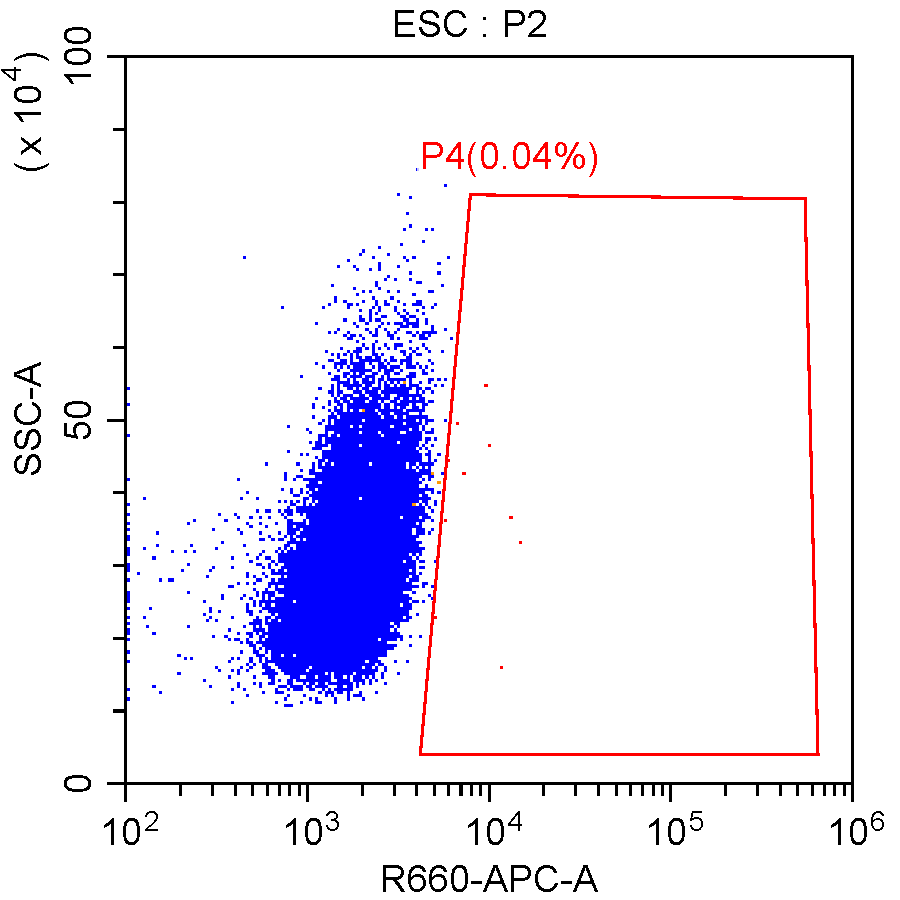

Supplement: Supplementary file 3 — Source data Fig. 1 [file 44318_2025_605_MOESM3_ESM.zip › Fig. 1/1C/Replication_2/ESC.tif]

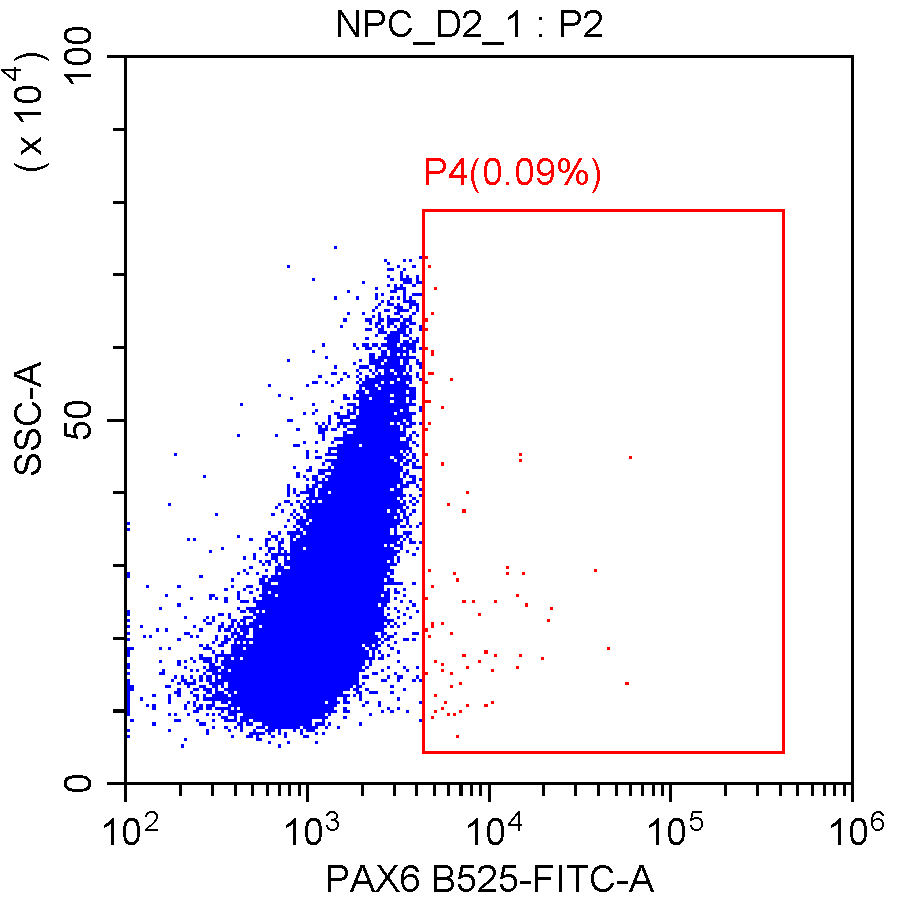

Supplement: Supplementary file 3 — Source data Fig. 1 [file 44318_2025_605_MOESM3_ESM.zip › Fig. 1/1C/Replication_2/NPC_D2.tiff]

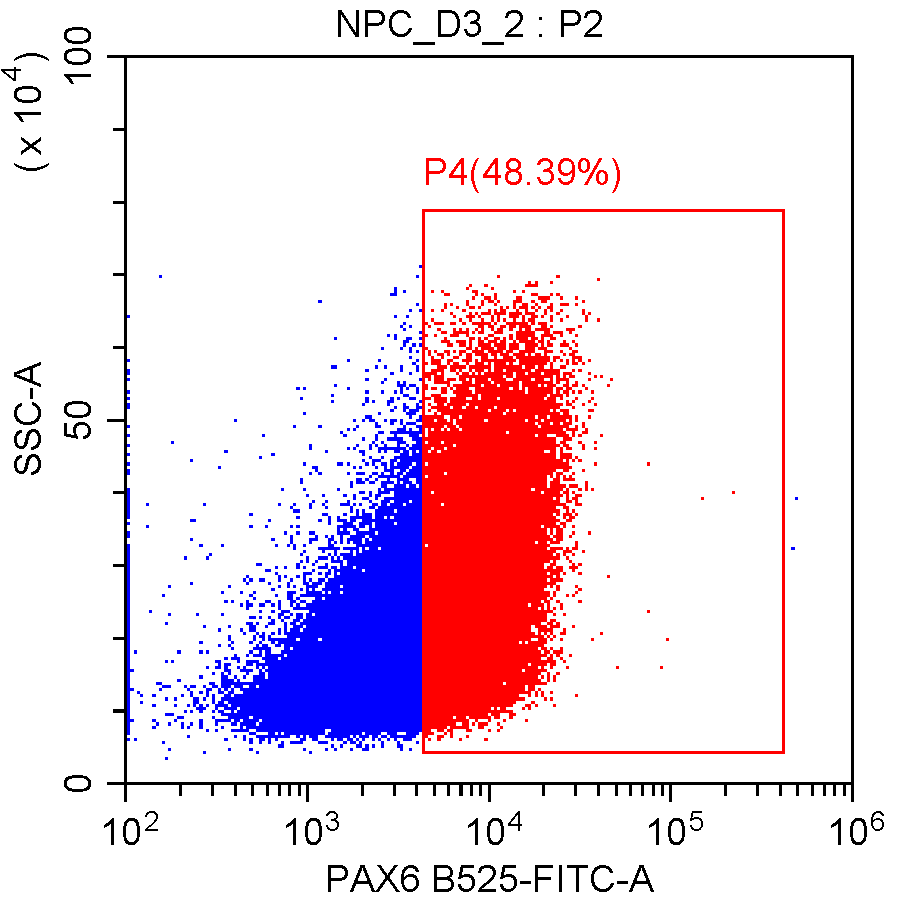

Supplement: Supplementary file 3 — Source data Fig. 1 [file 44318_2025_605_MOESM3_ESM.zip › Fig. 1/1C/Replication_2/NPC_D3.tif]

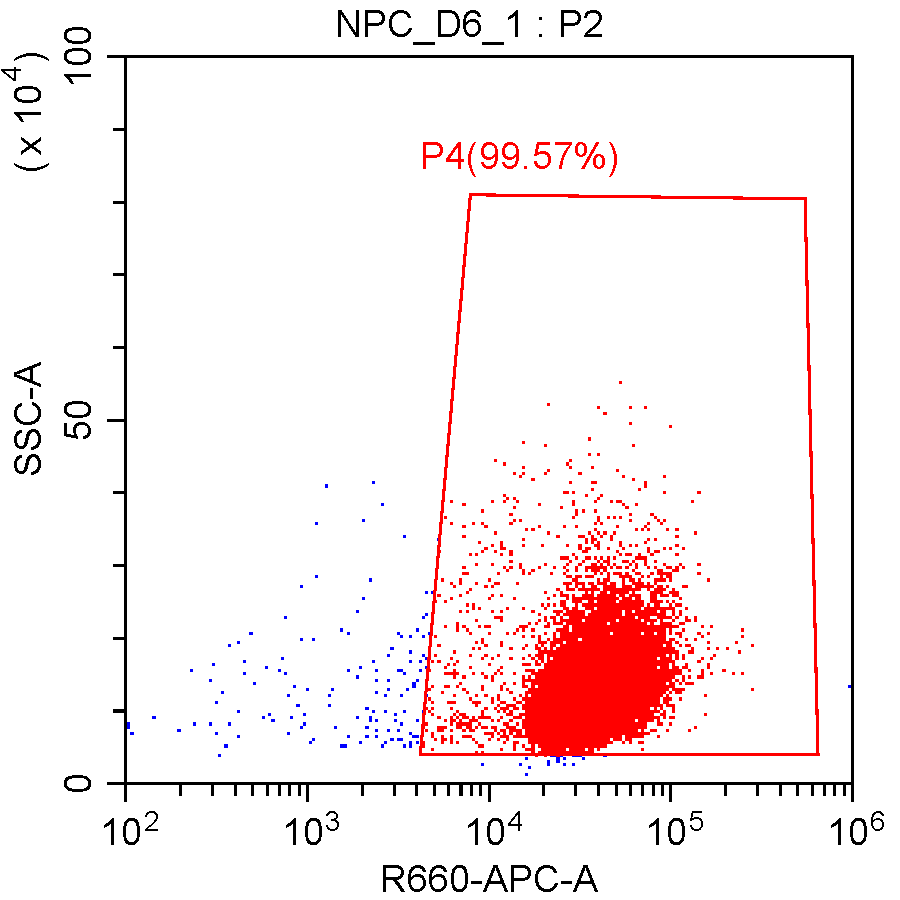

Supplement: Supplementary file 3 — Source data Fig. 1 [file 44318_2025_605_MOESM3_ESM.zip › Fig. 1/1C/Replication_2/NPC_D6.tiff]

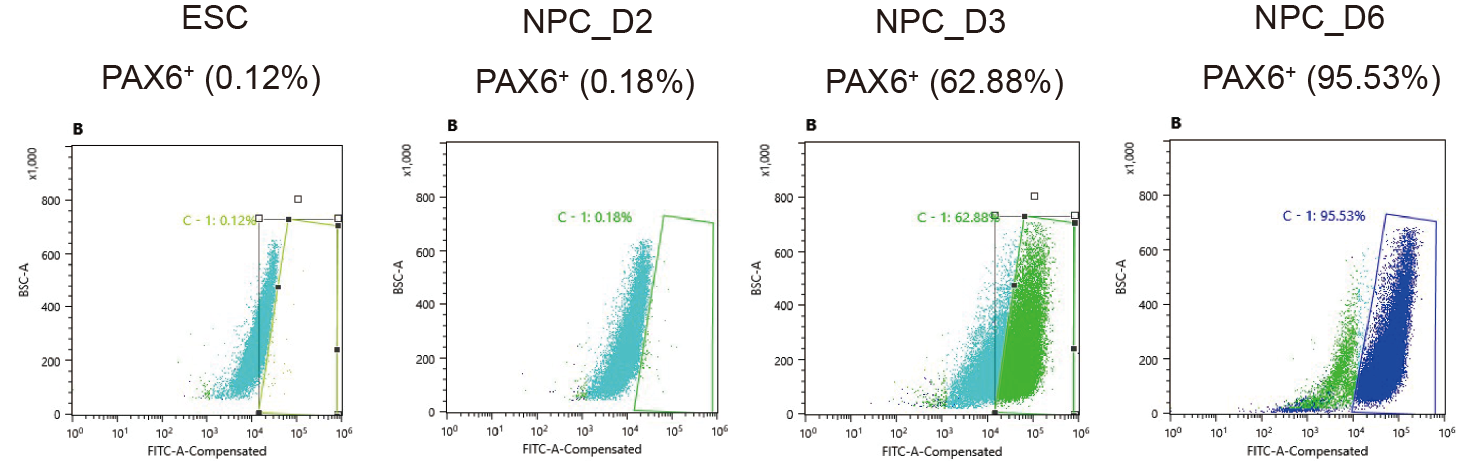

Supplement: Supplementary file 3 — Source data Fig. 1 [file 44318_2025_605_MOESM3_ESM.zip › Fig. 1/1C/Replication_3/PAX6+ cell_FACS replication 3.tif]

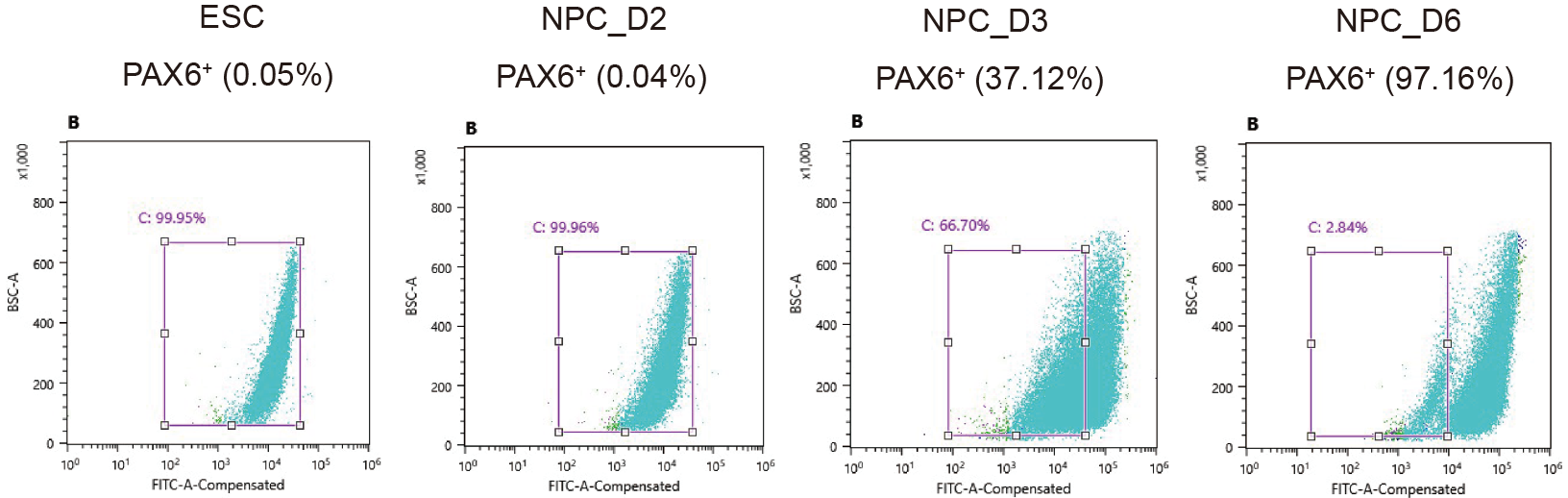

Supplement: Supplementary file 3 — Source data Fig. 1 [file 44318_2025_605_MOESM3_ESM.zip › Fig. 1/1C/Replication_4/PAX6+ cell_FACS replication 4.tif]

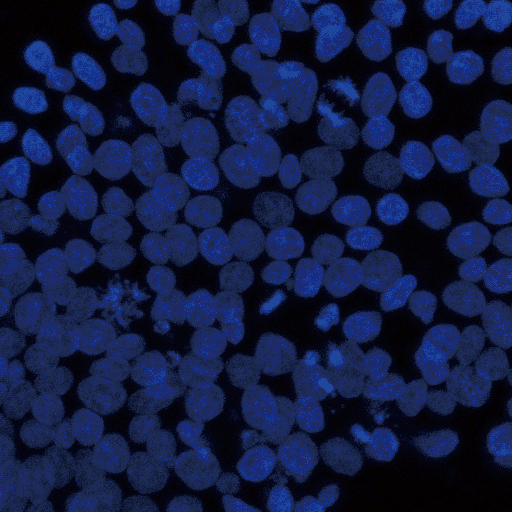

Supplement: Supplementary file 3 — Source data Fig. 1 [file 44318_2025_605_MOESM3_ESM.zip › Fig. 1/1E/D2/DAPI.tif]

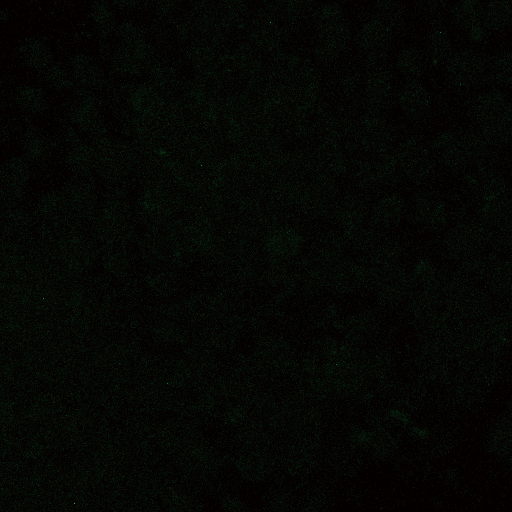

Supplement: Supplementary file 3 — Source data Fig. 1 [file 44318_2025_605_MOESM3_ESM.zip › Fig. 1/1E/D2/NANOG.tif]

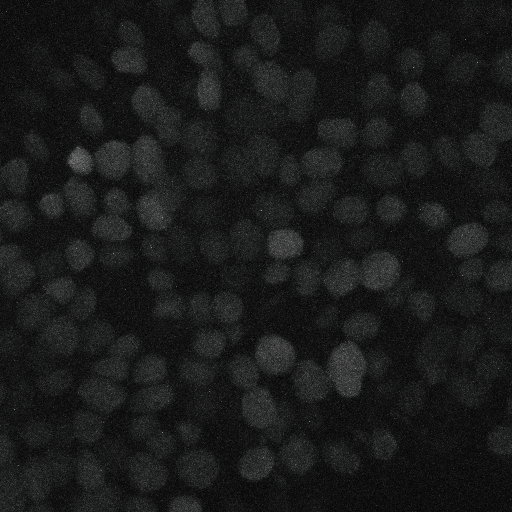

Supplement: Supplementary file 3 — Source data Fig. 1 [file 44318_2025_605_MOESM3_ESM.zip › Fig. 1/1E/D2/OCT4.tif]

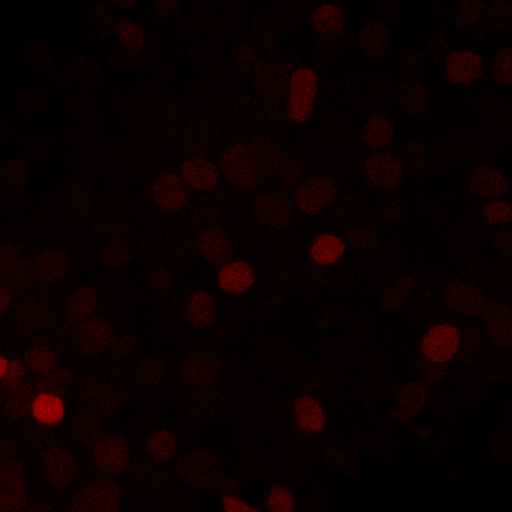

Supplement: Supplementary file 3 — Source data Fig. 1 [file 44318_2025_605_MOESM3_ESM.zip › Fig. 1/1E/D2/PAX6.tif]

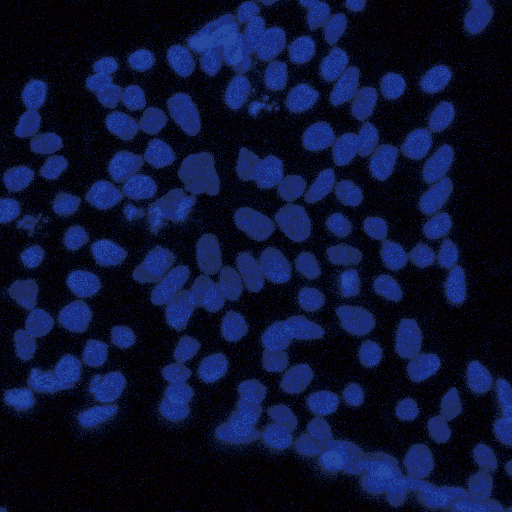

Supplement: Supplementary file 3 — Source data Fig. 1 [file 44318_2025_605_MOESM3_ESM.zip › Fig. 1/1E/D3/DAPI.tif]

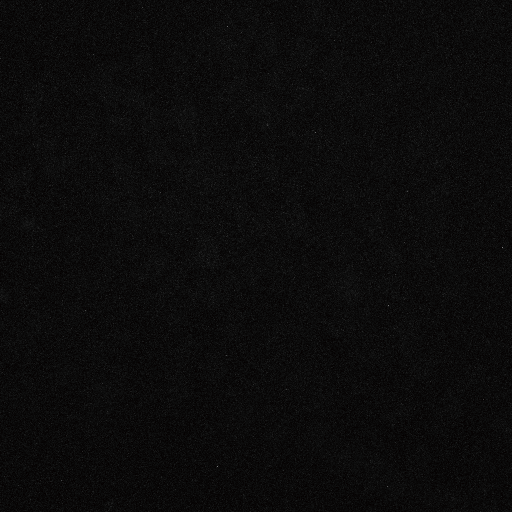

Supplement: Supplementary file 3 — Source data Fig. 1 [file 44318_2025_605_MOESM3_ESM.zip › Fig. 1/1E/D3/NANOG.tif]

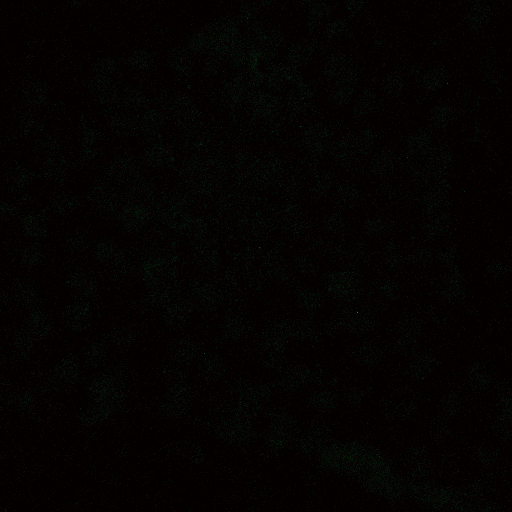

Supplement: Supplementary file 3 — Source data Fig. 1 [file 44318_2025_605_MOESM3_ESM.zip › Fig. 1/1E/D3/OCT4.tif]

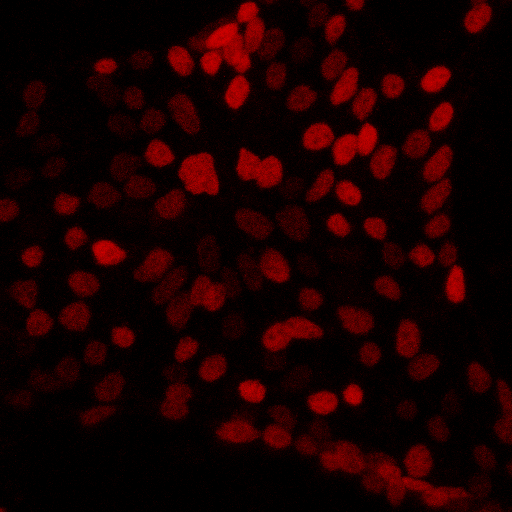

Supplement: Supplementary file 3 — Source data Fig. 1 [file 44318_2025_605_MOESM3_ESM.zip › Fig. 1/1E/D3/PAX6.tif]

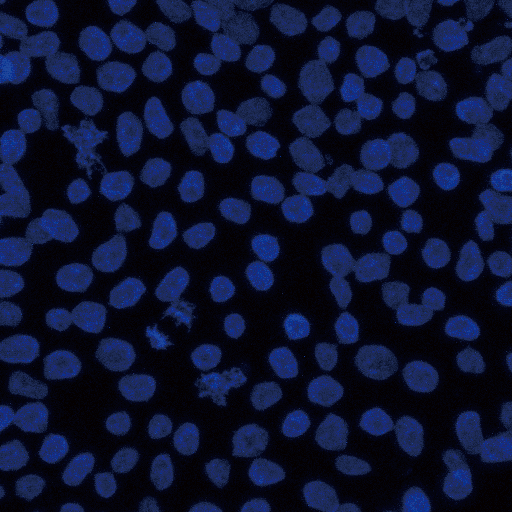

Supplement: Supplementary file 3 — Source data Fig. 1 [file 44318_2025_605_MOESM3_ESM.zip › Fig. 1/1E/ESC/DAPI.tif]

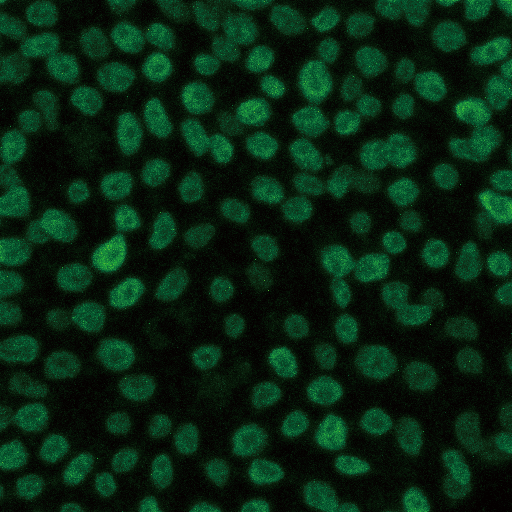

Supplement: Supplementary file 3 — Source data Fig. 1 [file 44318_2025_605_MOESM3_ESM.zip › Fig. 1/1E/ESC/NANOG.tif]

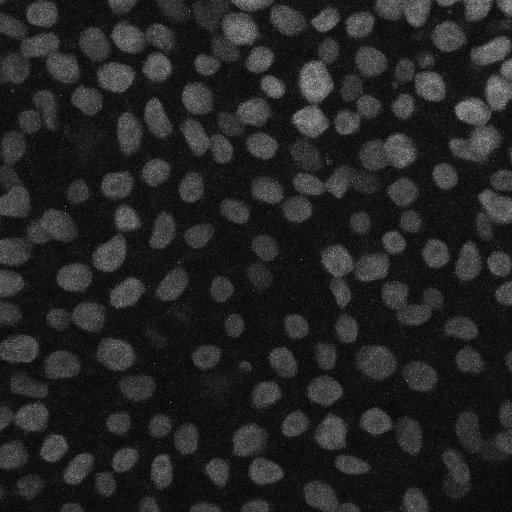

Supplement: Supplementary file 3 — Source data Fig. 1 [file 44318_2025_605_MOESM3_ESM.zip › Fig. 1/1E/ESC/OCT4.tif]

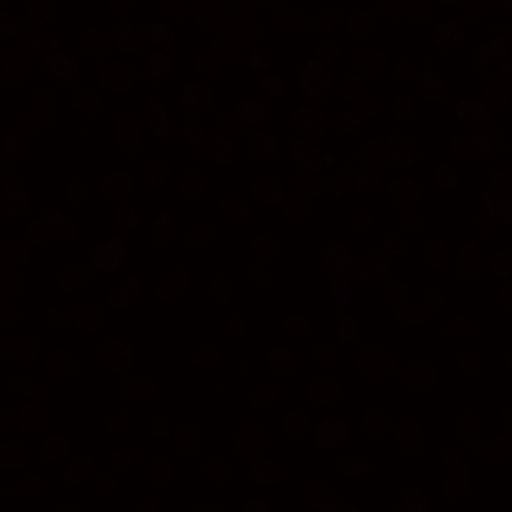

Supplement: Supplementary file 3 — Source data Fig. 1 [file 44318_2025_605_MOESM3_ESM.zip › Fig. 1/1E/ESC/PAX6.tif]

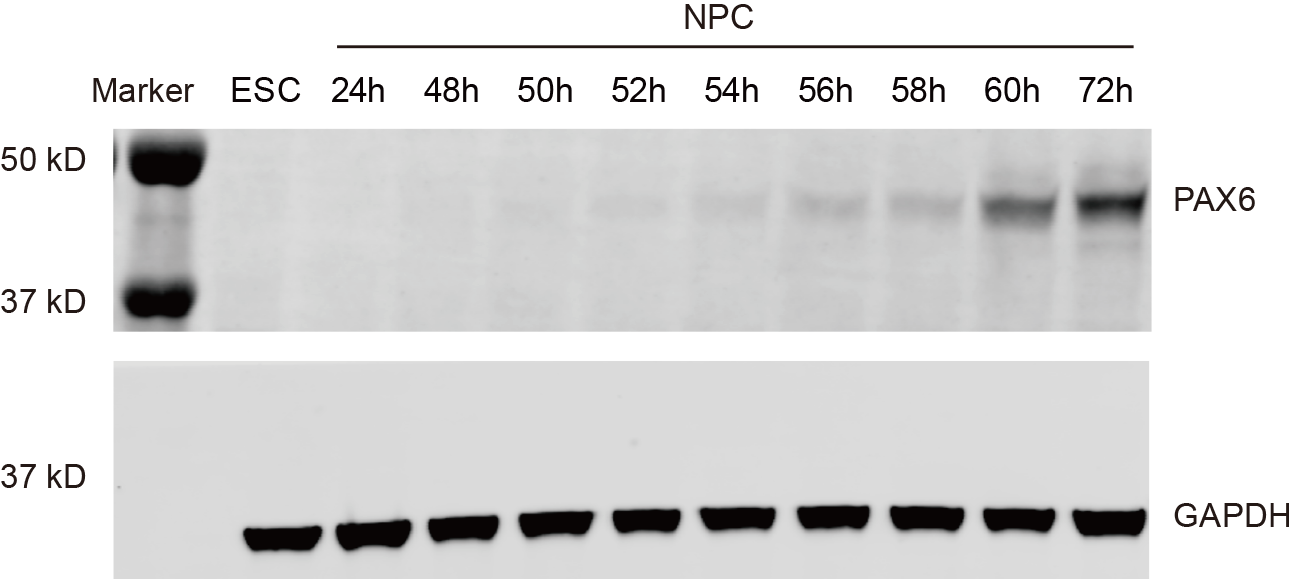

Supplement: Supplementary file 4 — Source data Fig. 2 [file 44318_2025_605_MOESM4_ESM.zip › Fig. 2/2A/2A.tif]

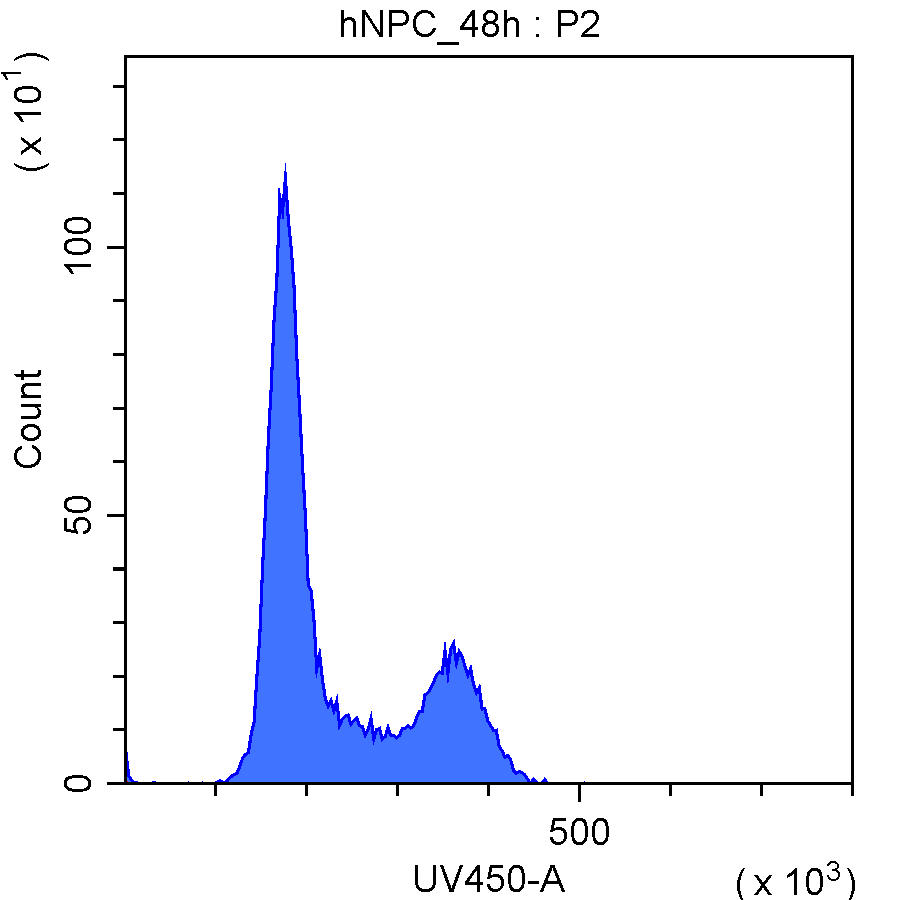

Supplement: Supplementary file 4 — Source data Fig. 2 [file 44318_2025_605_MOESM4_ESM.zip › Fig. 2/2B/Neural induction_48h_72h/DAPI_NPC_WT_48h_72h/hNPC_48h_Plot1.bmp]

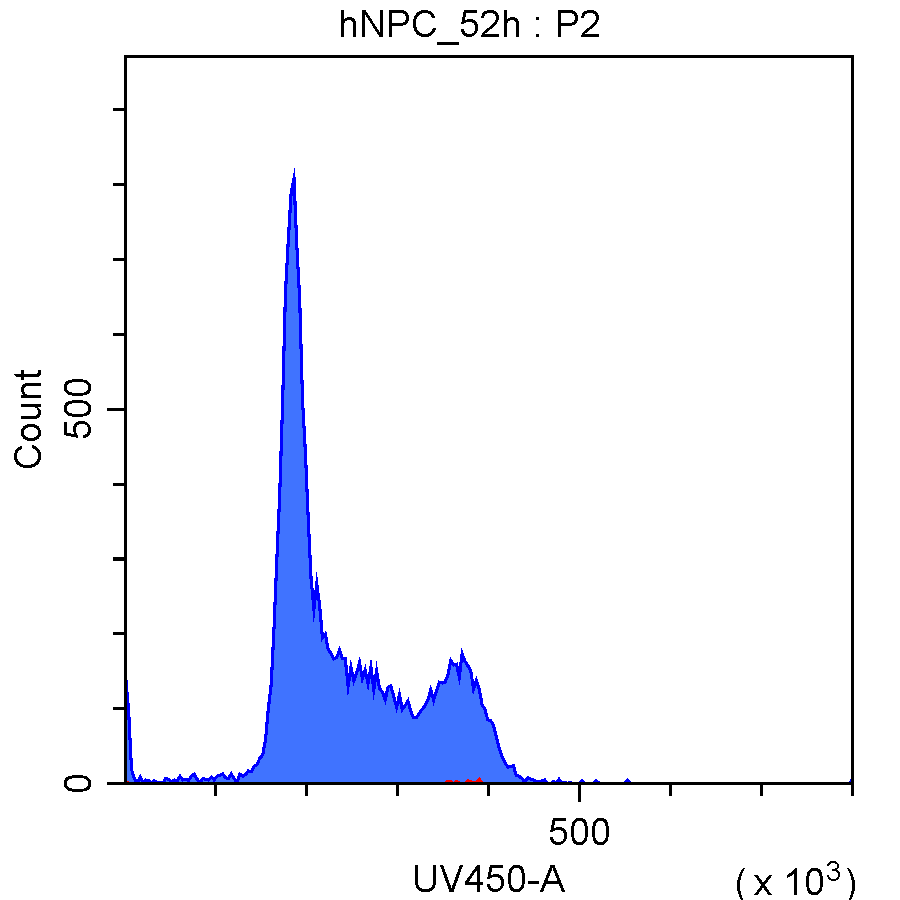

Supplement: Supplementary file 4 — Source data Fig. 2 [file 44318_2025_605_MOESM4_ESM.zip › Fig. 2/2B/Neural induction_48h_72h/DAPI_NPC_WT_48h_72h/hNPC_52h_Plot1.bmp]

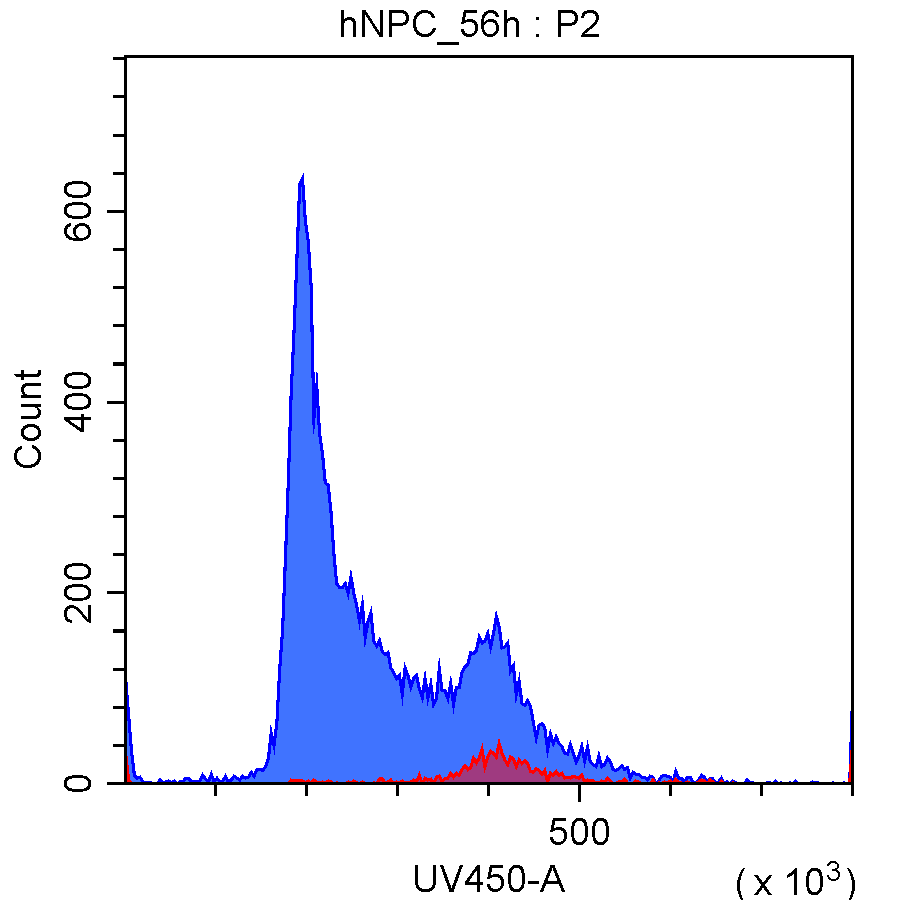

Supplement: Supplementary file 4 — Source data Fig. 2 [file 44318_2025_605_MOESM4_ESM.zip › Fig. 2/2B/Neural induction_48h_72h/DAPI_NPC_WT_48h_72h/hNPC_56h_Plot1.bmp]

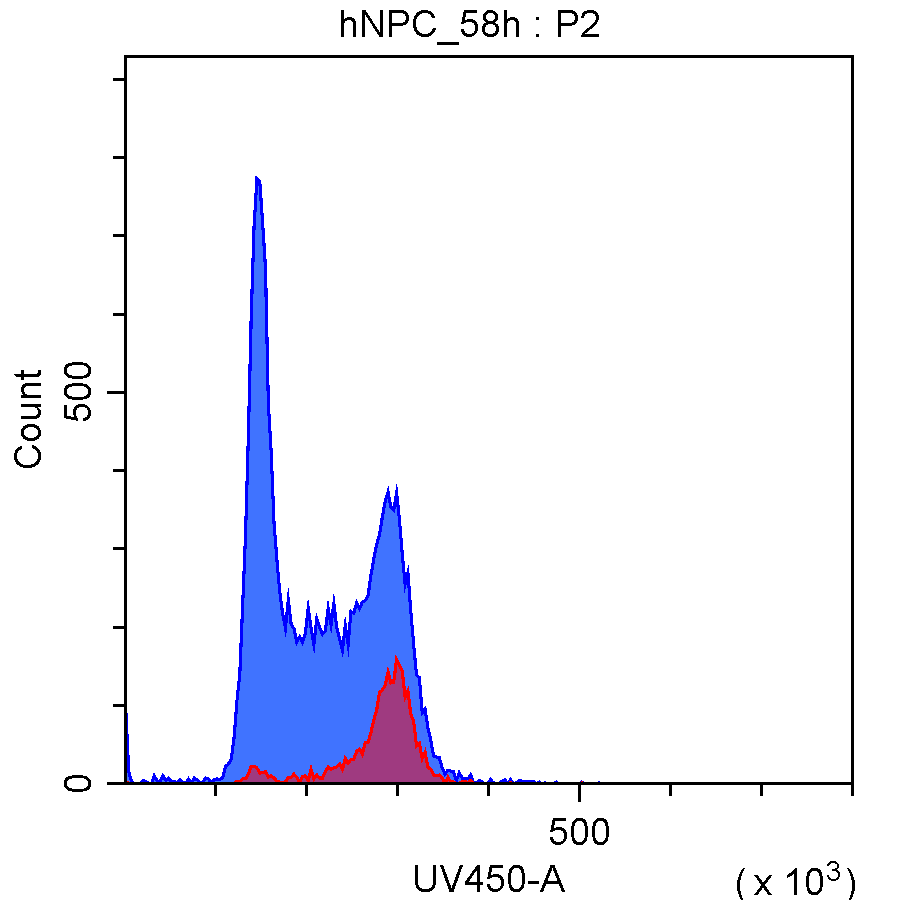

Supplement: Supplementary file 4 — Source data Fig. 2 [file 44318_2025_605_MOESM4_ESM.zip › Fig. 2/2B/Neural induction_48h_72h/DAPI_NPC_WT_48h_72h/hNPC_58h_Plot1.bmp]

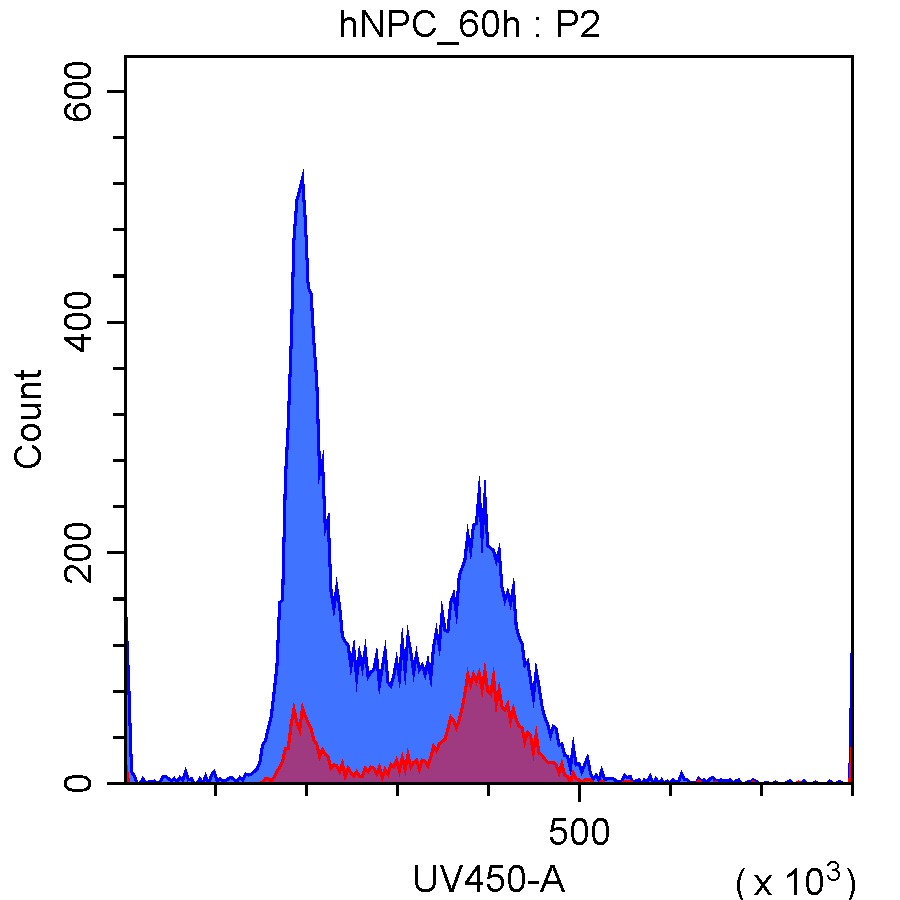

Supplement: Supplementary file 4 — Source data Fig. 2 [file 44318_2025_605_MOESM4_ESM.zip › Fig. 2/2B/Neural induction_48h_72h/DAPI_NPC_WT_48h_72h/hNPC_60h_Plot1.bmp]

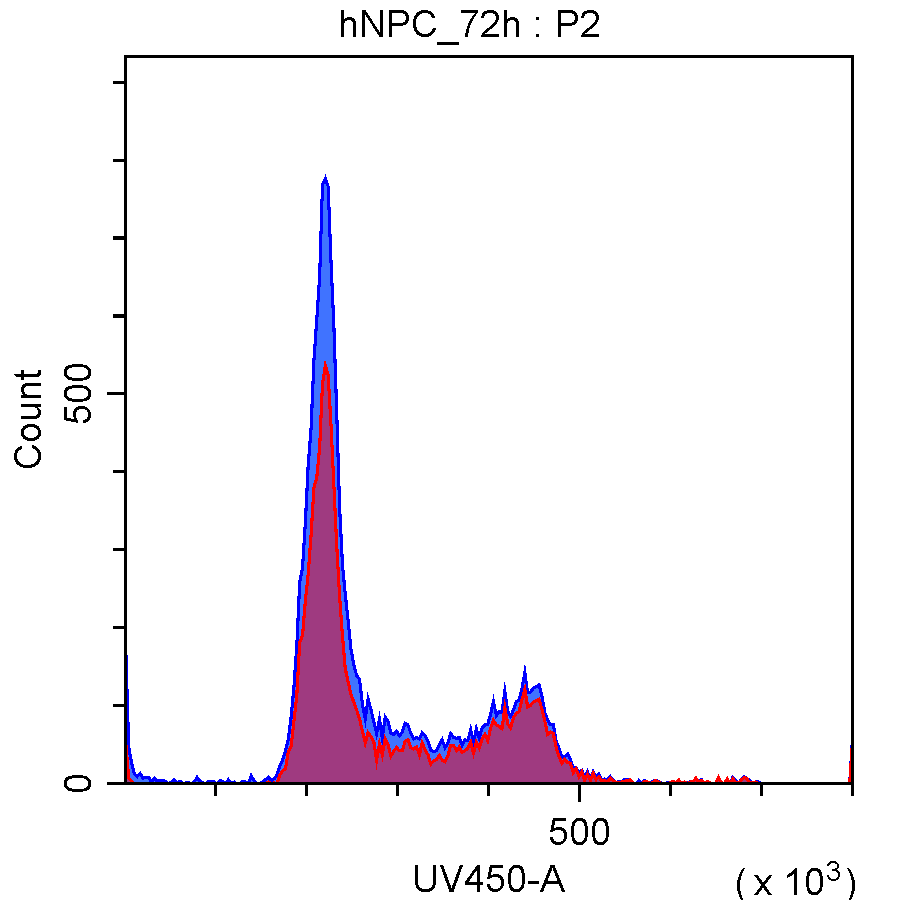

Supplement: Supplementary file 4 — Source data Fig. 2 [file 44318_2025_605_MOESM4_ESM.zip › Fig. 2/2B/Neural induction_48h_72h/DAPI_NPC_WT_48h_72h/hNPC_72h_Plot1.bmp]

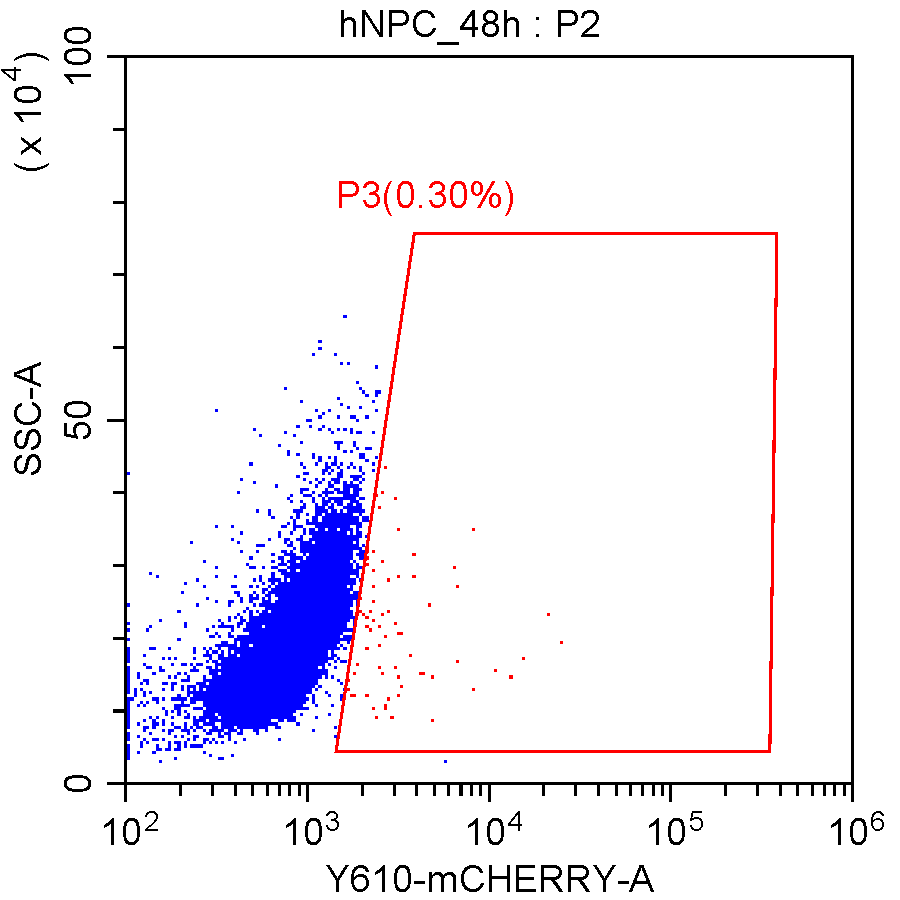

Supplement: Supplementary file 4 — Source data Fig. 2 [file 44318_2025_605_MOESM4_ESM.zip › Fig. 2/2B/Neural induction_48h_72h/PAX6_NPC_WT_48h_72h/hNPC_48h_Plot1.bmp]

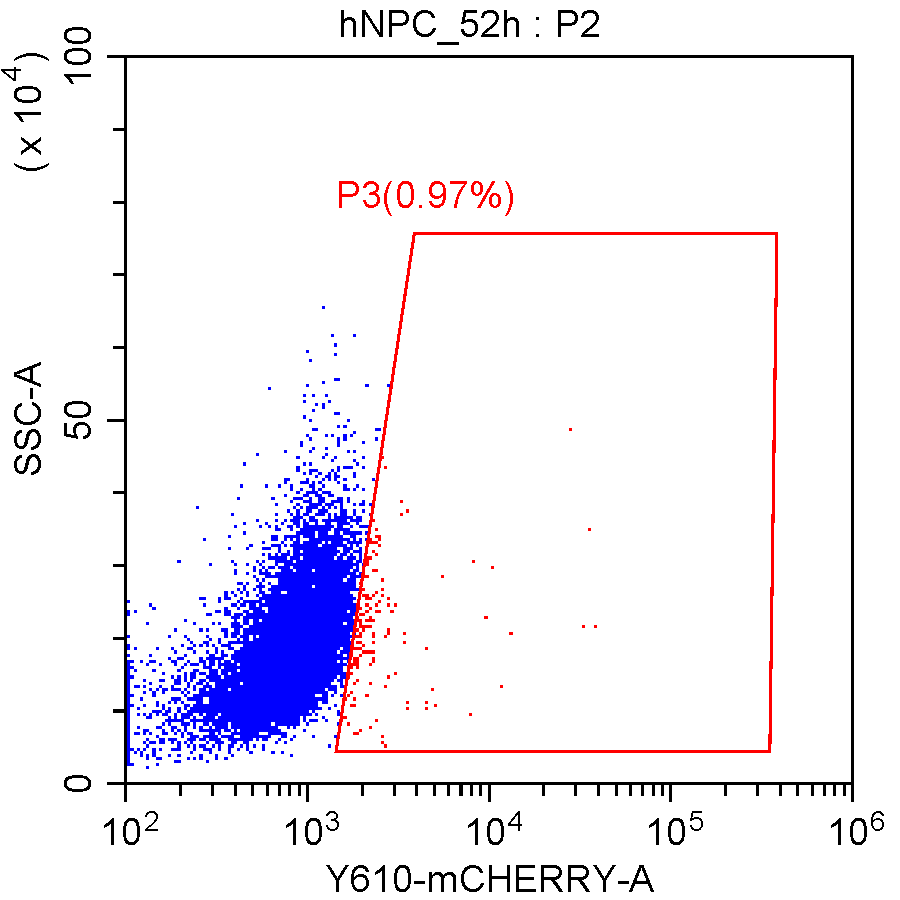

Supplement: Supplementary file 4 — Source data Fig. 2 [file 44318_2025_605_MOESM4_ESM.zip › Fig. 2/2B/Neural induction_48h_72h/PAX6_NPC_WT_48h_72h/hNPC_52h_Plot1.bmp]

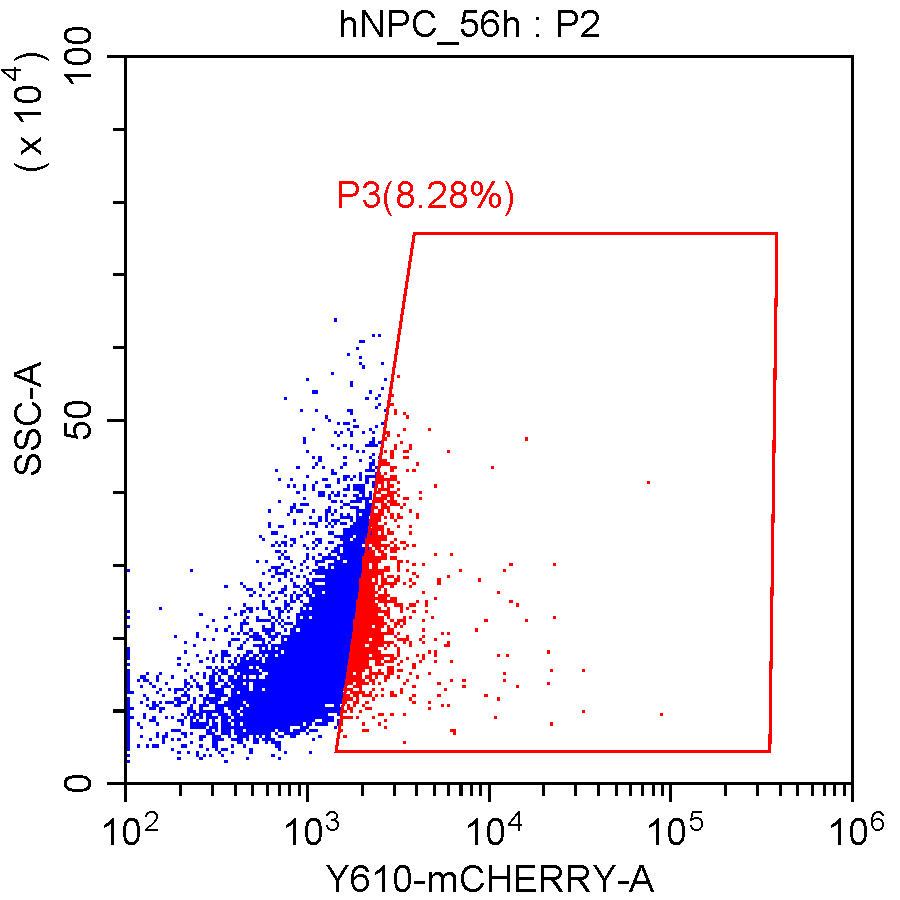

Supplement: Supplementary file 4 — Source data Fig. 2 [file 44318_2025_605_MOESM4_ESM.zip › Fig. 2/2B/Neural induction_48h_72h/PAX6_NPC_WT_48h_72h/hNPC_56h_Plot1.bmp]

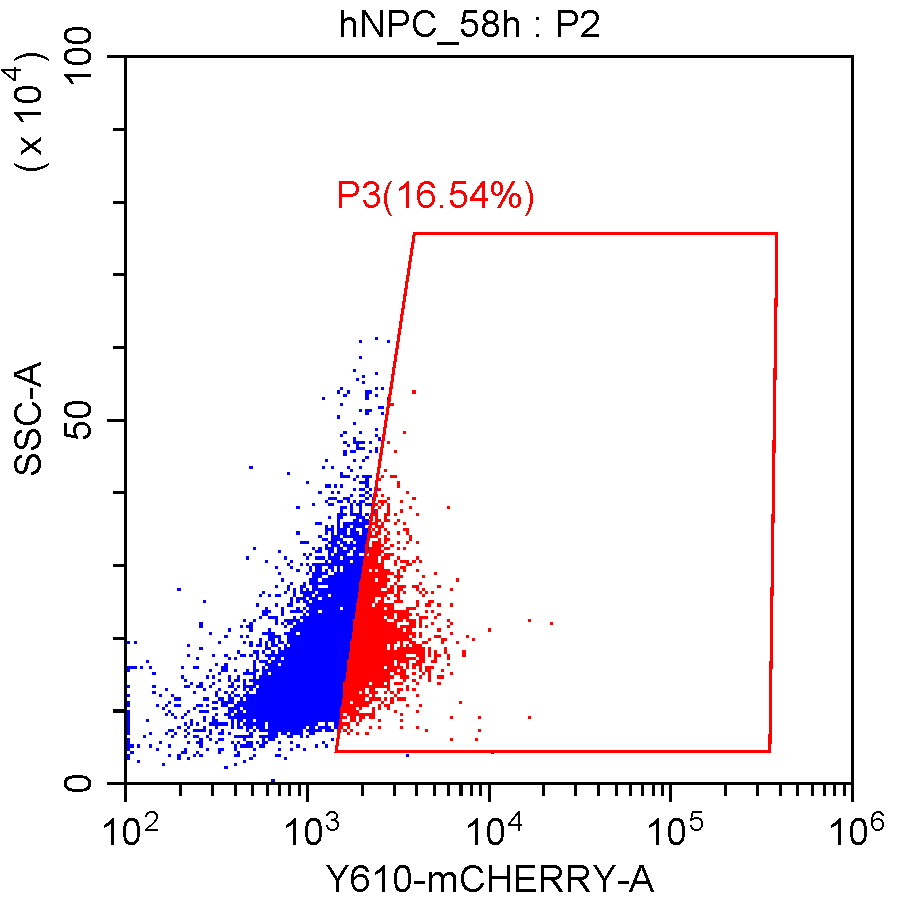

Supplement: Supplementary file 4 — Source data Fig. 2 [file 44318_2025_605_MOESM4_ESM.zip › Fig. 2/2B/Neural induction_48h_72h/PAX6_NPC_WT_48h_72h/hNPC_58h_Plot1.bmp]

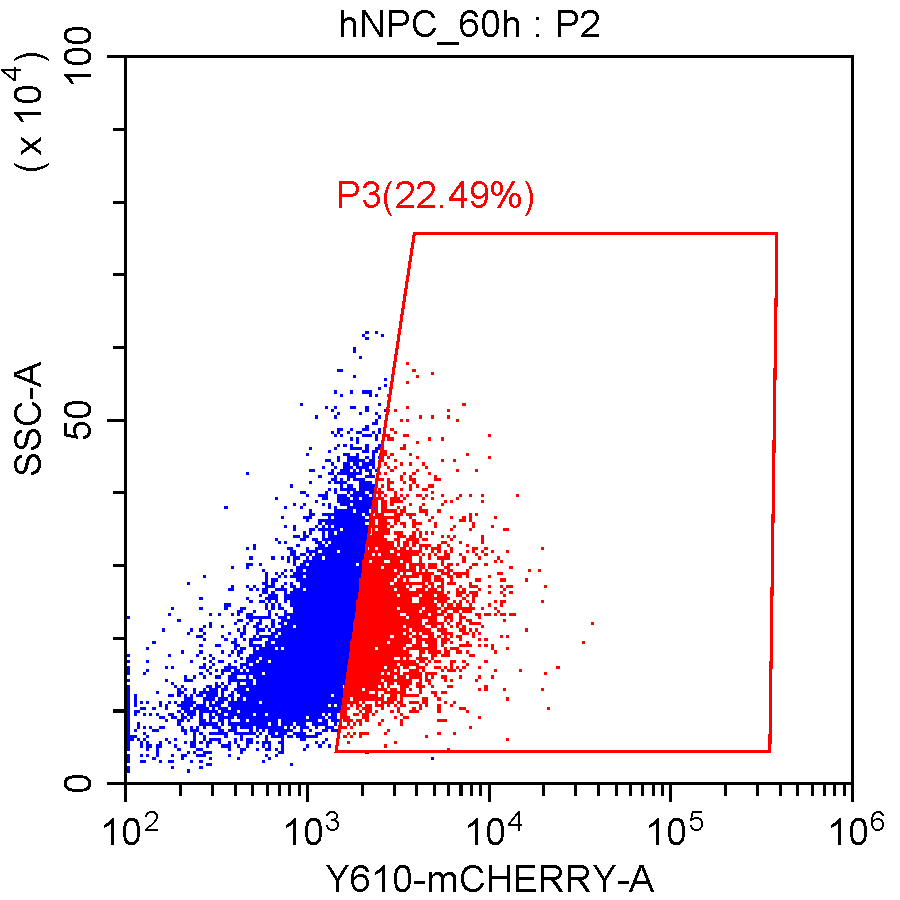

Supplement: Supplementary file 4 — Source data Fig. 2 [file 44318_2025_605_MOESM4_ESM.zip › Fig. 2/2B/Neural induction_48h_72h/PAX6_NPC_WT_48h_72h/hNPC_60h_Plot1.bmp]

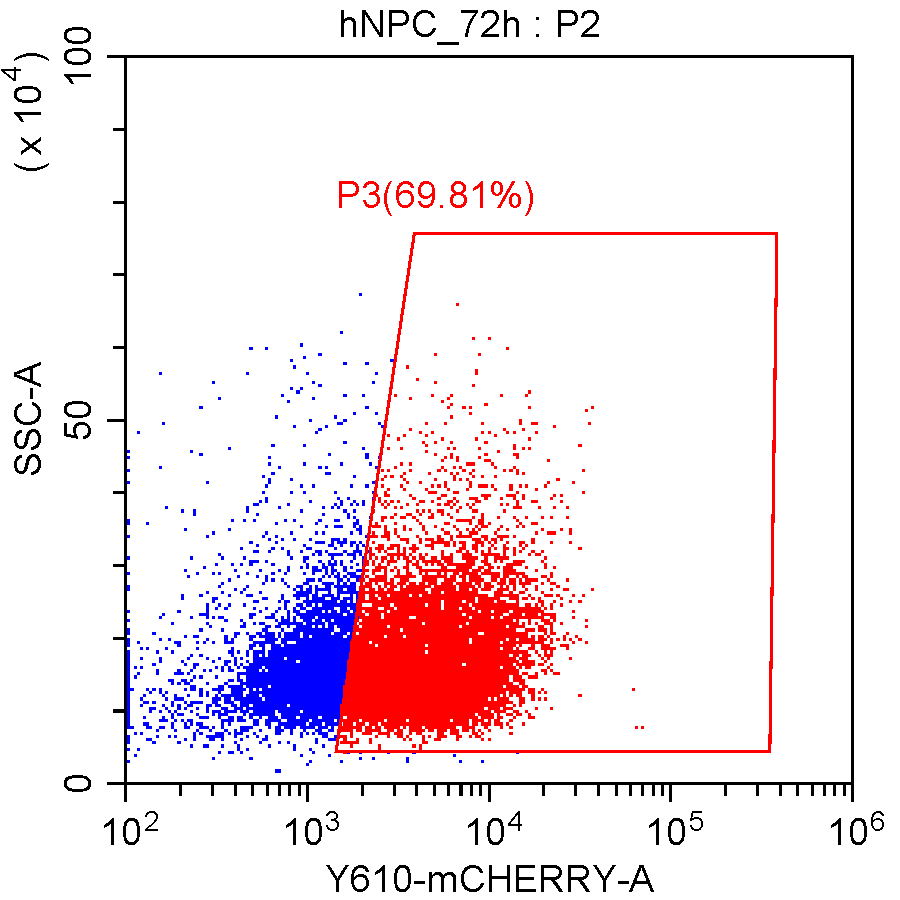

Supplement: Supplementary file 4 — Source data Fig. 2 [file 44318_2025_605_MOESM4_ESM.zip › Fig. 2/2B/Neural induction_48h_72h/PAX6_NPC_WT_48h_72h/hNPC_72h_Plot1.bmp]

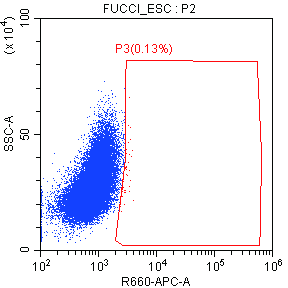

Supplement: Supplementary file 5 — Source data Fig. 3 [file 44318_2025_605_MOESM5_ESM.zip › Fig. 3/3C/Replicate_1/ESC/Ctrl.tif]

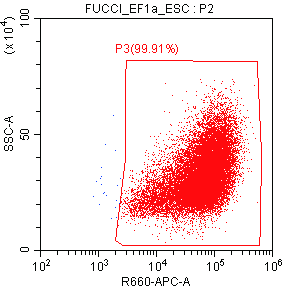

Supplement: Supplementary file 5 — Source data Fig. 3 [file 44318_2025_605_MOESM5_ESM.zip › Fig. 3/3C/Replicate_1/ESC/EF1α.tif]

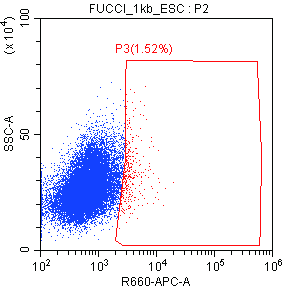

Supplement: Supplementary file 5 — Source data Fig. 3 [file 44318_2025_605_MOESM5_ESM.zip › Fig. 3/3C/Replicate_1/ESC/P1000.tif]

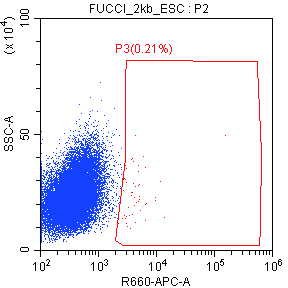

Supplement: Supplementary file 5 — Source data Fig. 3 [file 44318_2025_605_MOESM5_ESM.zip › Fig. 3/3C/Replicate_1/ESC/P2000.tif]

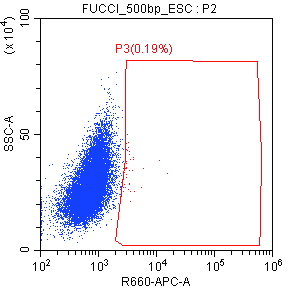

Supplement: Supplementary file 5 — Source data Fig. 3 [file 44318_2025_605_MOESM5_ESM.zip › Fig. 3/3C/Replicate_1/ESC/P500.tif]

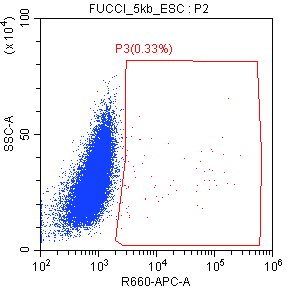

Supplement: Supplementary file 5 — Source data Fig. 3 [file 44318_2025_605_MOESM5_ESM.zip › Fig. 3/3C/Replicate_1/ESC/P5000.tif]

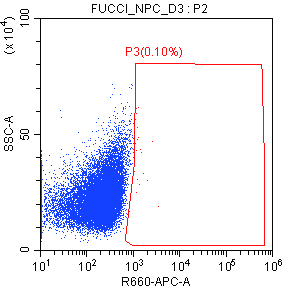

Supplement: Supplementary file 5 — Source data Fig. 3 [file 44318_2025_605_MOESM5_ESM.zip › Fig. 3/3C/Replicate_1/NPC_D3/Ctrl.tif]

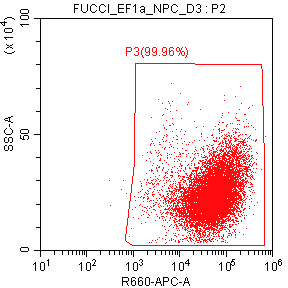

Supplement: Supplementary file 5 — Source data Fig. 3 [file 44318_2025_605_MOESM5_ESM.zip › Fig. 3/3C/Replicate_1/NPC_D3/EF1α.tif]

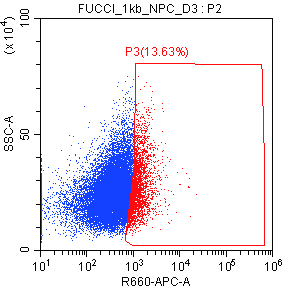

Supplement: Supplementary file 5 — Source data Fig. 3 [file 44318_2025_605_MOESM5_ESM.zip › Fig. 3/3C/Replicate_1/NPC_D3/P1000.tif]

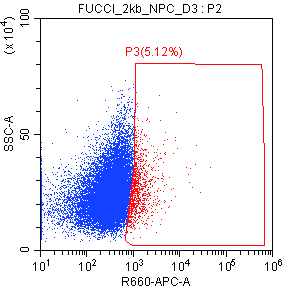

Supplement: Supplementary file 5 — Source data Fig. 3 [file 44318_2025_605_MOESM5_ESM.zip › Fig. 3/3C/Replicate_1/NPC_D3/P2000.tif]

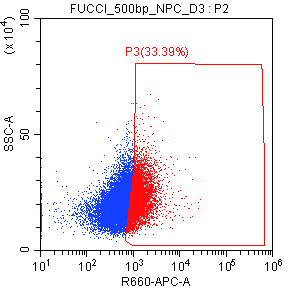

Supplement: Supplementary file 5 — Source data Fig. 3 [file 44318_2025_605_MOESM5_ESM.zip › Fig. 3/3C/Replicate_1/NPC_D3/P500.tif]

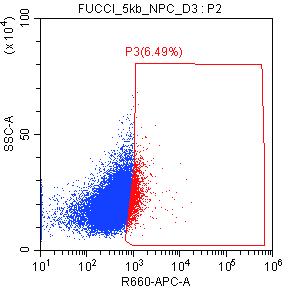

Supplement: Supplementary file 5 — Source data Fig. 3 [file 44318_2025_605_MOESM5_ESM.zip › Fig. 3/3C/Replicate_1/NPC_D3/P5000.tif]

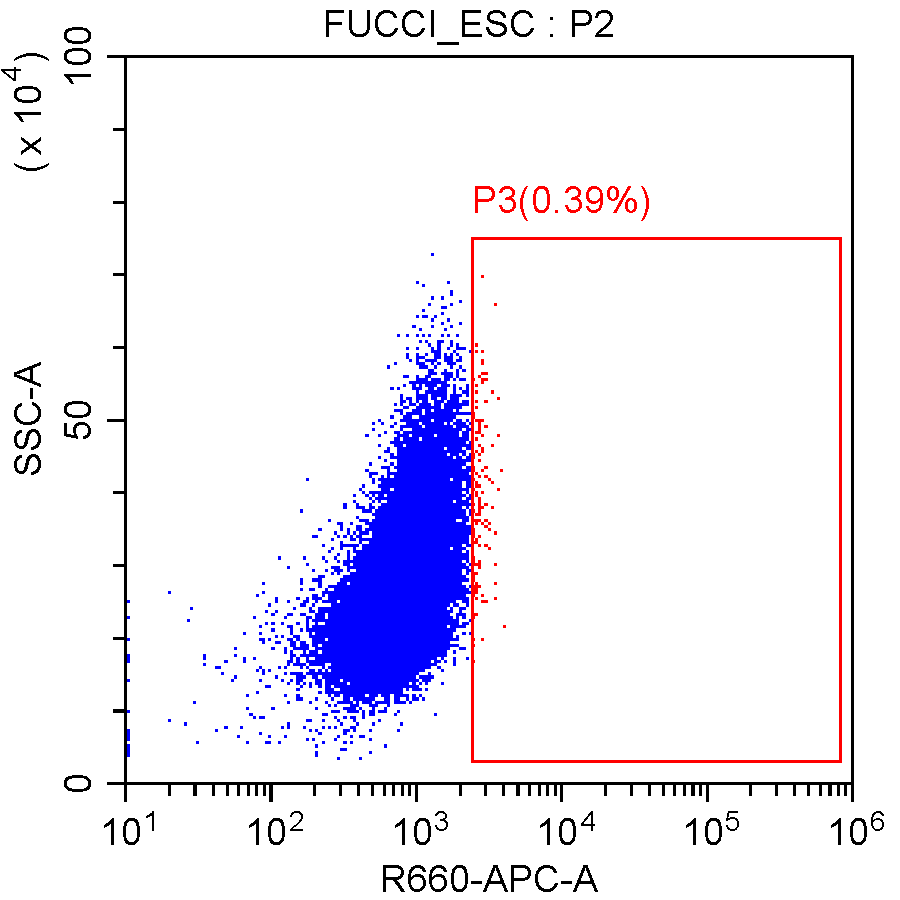

Supplement: Supplementary file 5 — Source data Fig. 3 [file 44318_2025_605_MOESM5_ESM.zip › Fig. 3/3C/Replicate_2/ESC/Ctrl.tif]

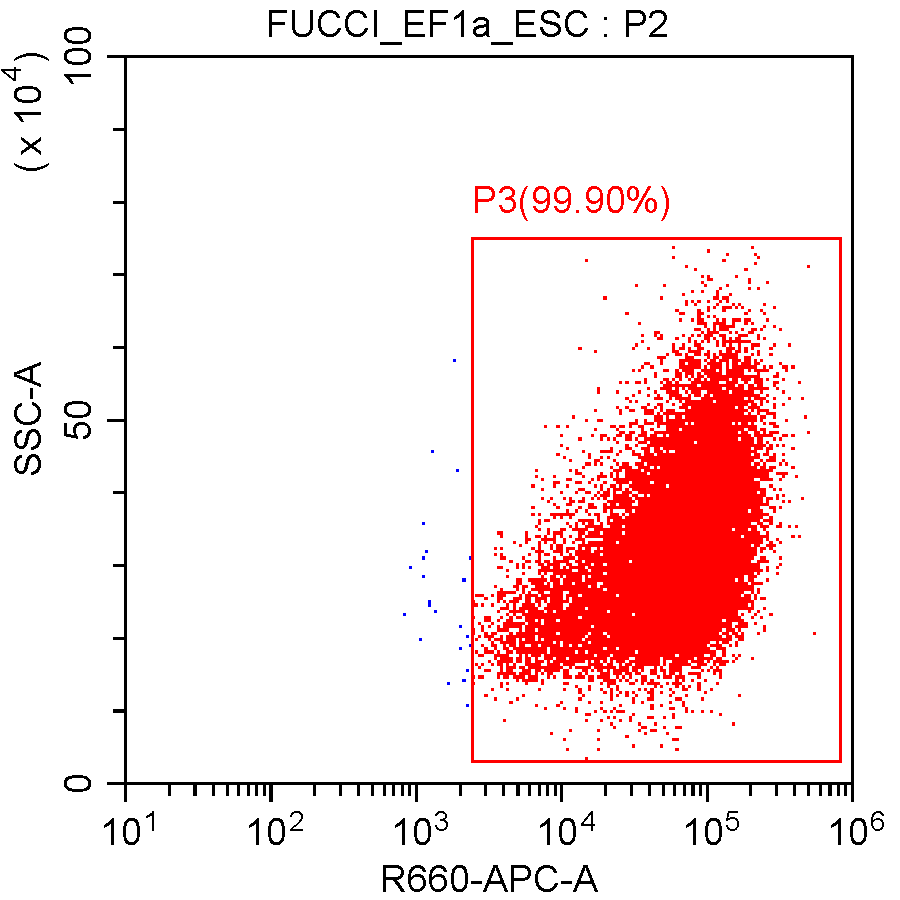

Supplement: Supplementary file 5 — Source data Fig. 3 [file 44318_2025_605_MOESM5_ESM.zip › Fig. 3/3C/Replicate_2/ESC/EF1α.tif]

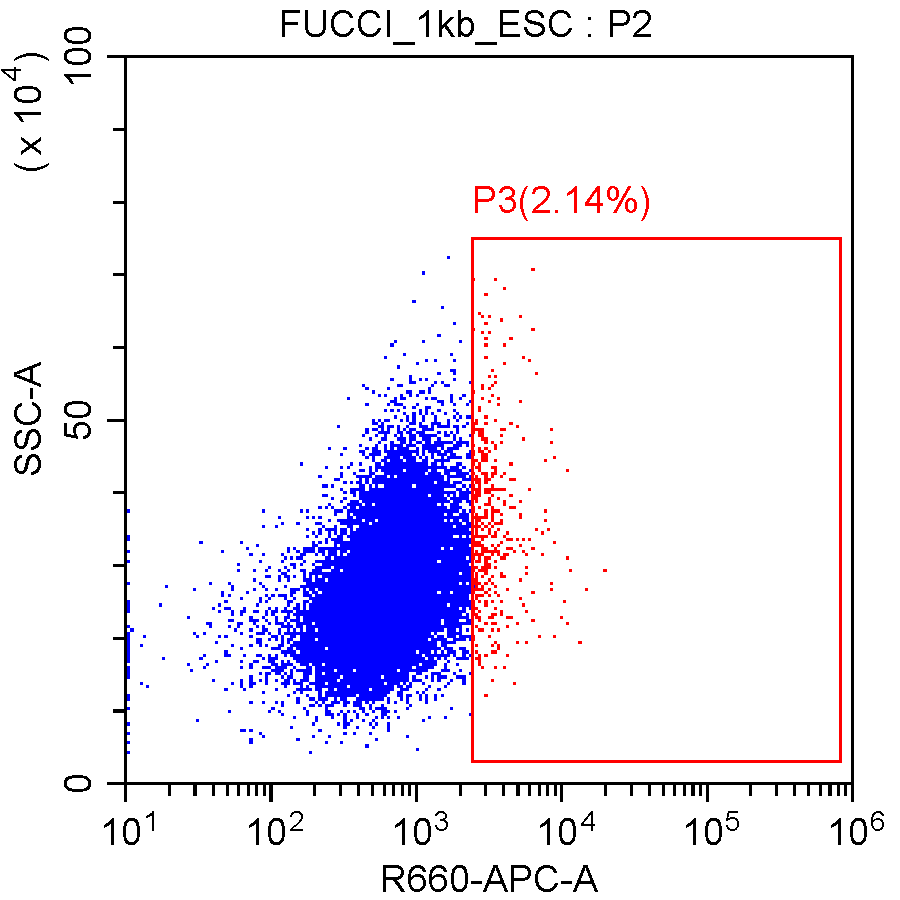

Supplement: Supplementary file 5 — Source data Fig. 3 [file 44318_2025_605_MOESM5_ESM.zip › Fig. 3/3C/Replicate_2/ESC/P1000.tif]

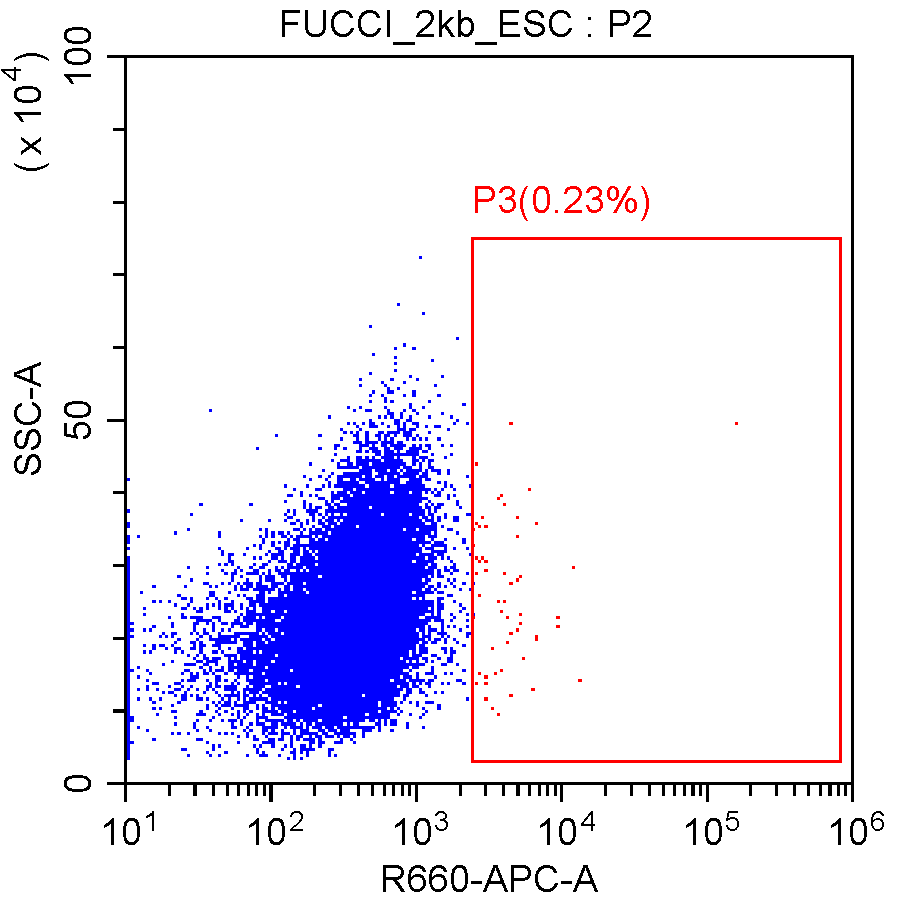

Supplement: Supplementary file 5 — Source data Fig. 3 [file 44318_2025_605_MOESM5_ESM.zip › Fig. 3/3C/Replicate_2/ESC/P2000.tif]

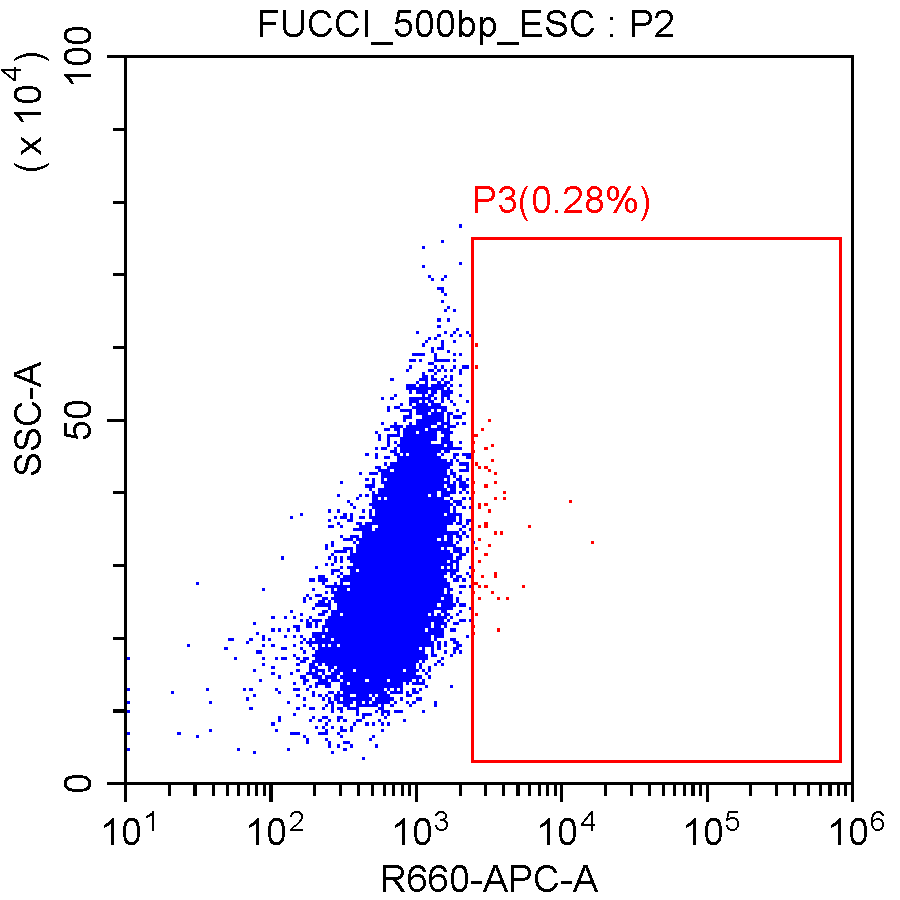

Supplement: Supplementary file 5 — Source data Fig. 3 [file 44318_2025_605_MOESM5_ESM.zip › Fig. 3/3C/Replicate_2/ESC/P500.tif]

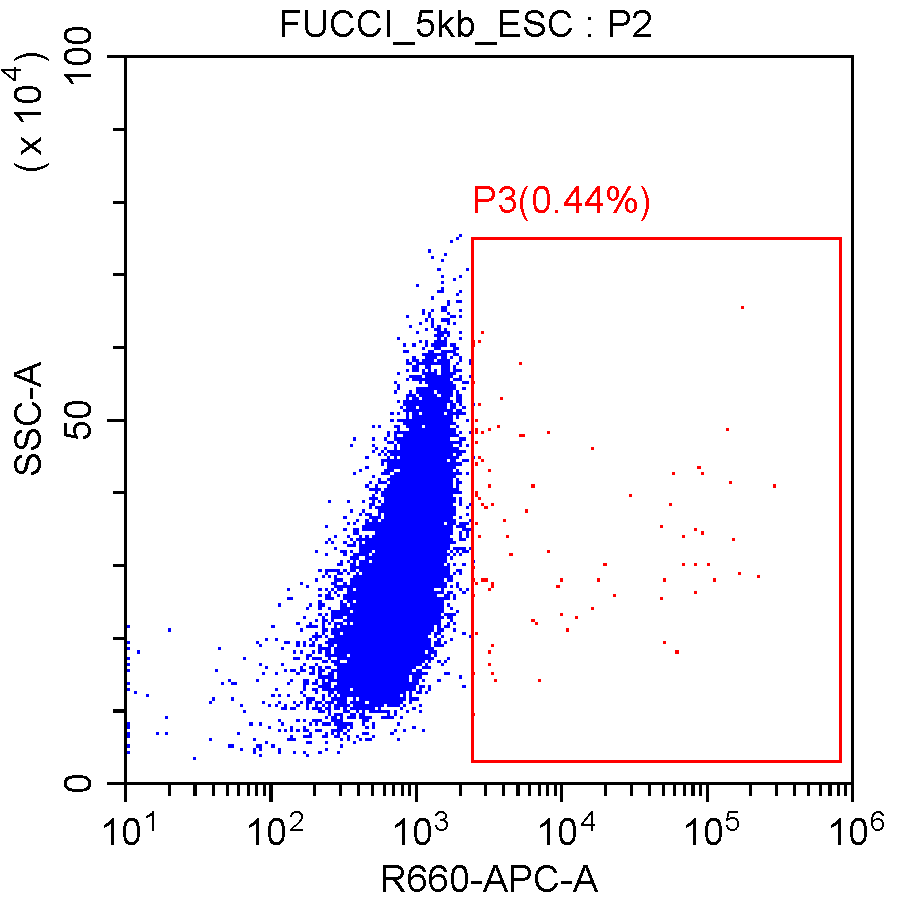

Supplement: Supplementary file 5 — Source data Fig. 3 [file 44318_2025_605_MOESM5_ESM.zip › Fig. 3/3C/Replicate_2/ESC/P5000.tif]

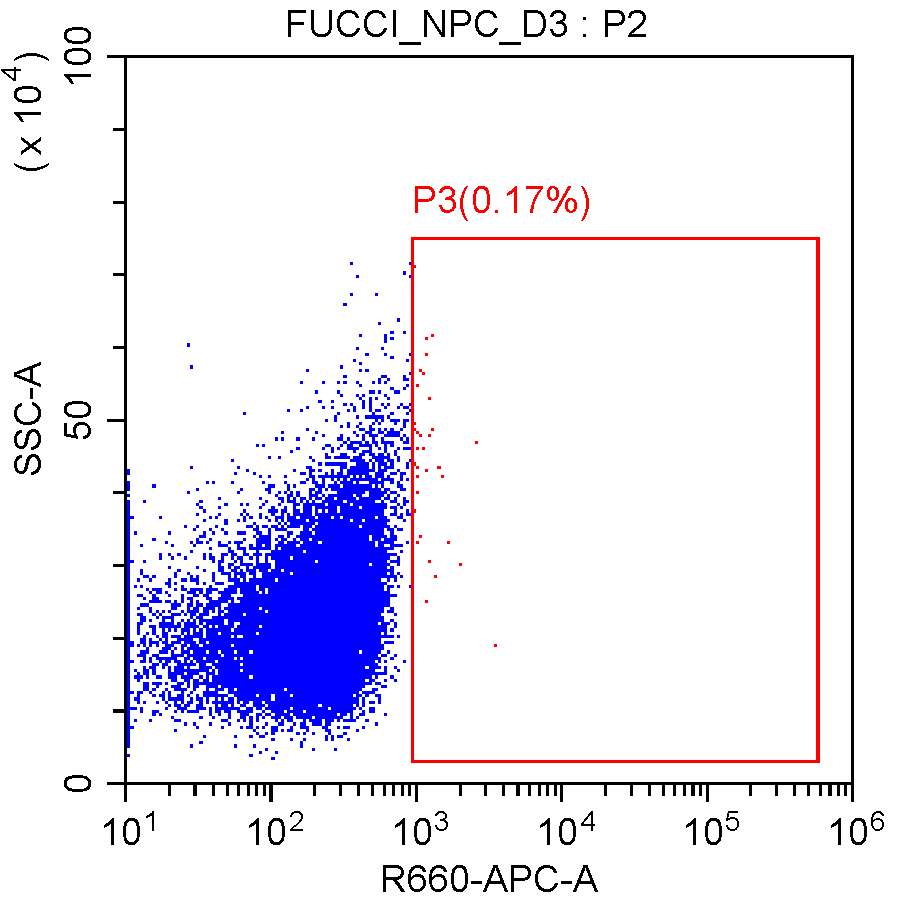

Supplement: Supplementary file 5 — Source data Fig. 3 [file 44318_2025_605_MOESM5_ESM.zip › Fig. 3/3C/Replicate_2/NPC_D3/Ctrl.tif]

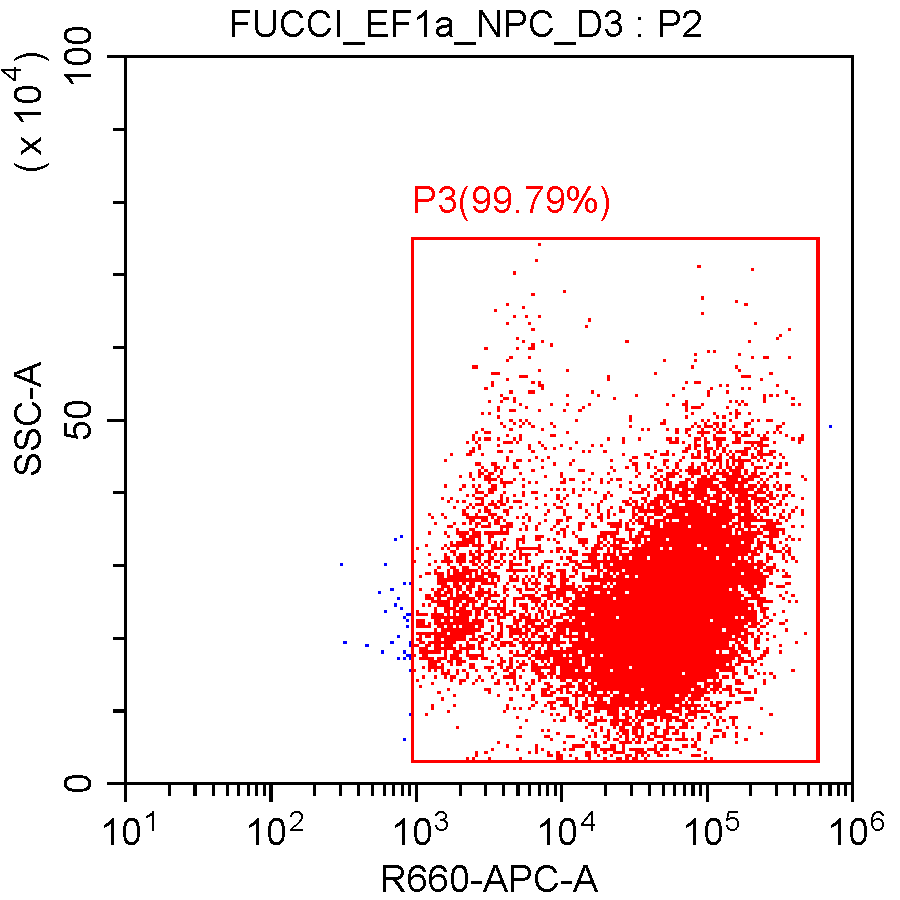

Supplement: Supplementary file 5 — Source data Fig. 3 [file 44318_2025_605_MOESM5_ESM.zip › Fig. 3/3C/Replicate_2/NPC_D3/EF1α.tif]

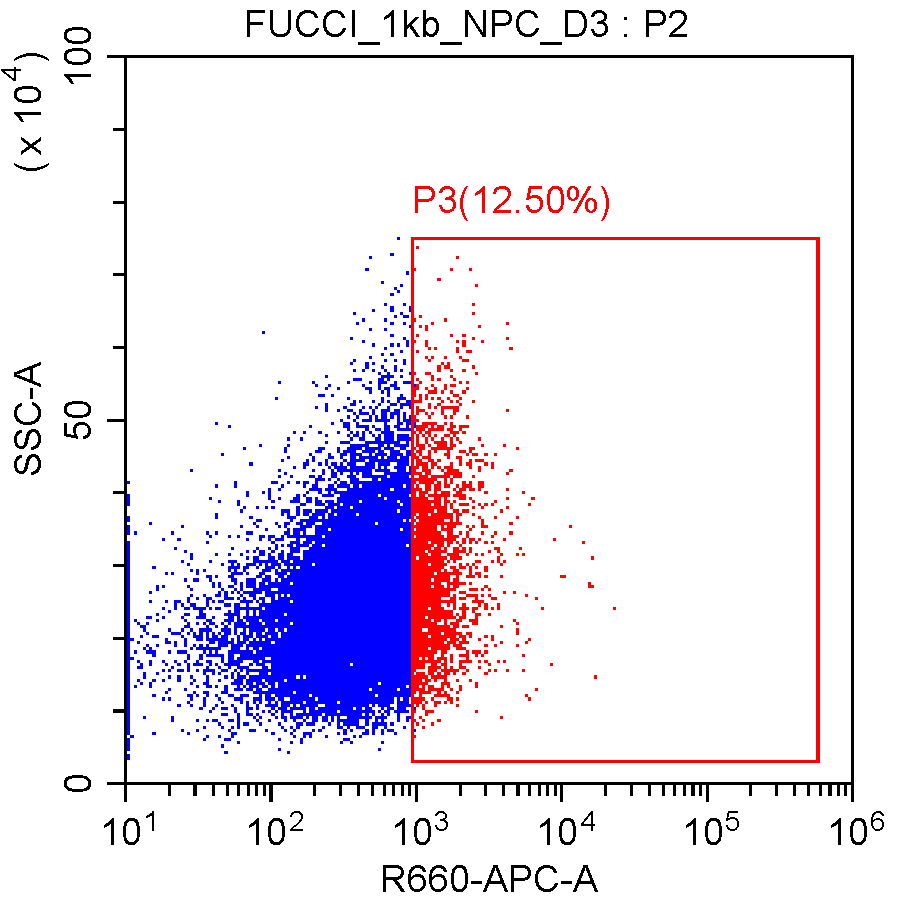

Supplement: Supplementary file 5 — Source data Fig. 3 [file 44318_2025_605_MOESM5_ESM.zip › Fig. 3/3C/Replicate_2/NPC_D3/P1000.tif]

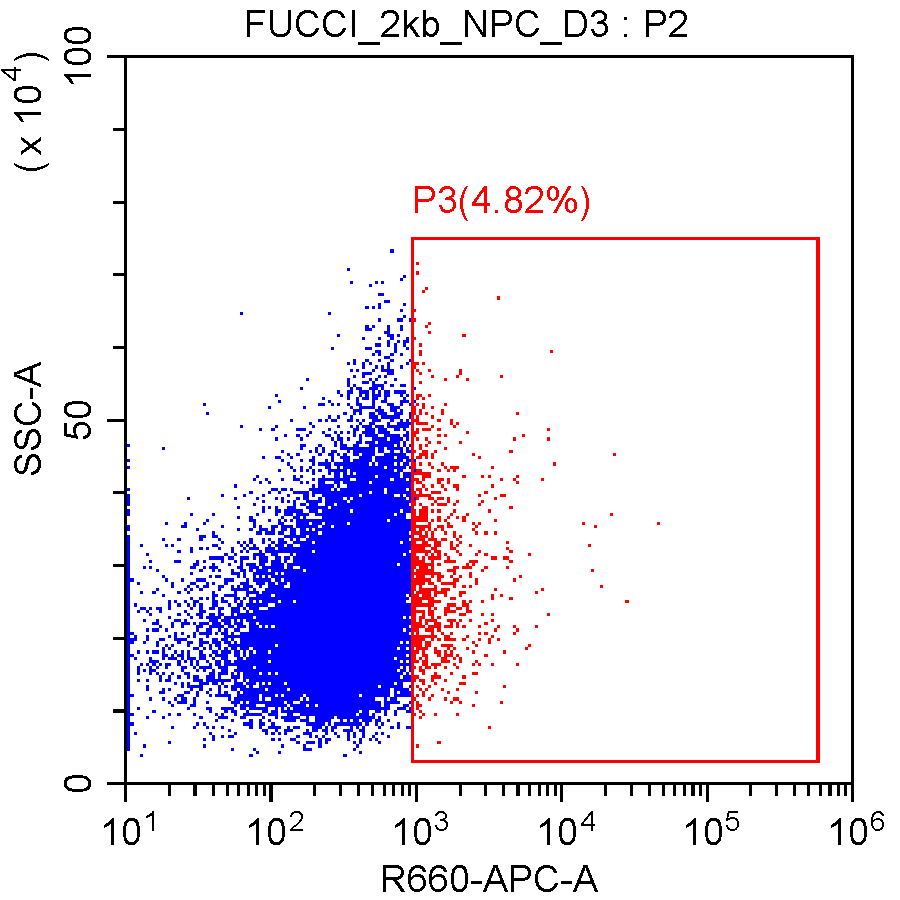

Supplement: Supplementary file 5 — Source data Fig. 3 [file 44318_2025_605_MOESM5_ESM.zip › Fig. 3/3C/Replicate_2/NPC_D3/P2000.tif]

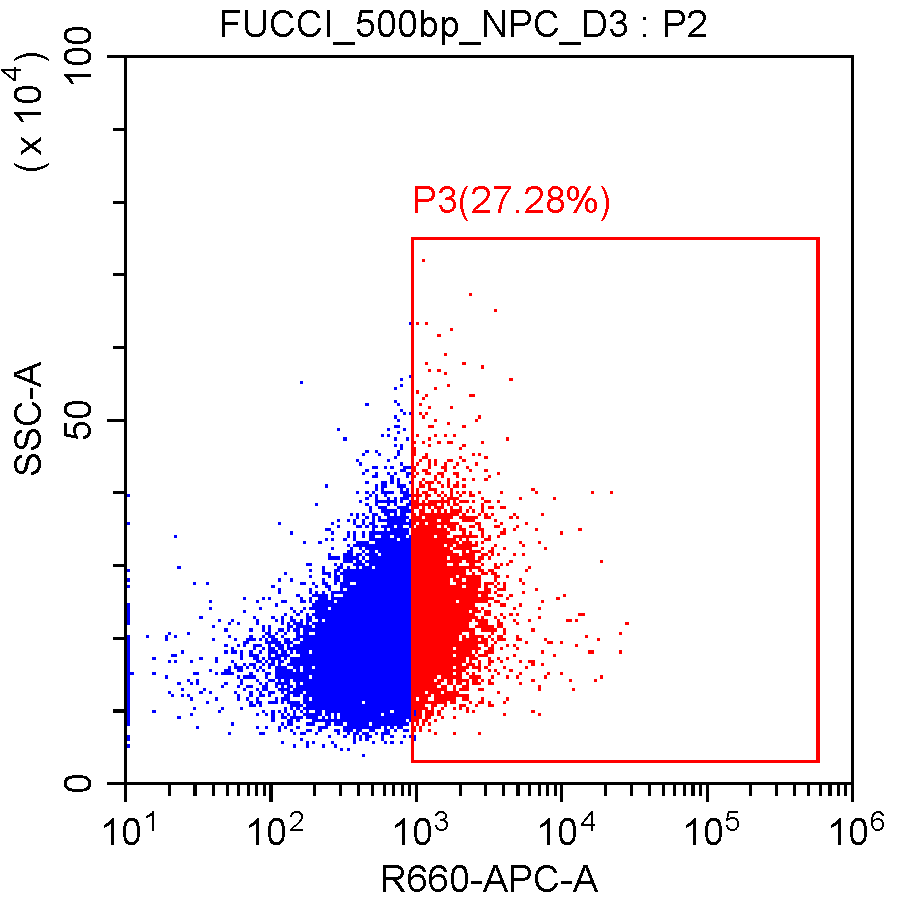

Supplement: Supplementary file 5 — Source data Fig. 3 [file 44318_2025_605_MOESM5_ESM.zip › Fig. 3/3C/Replicate_2/NPC_D3/P500.tif]

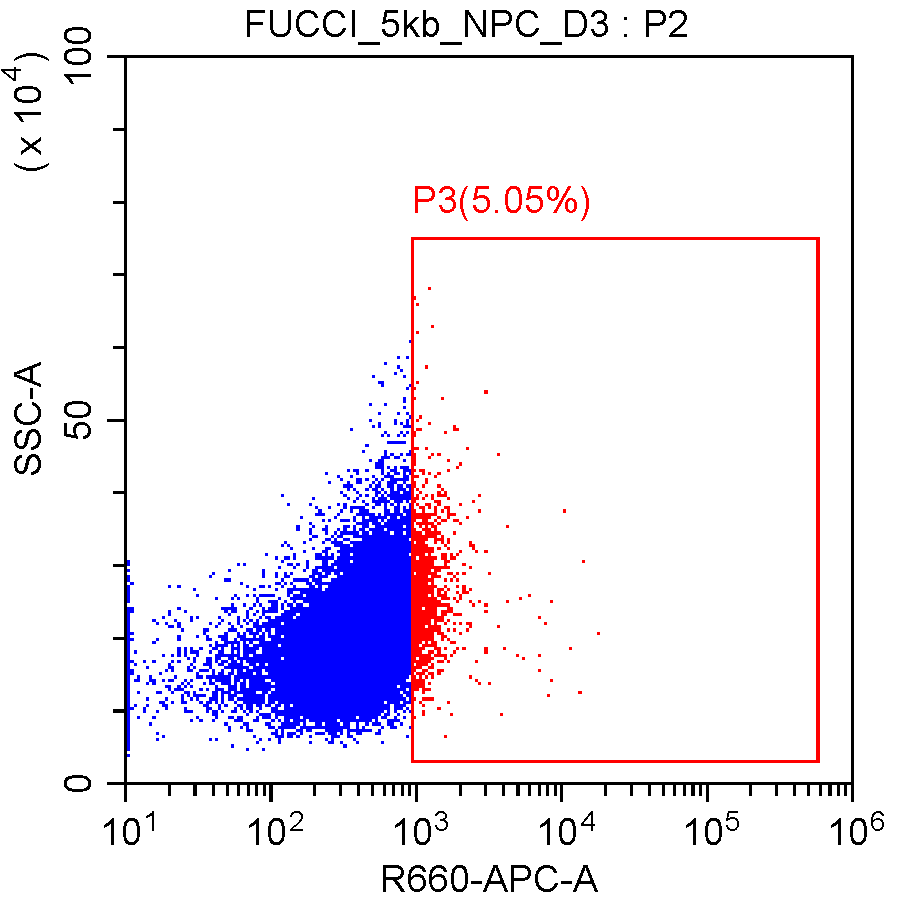

Supplement: Supplementary file 5 — Source data Fig. 3 [file 44318_2025_605_MOESM5_ESM.zip › Fig. 3/3C/Replicate_2/NPC_D3/P5000.tif]

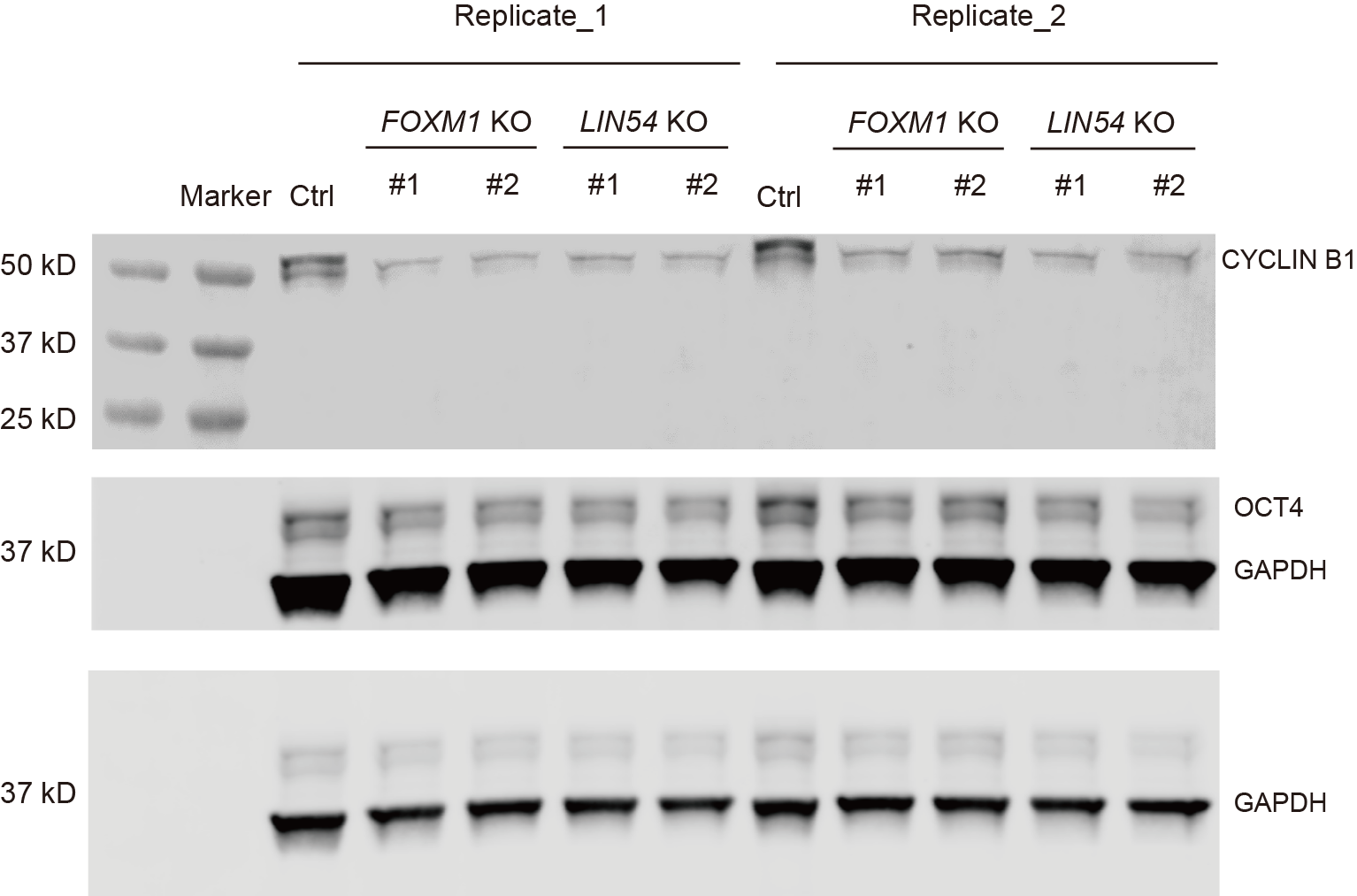

Supplement: Supplementary file 6 — Source data Fig. 4 [file 44318_2025_605_MOESM6_ESM.zip › Fig. 4/4B/4B.tif]

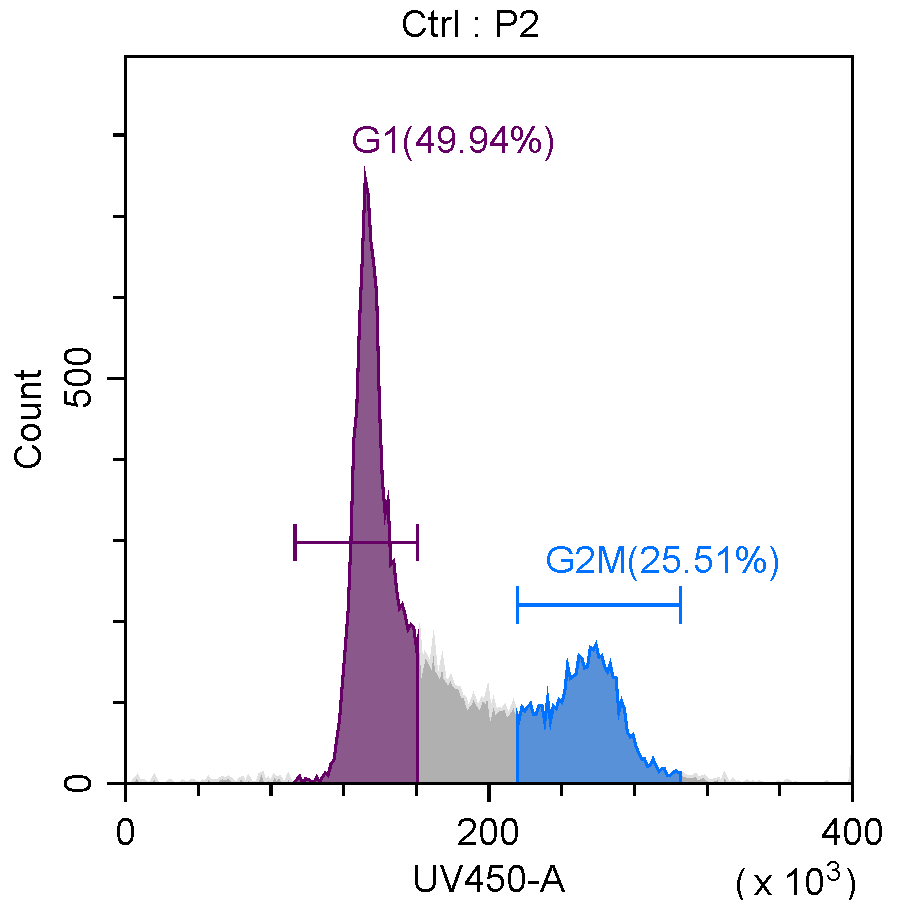

Supplement: Supplementary file 6 — Source data Fig. 4 [file 44318_2025_605_MOESM6_ESM.zip › Fig. 4/4C/Replicate 1/Ctrl.tif]

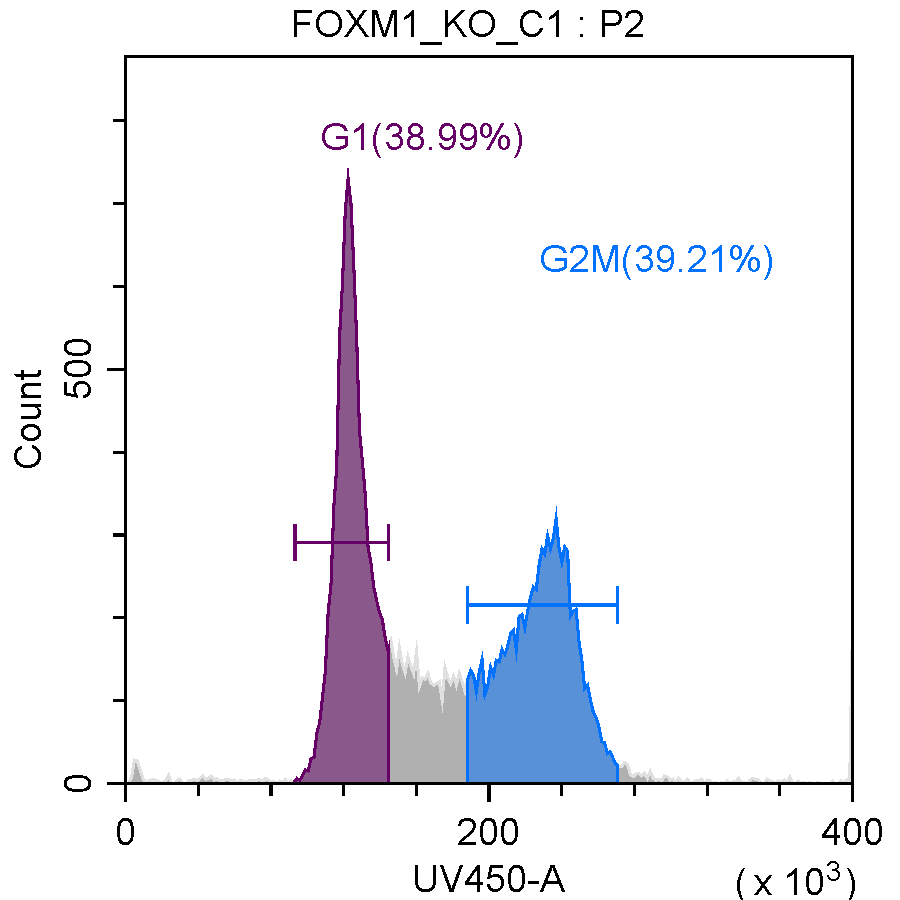

Supplement: Supplementary file 6 — Source data Fig. 4 [file 44318_2025_605_MOESM6_ESM.zip › Fig. 4/4C/Replicate 1/FOXM1_KO_#1.tif]

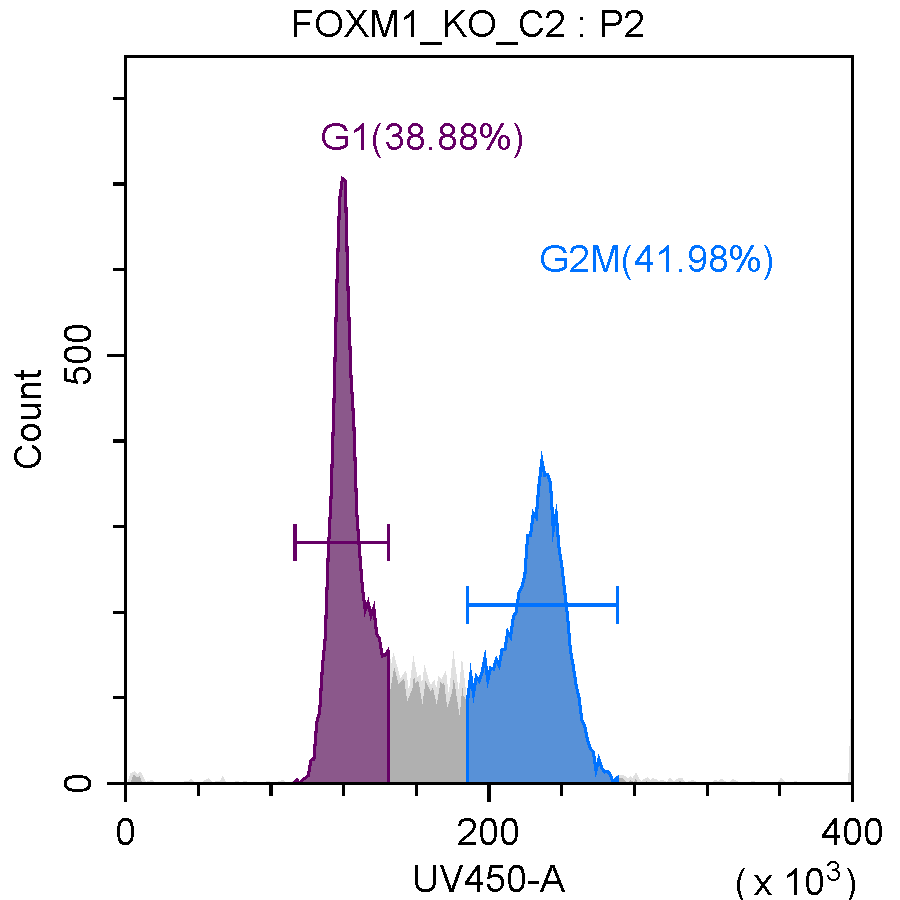

Supplement: Supplementary file 6 — Source data Fig. 4 [file 44318_2025_605_MOESM6_ESM.zip › Fig. 4/4C/Replicate 1/FOXM1_KO_#2.tif]

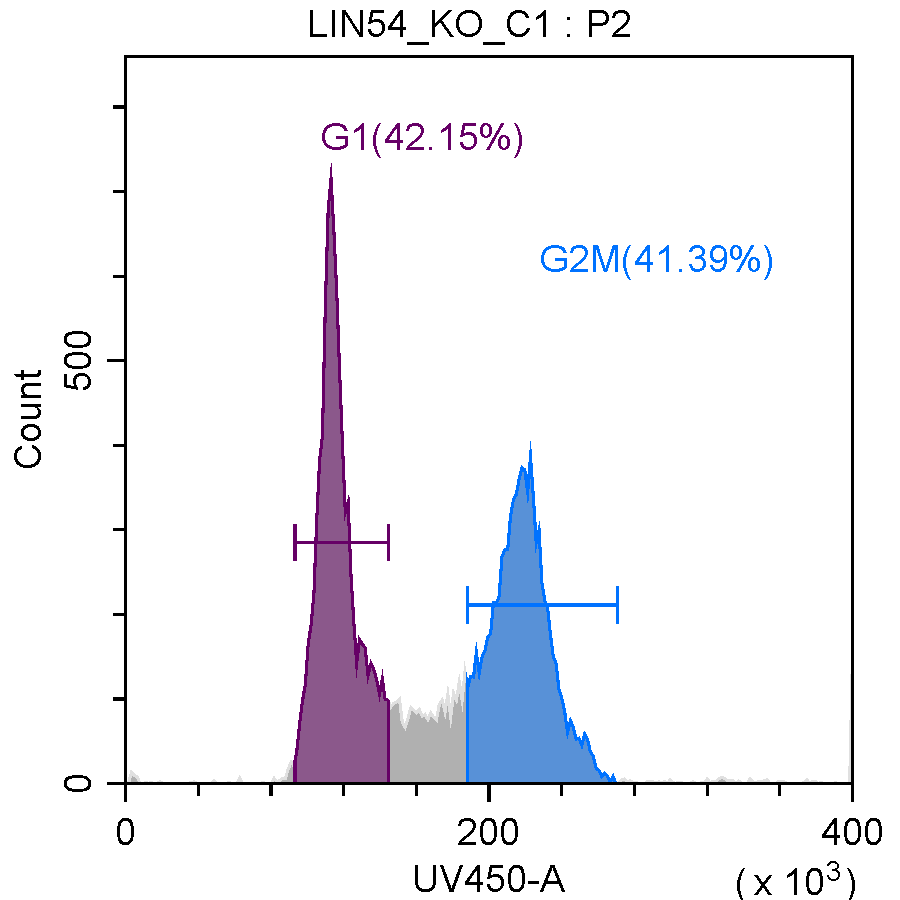

Supplement: Supplementary file 6 — Source data Fig. 4 [file 44318_2025_605_MOESM6_ESM.zip › Fig. 4/4C/Replicate 1/LIN54_KO_#1.tif]

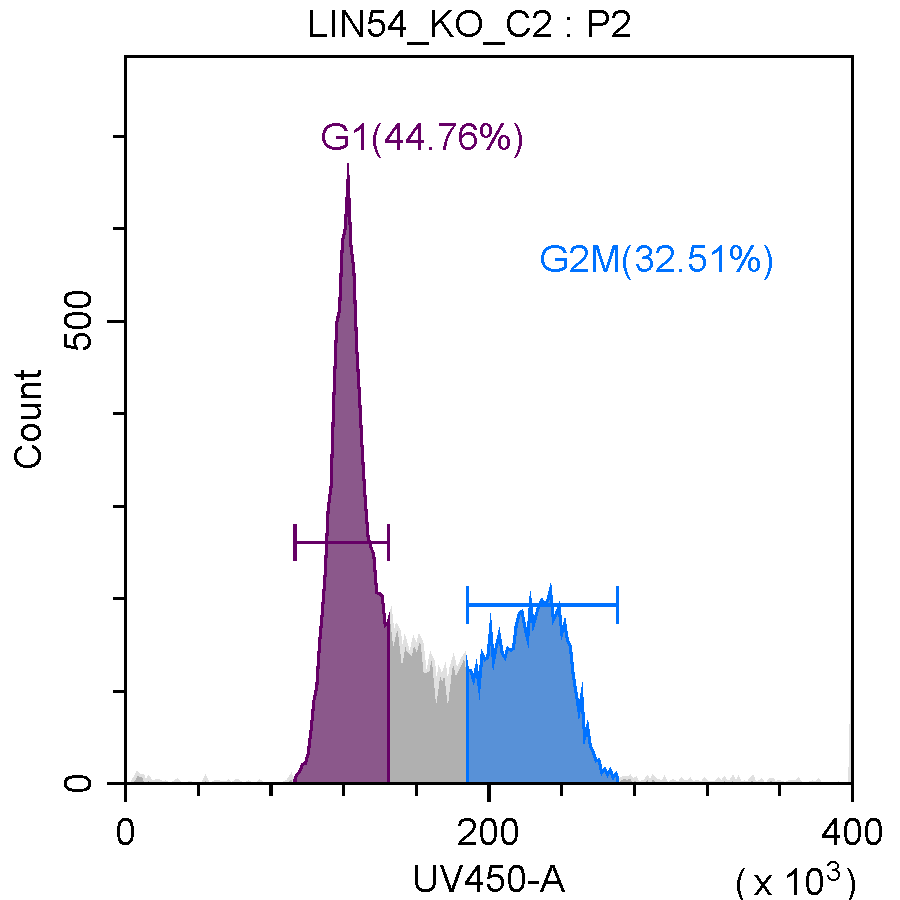

Supplement: Supplementary file 6 — Source data Fig. 4 [file 44318_2025_605_MOESM6_ESM.zip › Fig. 4/4C/Replicate 1/LIN54_KO_#2.tif]

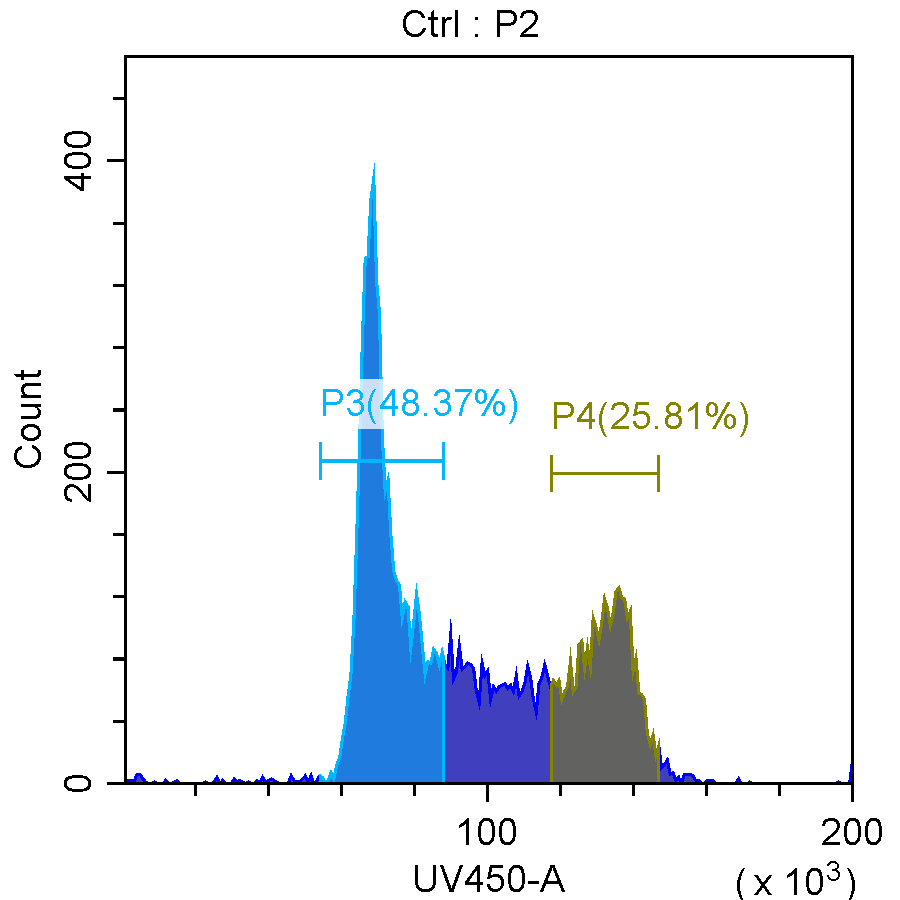

Supplement: Supplementary file 6 — Source data Fig. 4 [file 44318_2025_605_MOESM6_ESM.zip › Fig. 4/4C/Replicate 2/Ctrl.tif]

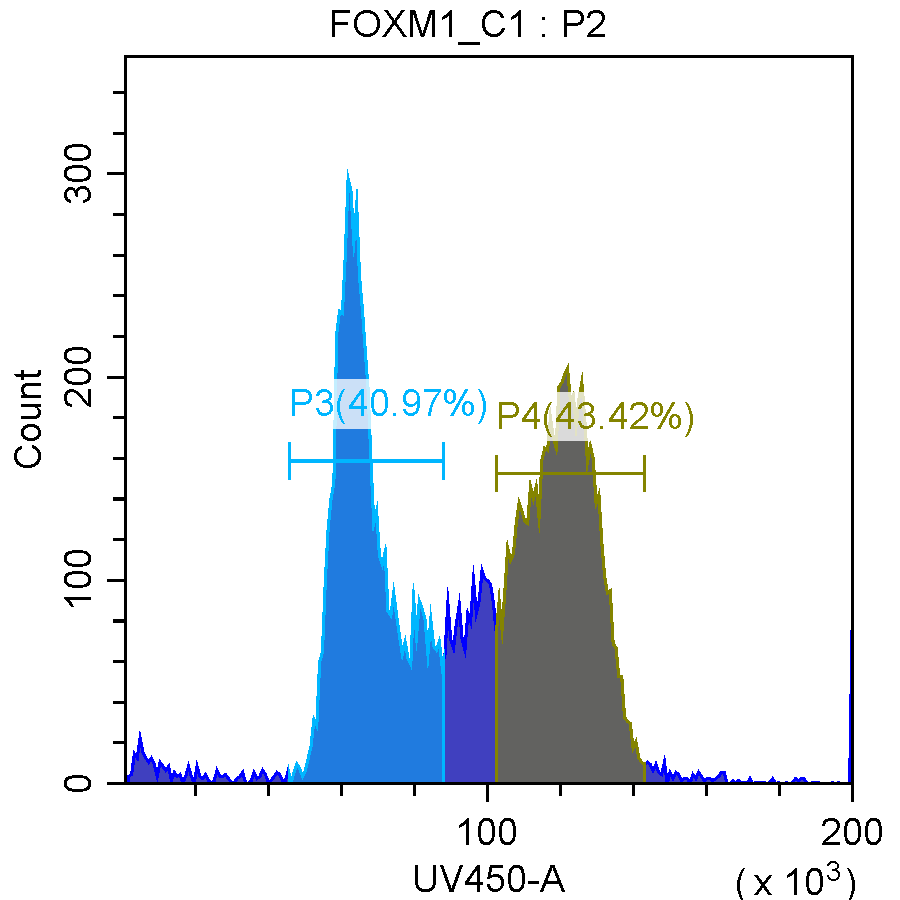

Supplement: Supplementary file 6 — Source data Fig. 4 [file 44318_2025_605_MOESM6_ESM.zip › Fig. 4/4C/Replicate 2/FOXM1_KO_#1.tif]

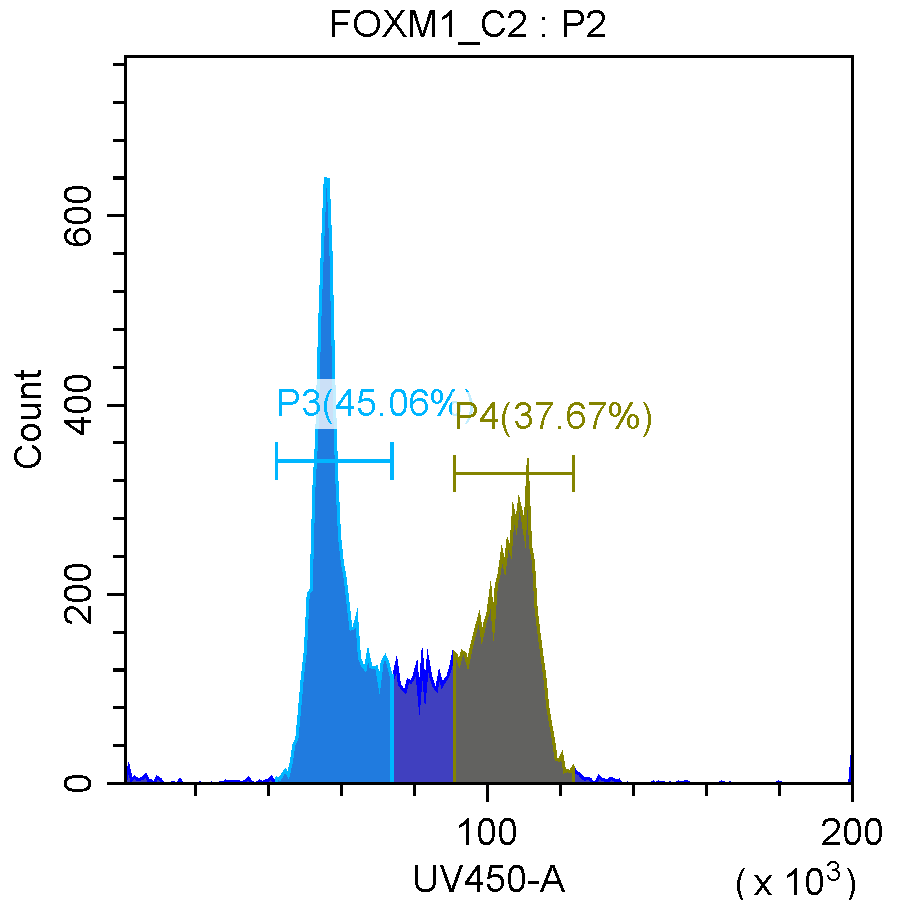

Supplement: Supplementary file 6 — Source data Fig. 4 [file 44318_2025_605_MOESM6_ESM.zip › Fig. 4/4C/Replicate 2/FOXM1_KO_#2.tif]

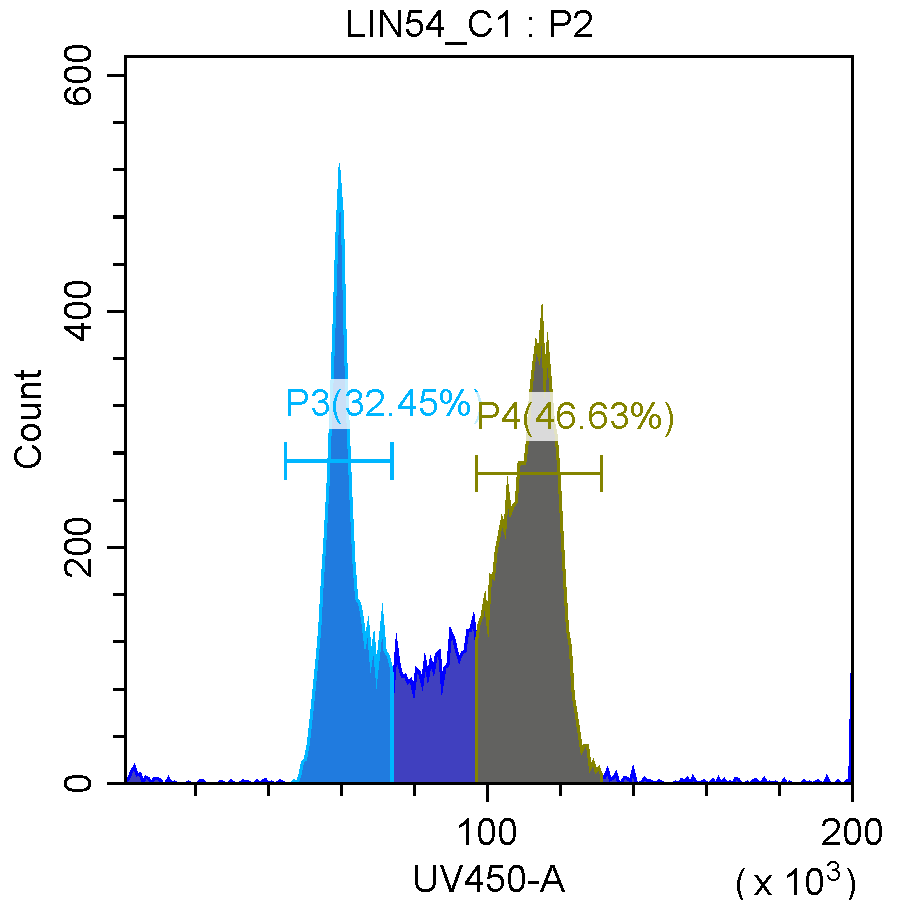

Supplement: Supplementary file 6 — Source data Fig. 4 [file 44318_2025_605_MOESM6_ESM.zip › Fig. 4/4C/Replicate 2/LIN54_KO_#1.tif]

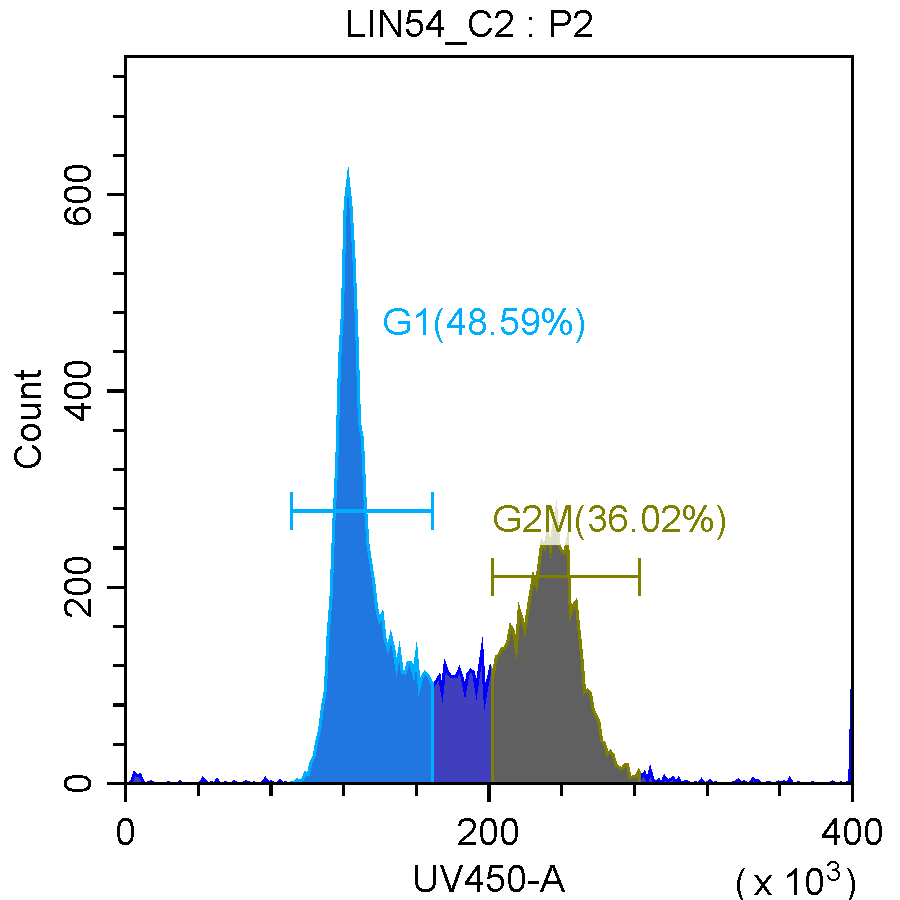

Supplement: Supplementary file 6 — Source data Fig. 4 [file 44318_2025_605_MOESM6_ESM.zip › Fig. 4/4C/Replicate 2/LIN54_KO_#2.tif]

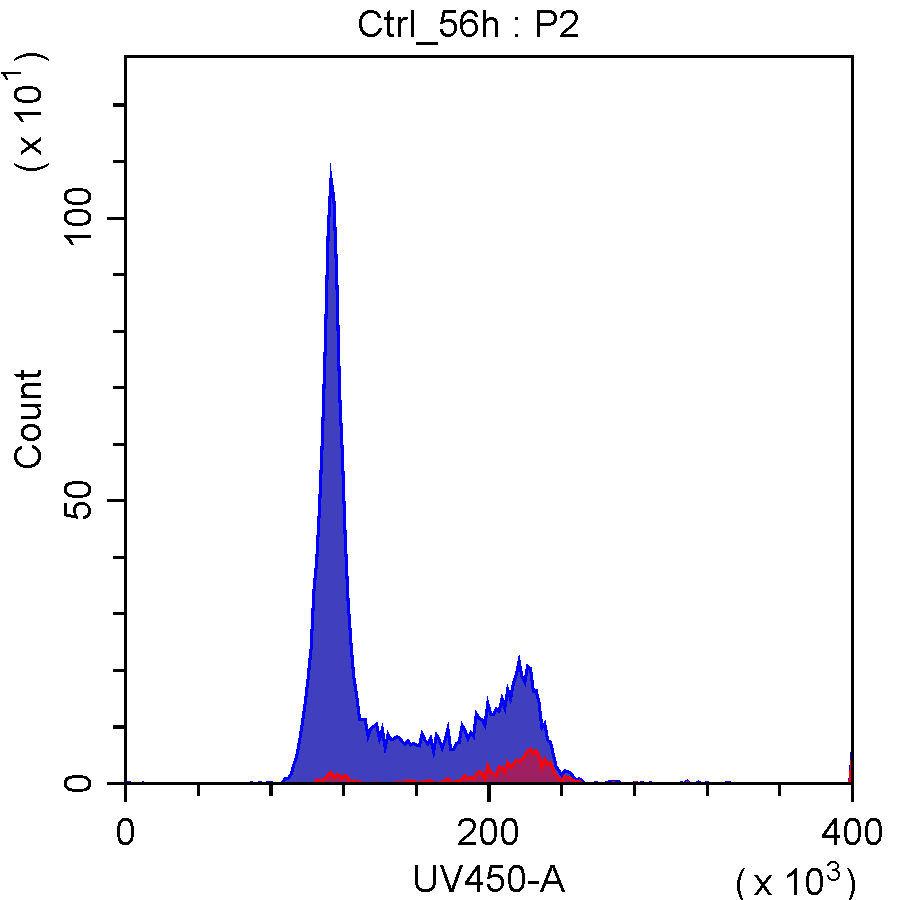

Supplement: Supplementary file 6 — Source data Fig. 4 [file 44318_2025_605_MOESM6_ESM.zip › Fig. 4/4E/Replicate_1/DAPI/Ctrl.tif]

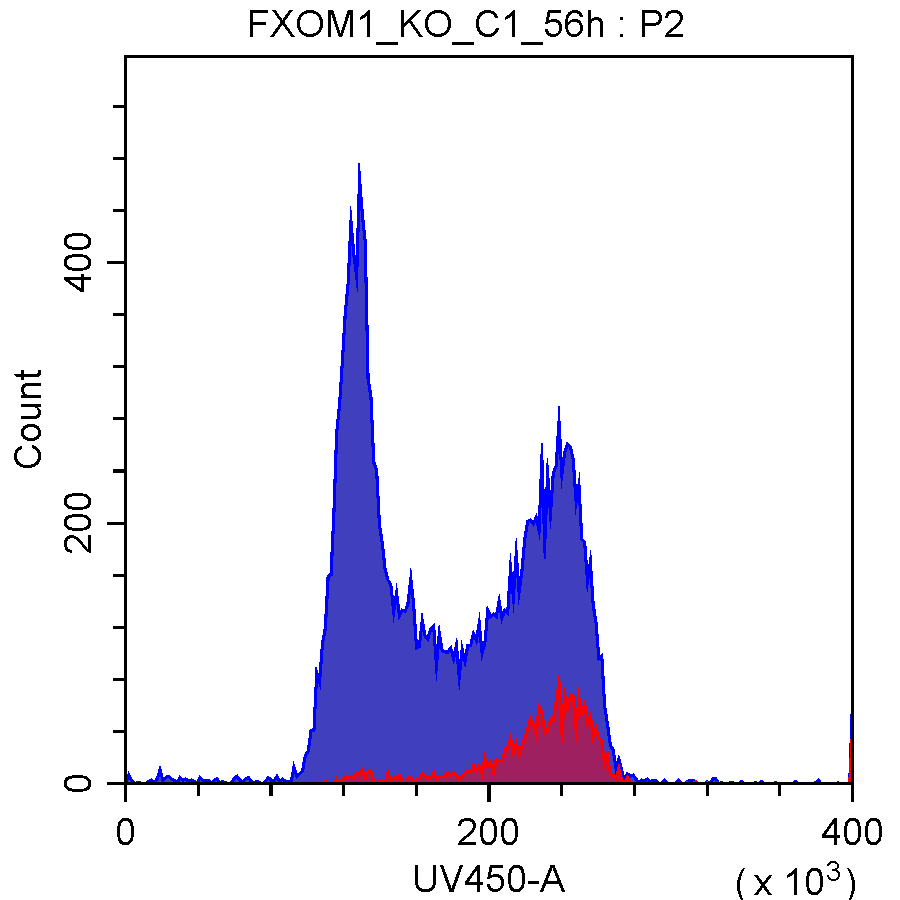

Supplement: Supplementary file 6 — Source data Fig. 4 [file 44318_2025_605_MOESM6_ESM.zip › Fig. 4/4E/Replicate_1/DAPI/FOXM1_KO_#1.tif]

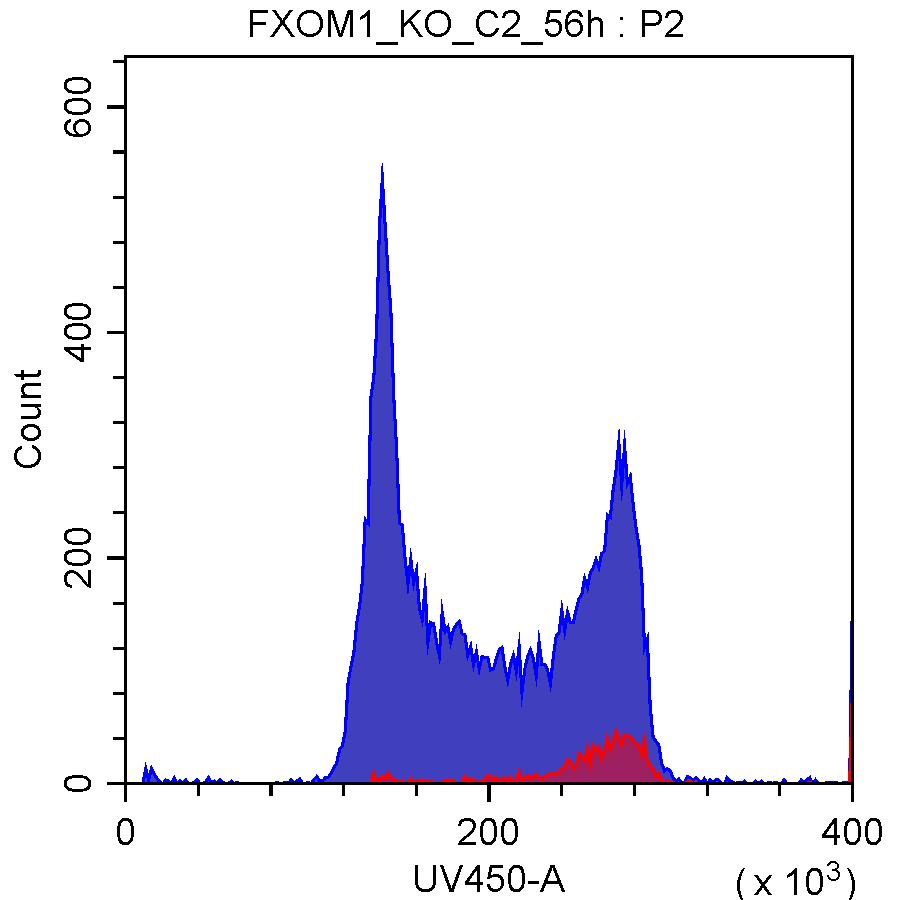

Supplement: Supplementary file 6 — Source data Fig. 4 [file 44318_2025_605_MOESM6_ESM.zip › Fig. 4/4E/Replicate_1/DAPI/FOXM1_KO_#2.tif]

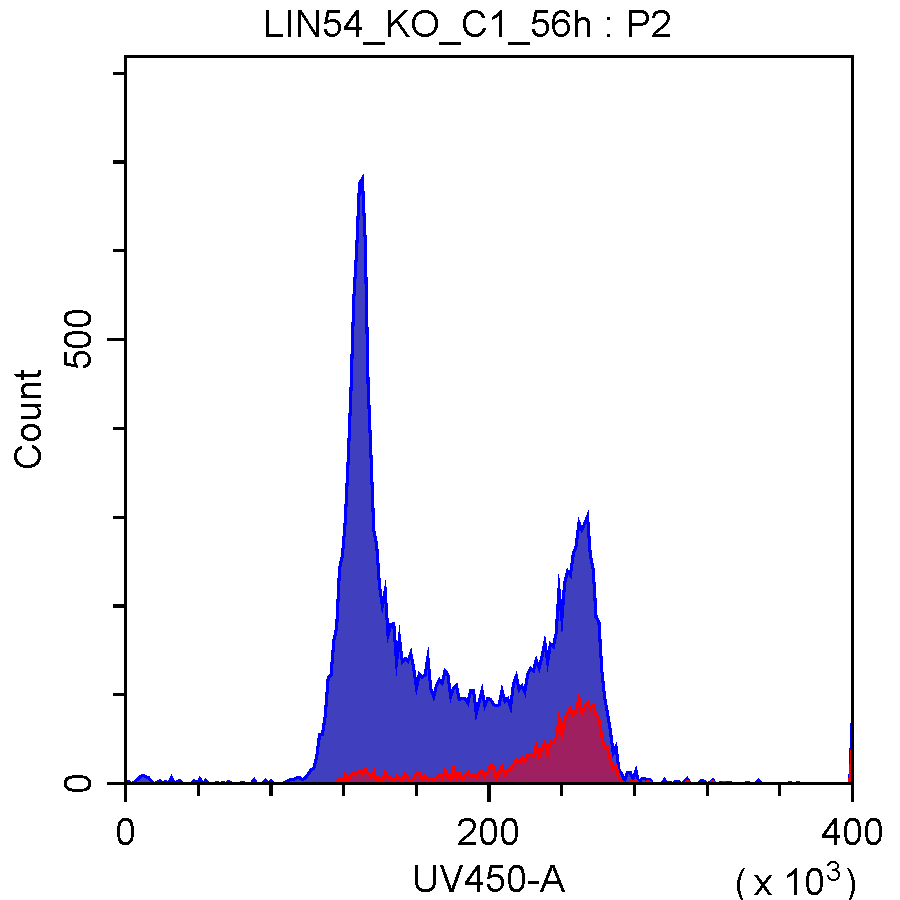

Supplement: Supplementary file 6 — Source data Fig. 4 [file 44318_2025_605_MOESM6_ESM.zip › Fig. 4/4E/Replicate_1/DAPI/LIN54_KO_#1.tif]

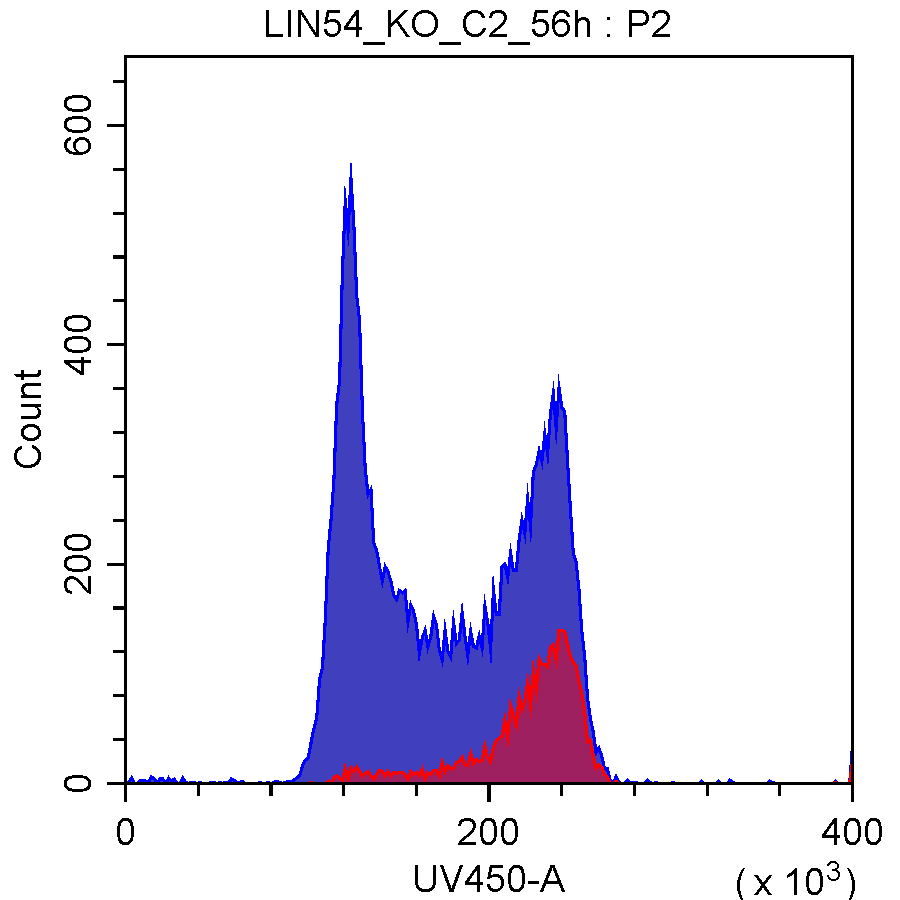

Supplement: Supplementary file 6 — Source data Fig. 4 [file 44318_2025_605_MOESM6_ESM.zip › Fig. 4/4E/Replicate_1/DAPI/LIN54_KO_#2.tif]

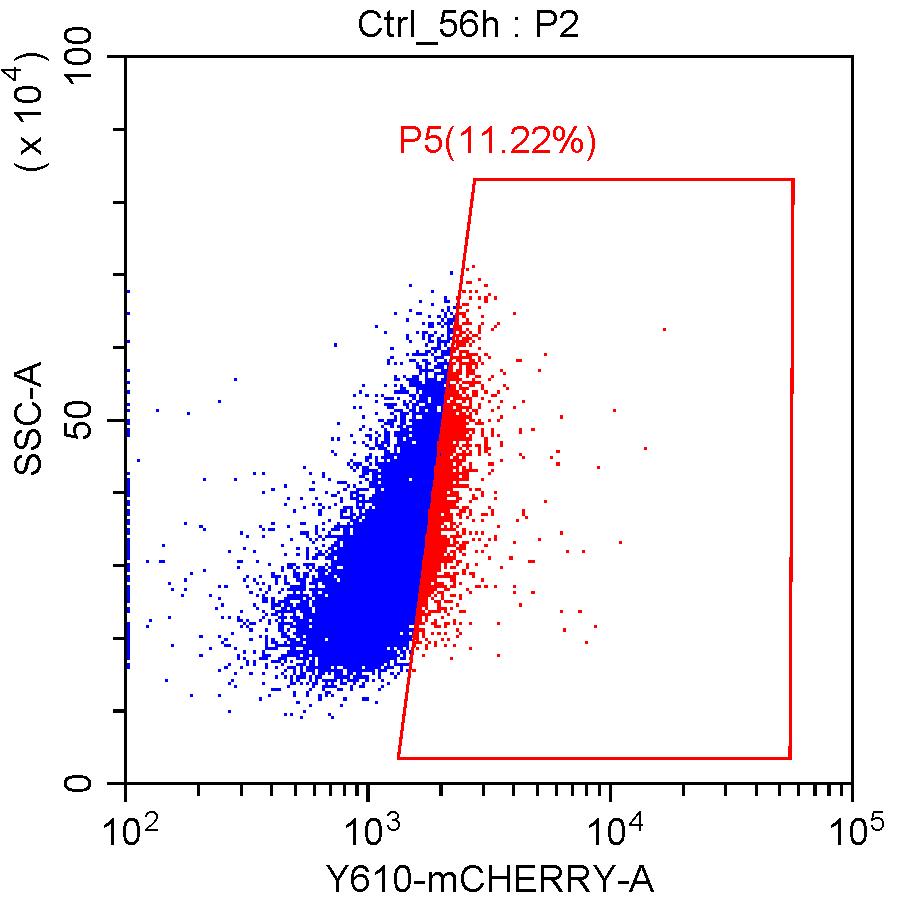

Supplement: Supplementary file 6 — Source data Fig. 4 [file 44318_2025_605_MOESM6_ESM.zip › Fig. 4/4E/Replicate_1/PAX6/Ctrl.tif]

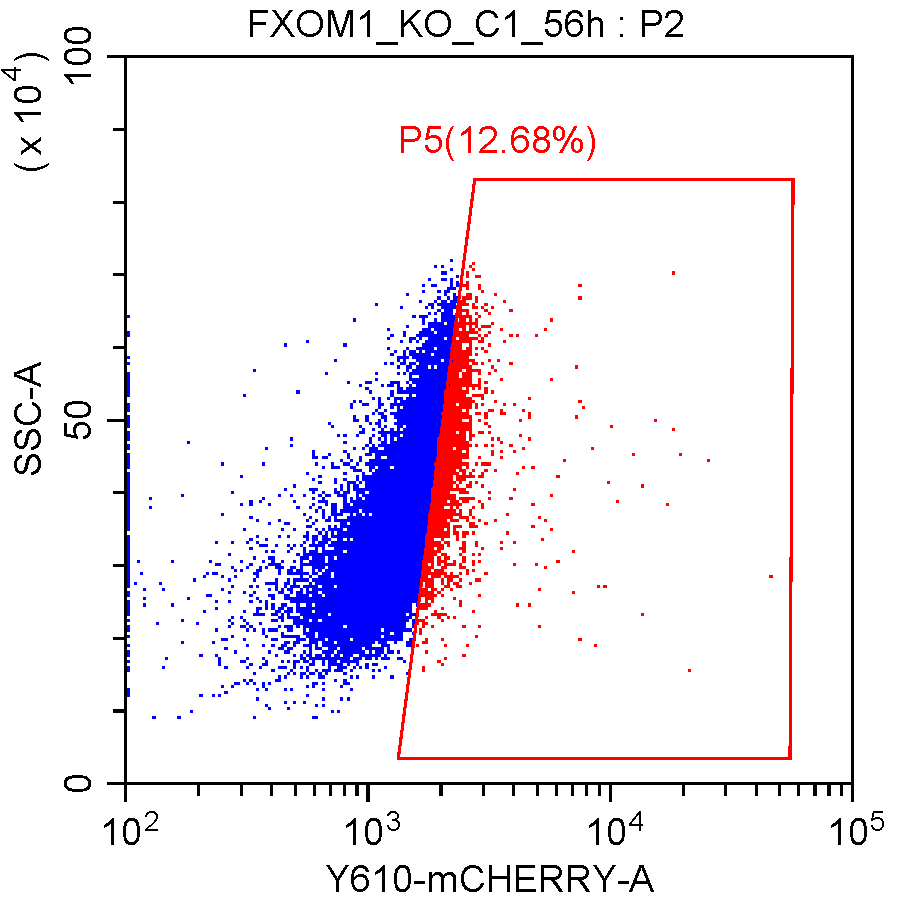

Supplement: Supplementary file 6 — Source data Fig. 4 [file 44318_2025_605_MOESM6_ESM.zip › Fig. 4/4E/Replicate_1/PAX6/FOXM1_KO_#1.tif]

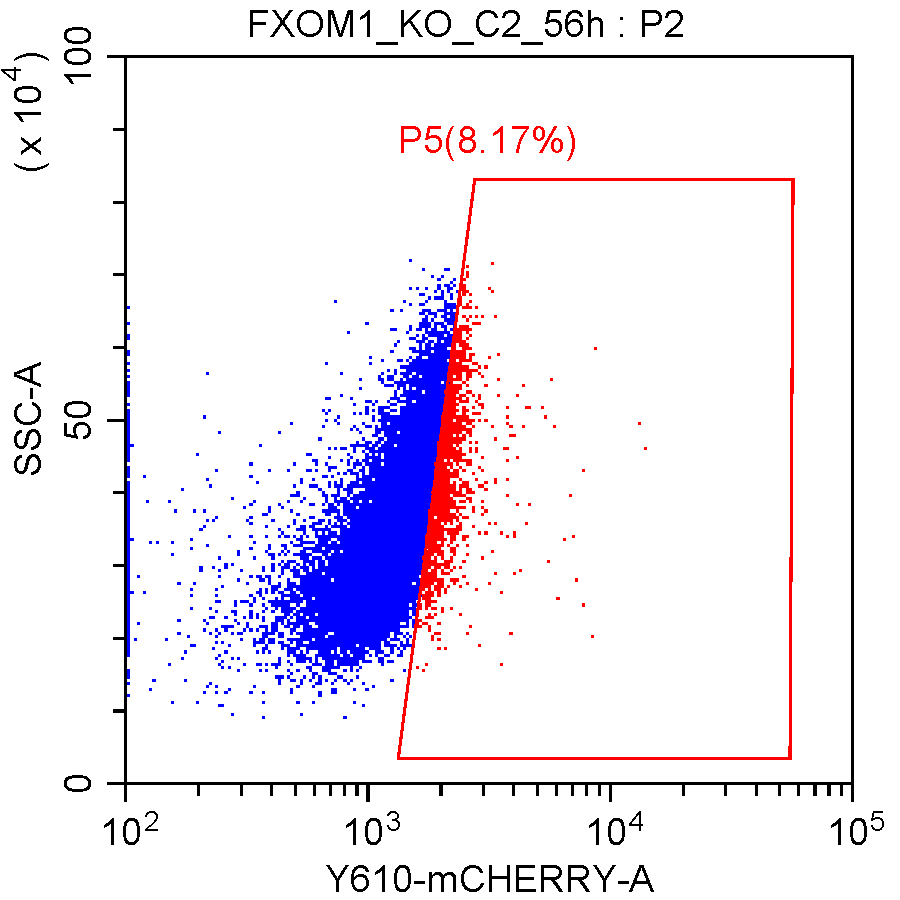

Supplement: Supplementary file 6 — Source data Fig. 4 [file 44318_2025_605_MOESM6_ESM.zip › Fig. 4/4E/Replicate_1/PAX6/FOXM1_KO_#2.tif]

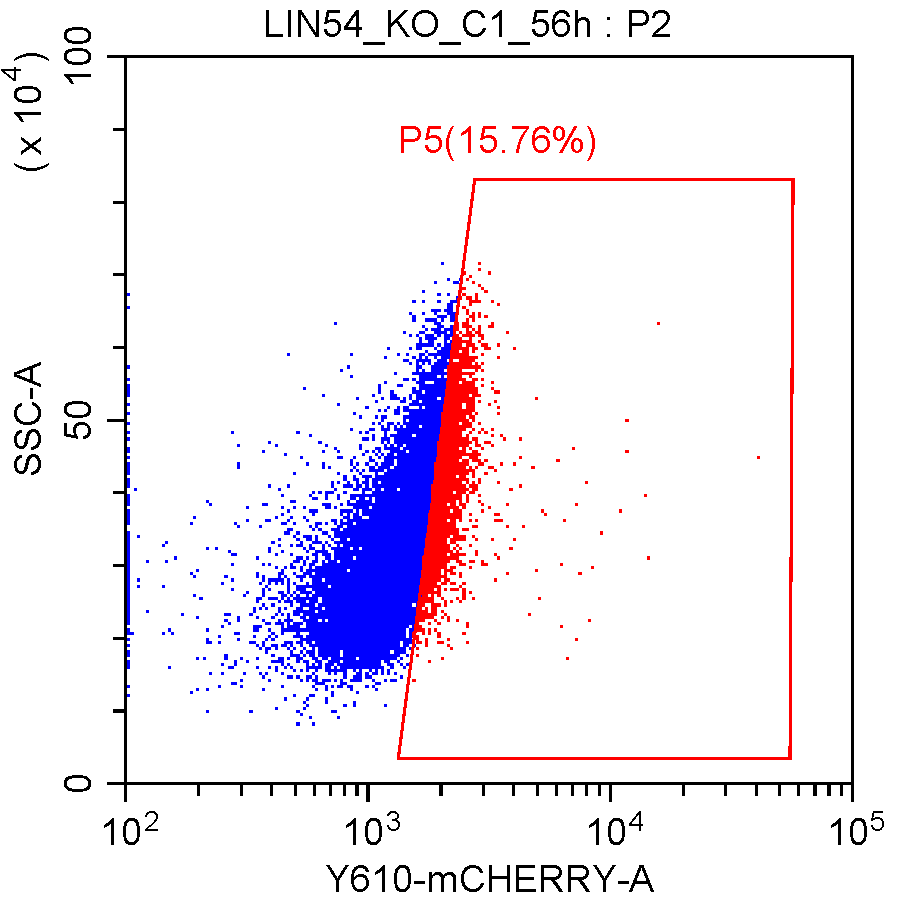

Supplement: Supplementary file 6 — Source data Fig. 4 [file 44318_2025_605_MOESM6_ESM.zip › Fig. 4/4E/Replicate_1/PAX6/LIN54_KO_#1.tif]

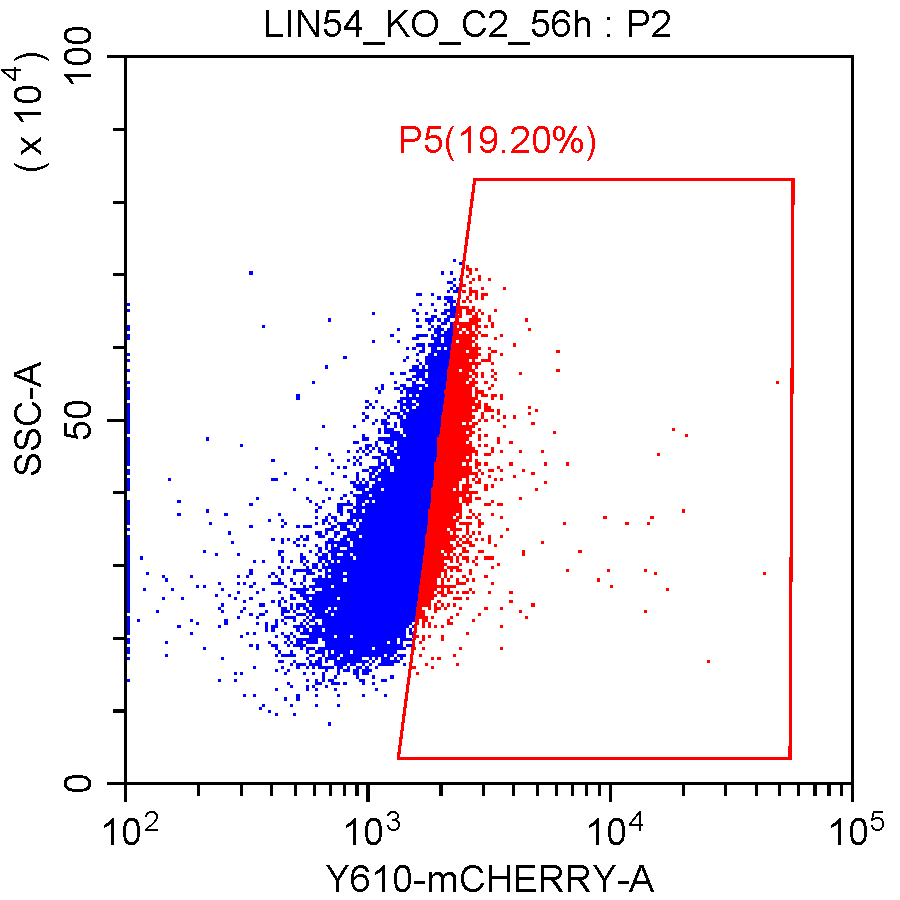

Supplement: Supplementary file 6 — Source data Fig. 4 [file 44318_2025_605_MOESM6_ESM.zip › Fig. 4/4E/Replicate_1/PAX6/LIN54_KO_#2.tif]

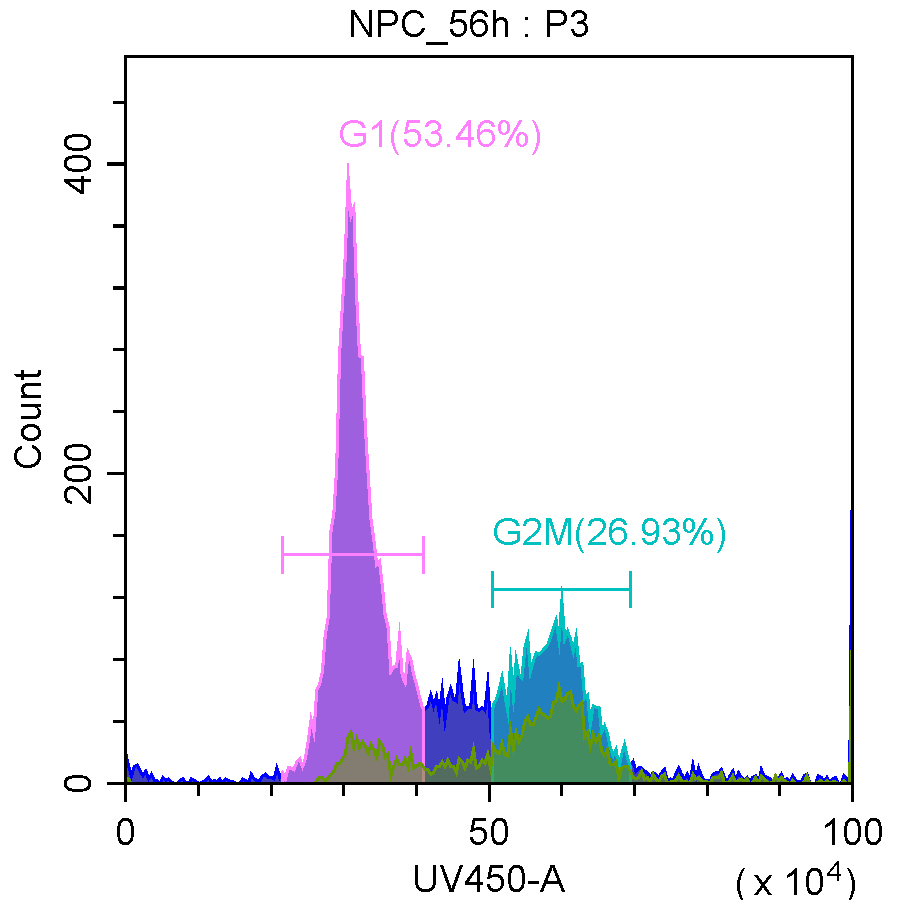

Supplement: Supplementary file 6 — Source data Fig. 4 [file 44318_2025_605_MOESM6_ESM.zip › Fig. 4/4E/Replicate_2/DAPI/Ctrl.tif]

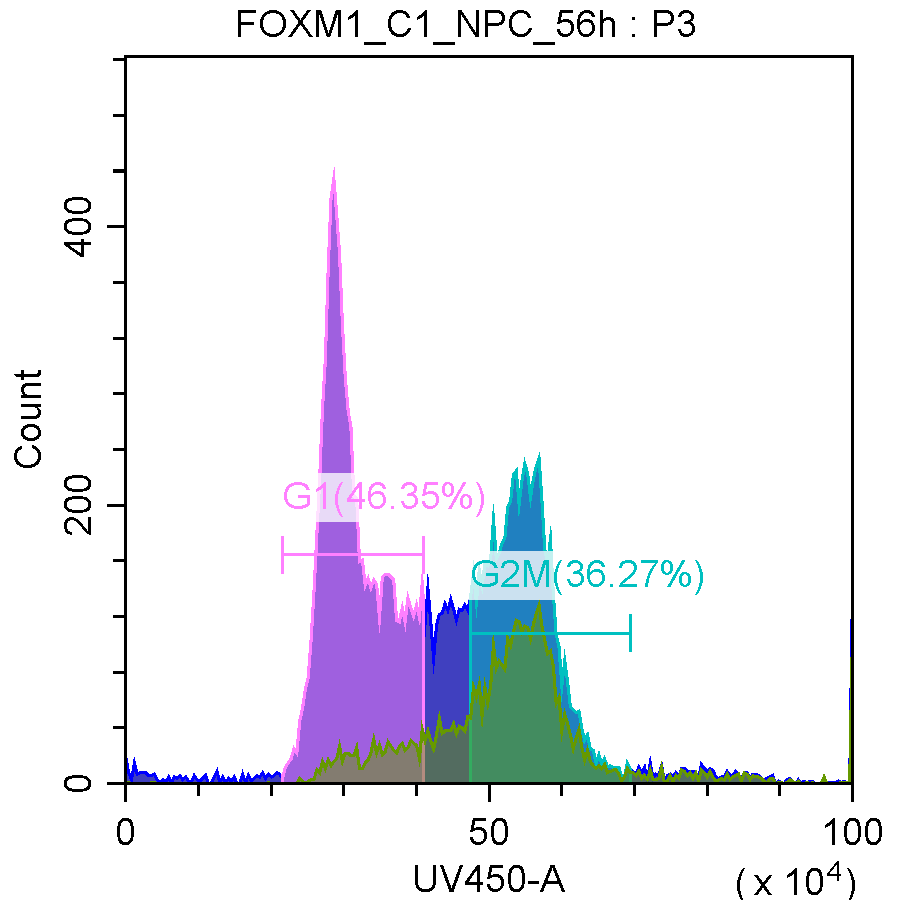

Supplement: Supplementary file 6 — Source data Fig. 4 [file 44318_2025_605_MOESM6_ESM.zip › Fig. 4/4E/Replicate_2/DAPI/FOXM1_KO_#1.tif]

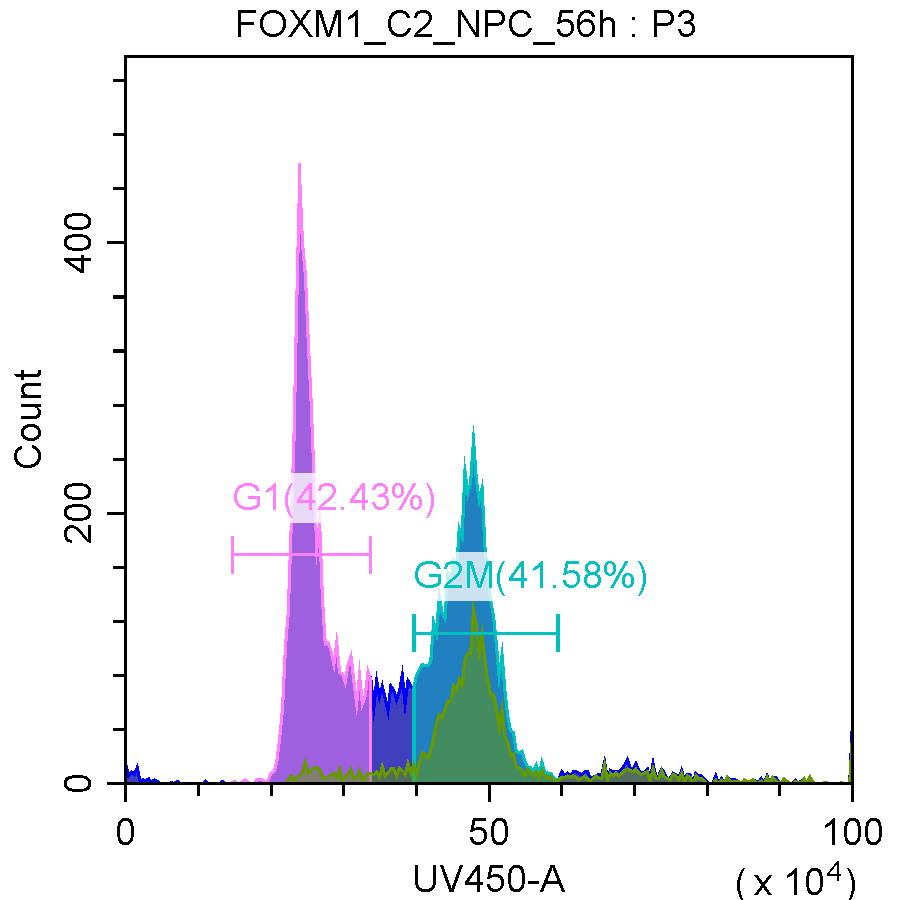

Supplement: Supplementary file 6 — Source data Fig. 4 [file 44318_2025_605_MOESM6_ESM.zip › Fig. 4/4E/Replicate_2/DAPI/FOXM1_KO_#2.tif]

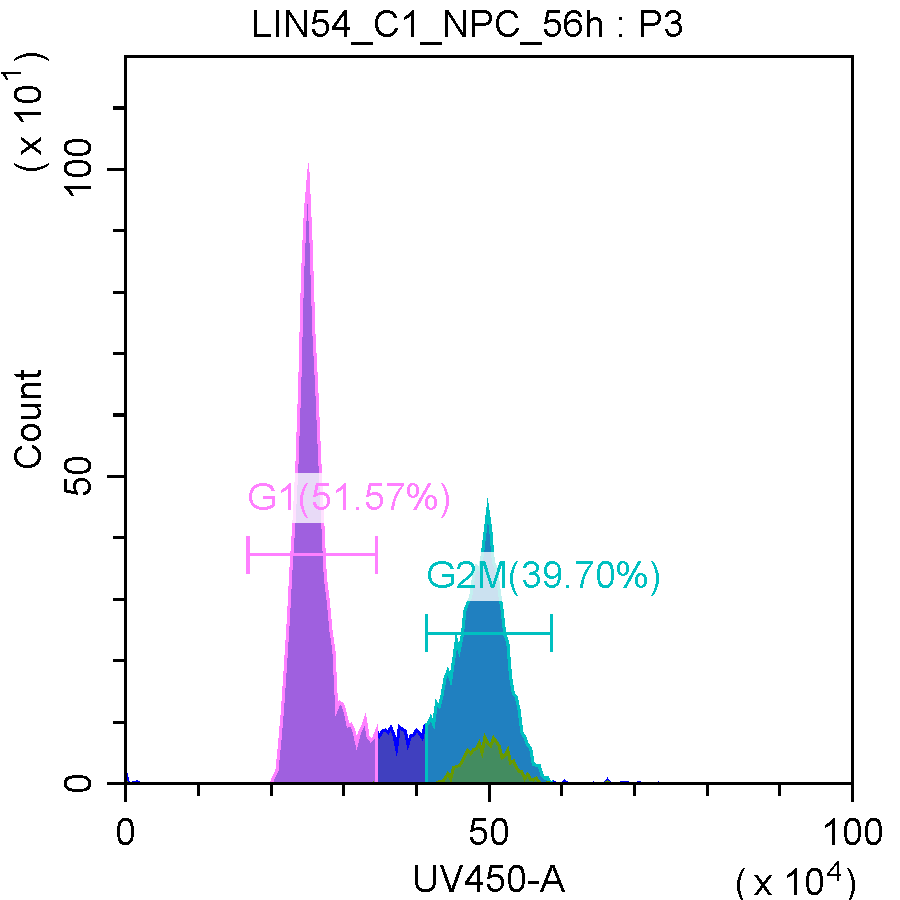

Supplement: Supplementary file 6 — Source data Fig. 4 [file 44318_2025_605_MOESM6_ESM.zip › Fig. 4/4E/Replicate_2/DAPI/LIN54_KO_#1.tif]

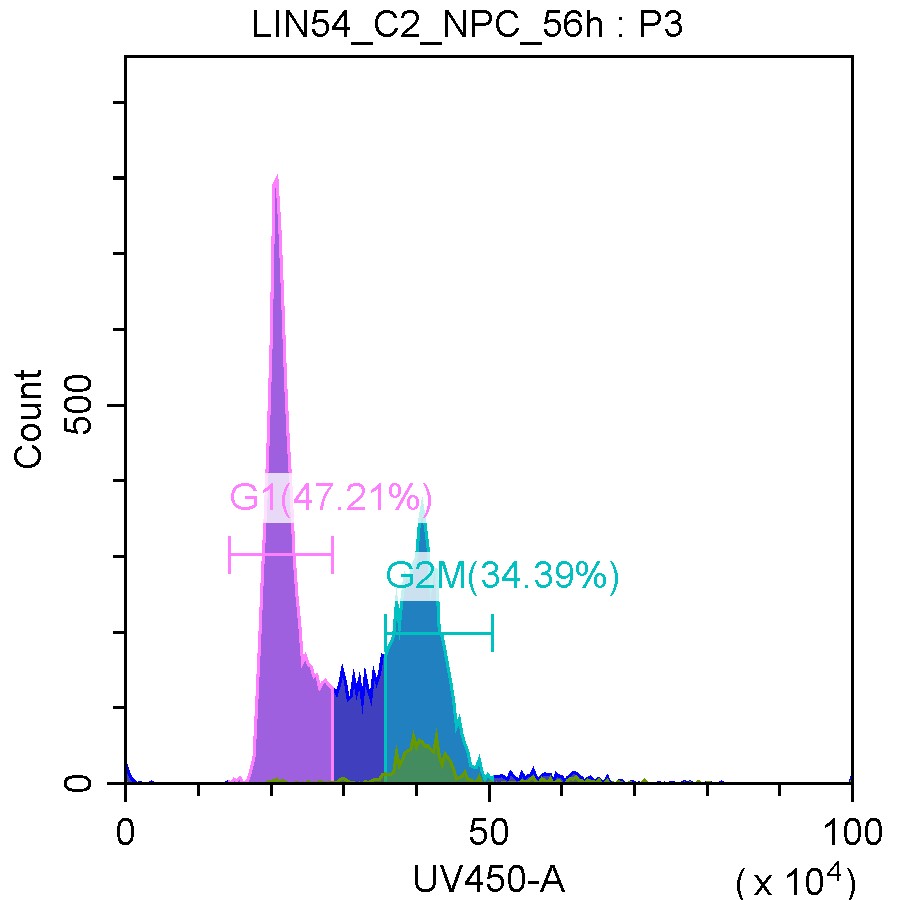

Supplement: Supplementary file 6 — Source data Fig. 4 [file 44318_2025_605_MOESM6_ESM.zip › Fig. 4/4E/Replicate_2/DAPI/LIN54_KO_#2.tif]

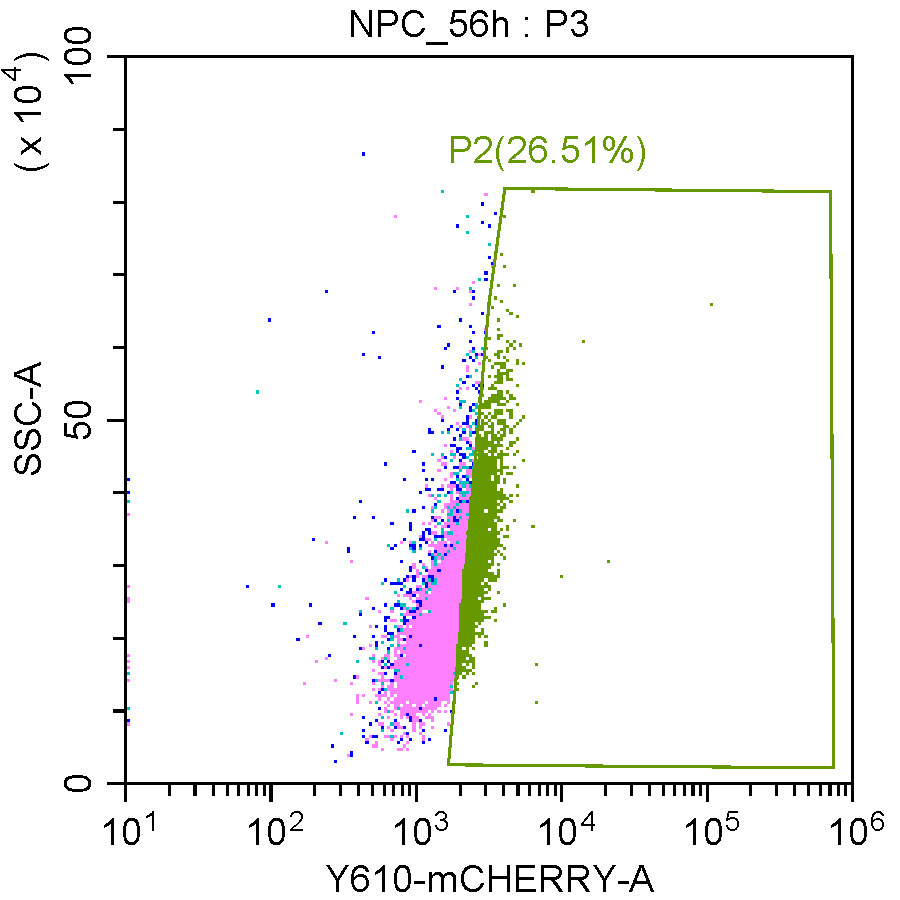

Supplement: Supplementary file 6 — Source data Fig. 4 [file 44318_2025_605_MOESM6_ESM.zip › Fig. 4/4E/Replicate_2/PAX6/Ctrl.tif]

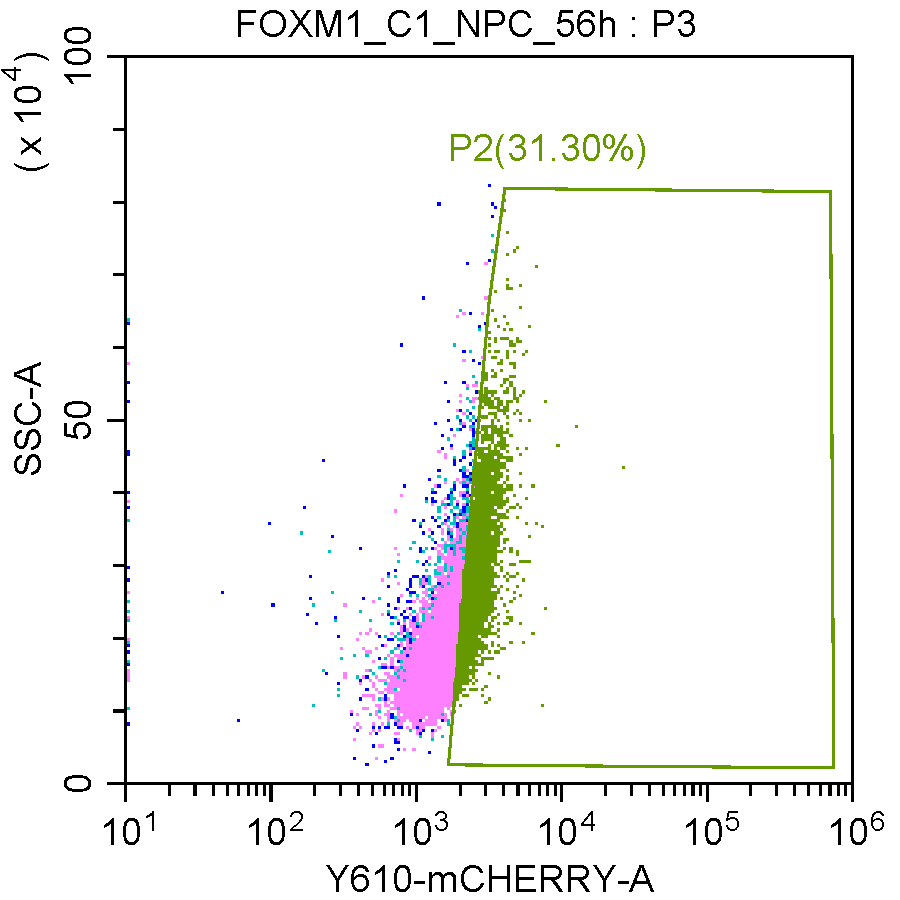

Supplement: Supplementary file 6 — Source data Fig. 4 [file 44318_2025_605_MOESM6_ESM.zip › Fig. 4/4E/Replicate_2/PAX6/FOXM1_KO_#1.tif]
