# Supplementary figures and images for: Post-replicative initial expression of PAX6 during neuroectoderm differentiation (part 2 of 2)
Source: EMBO J. 2025 Oct 21;44(23):7090–118. doi: 10.1038/s44318-025-00605-y (PMC12669799; doi:10.1038/s44318-025-00605-y)

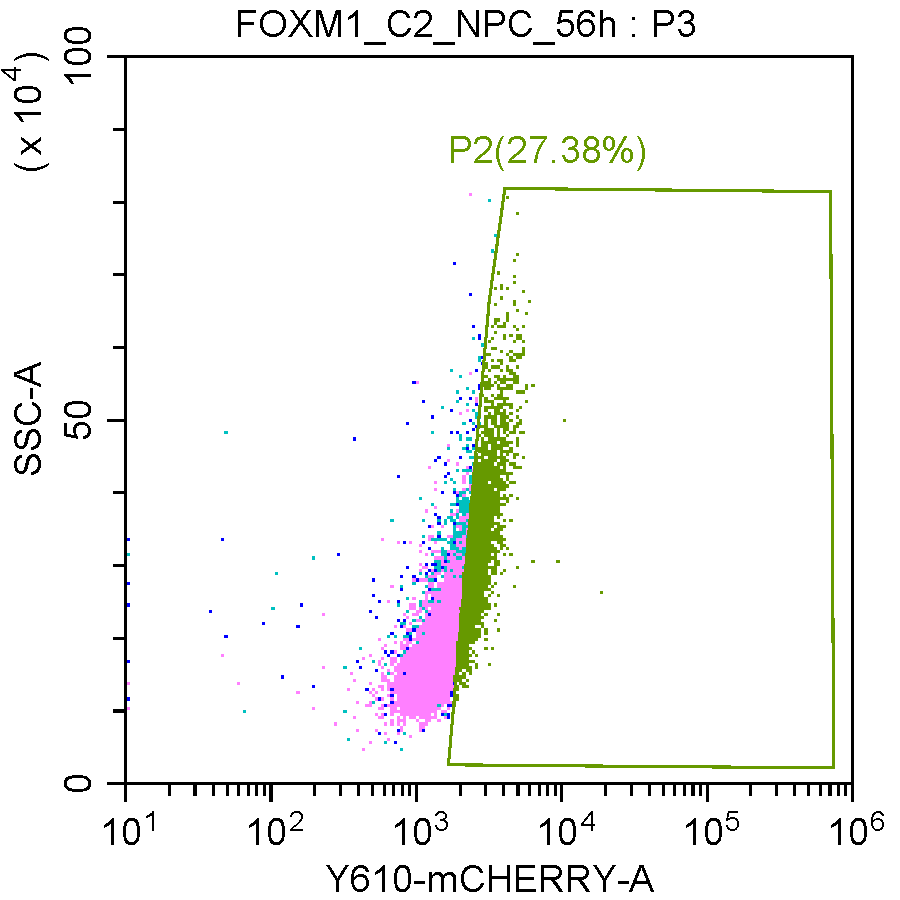

Supplement: Supplementary file 6 — Source data Fig. 4 [file 44318_2025_605_MOESM6_ESM.zip › Fig. 4/4E/Replicate_2/PAX6/FOXM1_KO_#2.tif]

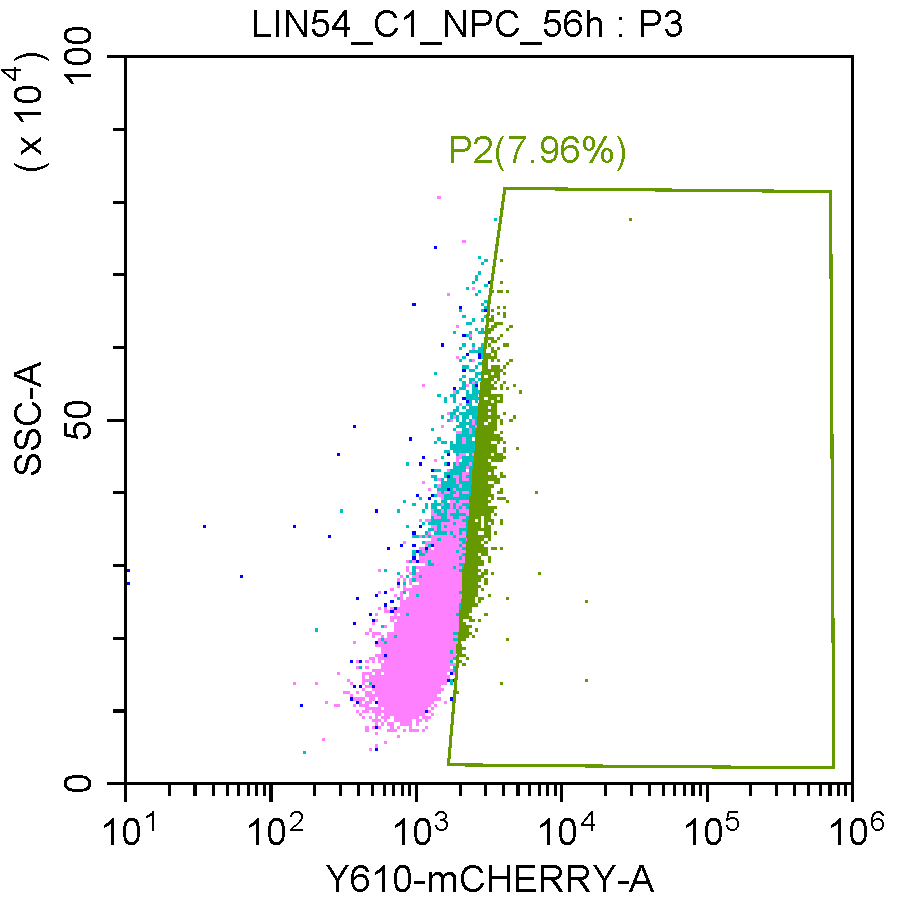

Supplement: Supplementary file 6 — Source data Fig. 4 [file 44318_2025_605_MOESM6_ESM.zip › Fig. 4/4E/Replicate_2/PAX6/LIN54_KO_#1.tif]

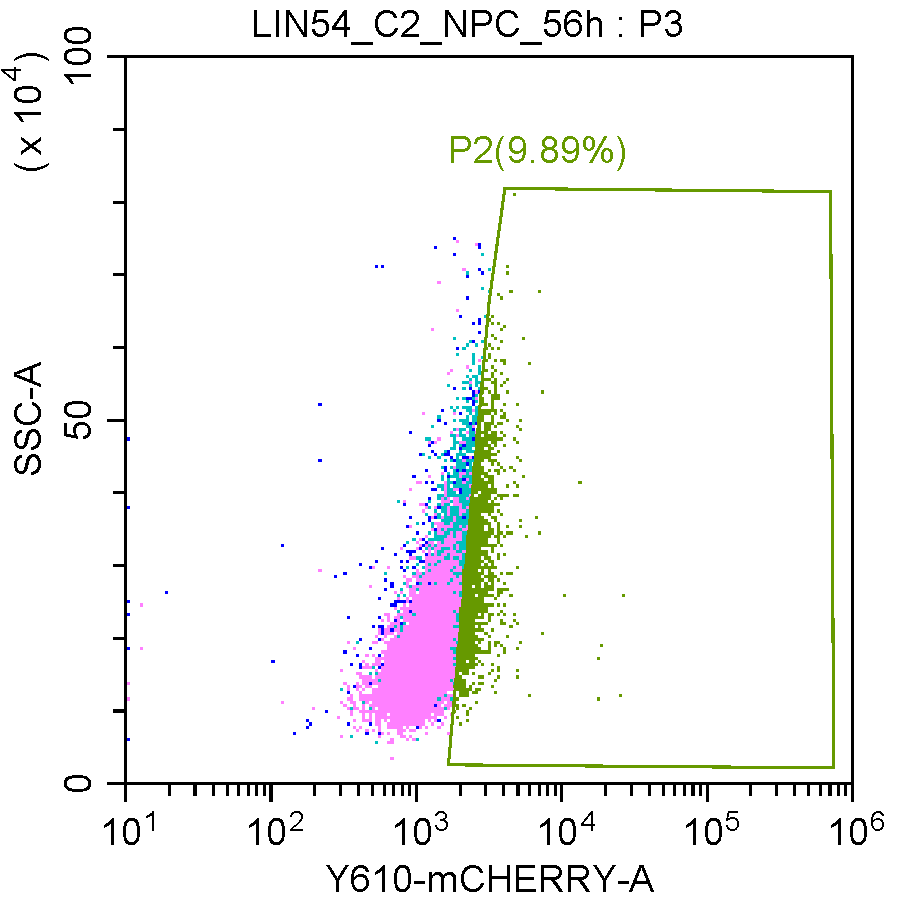

Supplement: Supplementary file 6 — Source data Fig. 4 [file 44318_2025_605_MOESM6_ESM.zip › Fig. 4/4E/Replicate_2/PAX6/LIN54_KO_#2.tif]

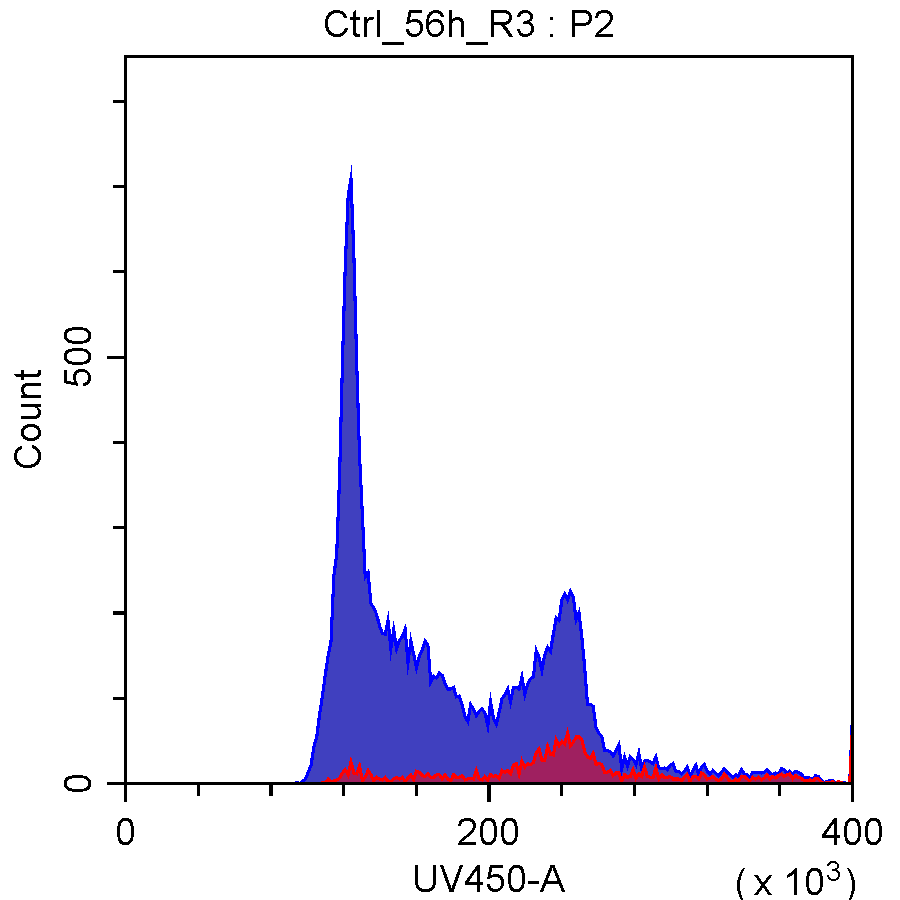

Supplement: Supplementary file 6 — Source data Fig. 4 [file 44318_2025_605_MOESM6_ESM.zip › Fig. 4/4E/Replicate_3/DAPI/Ctrl.tif]

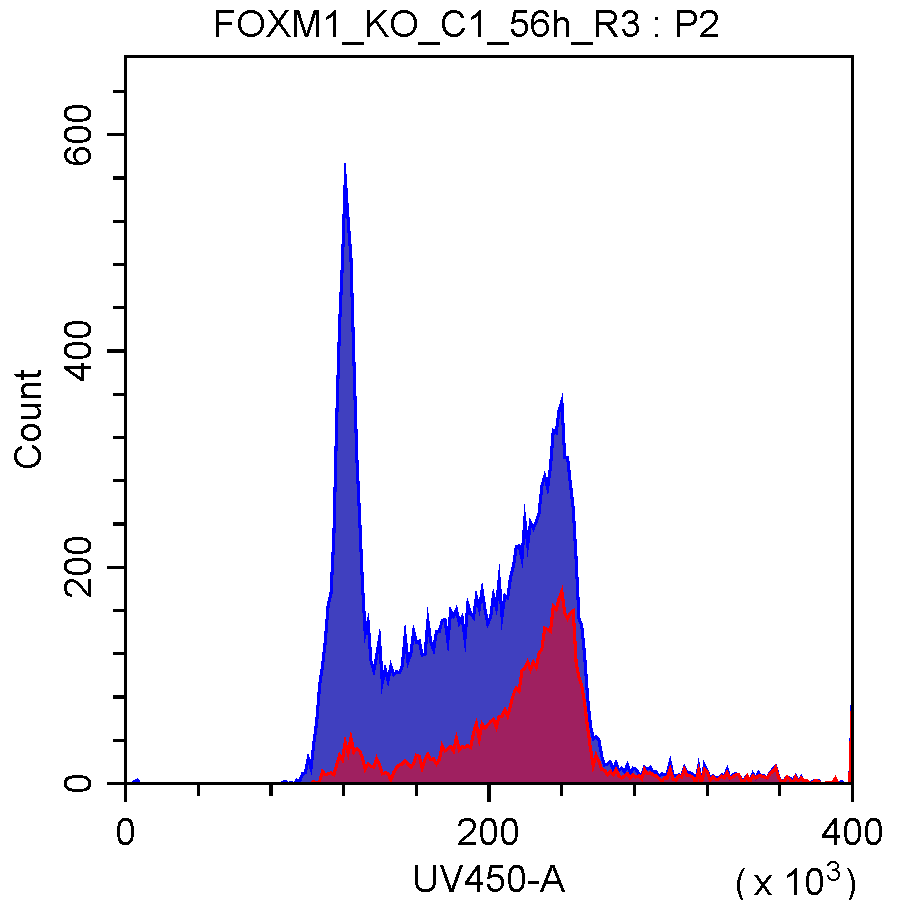

Supplement: Supplementary file 6 — Source data Fig. 4 [file 44318_2025_605_MOESM6_ESM.zip › Fig. 4/4E/Replicate_3/DAPI/FOXM1_KO_#1.tif]

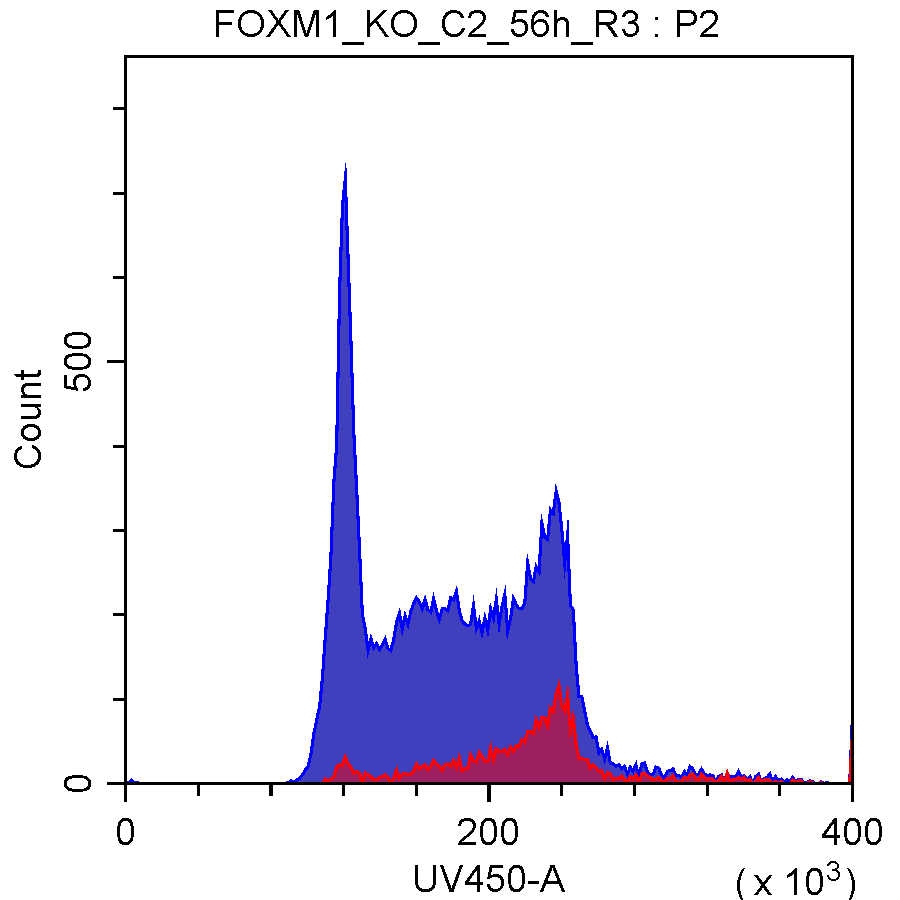

Supplement: Supplementary file 6 — Source data Fig. 4 [file 44318_2025_605_MOESM6_ESM.zip › Fig. 4/4E/Replicate_3/DAPI/FOXM1_KO_#2.tif]

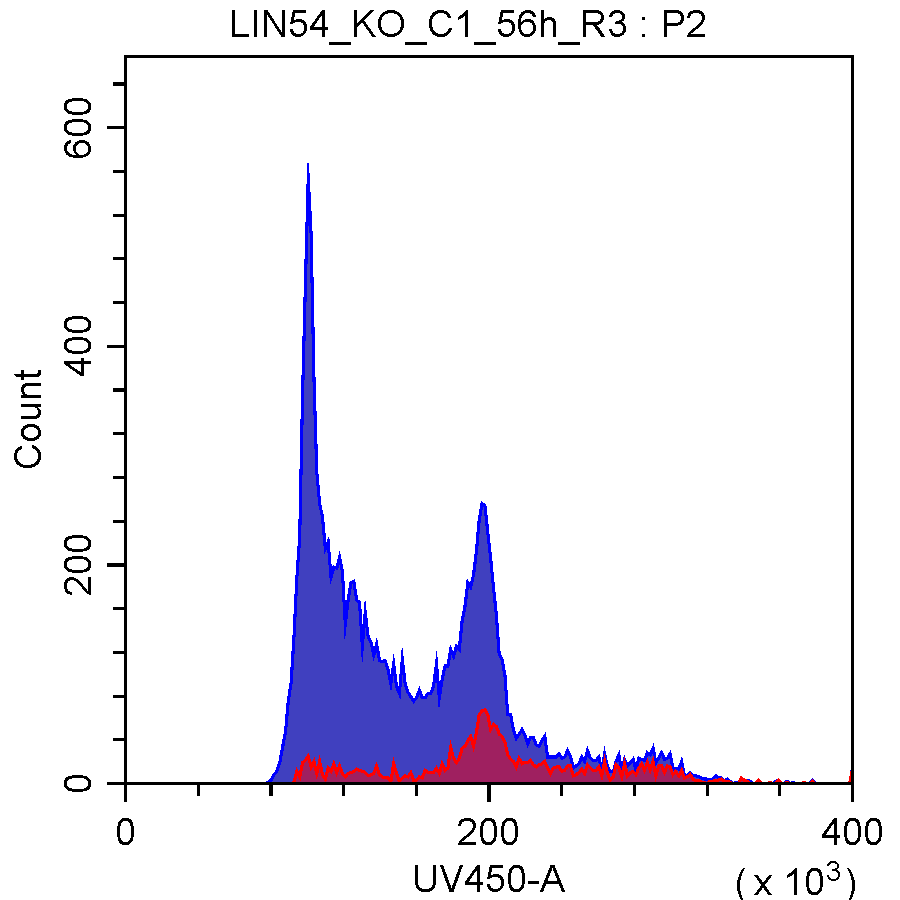

Supplement: Supplementary file 6 — Source data Fig. 4 [file 44318_2025_605_MOESM6_ESM.zip › Fig. 4/4E/Replicate_3/DAPI/LIN54_KO_#1.tif]

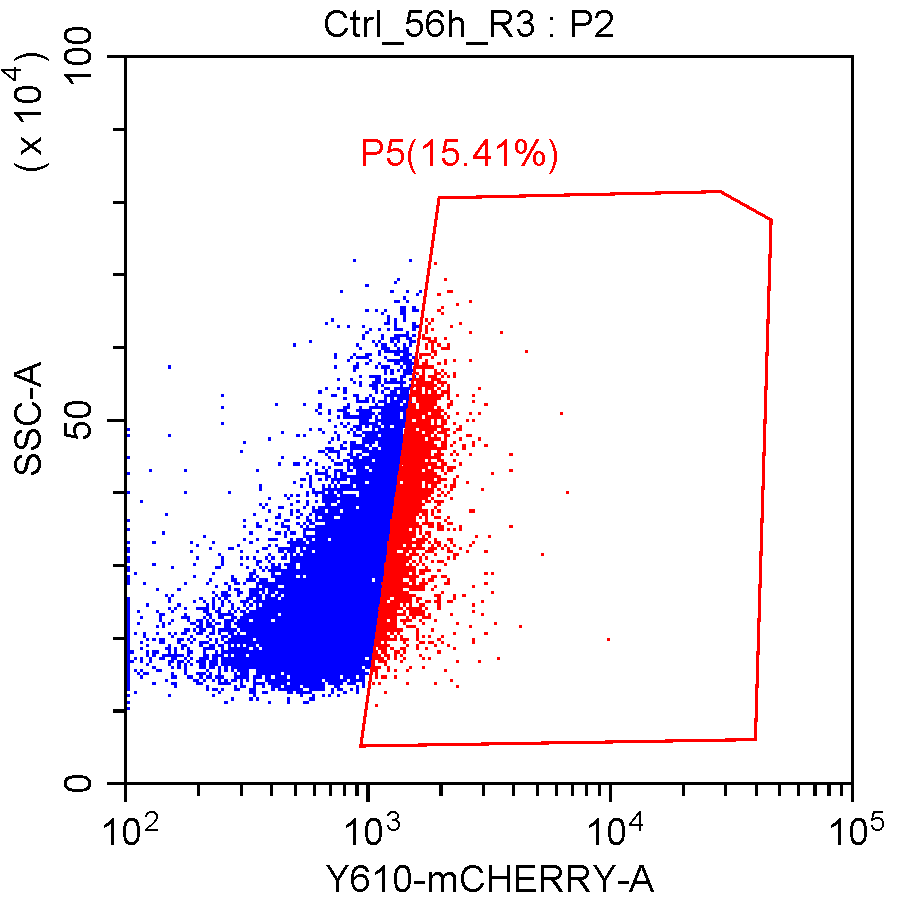

Supplement: Supplementary file 6 — Source data Fig. 4 [file 44318_2025_605_MOESM6_ESM.zip › Fig. 4/4E/Replicate_3/PAX6/Ctrl.tif]

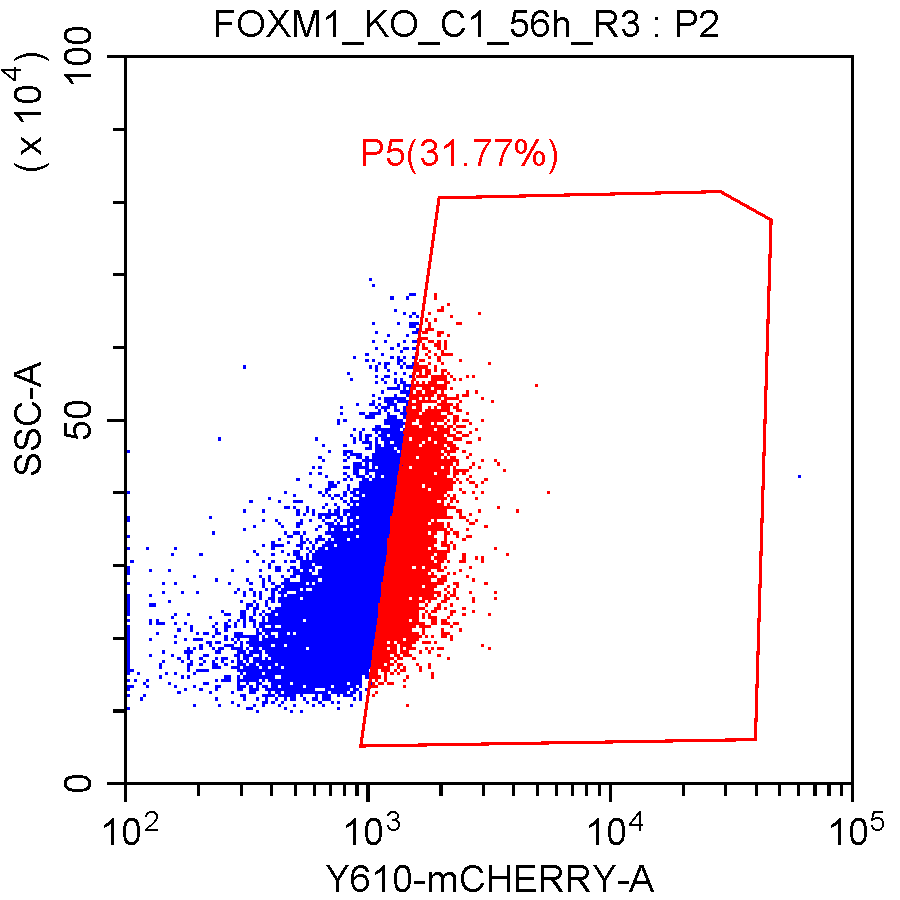

Supplement: Supplementary file 6 — Source data Fig. 4 [file 44318_2025_605_MOESM6_ESM.zip › Fig. 4/4E/Replicate_3/PAX6/FOXM1_KO_#1.tif]

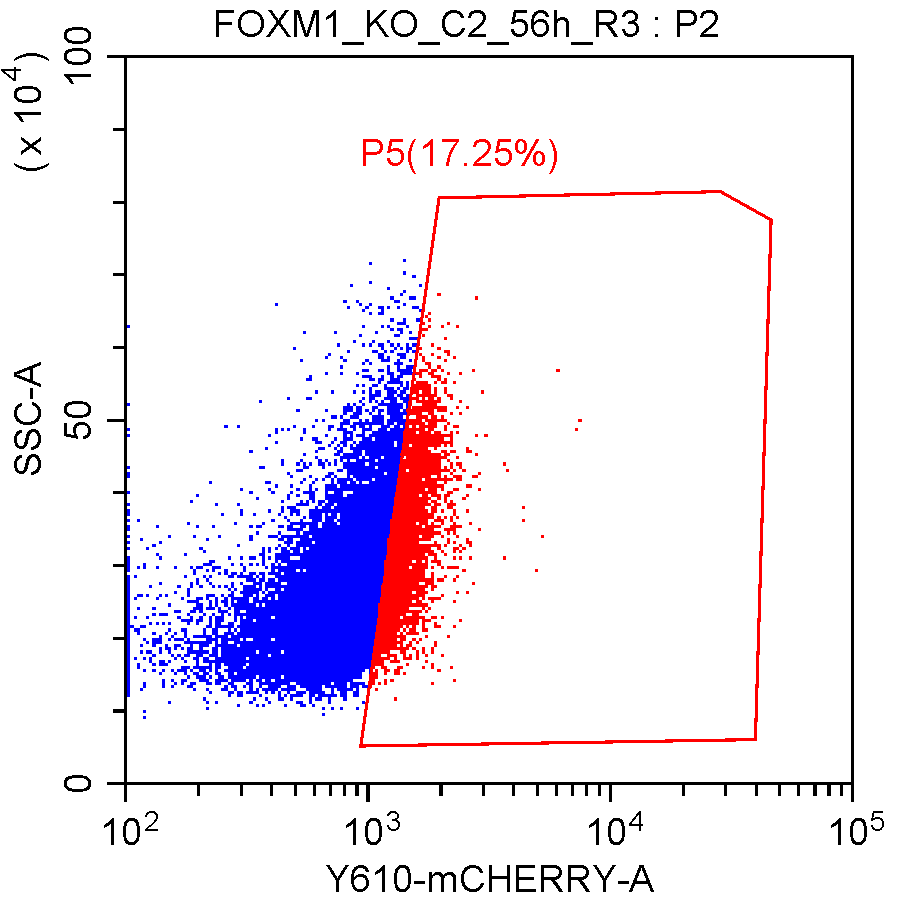

Supplement: Supplementary file 6 — Source data Fig. 4 [file 44318_2025_605_MOESM6_ESM.zip › Fig. 4/4E/Replicate_3/PAX6/FOXM1_KO_#2.tif]

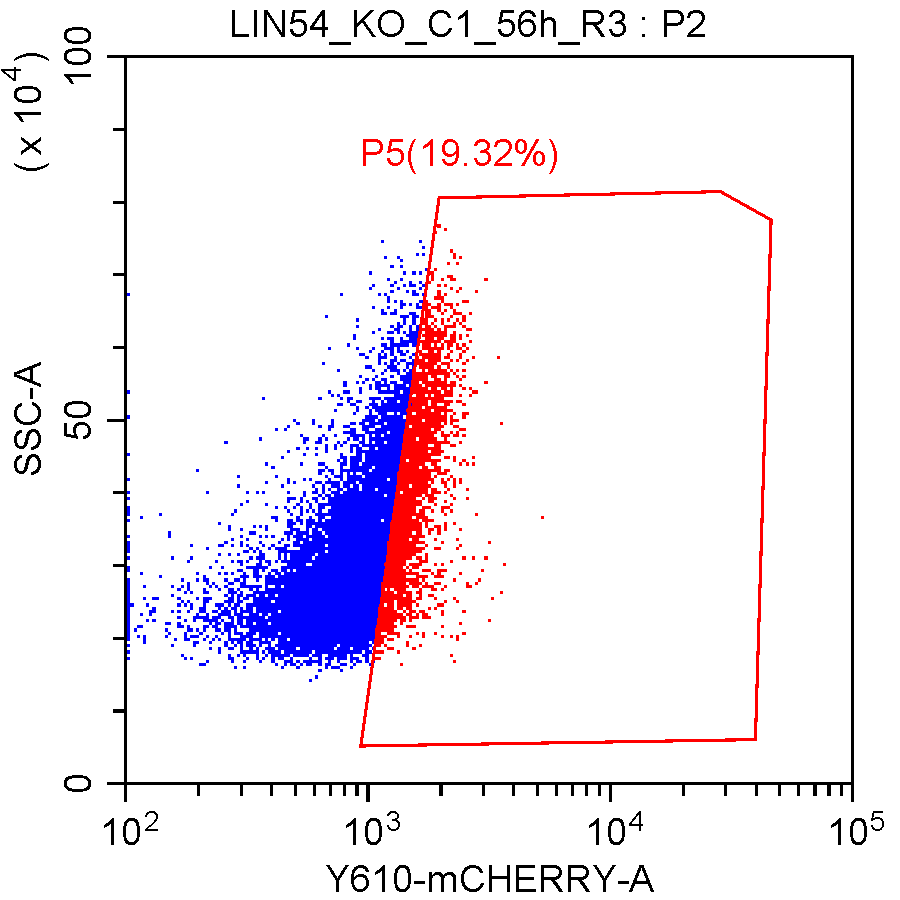

Supplement: Supplementary file 6 — Source data Fig. 4 [file 44318_2025_605_MOESM6_ESM.zip › Fig. 4/4E/Replicate_3/PAX6/LIN54_KO_#1.tif]

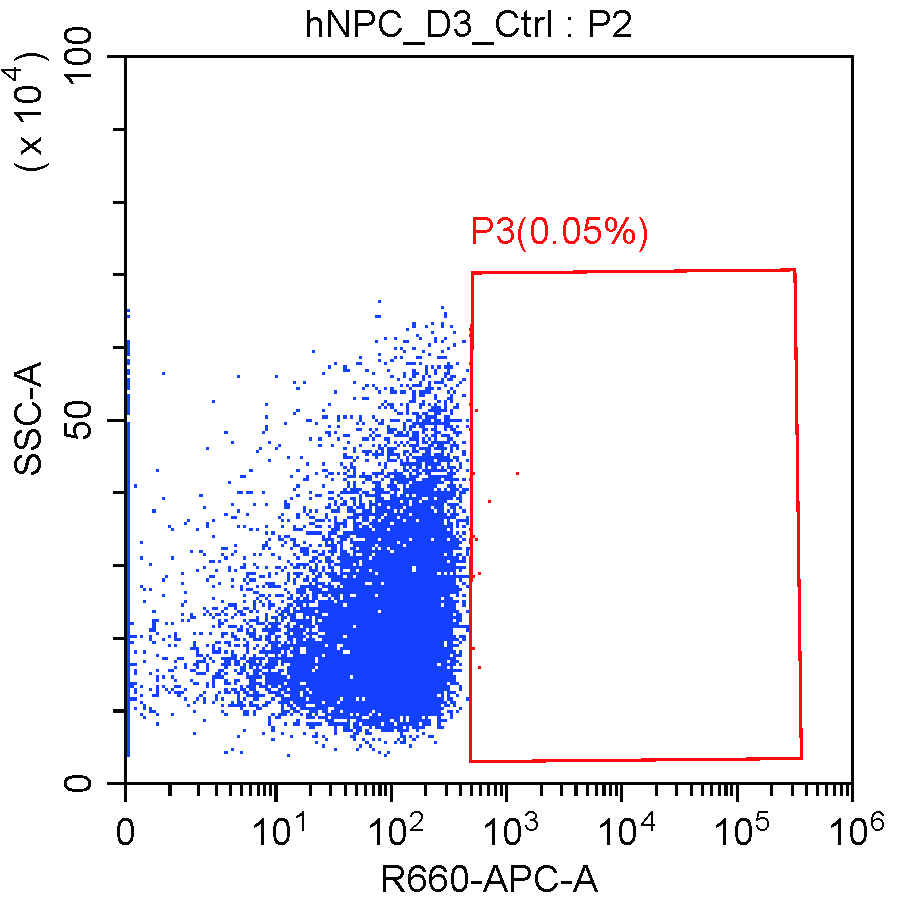

Supplement: Supplementary file 7 — Source data Fig. 5 [file 44318_2025_605_MOESM7_ESM.zip › SD figure 5_Revised/Fig. 5/5B/P500_Δ50 bp in NPC_D3/Replicate_1/Negative control_1.tif]

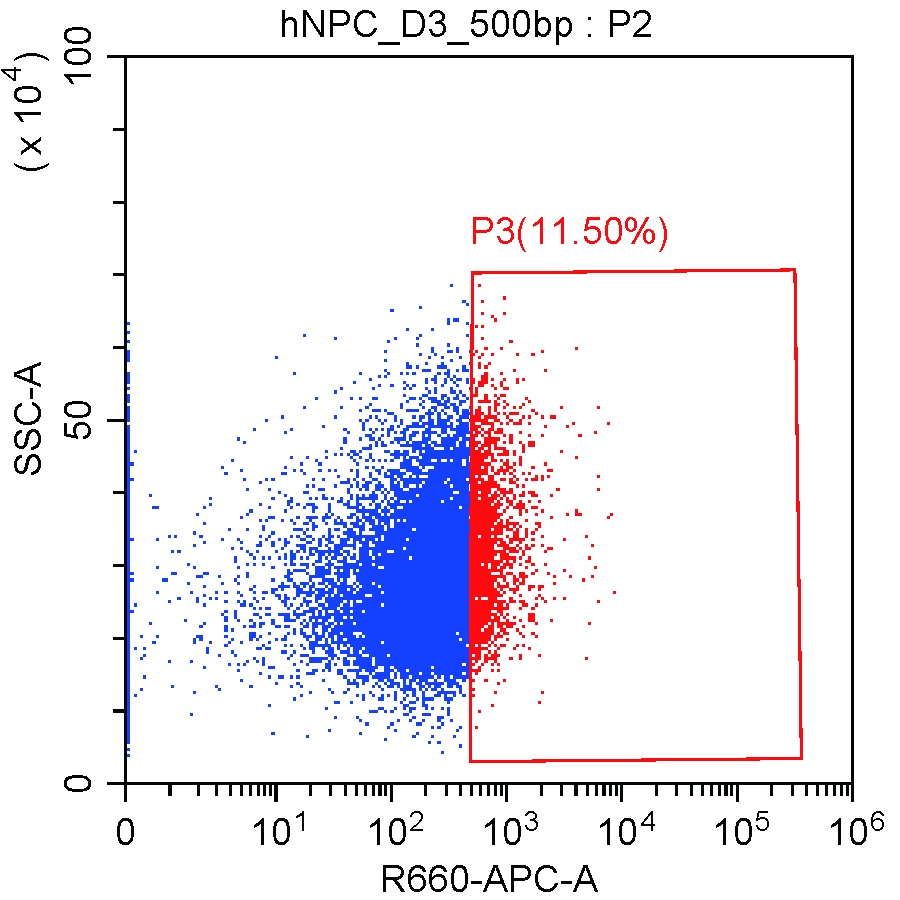

Supplement: Supplementary file 7 — Source data Fig. 5 [file 44318_2025_605_MOESM7_ESM.zip › SD figure 5_Revised/Fig. 5/5B/P500_Δ50 bp in NPC_D3/Replicate_1/P500_1.tif]

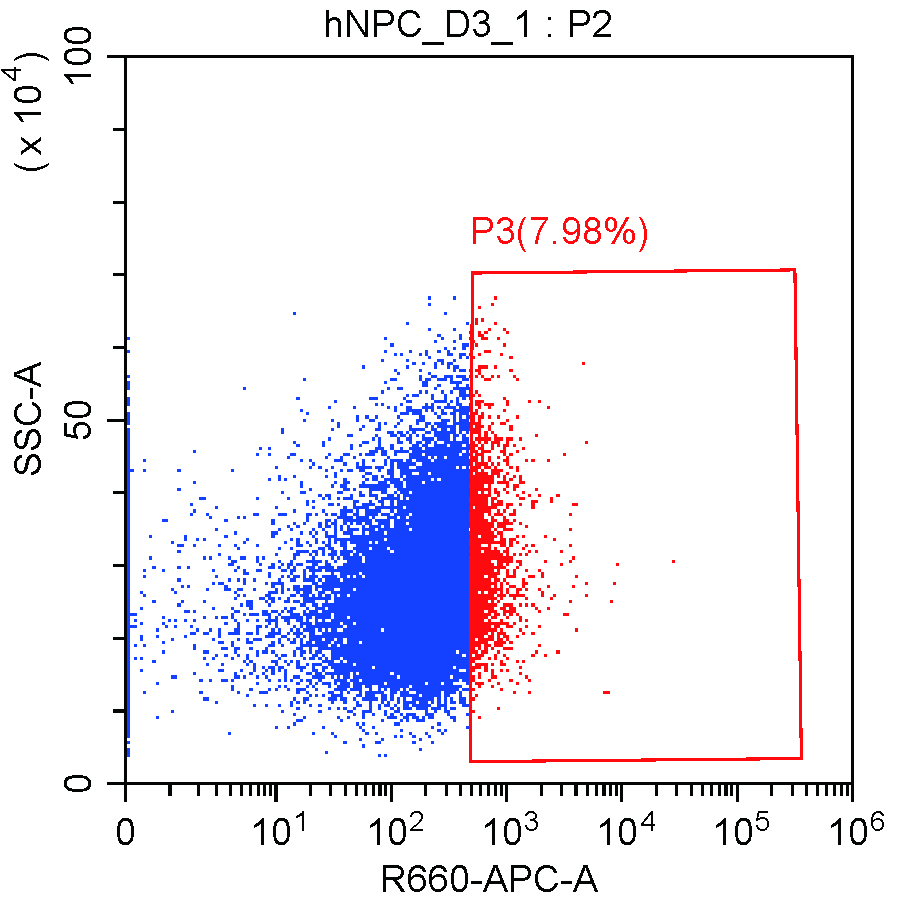

Supplement: Supplementary file 7 — Source data Fig. 5 [file 44318_2025_605_MOESM7_ESM.zip › SD figure 5_Revised/Fig. 5/5B/P500_Δ50 bp in NPC_D3/Replicate_1/Δ1_1.tif]

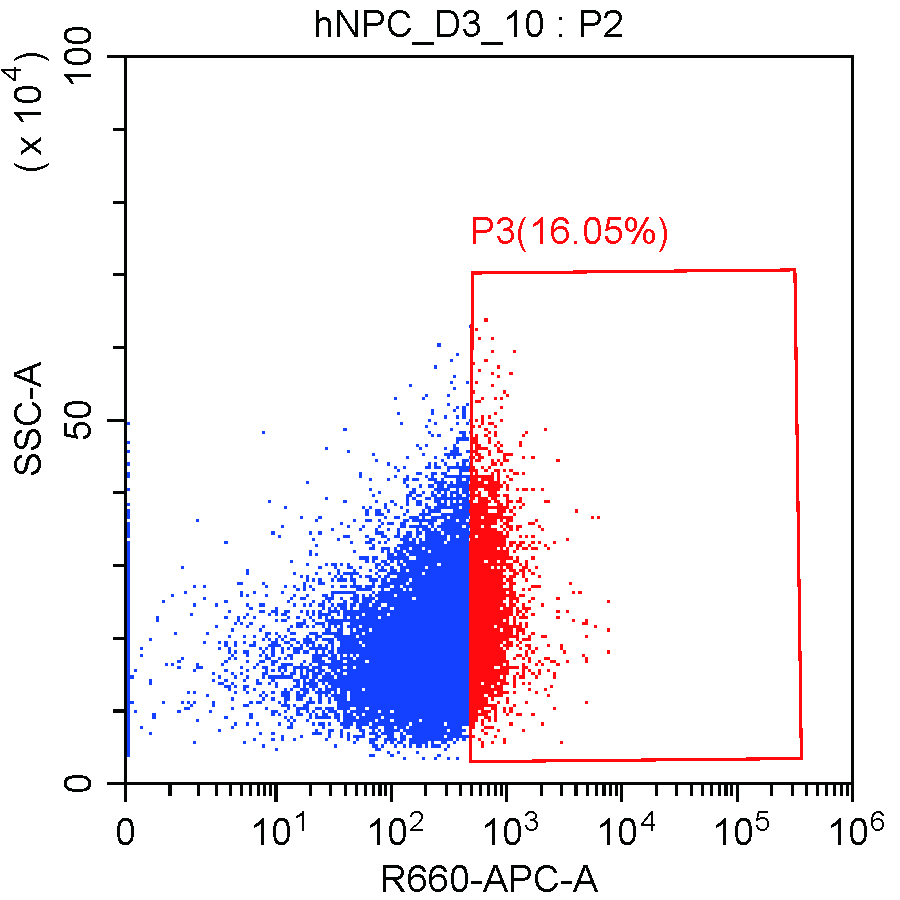

Supplement: Supplementary file 7 — Source data Fig. 5 [file 44318_2025_605_MOESM7_ESM.zip › SD figure 5_Revised/Fig. 5/5B/P500_Δ50 bp in NPC_D3/Replicate_1/Δ1_10.tif]

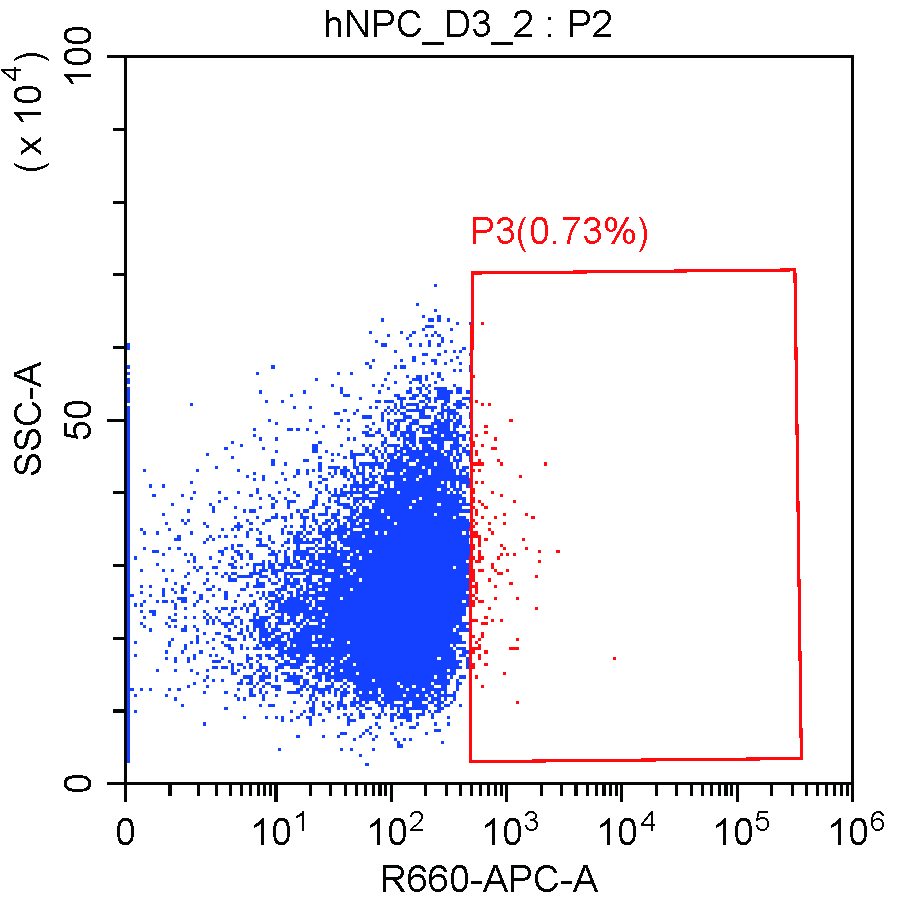

Supplement: Supplementary file 7 — Source data Fig. 5 [file 44318_2025_605_MOESM7_ESM.zip › SD figure 5_Revised/Fig. 5/5B/P500_Δ50 bp in NPC_D3/Replicate_1/Δ1_2.tif]

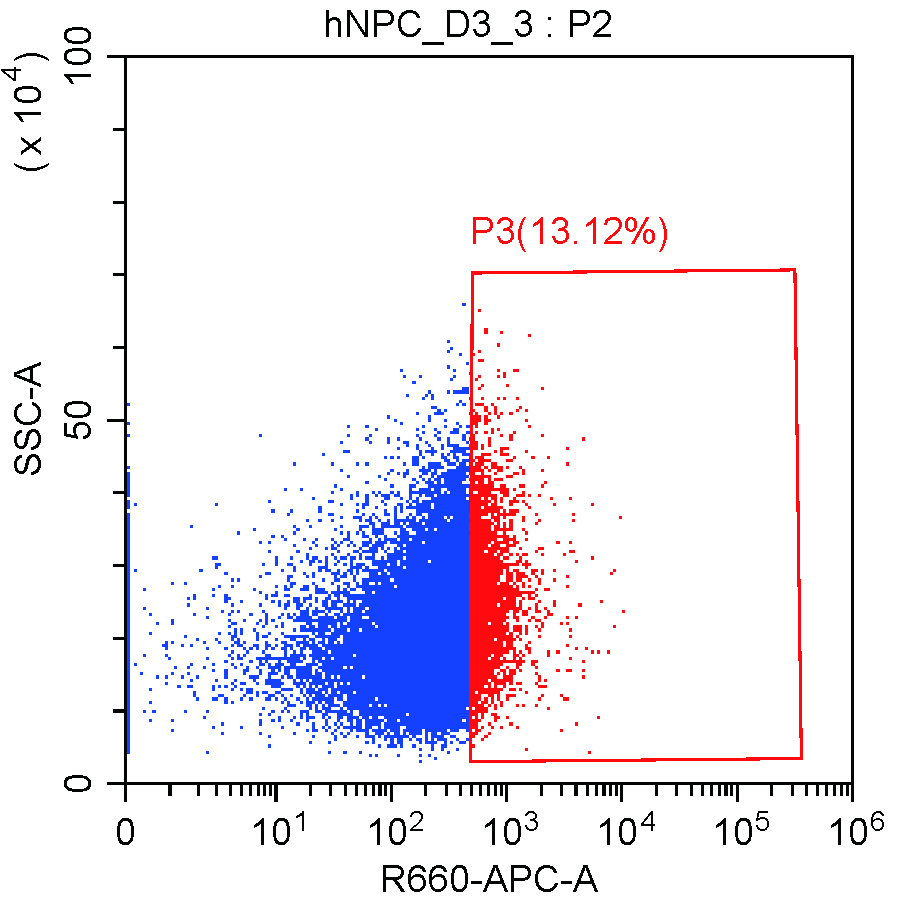

Supplement: Supplementary file 7 — Source data Fig. 5 [file 44318_2025_605_MOESM7_ESM.zip › SD figure 5_Revised/Fig. 5/5B/P500_Δ50 bp in NPC_D3/Replicate_1/Δ1_3.tif]

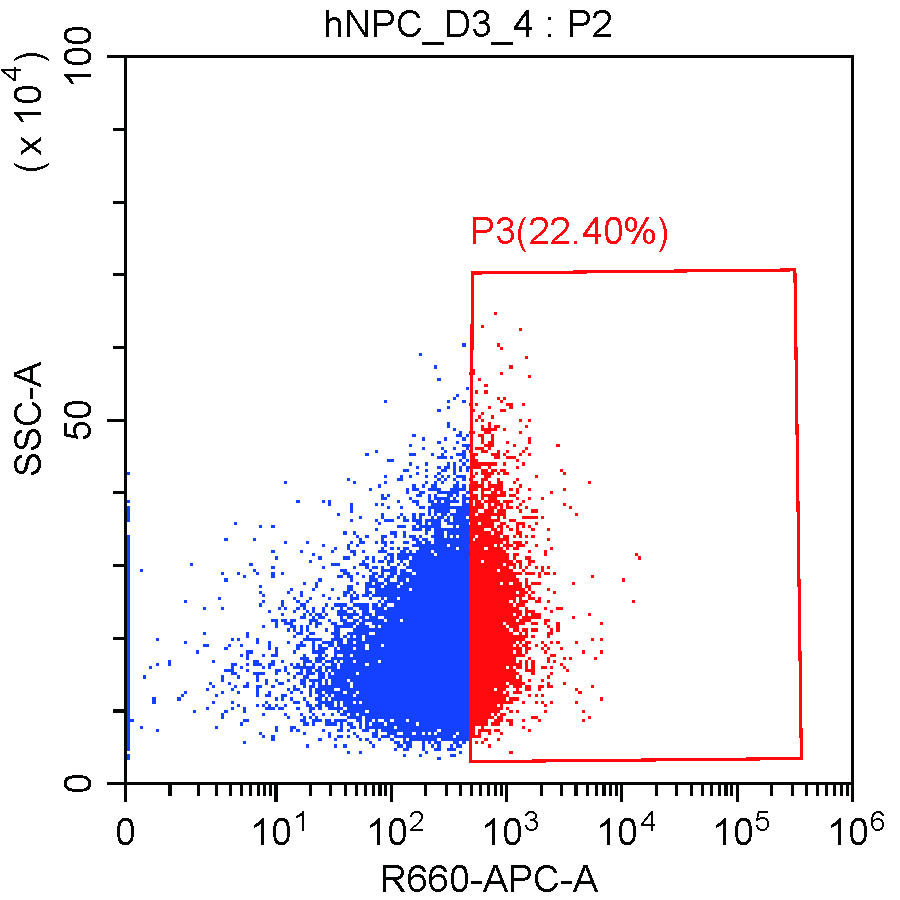

Supplement: Supplementary file 7 — Source data Fig. 5 [file 44318_2025_605_MOESM7_ESM.zip › SD figure 5_Revised/Fig. 5/5B/P500_Δ50 bp in NPC_D3/Replicate_1/Δ1_4.tif]

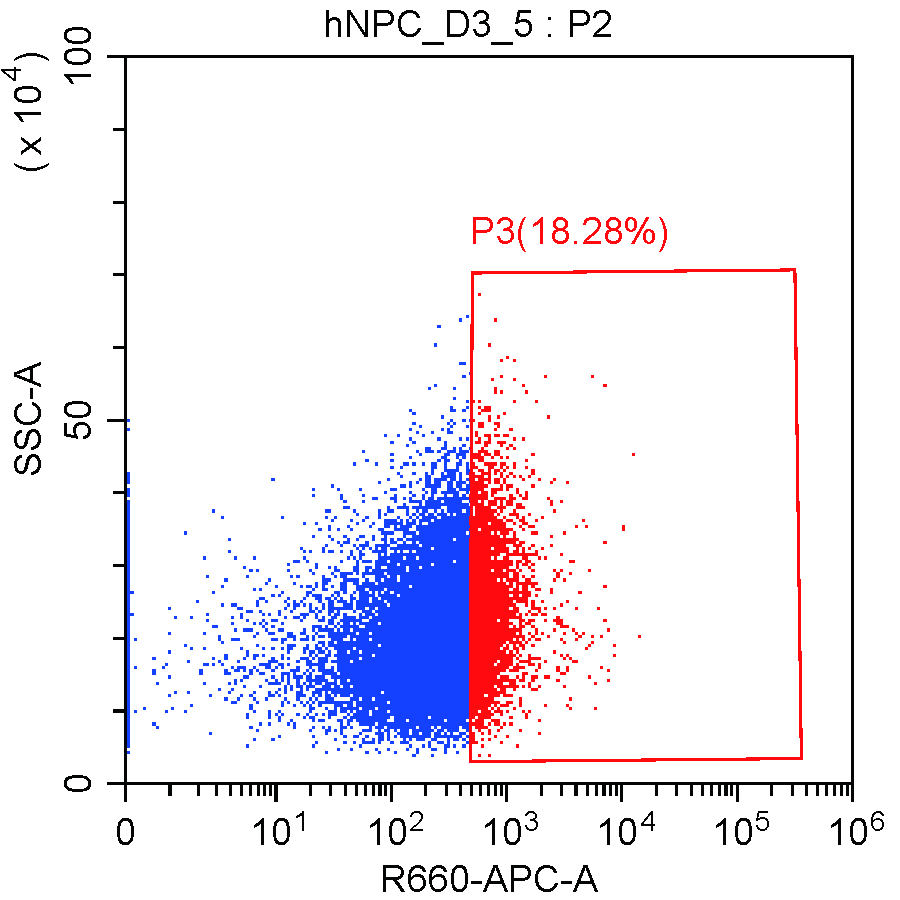

Supplement: Supplementary file 7 — Source data Fig. 5 [file 44318_2025_605_MOESM7_ESM.zip › SD figure 5_Revised/Fig. 5/5B/P500_Δ50 bp in NPC_D3/Replicate_1/Δ1_5.tif]

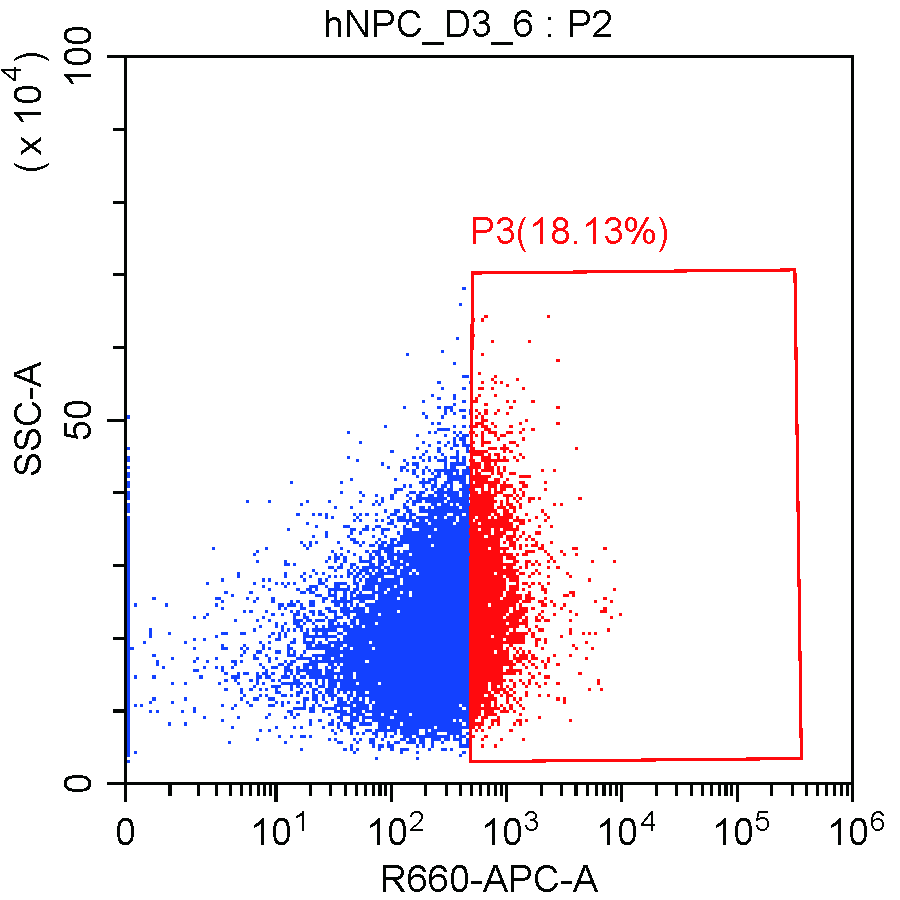

Supplement: Supplementary file 7 — Source data Fig. 5 [file 44318_2025_605_MOESM7_ESM.zip › SD figure 5_Revised/Fig. 5/5B/P500_Δ50 bp in NPC_D3/Replicate_1/Δ1_6.tif]

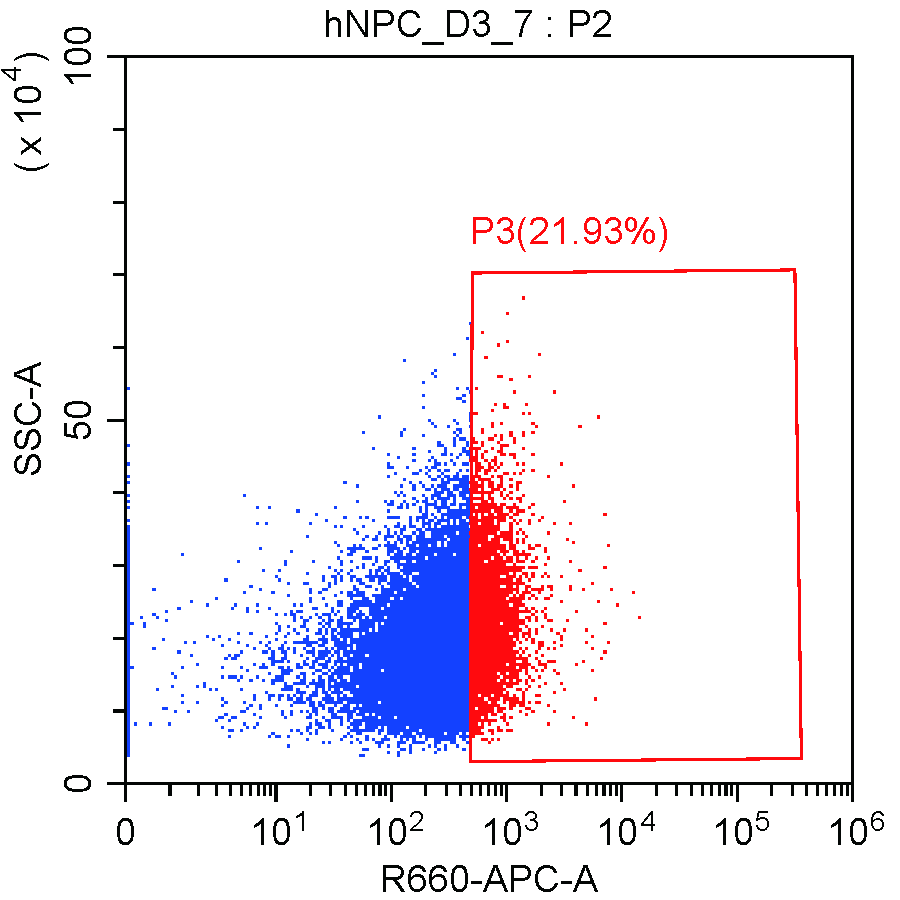

Supplement: Supplementary file 7 — Source data Fig. 5 [file 44318_2025_605_MOESM7_ESM.zip › SD figure 5_Revised/Fig. 5/5B/P500_Δ50 bp in NPC_D3/Replicate_1/Δ1_7.tif]

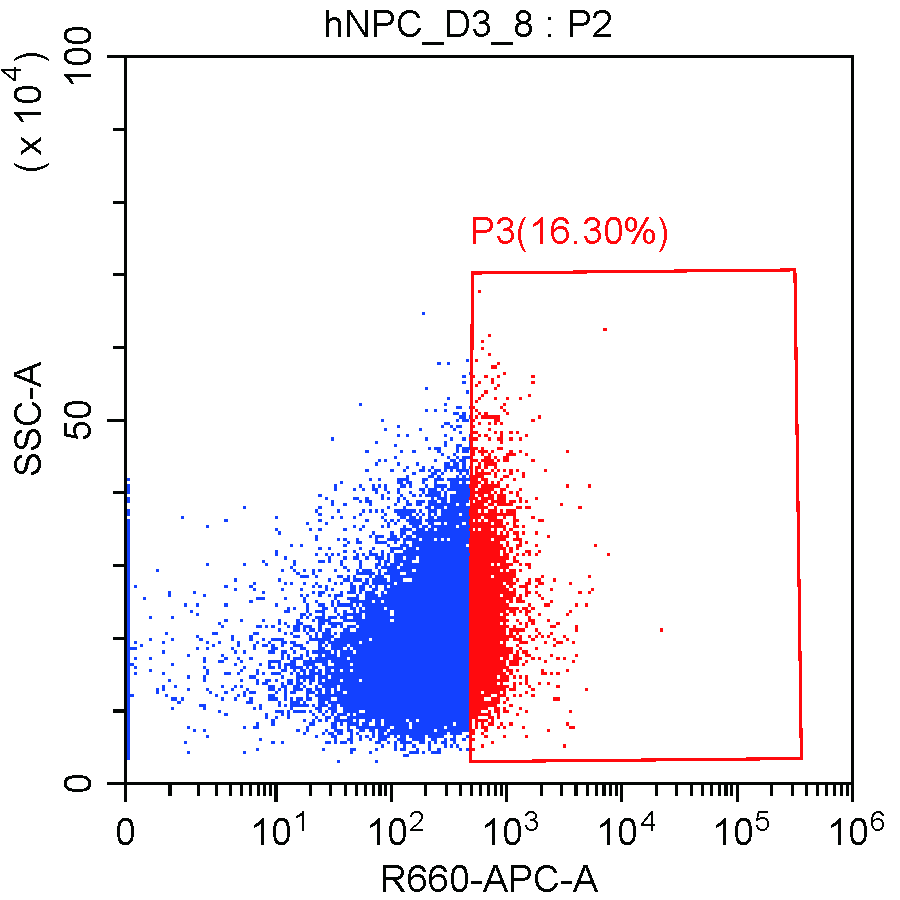

Supplement: Supplementary file 7 — Source data Fig. 5 [file 44318_2025_605_MOESM7_ESM.zip › SD figure 5_Revised/Fig. 5/5B/P500_Δ50 bp in NPC_D3/Replicate_1/Δ1_8.tif]

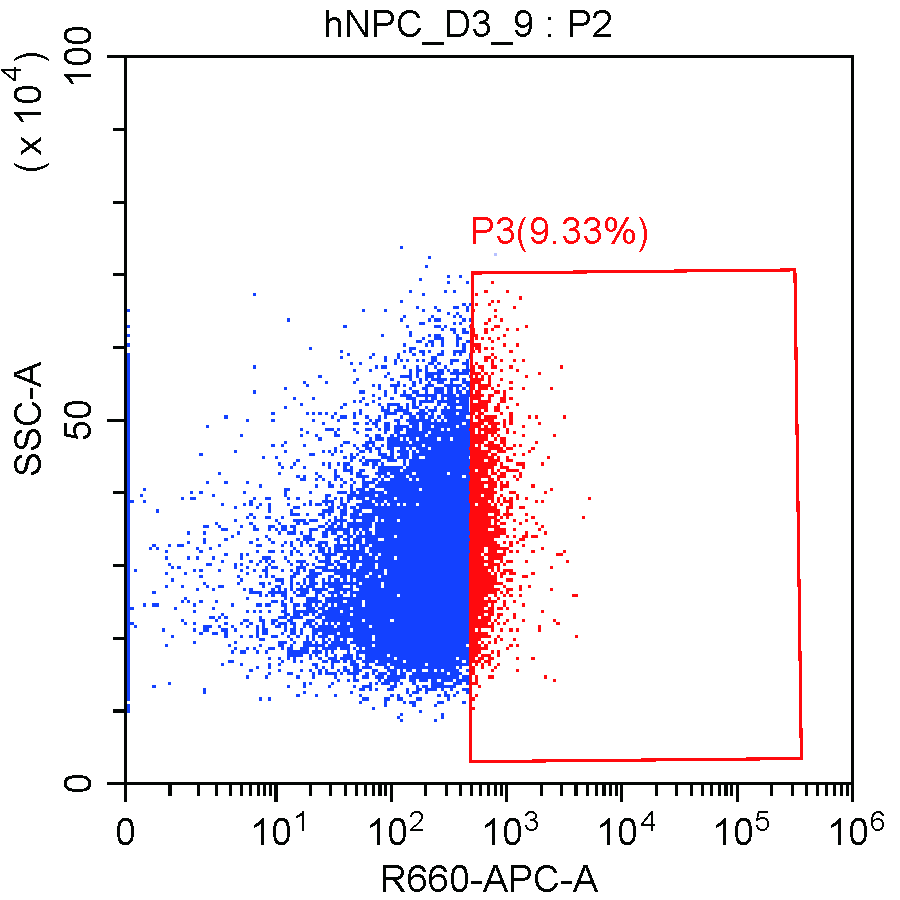

Supplement: Supplementary file 7 — Source data Fig. 5 [file 44318_2025_605_MOESM7_ESM.zip › SD figure 5_Revised/Fig. 5/5B/P500_Δ50 bp in NPC_D3/Replicate_1/Δ1_9.tif]

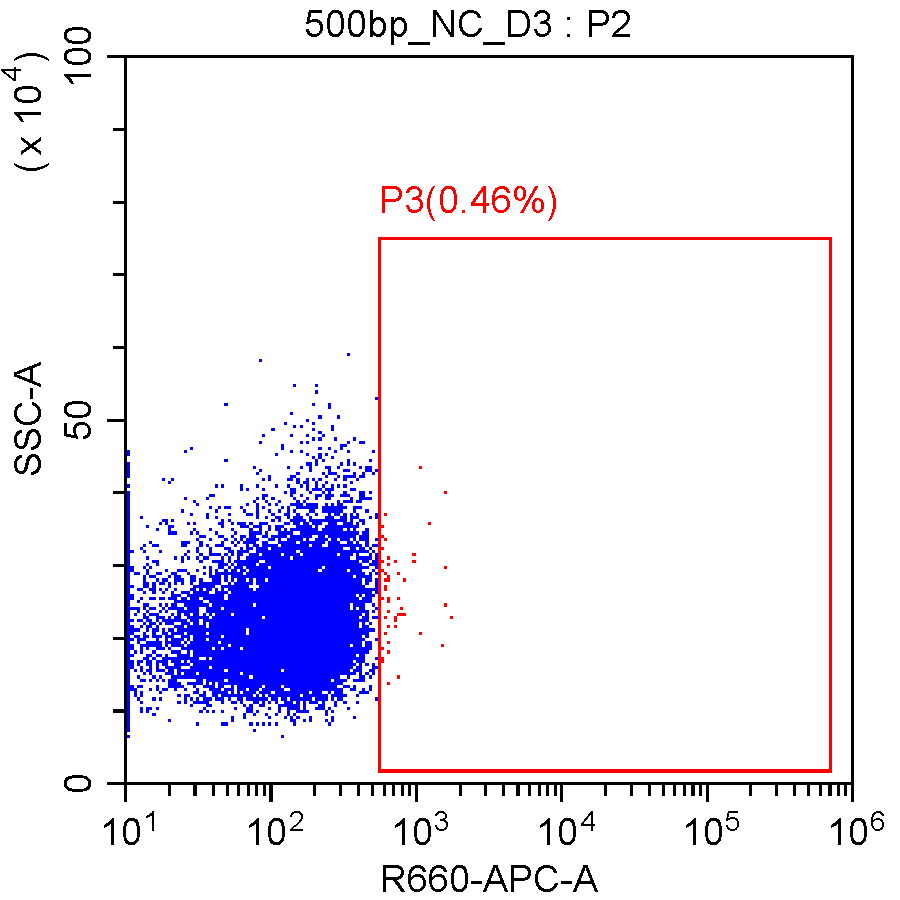

Supplement: Supplementary file 7 — Source data Fig. 5 [file 44318_2025_605_MOESM7_ESM.zip › SD figure 5_Revised/Fig. 5/5B/P500_Δ50 bp in NPC_D3/Replicate_2/Negative control_2.tiff]

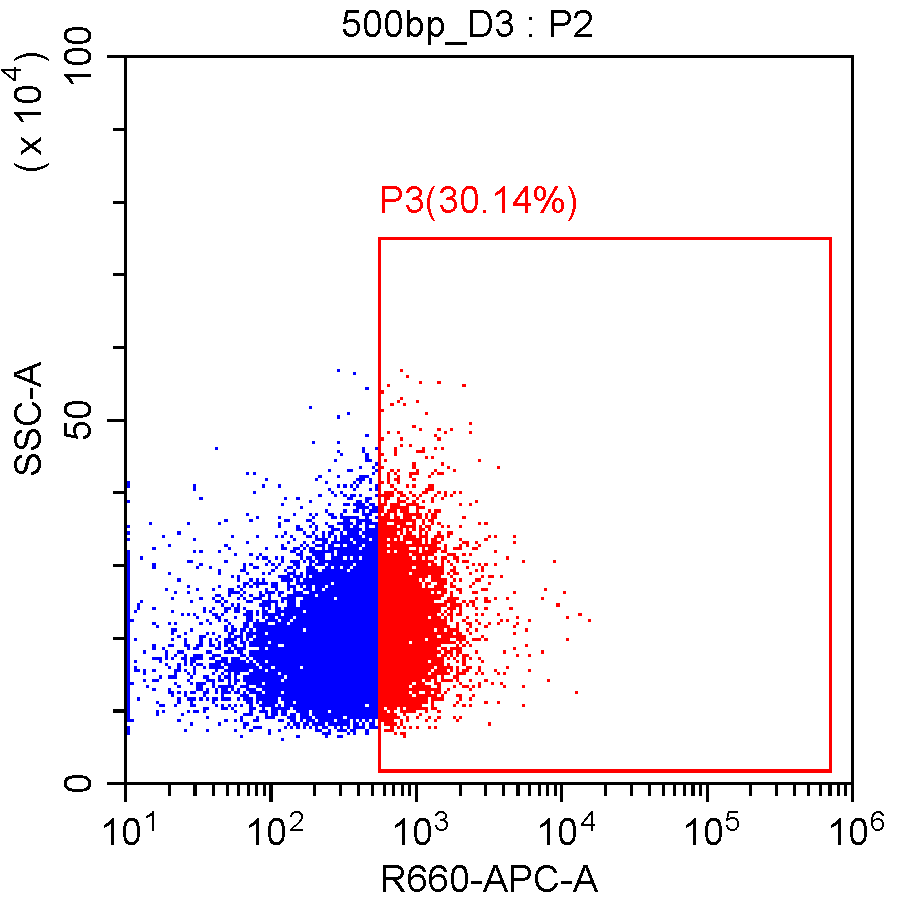

Supplement: Supplementary file 7 — Source data Fig. 5 [file 44318_2025_605_MOESM7_ESM.zip › SD figure 5_Revised/Fig. 5/5B/P500_Δ50 bp in NPC_D3/Replicate_2/P500_2.tiff]

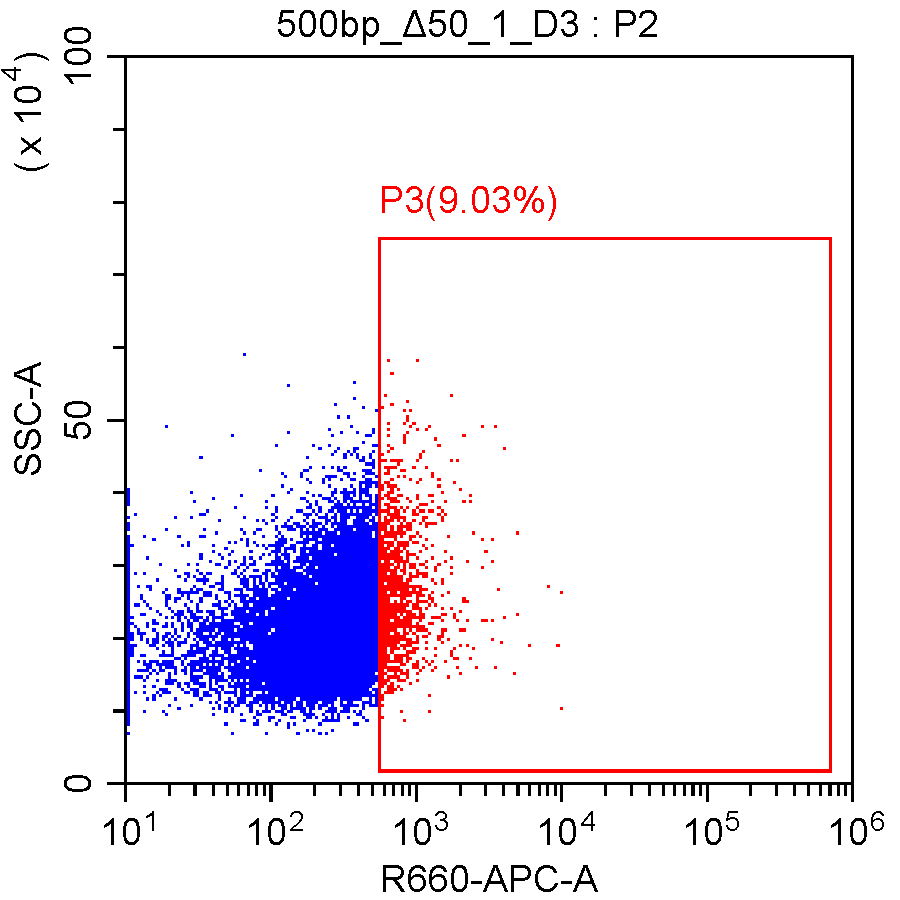

Supplement: Supplementary file 7 — Source data Fig. 5 [file 44318_2025_605_MOESM7_ESM.zip › SD figure 5_Revised/Fig. 5/5B/P500_Δ50 bp in NPC_D3/Replicate_2/Δ1_1.tiff]

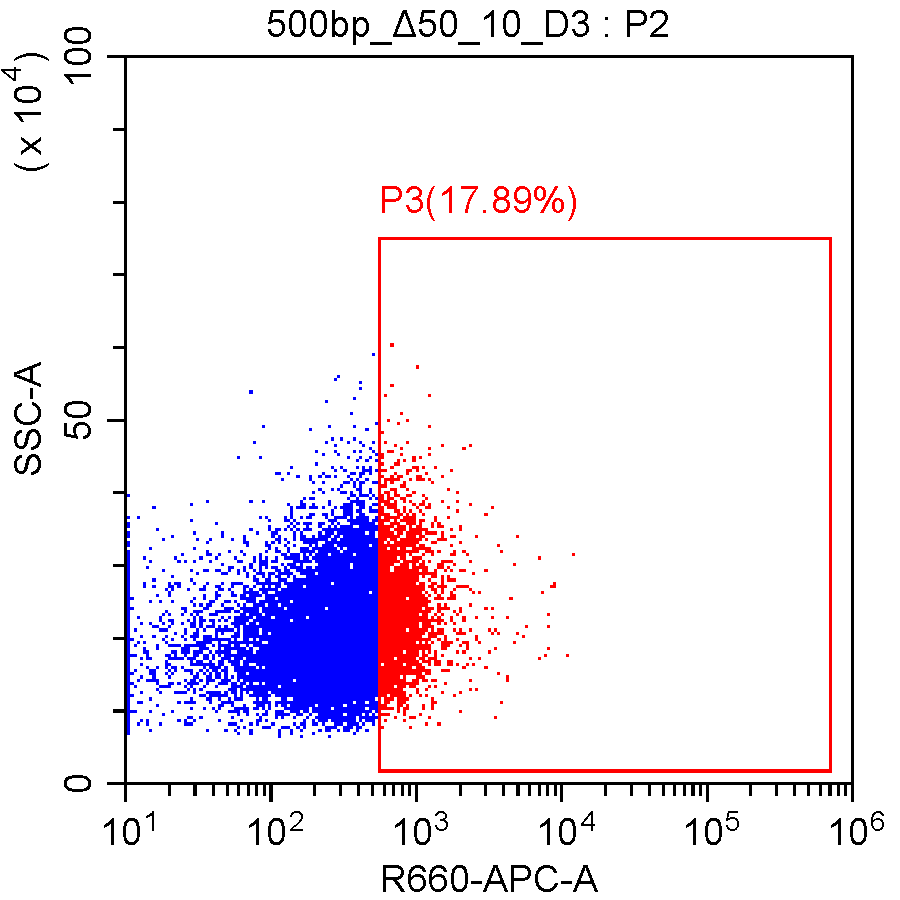

Supplement: Supplementary file 7 — Source data Fig. 5 [file 44318_2025_605_MOESM7_ESM.zip › SD figure 5_Revised/Fig. 5/5B/P500_Δ50 bp in NPC_D3/Replicate_2/Δ1_10.tiff]

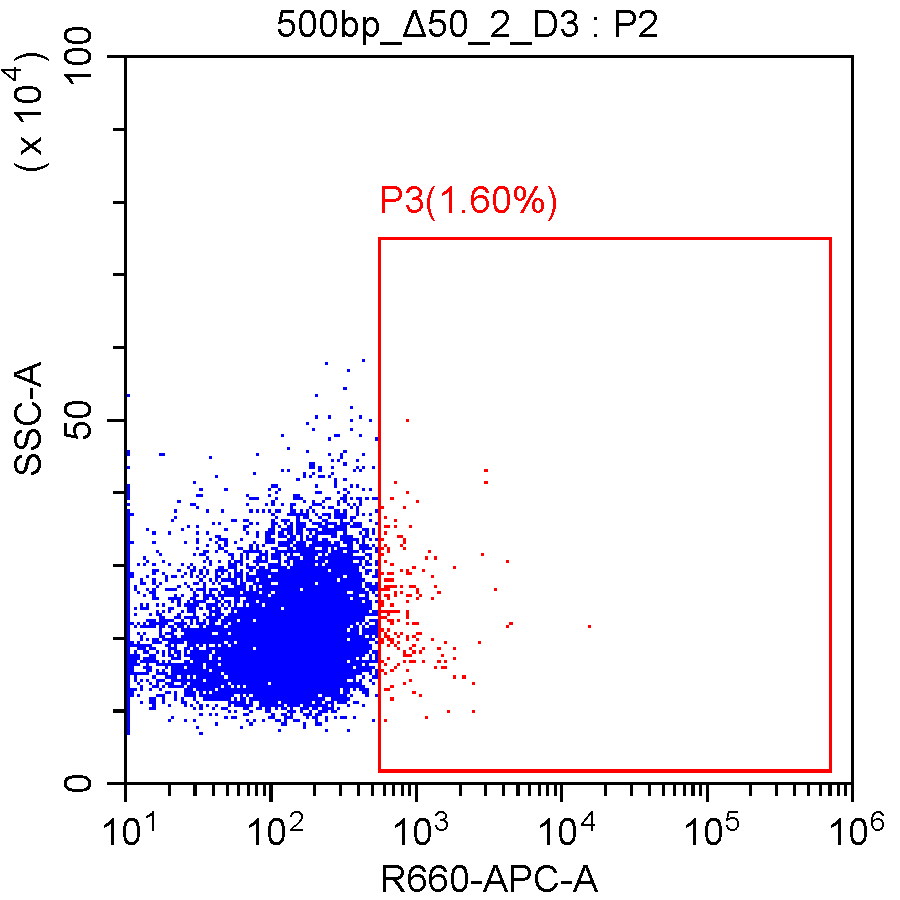

Supplement: Supplementary file 7 — Source data Fig. 5 [file 44318_2025_605_MOESM7_ESM.zip › SD figure 5_Revised/Fig. 5/5B/P500_Δ50 bp in NPC_D3/Replicate_2/Δ1_2.tiff]

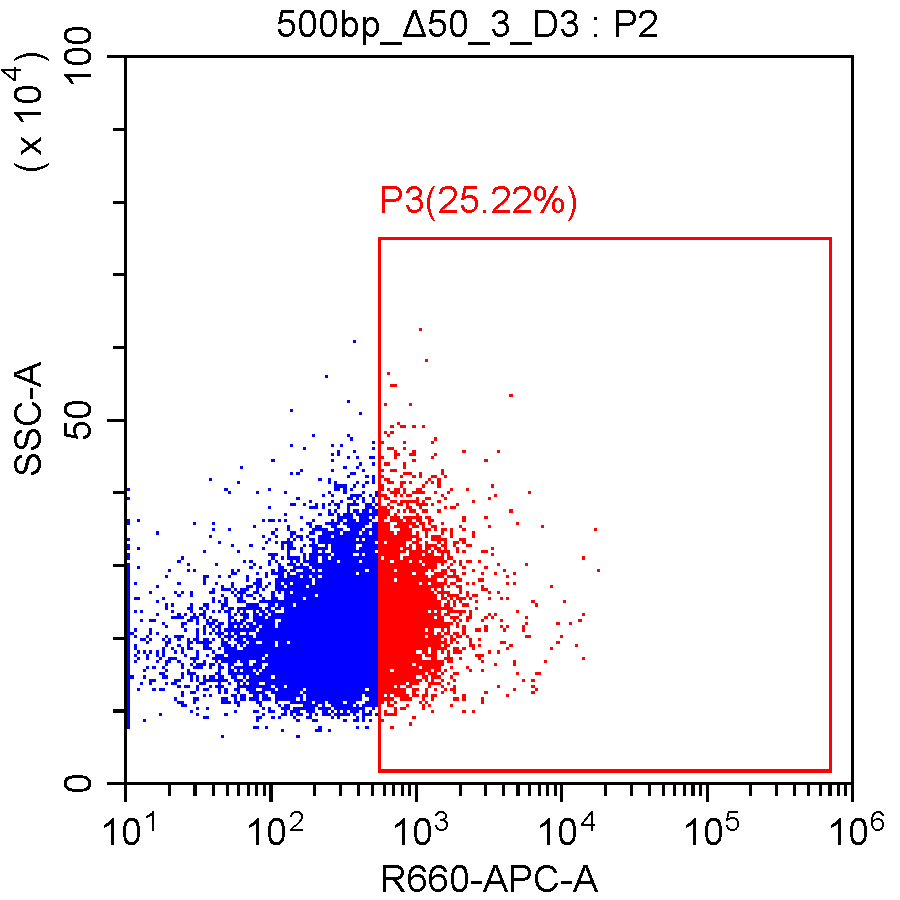

Supplement: Supplementary file 7 — Source data Fig. 5 [file 44318_2025_605_MOESM7_ESM.zip › SD figure 5_Revised/Fig. 5/5B/P500_Δ50 bp in NPC_D3/Replicate_2/Δ1_3.tiff]

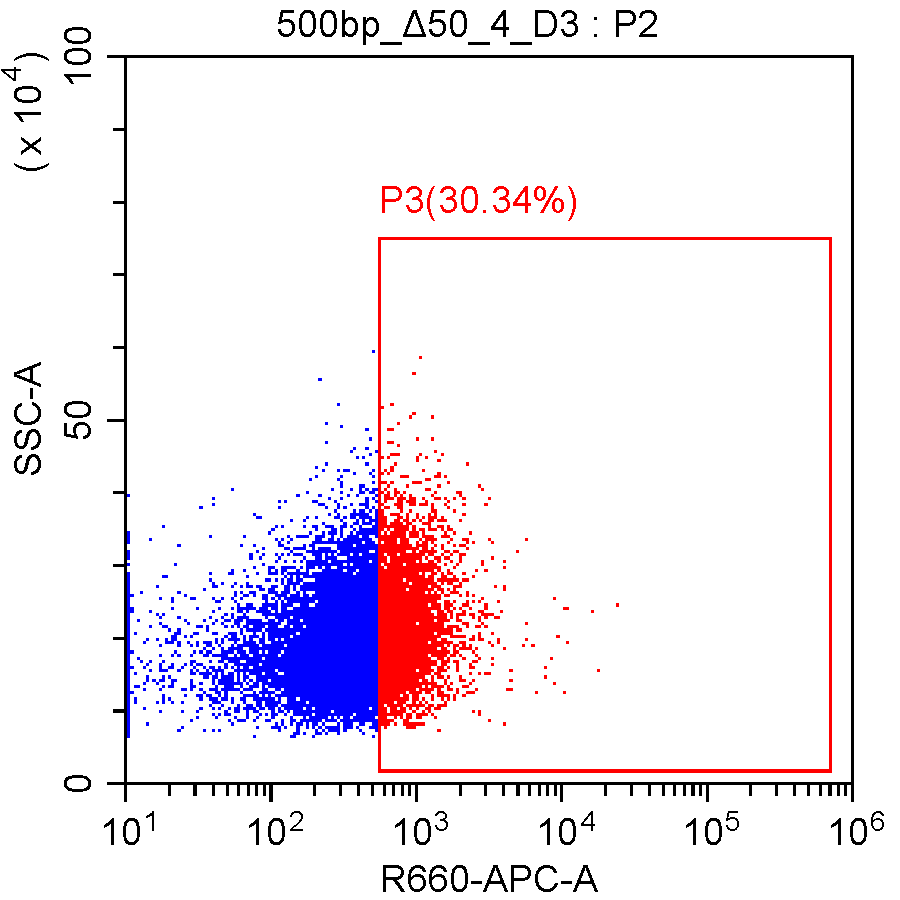

Supplement: Supplementary file 7 — Source data Fig. 5 [file 44318_2025_605_MOESM7_ESM.zip › SD figure 5_Revised/Fig. 5/5B/P500_Δ50 bp in NPC_D3/Replicate_2/Δ1_4.tiff]

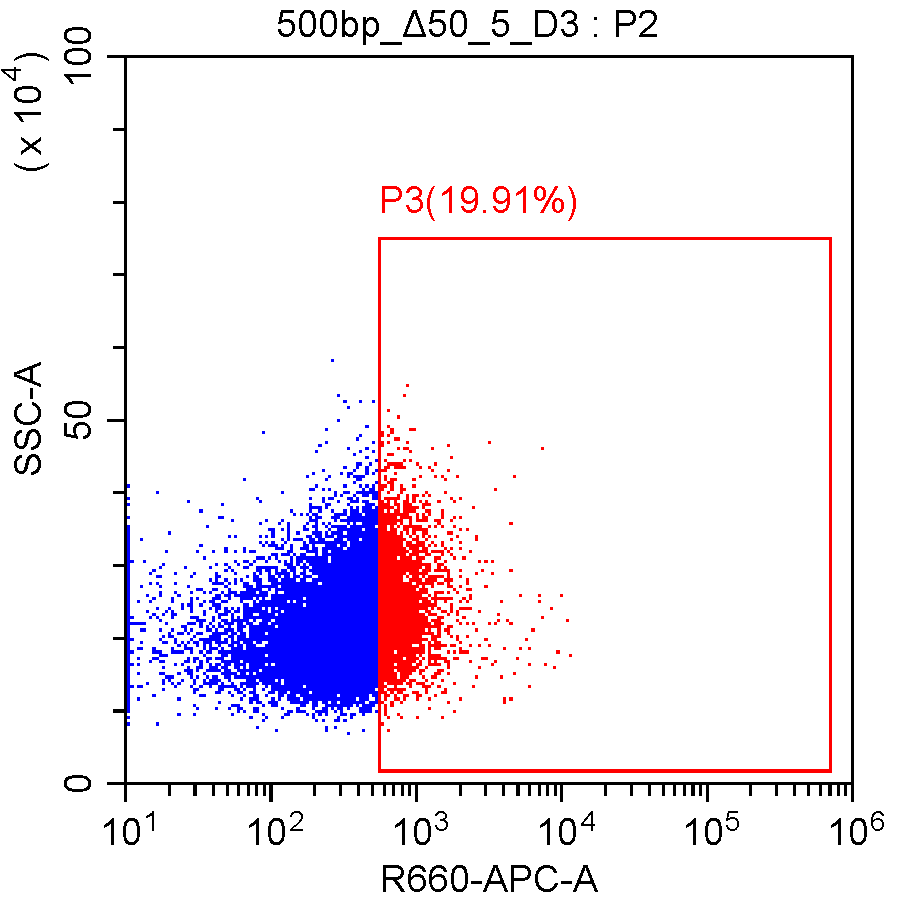

Supplement: Supplementary file 7 — Source data Fig. 5 [file 44318_2025_605_MOESM7_ESM.zip › SD figure 5_Revised/Fig. 5/5B/P500_Δ50 bp in NPC_D3/Replicate_2/Δ1_5.tiff]

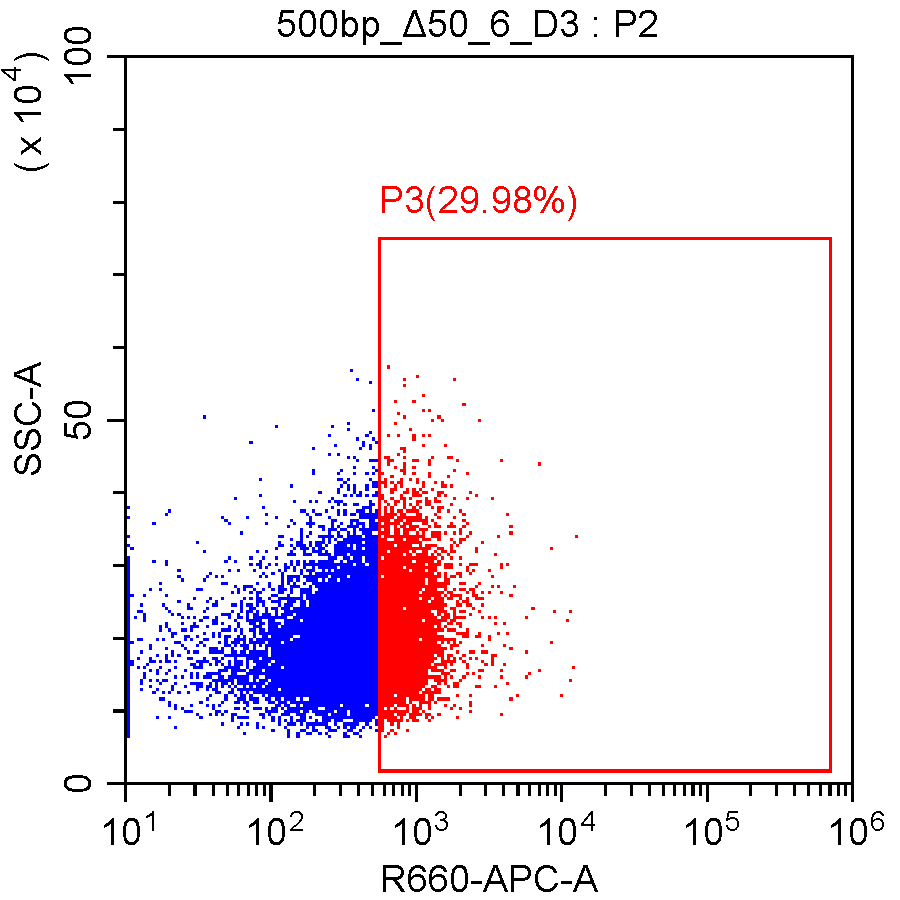

Supplement: Supplementary file 7 — Source data Fig. 5 [file 44318_2025_605_MOESM7_ESM.zip › SD figure 5_Revised/Fig. 5/5B/P500_Δ50 bp in NPC_D3/Replicate_2/Δ1_6.tiff]

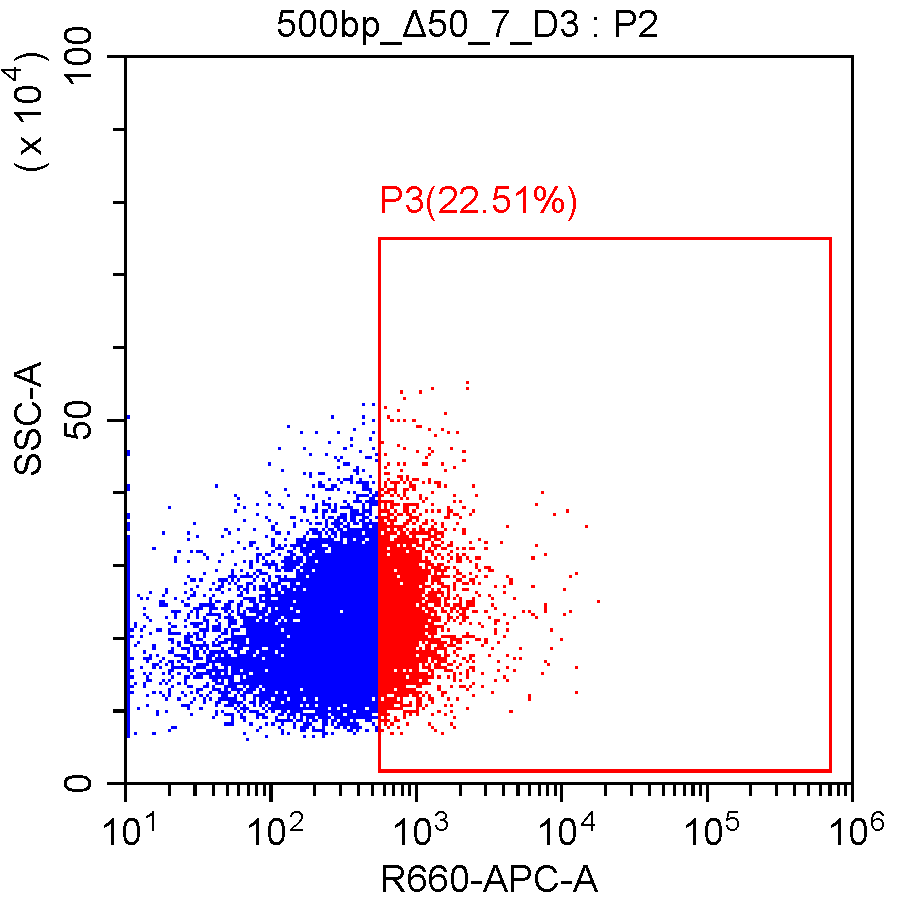

Supplement: Supplementary file 7 — Source data Fig. 5 [file 44318_2025_605_MOESM7_ESM.zip › SD figure 5_Revised/Fig. 5/5B/P500_Δ50 bp in NPC_D3/Replicate_2/Δ1_7.tiff]

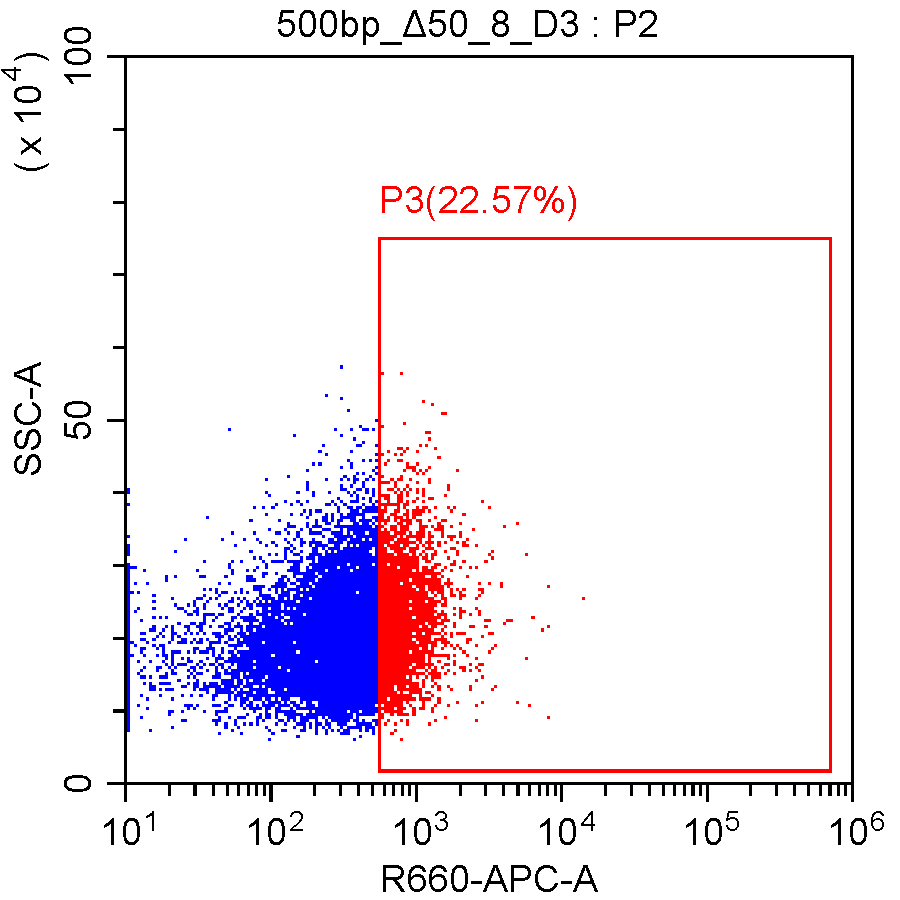

Supplement: Supplementary file 7 — Source data Fig. 5 [file 44318_2025_605_MOESM7_ESM.zip › SD figure 5_Revised/Fig. 5/5B/P500_Δ50 bp in NPC_D3/Replicate_2/Δ1_8.tiff]

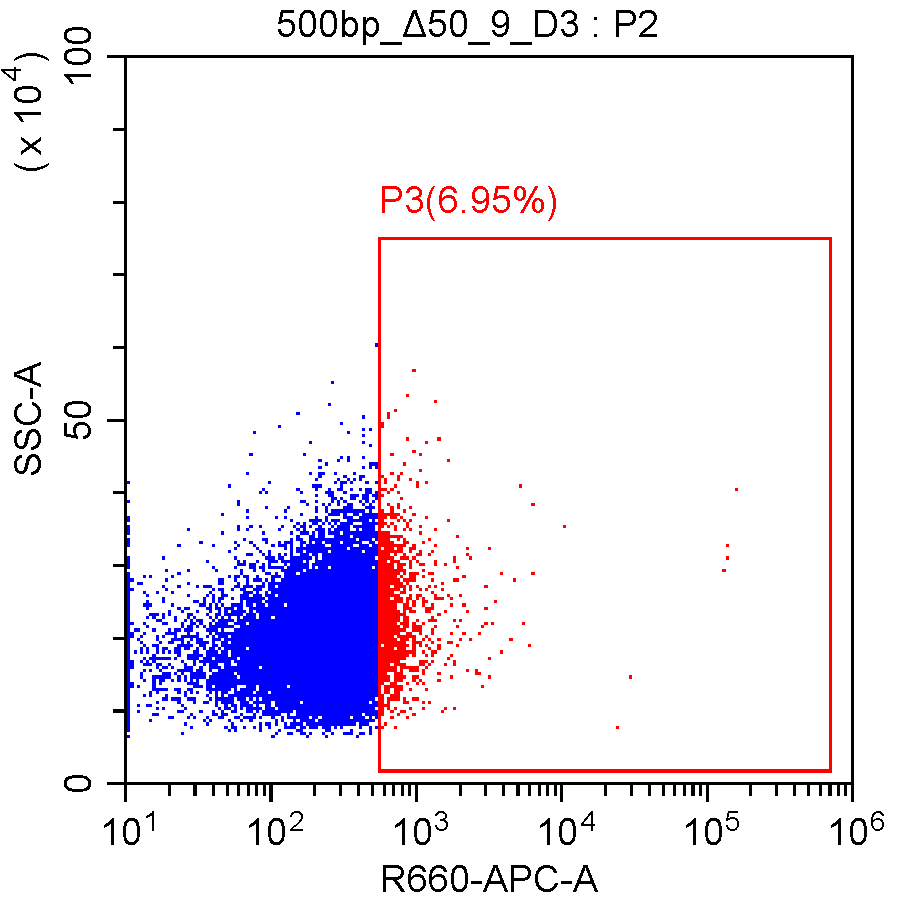

Supplement: Supplementary file 7 — Source data Fig. 5 [file 44318_2025_605_MOESM7_ESM.zip › SD figure 5_Revised/Fig. 5/5B/P500_Δ50 bp in NPC_D3/Replicate_2/Δ1_9.tiff]

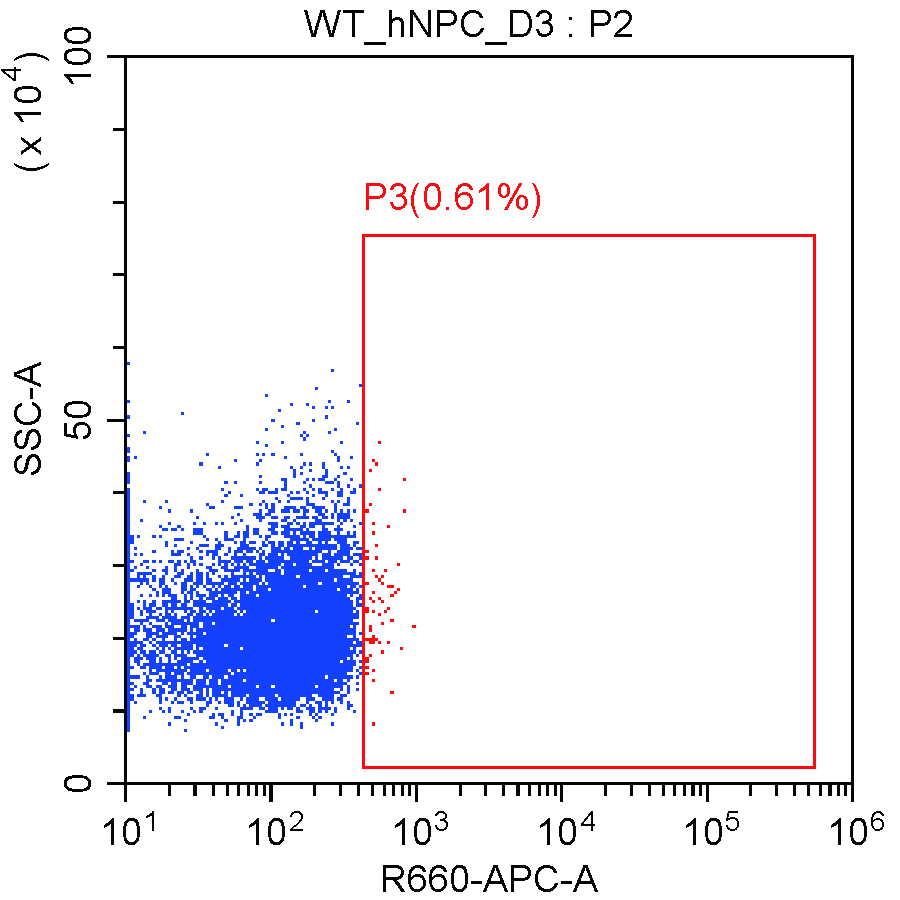

Supplement: Supplementary file 7 — Source data Fig. 5 [file 44318_2025_605_MOESM7_ESM.zip › SD figure 5_Revised/Fig. 5/5E/Replicate_1/Ctrl.tif]

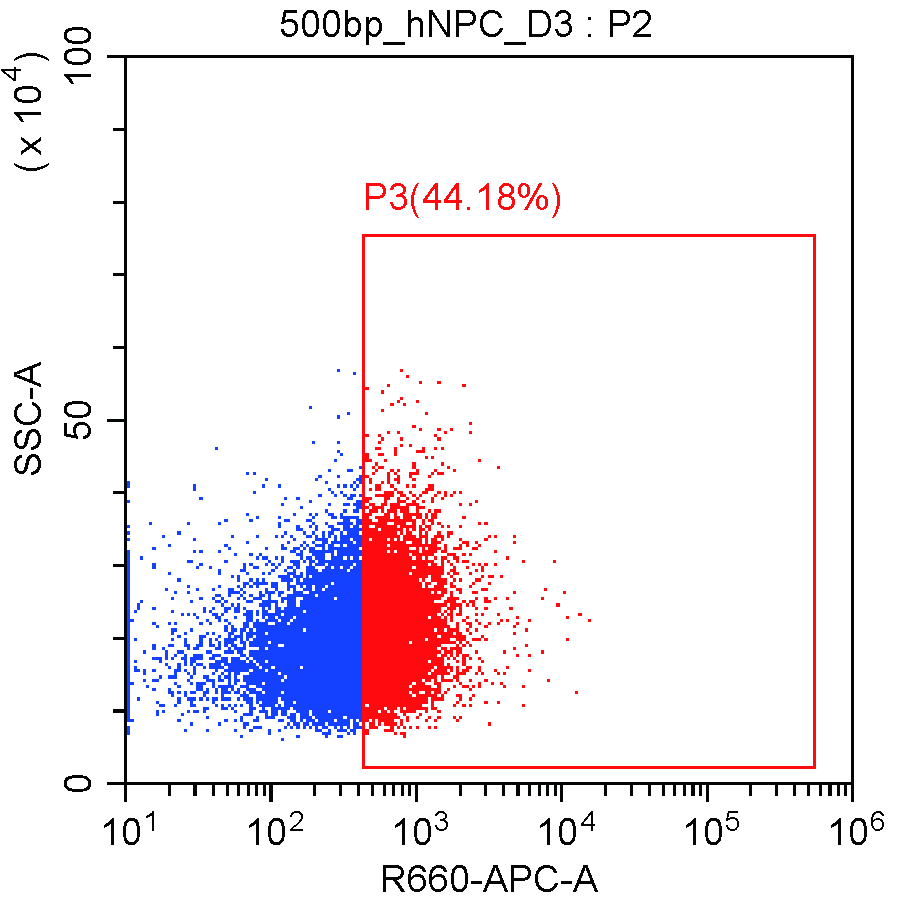

Supplement: Supplementary file 7 — Source data Fig. 5 [file 44318_2025_605_MOESM7_ESM.zip › SD figure 5_Revised/Fig. 5/5E/Replicate_1/P500_1.tif]

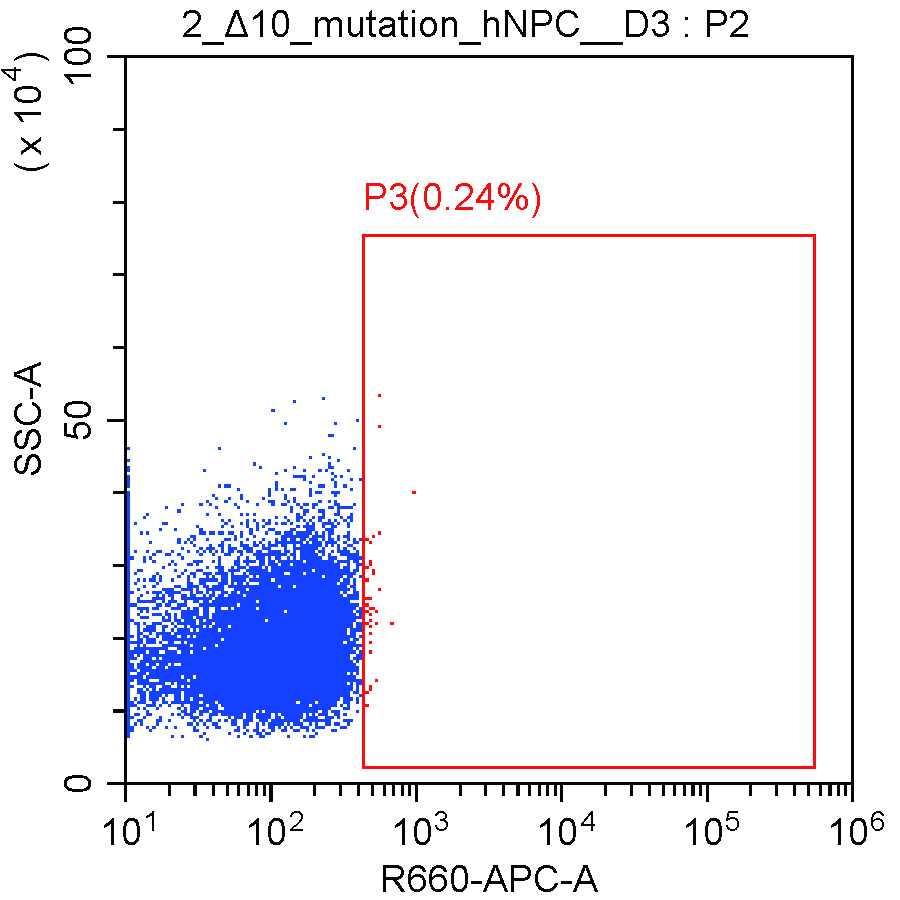

Supplement: Supplementary file 7 — Source data Fig. 5 [file 44318_2025_605_MOESM7_ESM.zip › SD figure 5_Revised/Fig. 5/5E/Replicate_1/Δ12_1.tif]

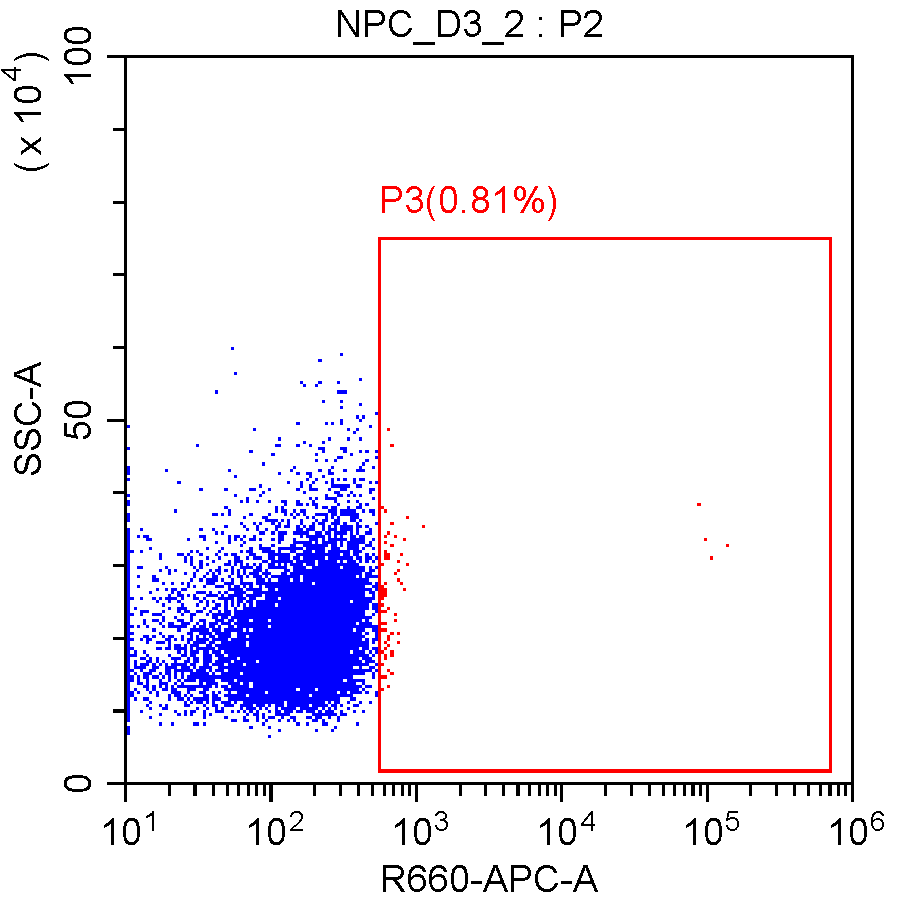

Supplement: Supplementary file 7 — Source data Fig. 5 [file 44318_2025_605_MOESM7_ESM.zip › SD figure 5_Revised/Fig. 5/5E/Replicate_2/Ctrl_2.bmp]

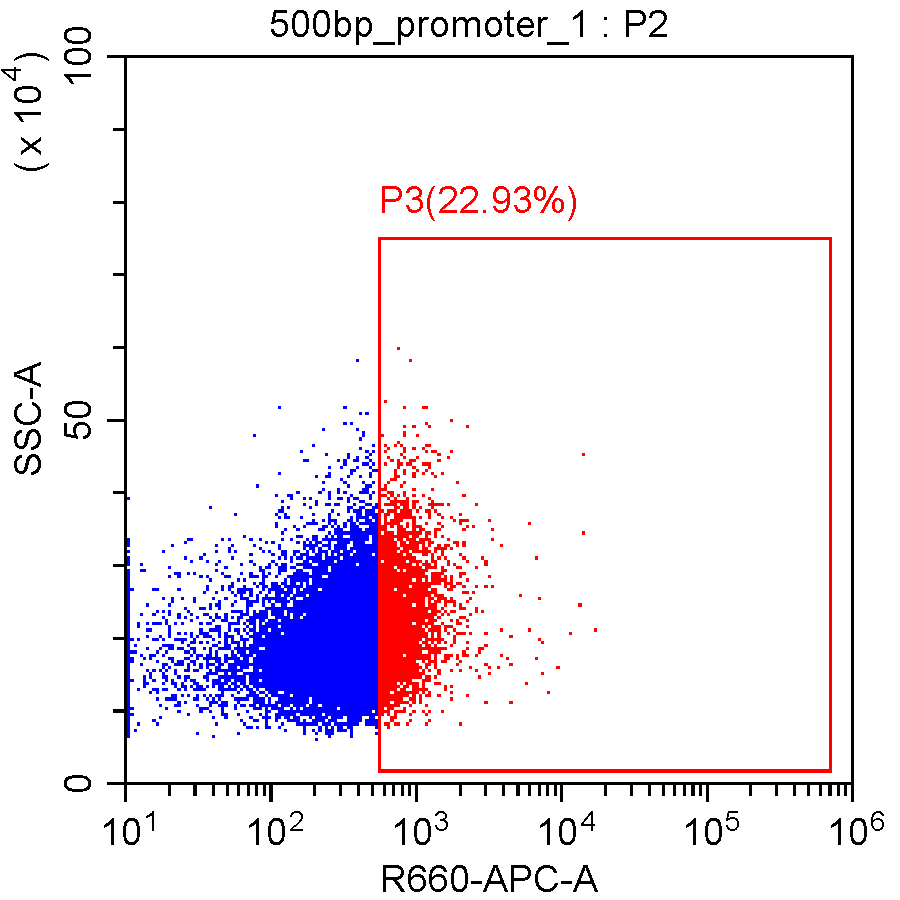

Supplement: Supplementary file 7 — Source data Fig. 5 [file 44318_2025_605_MOESM7_ESM.zip › SD figure 5_Revised/Fig. 5/5E/Replicate_2/P500_2.bmp]

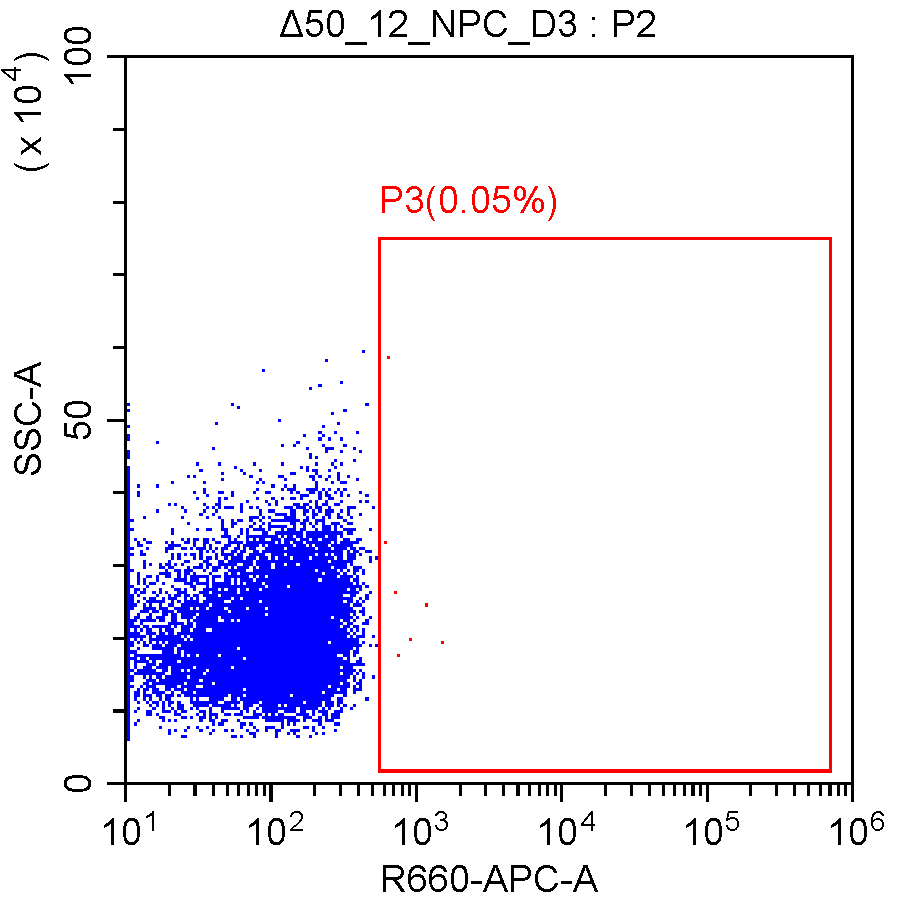

Supplement: Supplementary file 7 — Source data Fig. 5 [file 44318_2025_605_MOESM7_ESM.zip › SD figure 5_Revised/Fig. 5/5E/Replicate_2/Δ12_2.bmp]

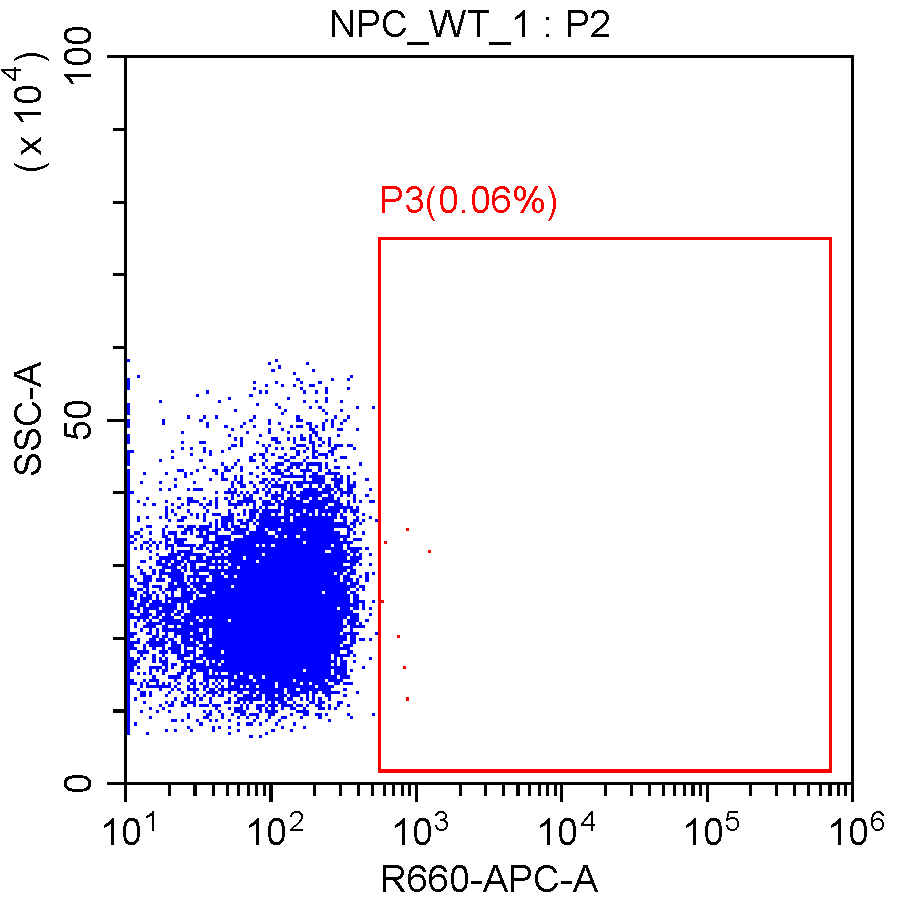

Supplement: Supplementary file 7 — Source data Fig. 5 [file 44318_2025_605_MOESM7_ESM.zip › SD figure 5_Revised/Fig. 5/5E/Replicate_3/Ctrl_3.tiff]

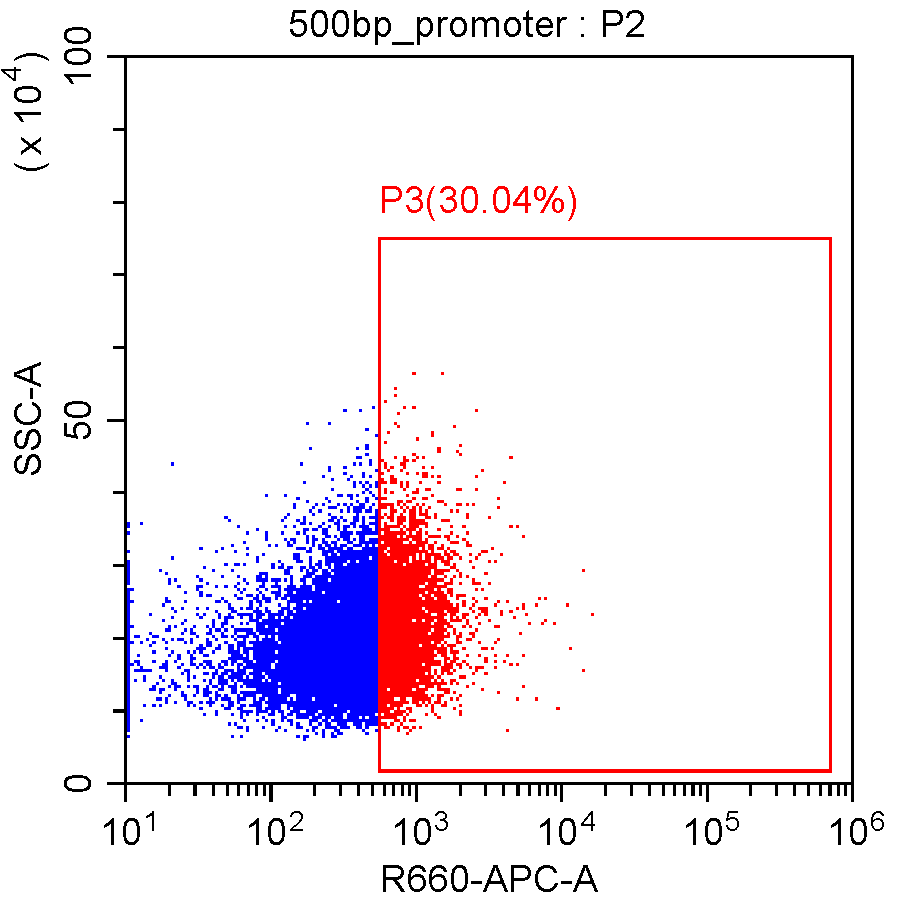

Supplement: Supplementary file 7 — Source data Fig. 5 [file 44318_2025_605_MOESM7_ESM.zip › SD figure 5_Revised/Fig. 5/5E/Replicate_3/P500_3.tiff]

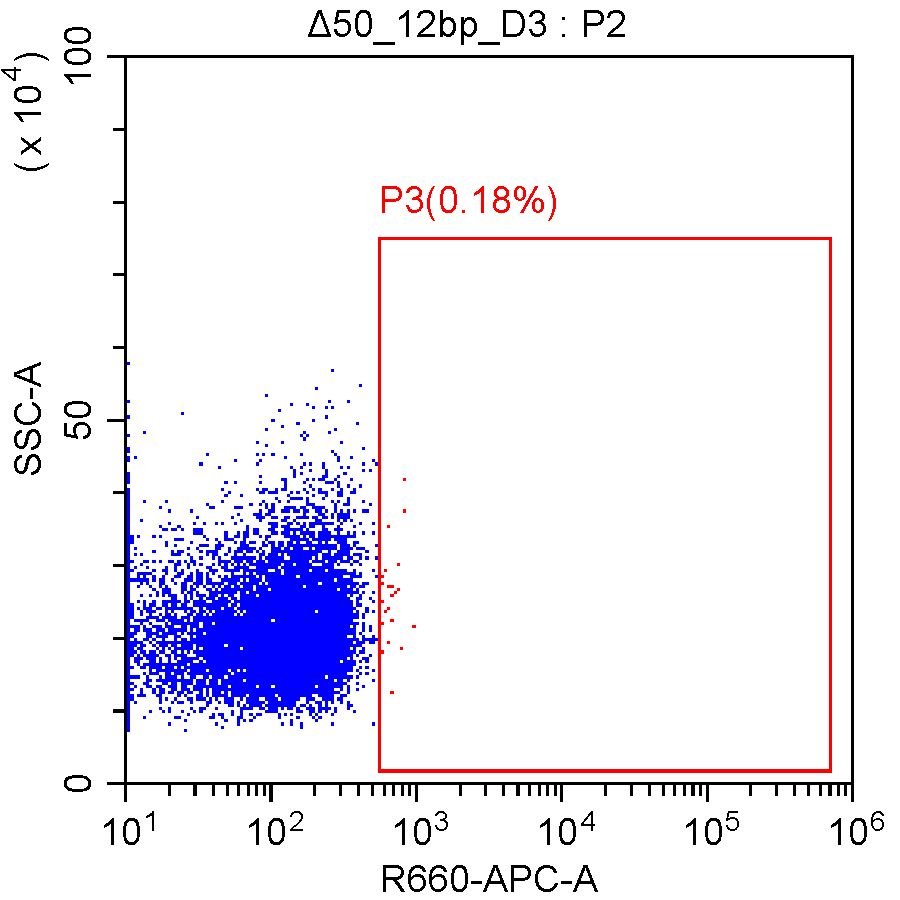

Supplement: Supplementary file 7 — Source data Fig. 5 [file 44318_2025_605_MOESM7_ESM.zip › SD figure 5_Revised/Fig. 5/5E/Replicate_3/Δ12_3.tiff]

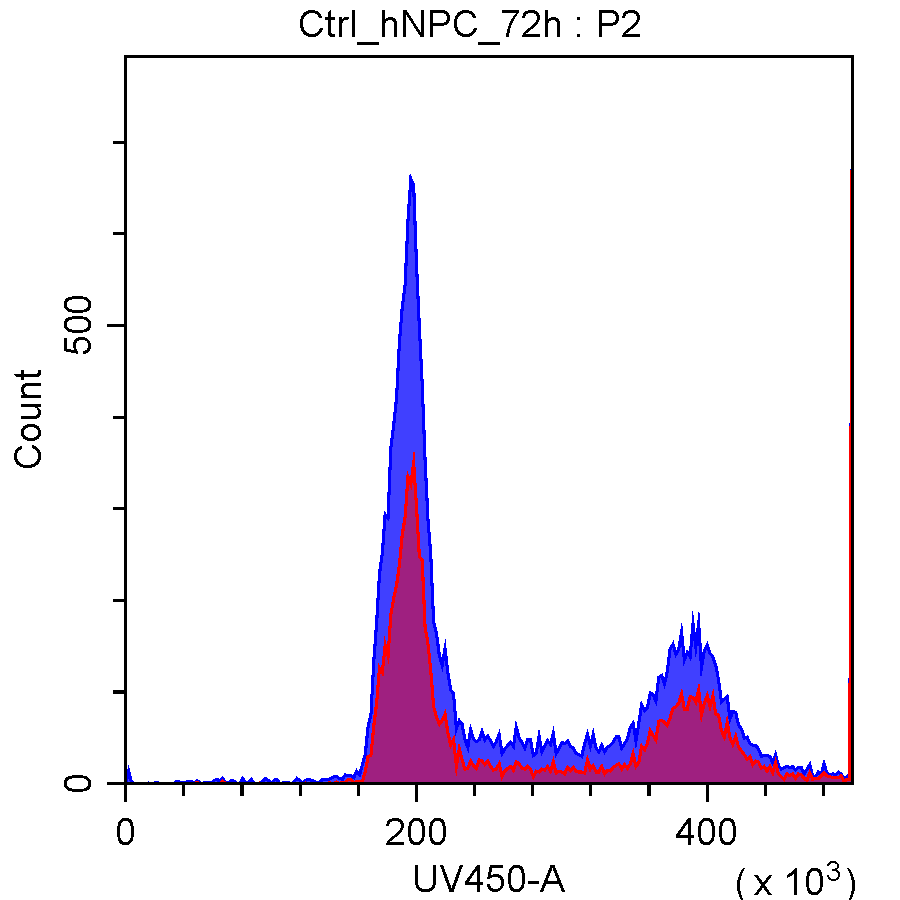

Supplement: Supplementary file 8 — Source data Fig. 6 [file 44318_2025_605_MOESM8_ESM.zip › Fig. 6/6B/Replicate_1/DAPI/Ctrl_NPC_D3.tif]

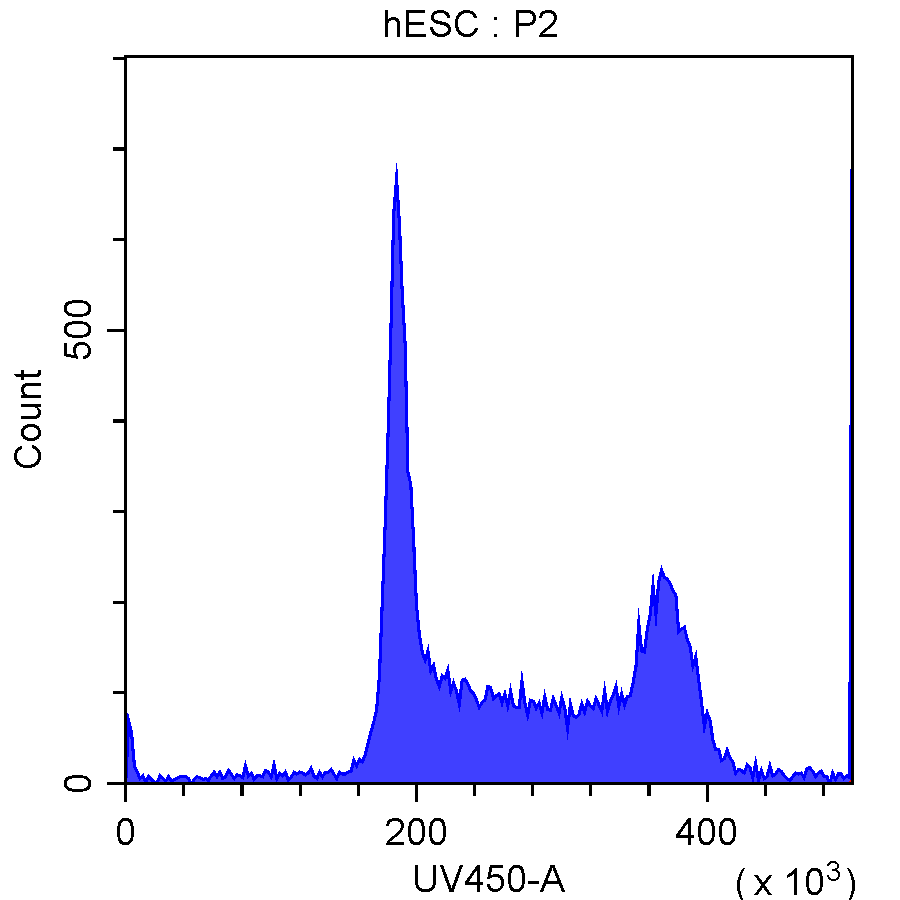

Supplement: Supplementary file 8 — Source data Fig. 6 [file 44318_2025_605_MOESM8_ESM.zip › Fig. 6/6B/Replicate_1/DAPI/hESC_Plot1.bmp]

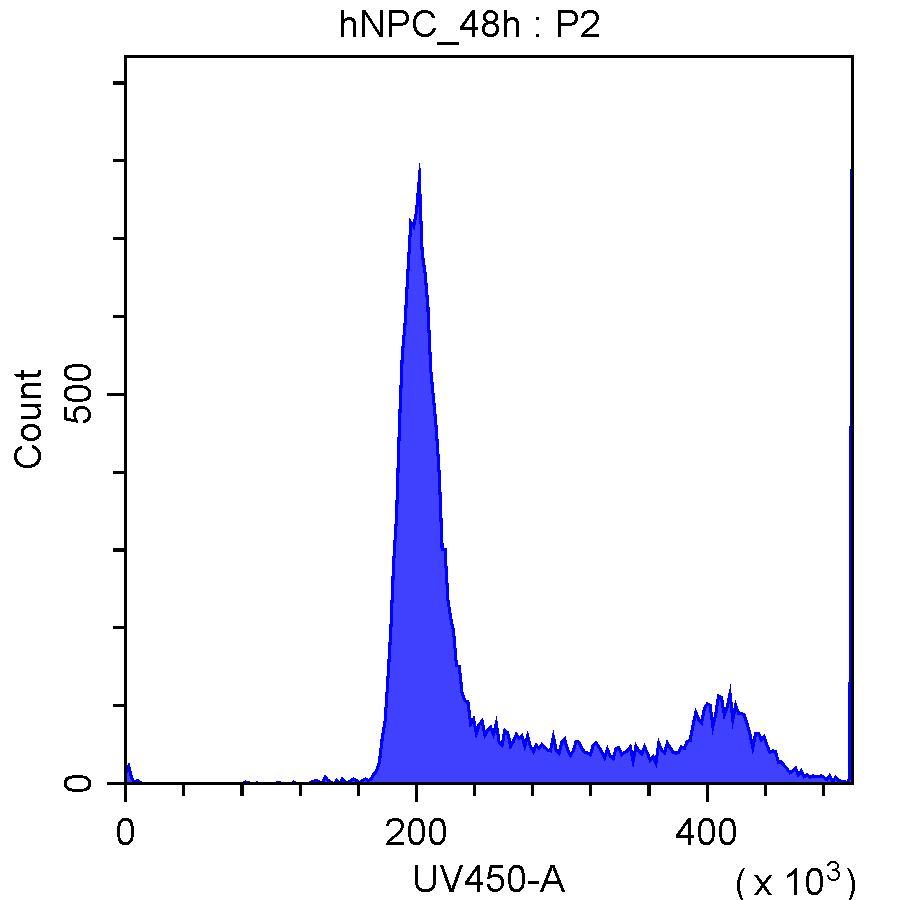

Supplement: Supplementary file 8 — Source data Fig. 6 [file 44318_2025_605_MOESM8_ESM.zip › Fig. 6/6B/Replicate_1/DAPI/hNPC_48h_Plot1.bmp]

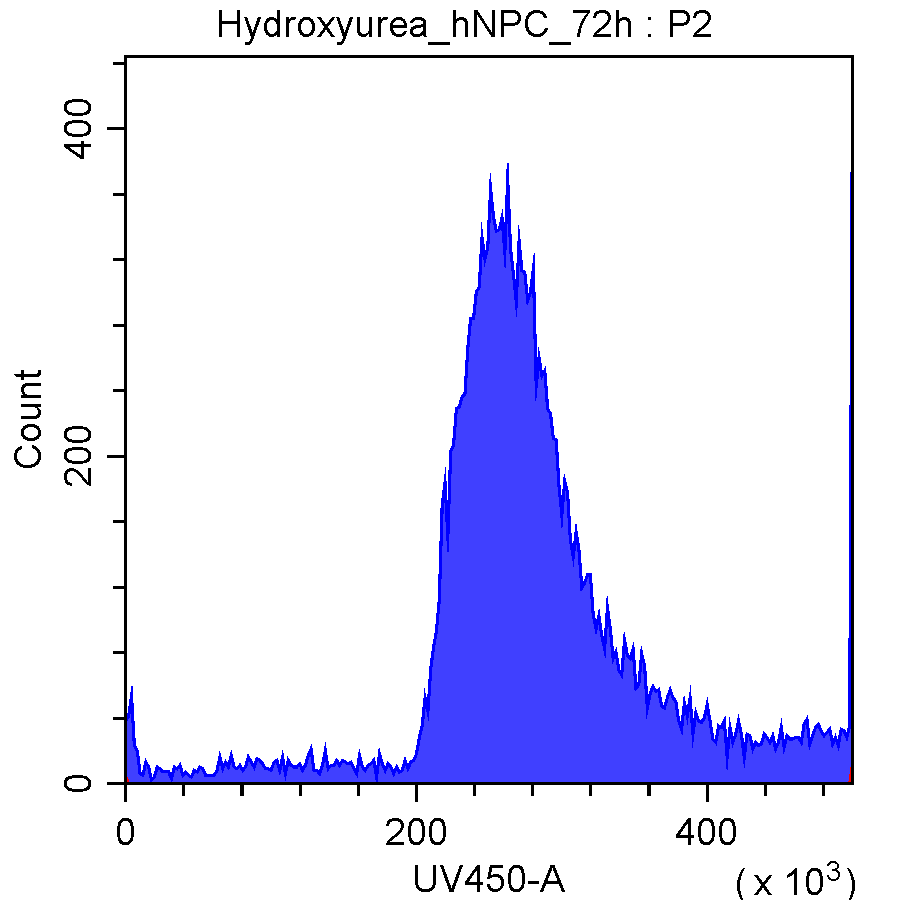

Supplement: Supplementary file 8 — Source data Fig. 6 [file 44318_2025_605_MOESM8_ESM.zip › Fig. 6/6B/Replicate_1/DAPI/Hydroxyurea_hNPC_72h_Plot1.bmp]

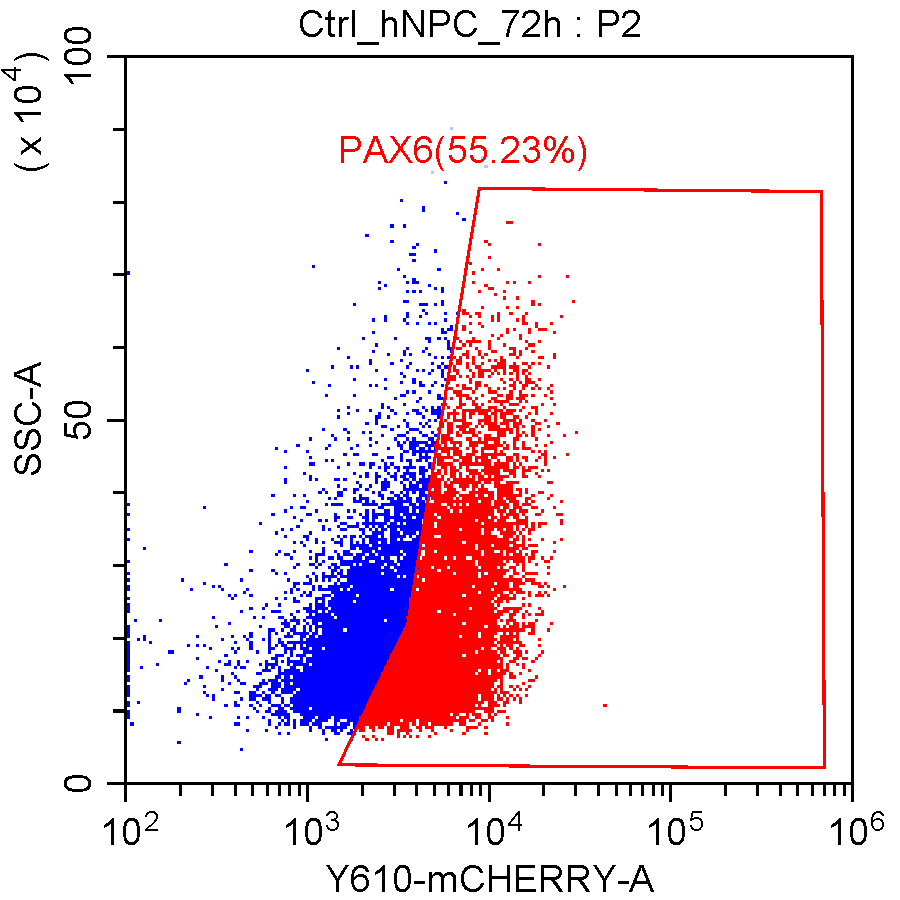

Supplement: Supplementary file 8 — Source data Fig. 6 [file 44318_2025_605_MOESM8_ESM.zip › Fig. 6/6B/Replicate_1/PAX6/Ctrl_NPC_72h.tif]

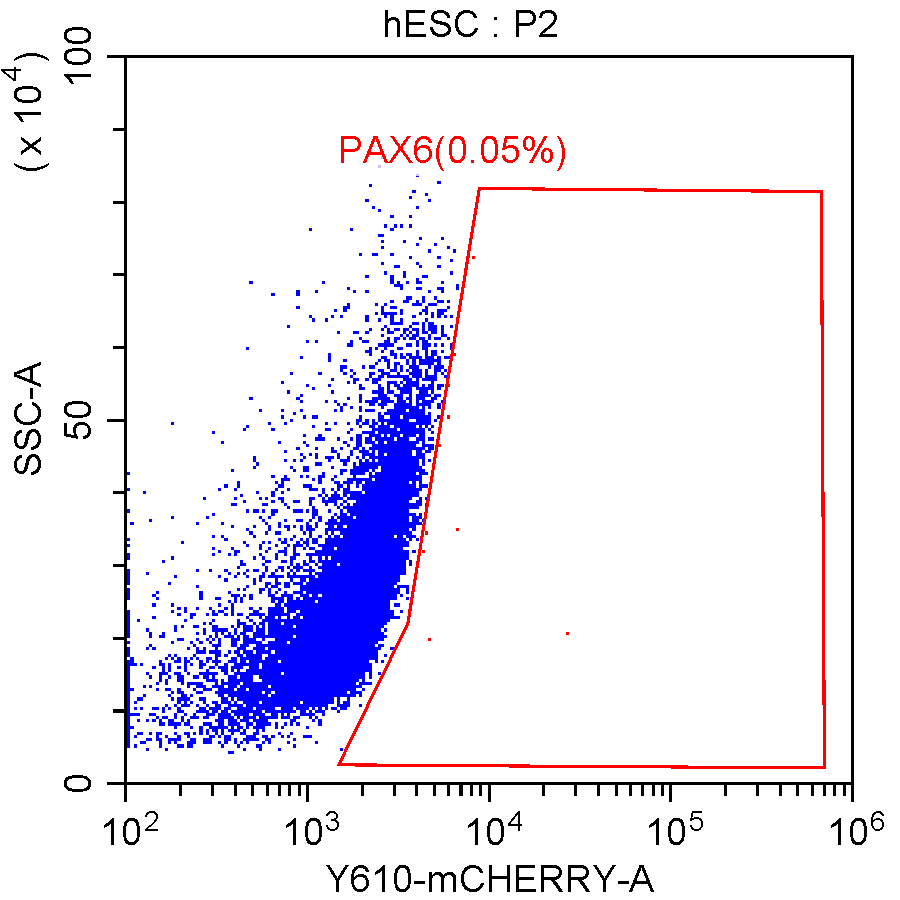

Supplement: Supplementary file 8 — Source data Fig. 6 [file 44318_2025_605_MOESM8_ESM.zip › Fig. 6/6B/Replicate_1/PAX6/ESC.tif]

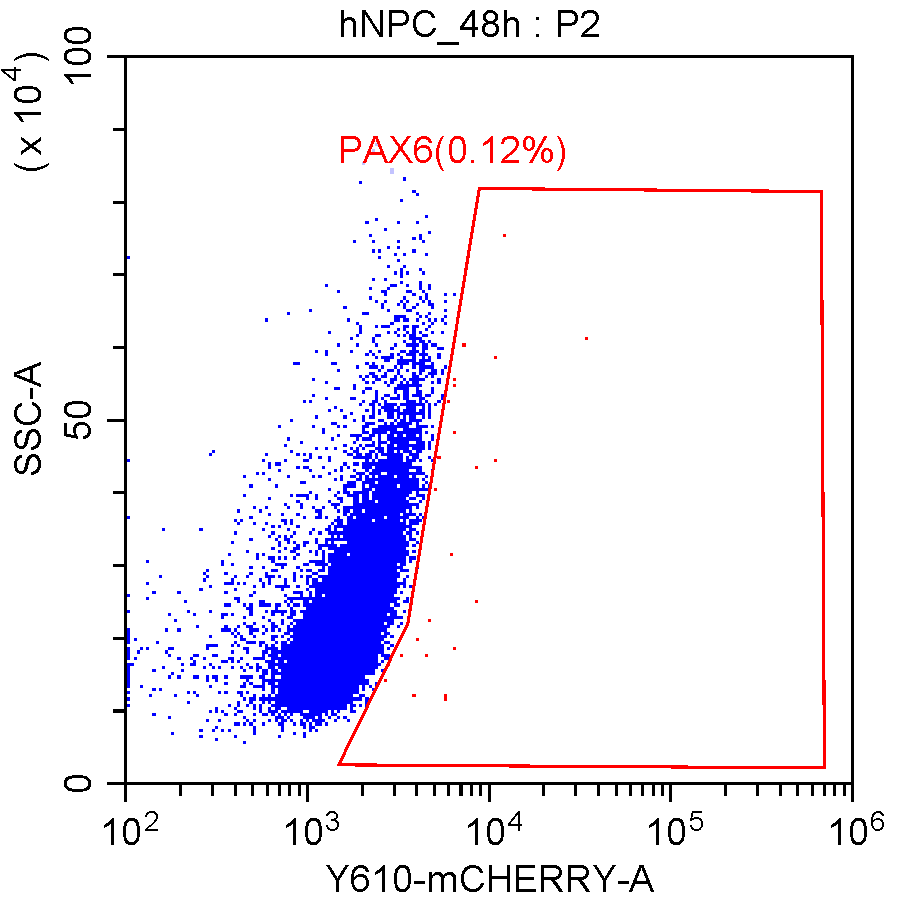

Supplement: Supplementary file 8 — Source data Fig. 6 [file 44318_2025_605_MOESM8_ESM.zip › Fig. 6/6B/Replicate_1/PAX6/NPC_D2.tif]

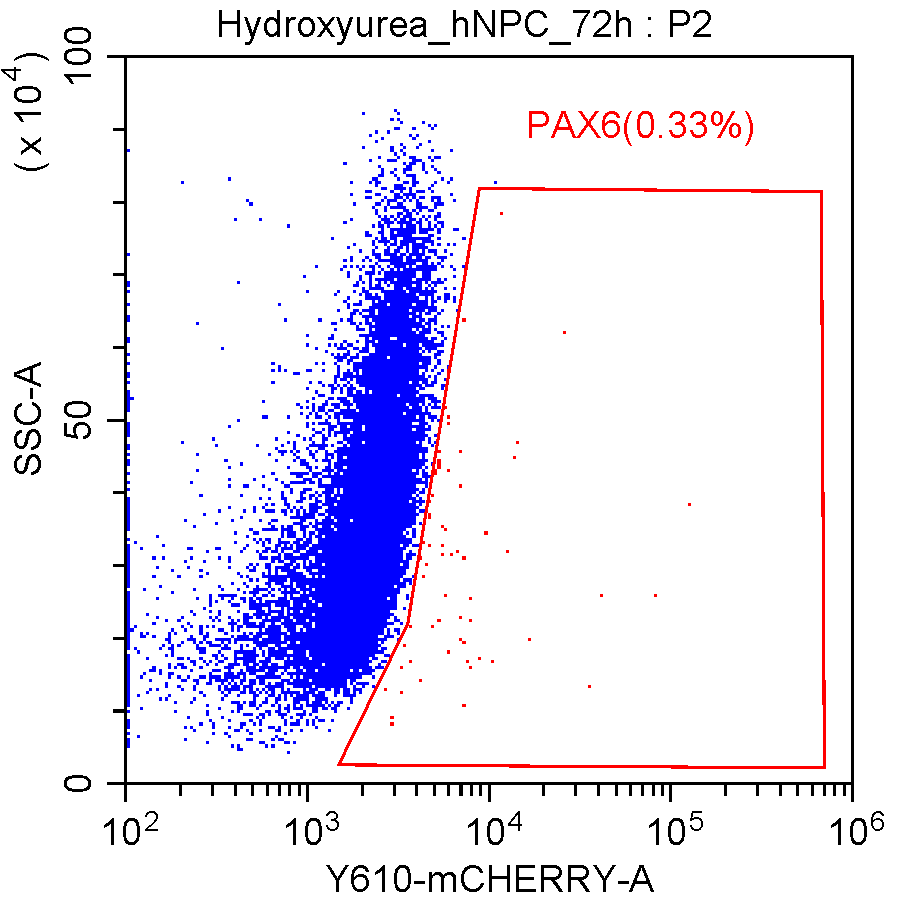

Supplement: Supplementary file 8 — Source data Fig. 6 [file 44318_2025_605_MOESM8_ESM.zip › Fig. 6/6B/Replicate_1/PAX6/NPC_D3_HU 24h.tif]

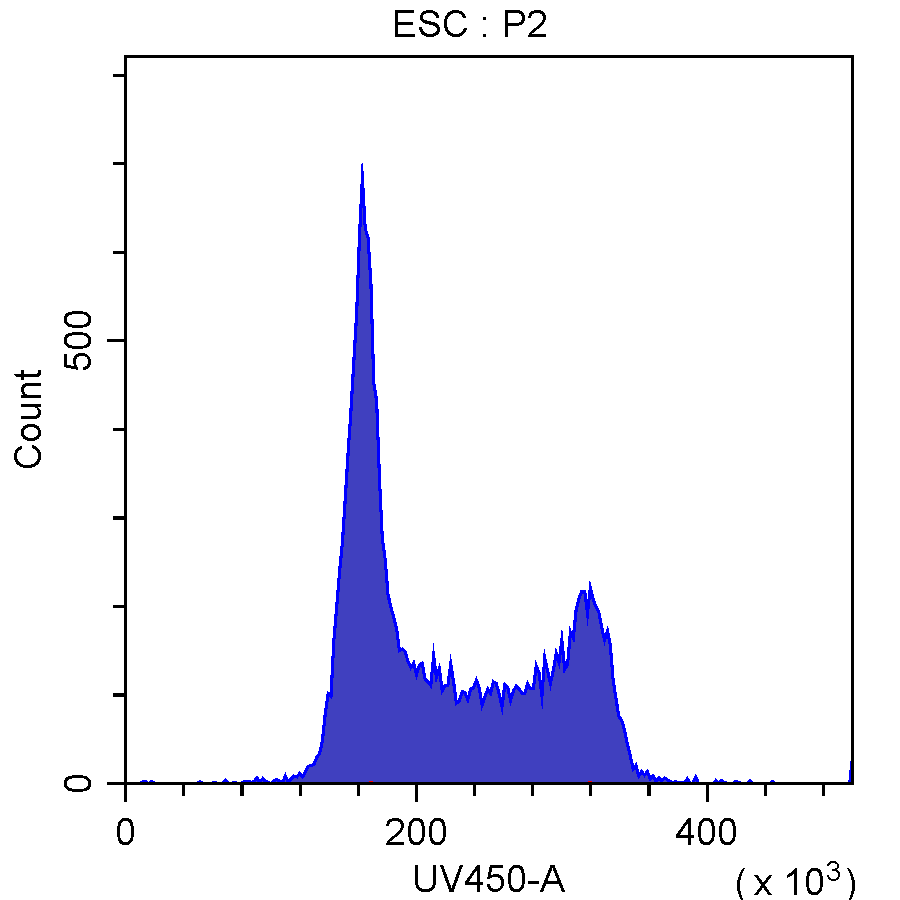

Supplement: Supplementary file 8 — Source data Fig. 6 [file 44318_2025_605_MOESM8_ESM.zip › Fig. 6/6B/Replicate_2/DAPI/ESC.tif]

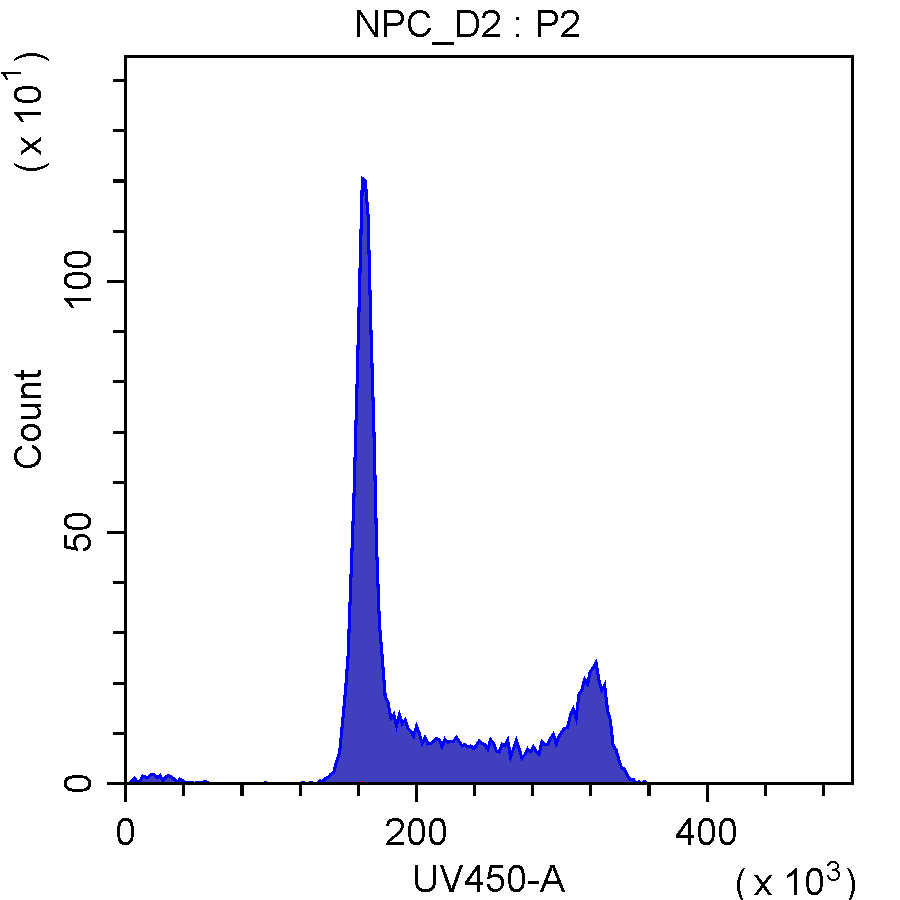

Supplement: Supplementary file 8 — Source data Fig. 6 [file 44318_2025_605_MOESM8_ESM.zip › Fig. 6/6B/Replicate_2/DAPI/NPC_D2.tif]

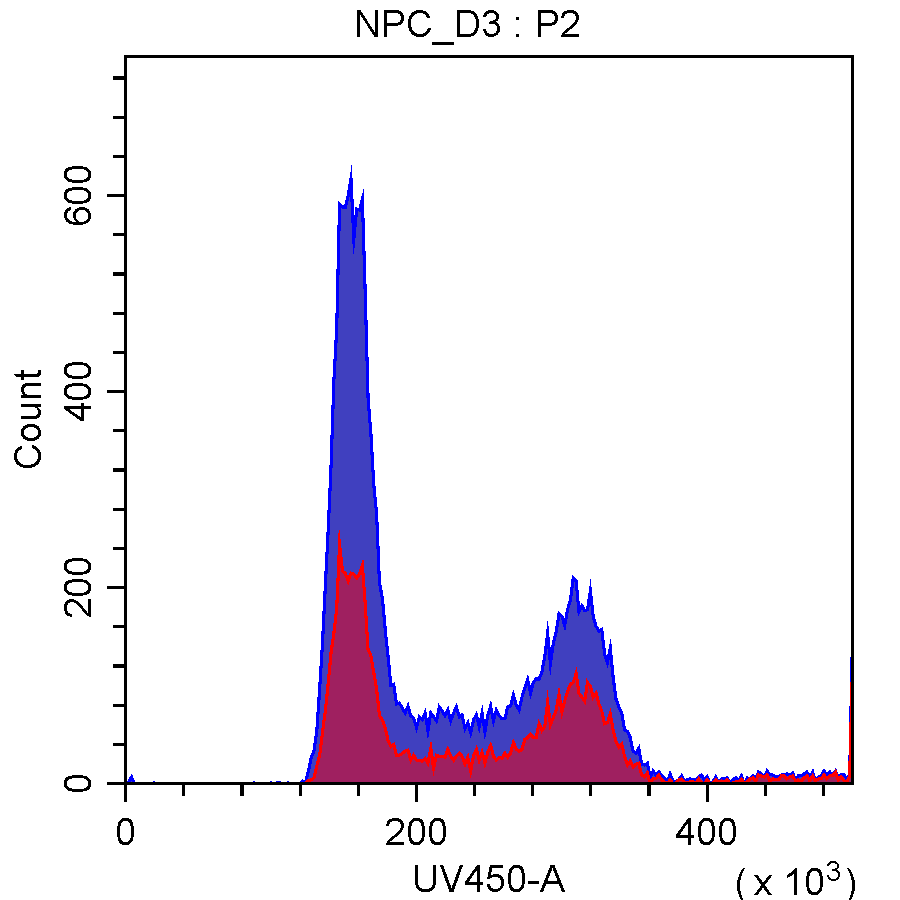

Supplement: Supplementary file 8 — Source data Fig. 6 [file 44318_2025_605_MOESM8_ESM.zip › Fig. 6/6B/Replicate_2/DAPI/NPC_D3_Ctrl.tif]

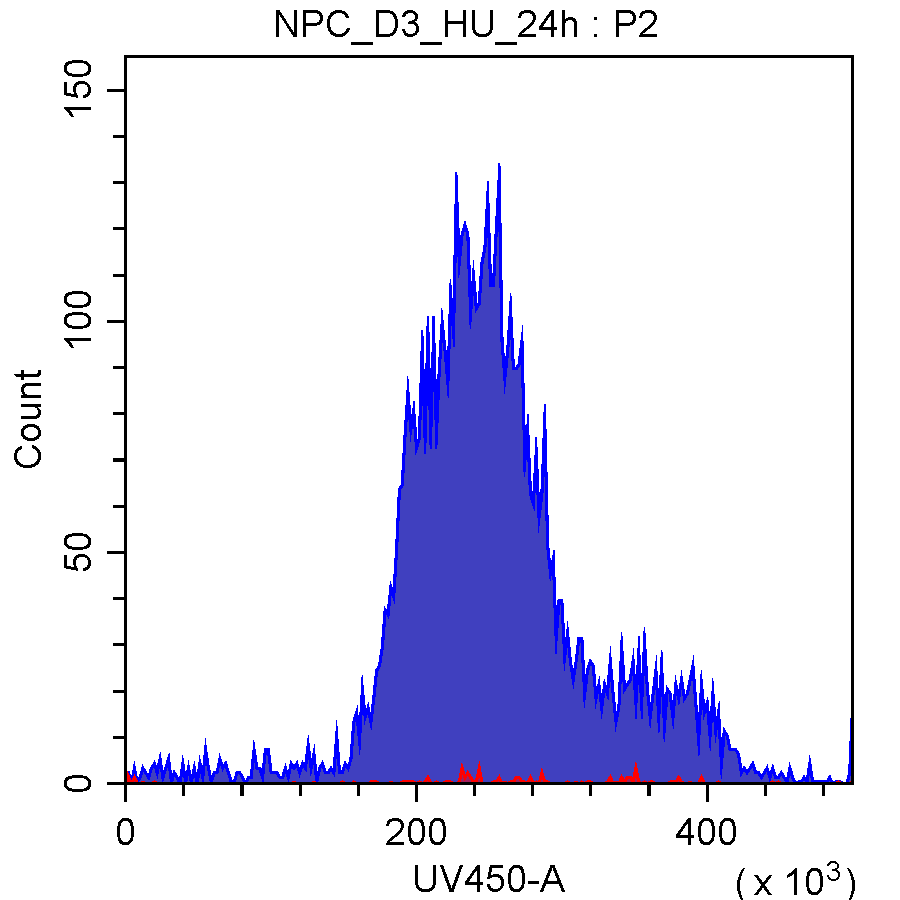

Supplement: Supplementary file 8 — Source data Fig. 6 [file 44318_2025_605_MOESM8_ESM.zip › Fig. 6/6B/Replicate_2/DAPI/NPC_D3_HU_24h.tif]

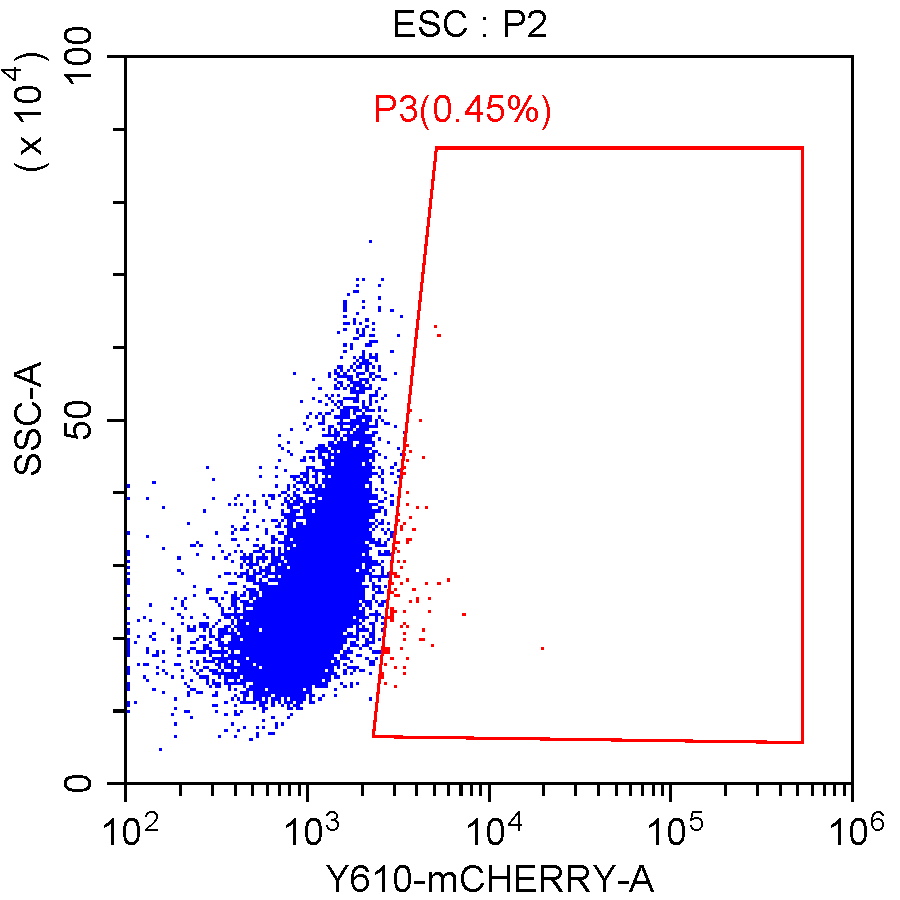

Supplement: Supplementary file 8 — Source data Fig. 6 [file 44318_2025_605_MOESM8_ESM.zip › Fig. 6/6B/Replicate_2/PAX6/ESC.tif]

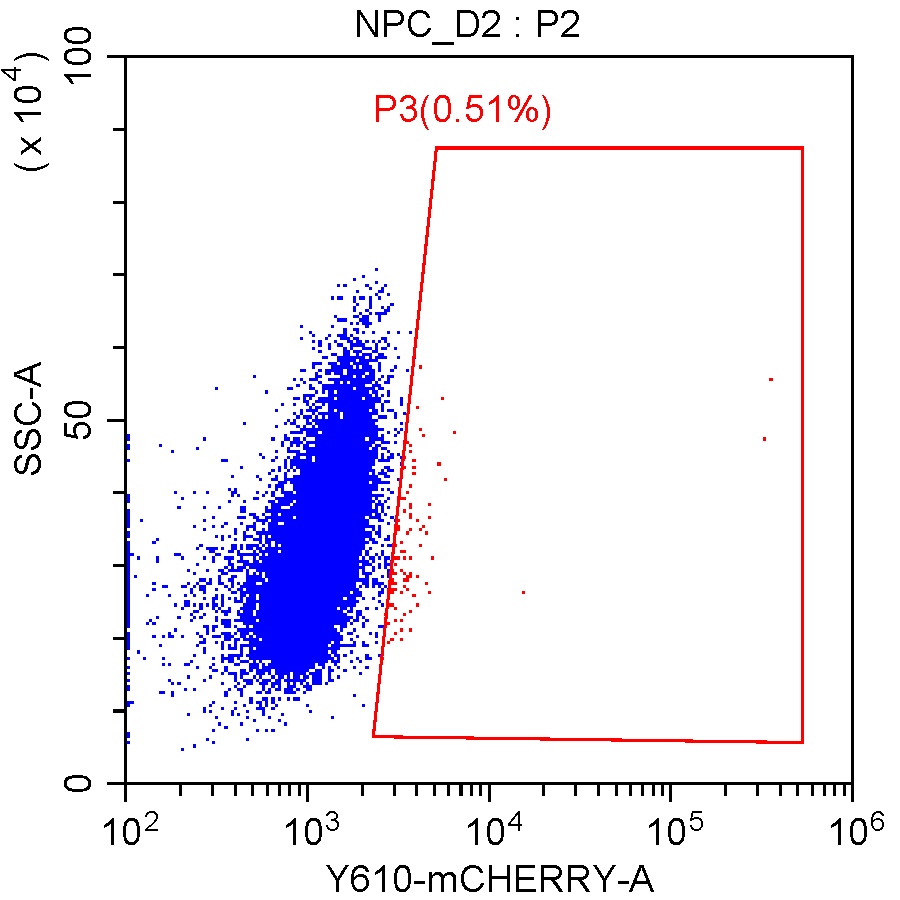

Supplement: Supplementary file 8 — Source data Fig. 6 [file 44318_2025_605_MOESM8_ESM.zip › Fig. 6/6B/Replicate_2/PAX6/NPC_D2.tif]

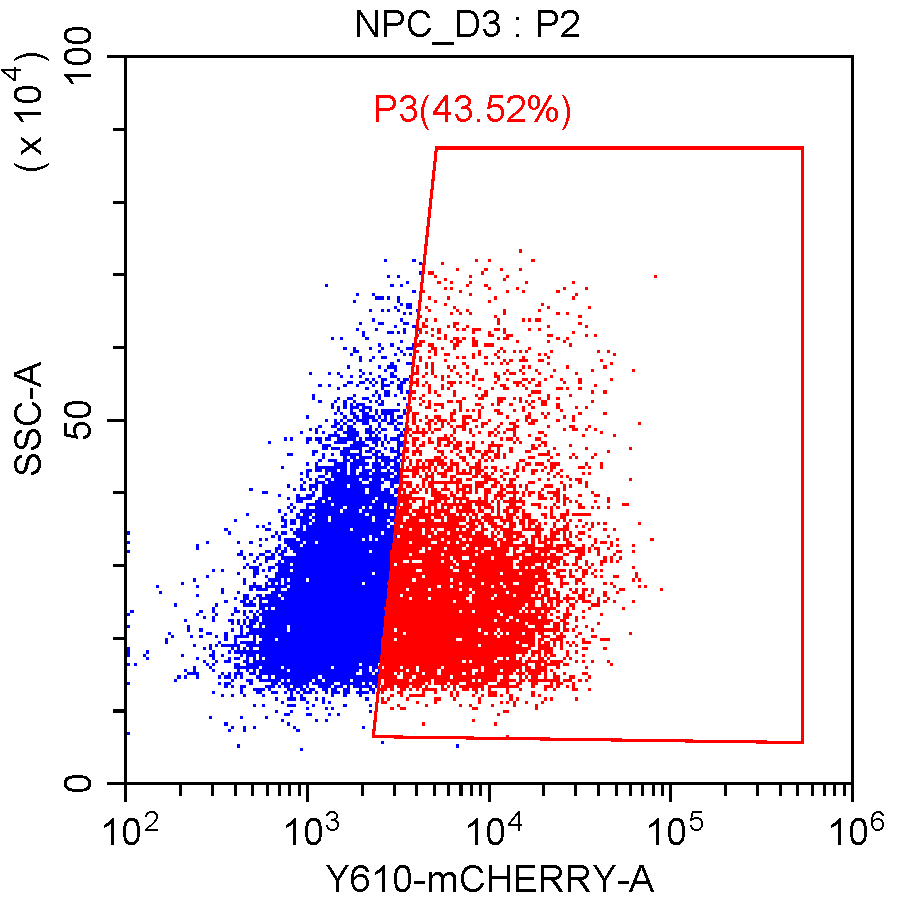

Supplement: Supplementary file 8 — Source data Fig. 6 [file 44318_2025_605_MOESM8_ESM.zip › Fig. 6/6B/Replicate_2/PAX6/NPC_D3_Ctrl.tif]

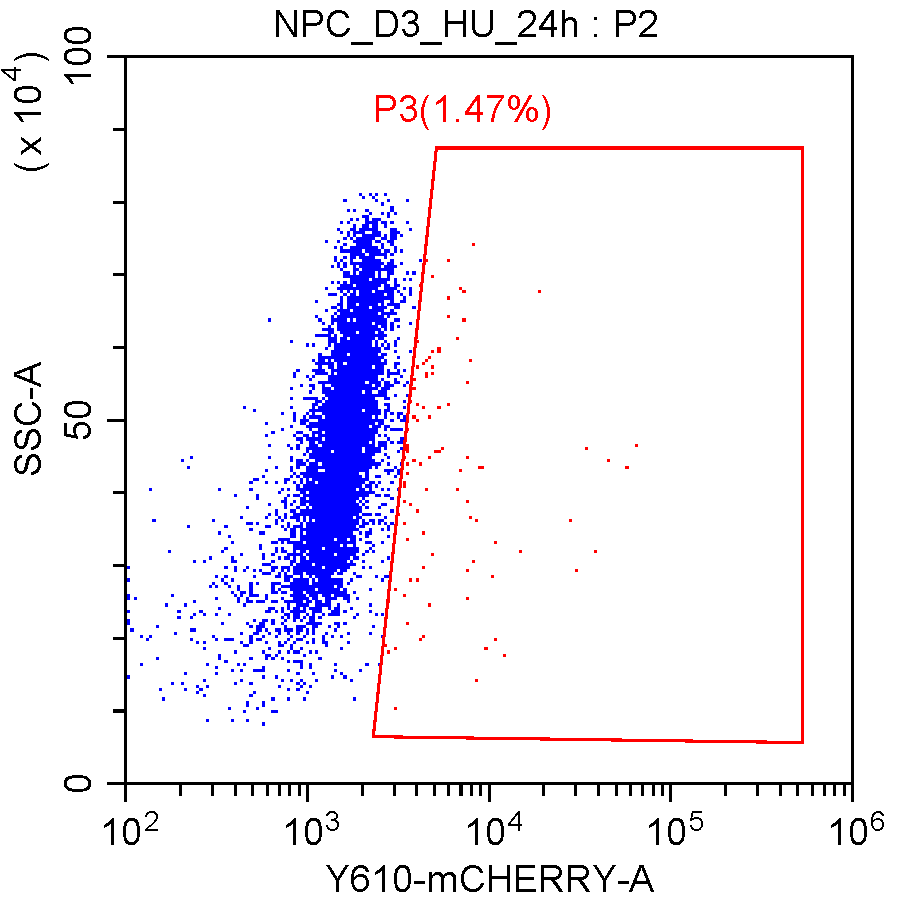

Supplement: Supplementary file 8 — Source data Fig. 6 [file 44318_2025_605_MOESM8_ESM.zip › Fig. 6/6B/Replicate_2/PAX6/NPC_D3_HU_24h.tif]

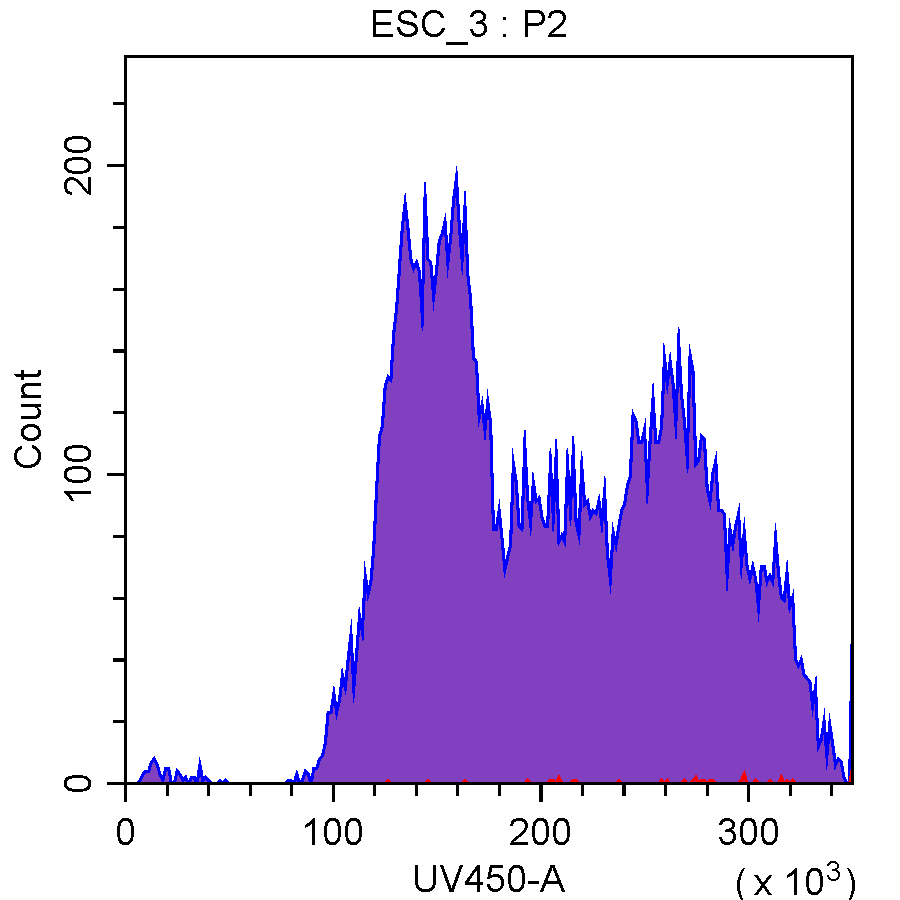

Supplement: Supplementary file 8 — Source data Fig. 6 [file 44318_2025_605_MOESM8_ESM.zip › Fig. 6/6B/Replicate_3/DAPI/ESC.tif]

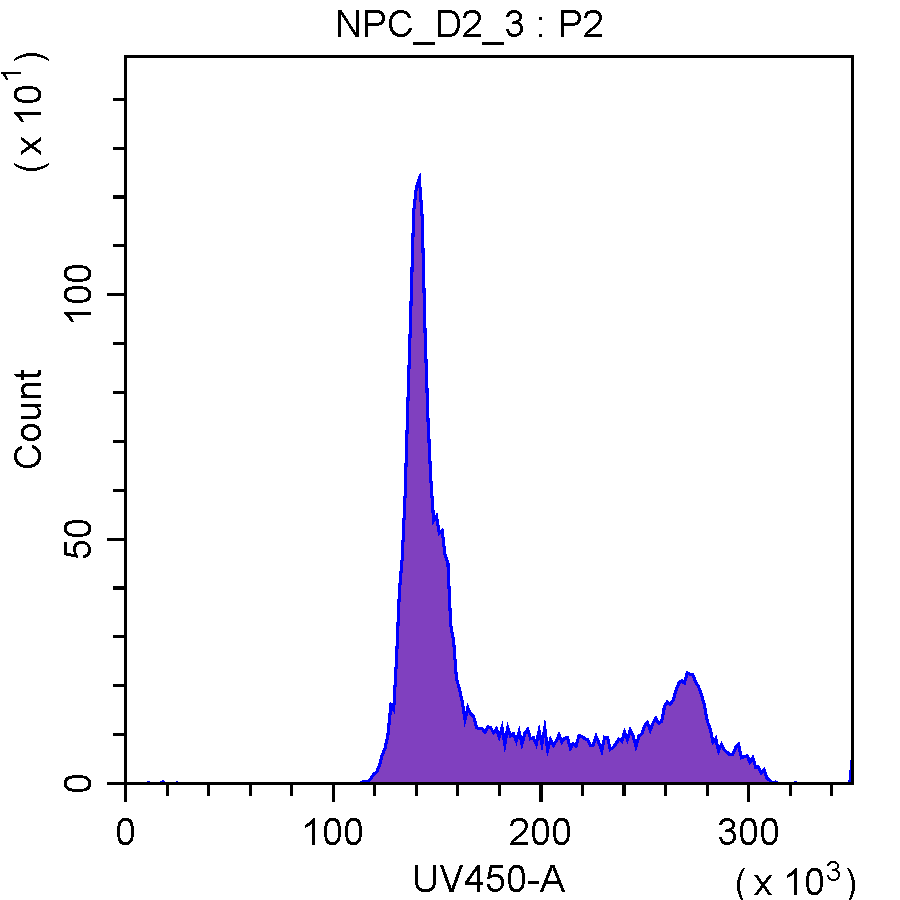

Supplement: Supplementary file 8 — Source data Fig. 6 [file 44318_2025_605_MOESM8_ESM.zip › Fig. 6/6B/Replicate_3/DAPI/NPC_D2.tif]

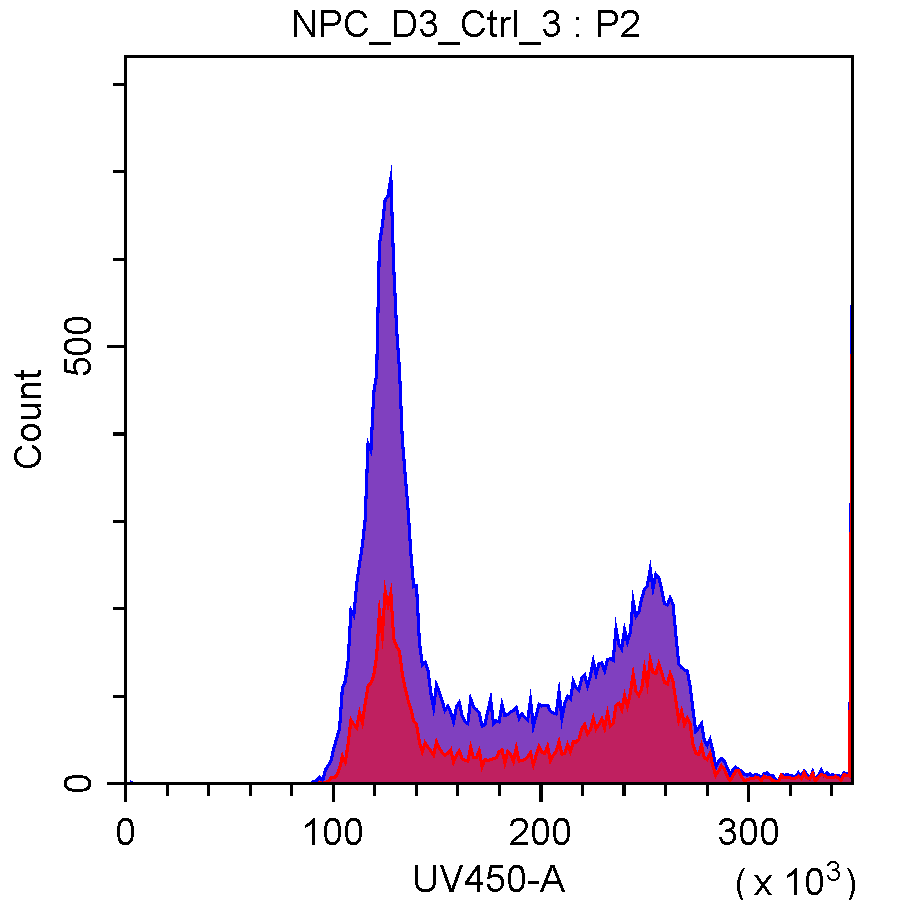

Supplement: Supplementary file 8 — Source data Fig. 6 [file 44318_2025_605_MOESM8_ESM.zip › Fig. 6/6B/Replicate_3/DAPI/NPC_D3_Ctrl.tif]

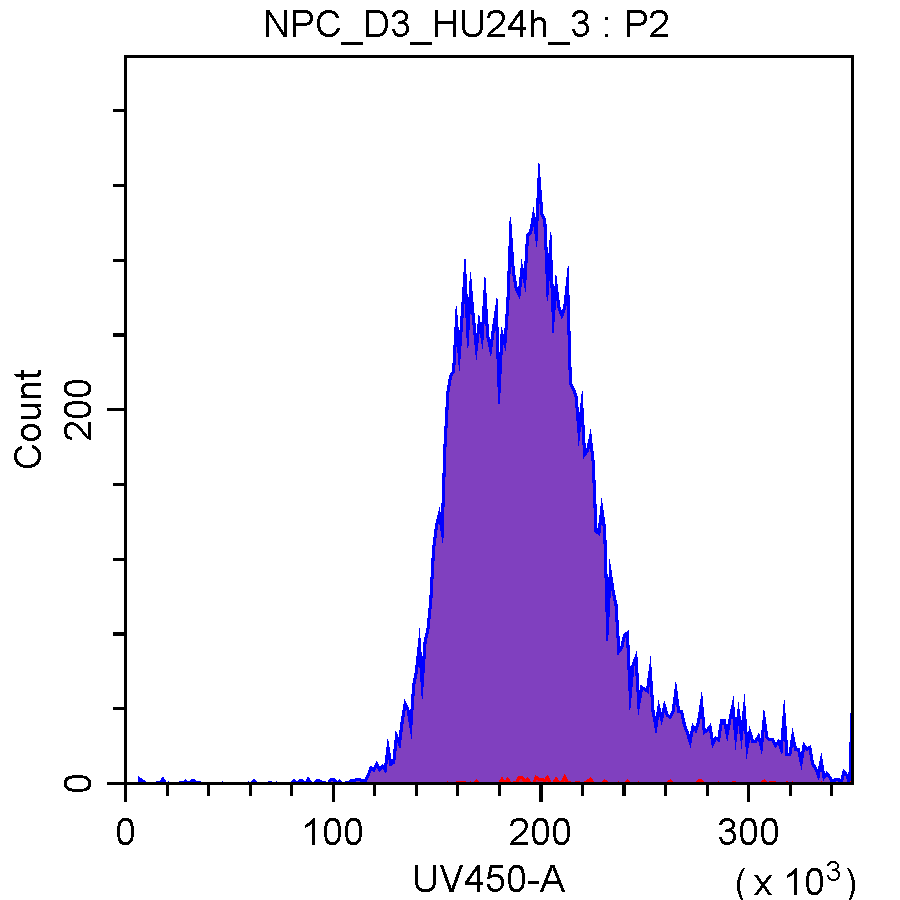

Supplement: Supplementary file 8 — Source data Fig. 6 [file 44318_2025_605_MOESM8_ESM.zip › Fig. 6/6B/Replicate_3/DAPI/NPC_D3_HU24h.tif]

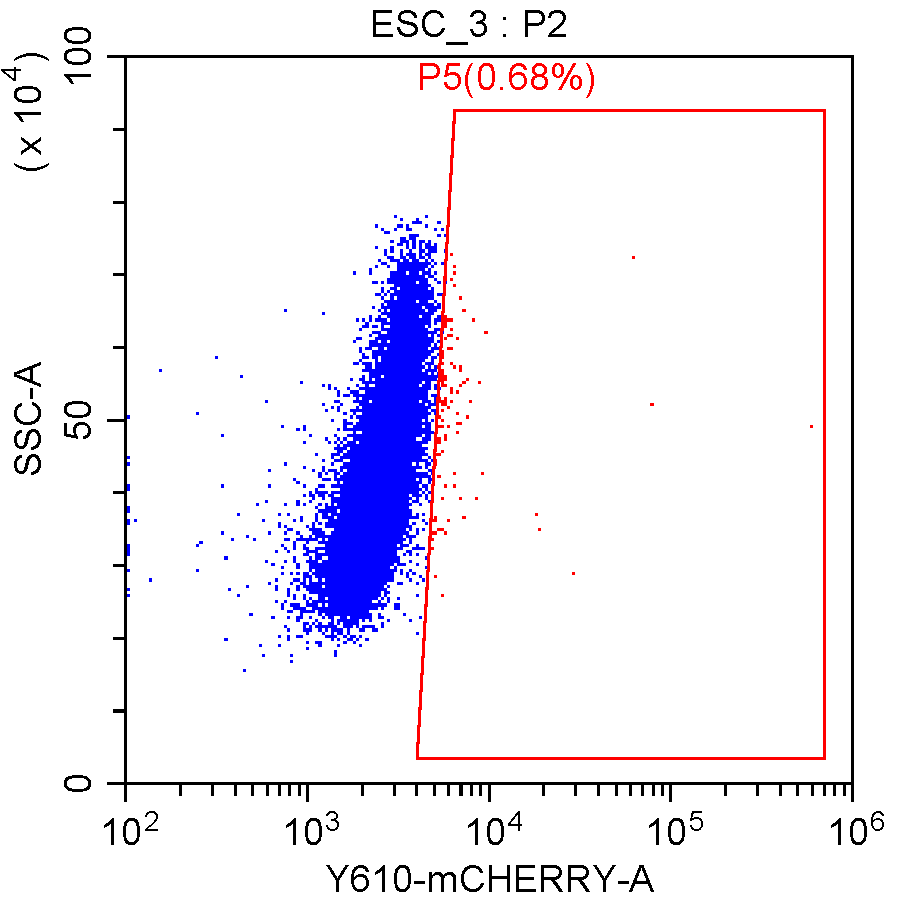

Supplement: Supplementary file 8 — Source data Fig. 6 [file 44318_2025_605_MOESM8_ESM.zip › Fig. 6/6B/Replicate_3/PAX6/ESC.tif]

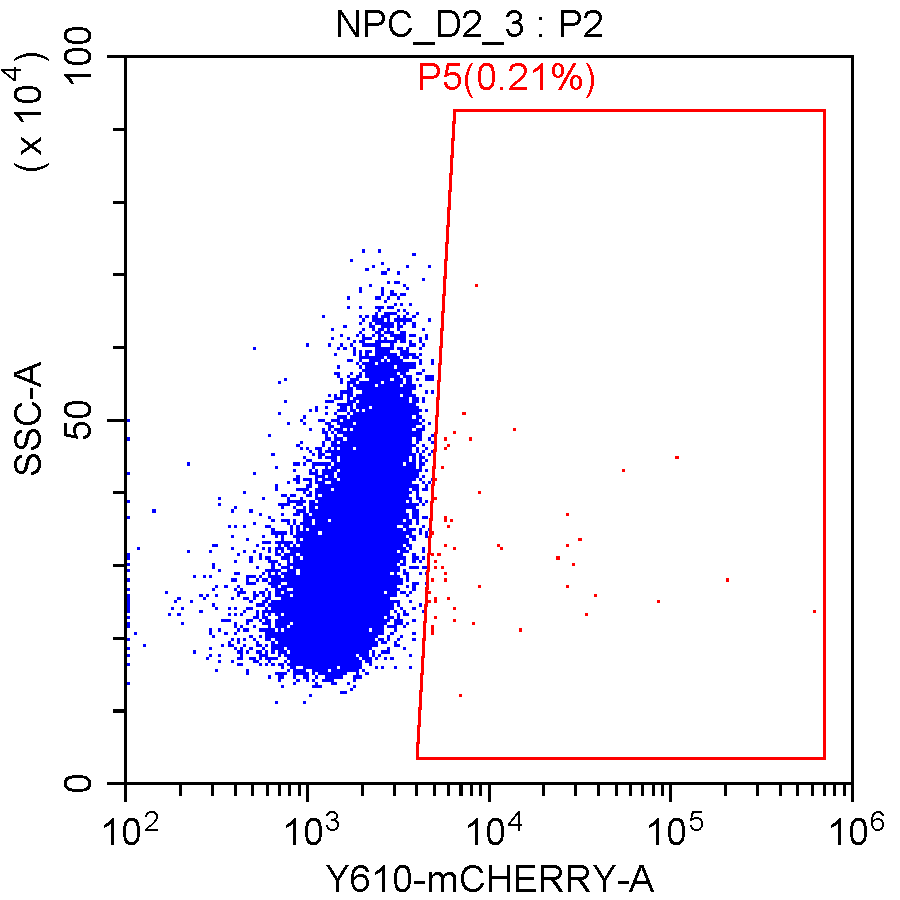

Supplement: Supplementary file 8 — Source data Fig. 6 [file 44318_2025_605_MOESM8_ESM.zip › Fig. 6/6B/Replicate_3/PAX6/NPC_D2.tif]

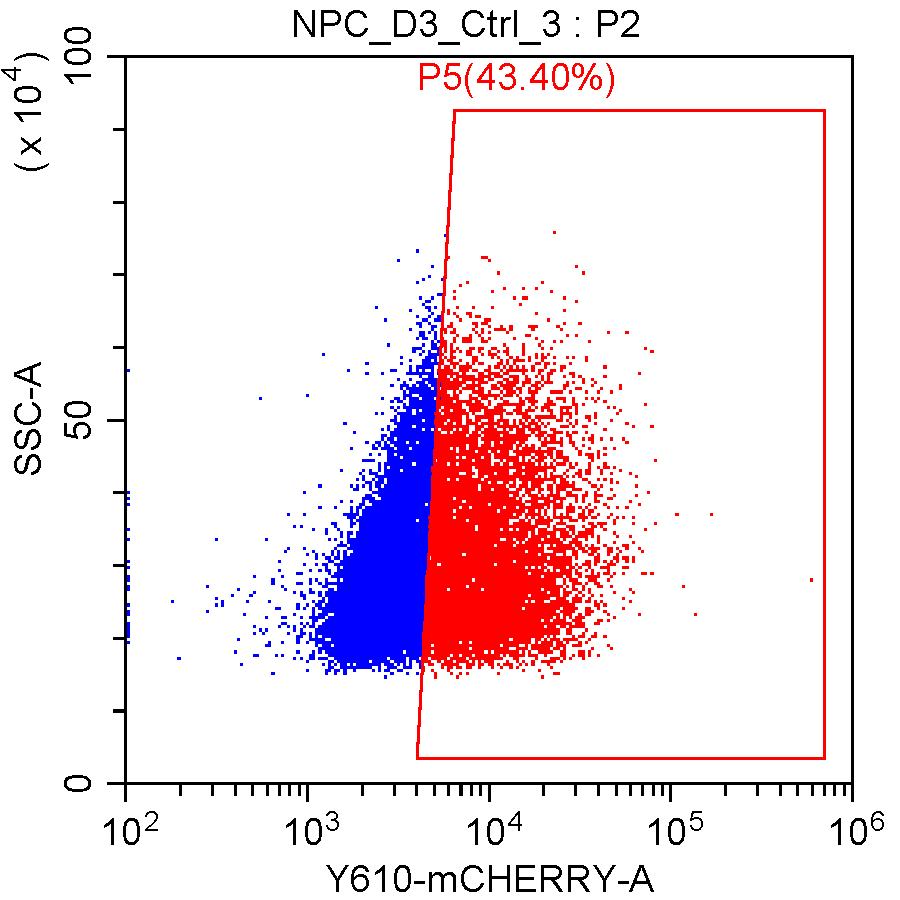

Supplement: Supplementary file 8 — Source data Fig. 6 [file 44318_2025_605_MOESM8_ESM.zip › Fig. 6/6B/Replicate_3/PAX6/NPC_D3_Ctrl.tif]

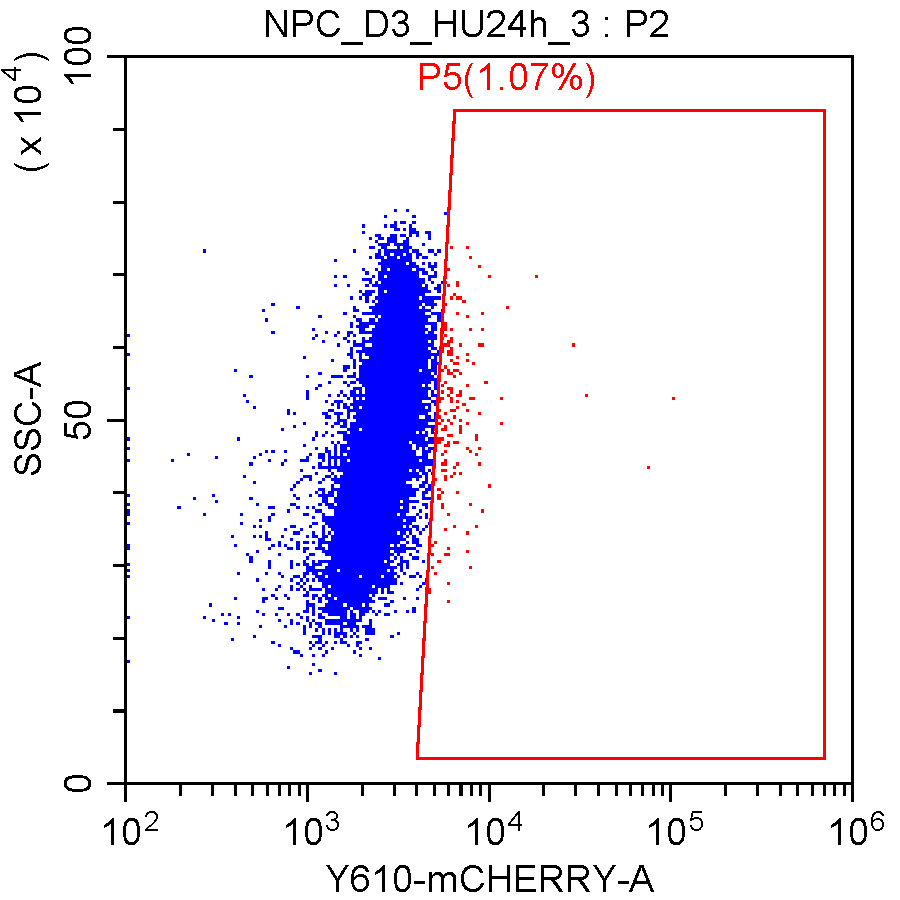

Supplement: Supplementary file 8 — Source data Fig. 6 [file 44318_2025_605_MOESM8_ESM.zip › Fig. 6/6B/Replicate_3/PAX6/NPC_D3_HU24h.tif]
