# Supplementary material for: Post-replicative initial expression of PAX6 during neuroectoderm differentiation
Source: EMBO J. 2025 Oct 21;44(23):7090–118. doi: 10.1038/s44318-025-00605-y (PMC12669799; doi:10.1038/s44318-025-00605-y)
Supplement: Supplementary file 9 — Expanded View Figures [file 44318_2025_605_MOESM9_ESM.pdf]

Expanded View Figures

Figure EV1. Transcriptomic and Proteomic characterization of cells during ESC–NPC differentiation.

(**A**) Volcano plot showing up- and downregulated genes in NPC_D3 compared to NPC_D2. The horizontal and vertical dotted lines indicate P value < 0.01 and |log2FC| >1.5, respectively. $n = 2$ independent experiments. (**B**) Volcano plot showing up- and downregulated proteins in NPC_D3 compared to NPC_D2. The horizontal and vertical dotted lines indicate P value < 0.05 and |log2FC| >0.5, respectively. $n = 2$ independent experiments. (**C**) Top 10 enriched Gene Ontology (GO) terms of upregulated genes in NPC_D3 compared to NPC_D2. (**D**) Top 10 enriched GO terms of upregulated proteins in NPC_D3 compared to NPC_D2. (**E**) Scatter plot showing the correlation between the changes in transcripts and the corresponding proteins between NPC_D3 and NPC_D2. The color of the points indicates the density of points at that location. (**F**) Venn diagram showing the count numbers of up- and downregulated transcripts and proteins in NPC_D3 compared to NPC_D2. (**G**) Time-series analysis of the proteomics (mfuzz) data of ESCs and NPCs at indicated times of neural induction. (**H**) GO analysis of the four protein clusters in (**G**). For the volcano plots, statistical analyses were performed using the negative binomial distribution model for transcriptomic data (**A**) and the t test for proteomic data (**B**). Statistical significance of GO enrichment analysis in (**C**, **D**) is determined using the hypergeometric test and Benjamini-Hochberg false discovery rate (FDR) correction, with a significance threshold of $P < 0.05$.

 © The Author(s)

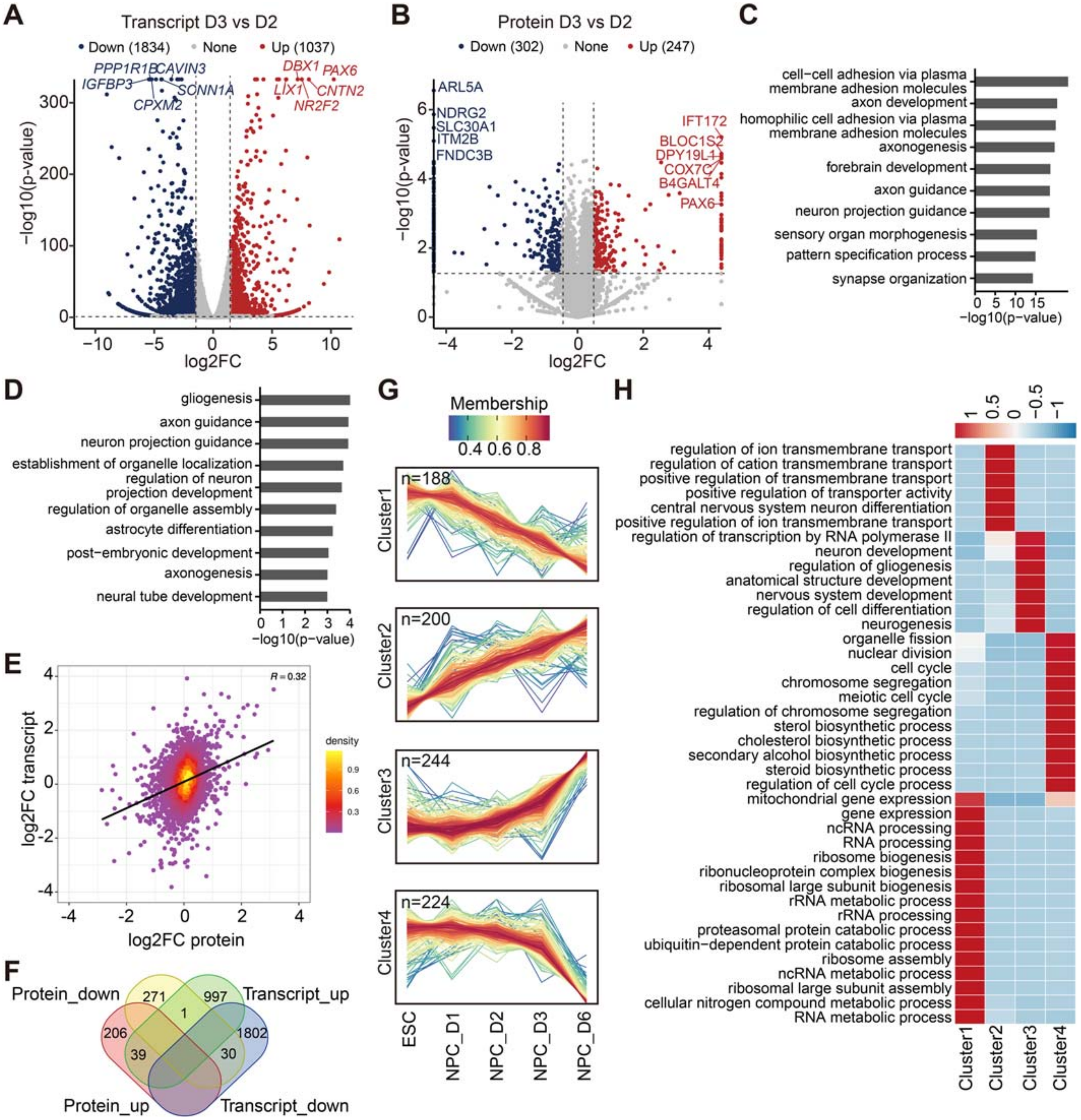
A
Transcript D3 vs D2
Down (1834) None Up (1037)
PPP1R1B CAVIN3 IGFBP3 SCNN1A CPXM2
DBX1 PAX6 LIX1 CNTN2 NR2F2
−log10(p−value)
log2FC
B
Protein D3 vs D2
Down (302) None Up (247)
ARL5A NDRG2 SLC30A1 ITM2B FNDC3B
IFT172 BLOC1S2 DPY19L1 COX7C B4GALT4 PAX6
−log10(p−value)
log2FC
C
cell−cell adhesion via plasma membrane adhesion molecules
axon development
homophilic cell adhesion via plasma membrane adhesion molecules
axonogenesis
forebrain development
axon guidance
neuron projection guidance
sensory organ morphogenesis
pattern specification process
synapse organization
−log10(p−value)
D
gliogenesis
axon guidance
neuron projection guidance
establishment of organelle localization
regulation of neuron projection development
regulation of organelle assembly
astrocyte differentiation
post−embryonic development
axonogenesis
neural tube development
−log10(p−value)
E
R = 0.32
density
log2FC transcript
log2FC protein
F
Protein_down Transcript_up Protein_up Transcript_down
271 997 1 206 1802 39 30
G
Membership
Cluster1 n=188
Cluster2 n=200
Cluster3 n=244
Cluster4 n=224
ESC NPC_D1 NPC_D2 NPC_D3 NPC_D6
H
regulation of ion transmembrane transport
regulation of cation transmembrane transport
positive regulation of transmembrane transport
positive regulation of transporter activity
central nervous system neuron differentiation
positive regulation of ion transmembrane transport
regulation of transcription by RNA polymerase II
neuron development
regulation of gliogenesis
anatomical structure development
nervous system development
regulation of cell differentiation
neurogenesis
organelle fission
nuclear division
cell cycle
chromosome segregation
meiotic cell cycle
regulation of chromosome segregation
sterol biosynthetic process
cholesterol biosynthetic process
secondary alcohol biosynthetic process
steroid biosynthetic process
regulation of cell cycle process
mitochondrial gene expression
gene expression
ncRNA processing
RNA processing
ribosome biogenesis
ribonucleoprotein complex biogenesis
ribosomal large subunit biogenesis
rRNA metabolic process
rRNA processing
proteasomal protein catabolic process
ubiquitin−dependent protein catabolic process
ribosome assembly
ncRNA metabolic process
ribosomal large subunit assembly
cellular nitrogen compound metabolic process
RNA metabolic process
Cluster1 Cluster2 Cluster3 Cluster4

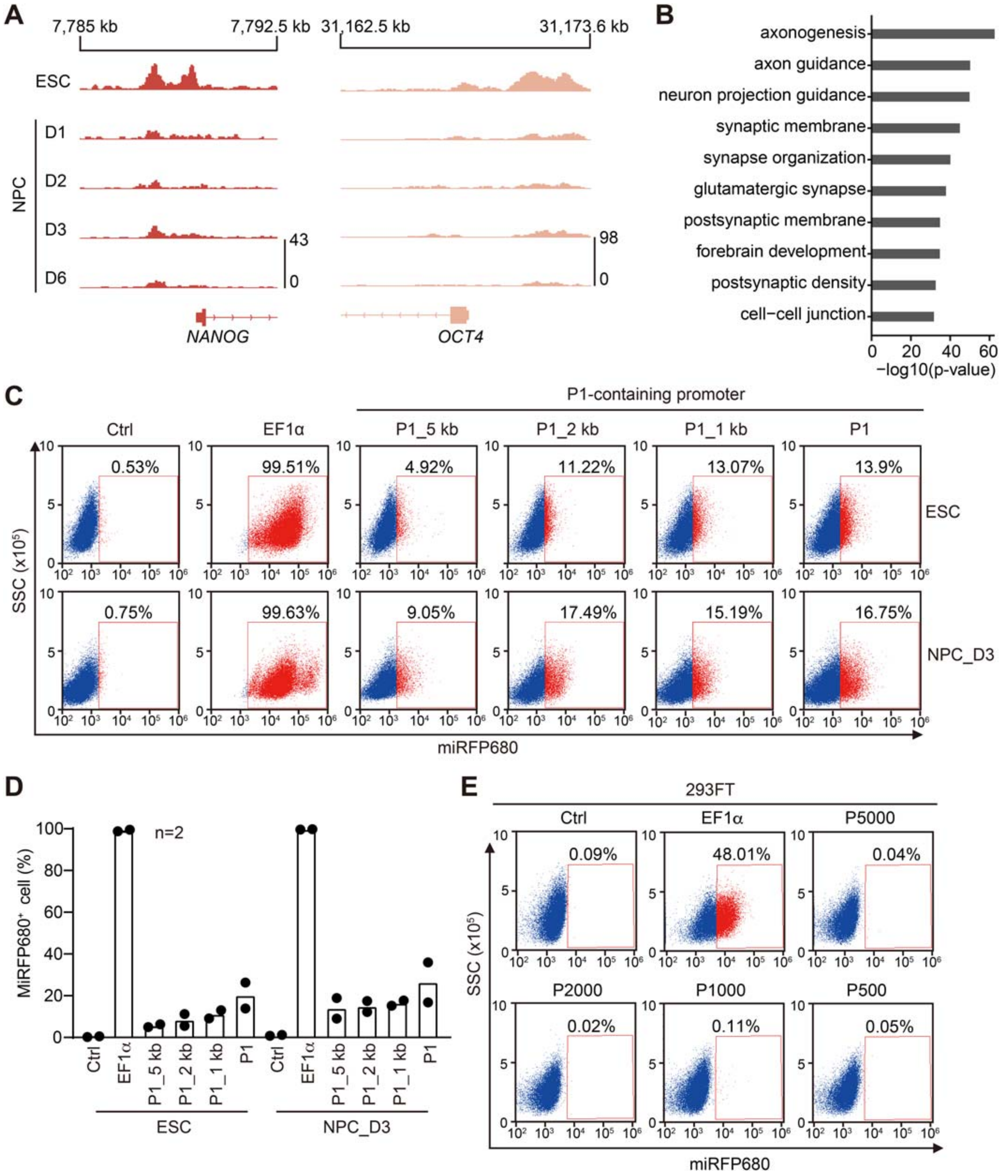
A
7,785 kb
7,792.5 kb
31,162.5 kb
31,173.6 kb
ESC
D1
D2
D3
D6
NPC
43
0
98
0
NANOG
OCT4
B
axonogenesis
axon guidance
neuron projection guidance
synaptic membrane
synapse organization
glutamatergic synapse
postsynaptic membrane
forebrain development
postsynaptic density
cell−cell junction
0 20 40 60
−log10(p-value)
C
P1-containing promoter
Ctrl
EF1α
P1_5 kb
P1_2 kb
P1_1 kb
P1
0.53%
99.51%
4.92%
11.22%
13.07%
13.9%
ESC
0.75%
99.63%
9.05%
17.49%
15.19%
16.75%
NPC_D3
SSC (x10⁵)
miRFP680
D
MiRFP680⁺ cell (%)
n=2
Ctrl
EF1α
P1_5 kb
P1_2 kb
P1_1 kb
P1
ESC
NPC_D3
E
293FT
Ctrl
EF1α
P5000
0.09%
48.01%
0.04%
P2000
P1000
P500
0.02%
0.11%
0.05%
SSC (x10⁵)
miRFP680

Figure EV2. G2-enhanced PAX6 transcription is driven by the 500-bp promoter.

(**A**) ATAC-seq tracks showing the chromatin accessibility at the *NANOG* and *OCT4* gene loci in ESCs and NPCs from days 1–6 of neural induction. (**B**) Top 10 enriched Gene Ontology (GO) pathways of genes with increased chromatin accessibility in NPC_D3 compared to NPC_D2. Statistical significance of GO enrichment analysis is determined using the hypergeometric test and Benjamini-Hochberg false discovery rate (FDR) correction, with a significance threshold of $P < 0.05$. (**C**) FACS plots showing the expression of the miRFP680 reporter driven by *PAX6* P1-containing promoters of different lengths in ESCs and NPCs at day 3 of neural induction (NPC_D3). The EF1α promoter was used as the positive control. Uninfected cells (Ctrl) were used as the negative control. (**D**) Quantification of the percentage of miRFP680^{+} cells in (**C**). $n = 2$ independent experiments. (**E**) FACS plots showing the expression of the miRFP680 reporter driven by P500-containing promoters in 293FT cells. The EF1α promoter was used as the positive control, and uninfected cells (Ctrl) were used as the negative control.

© The Author(s)

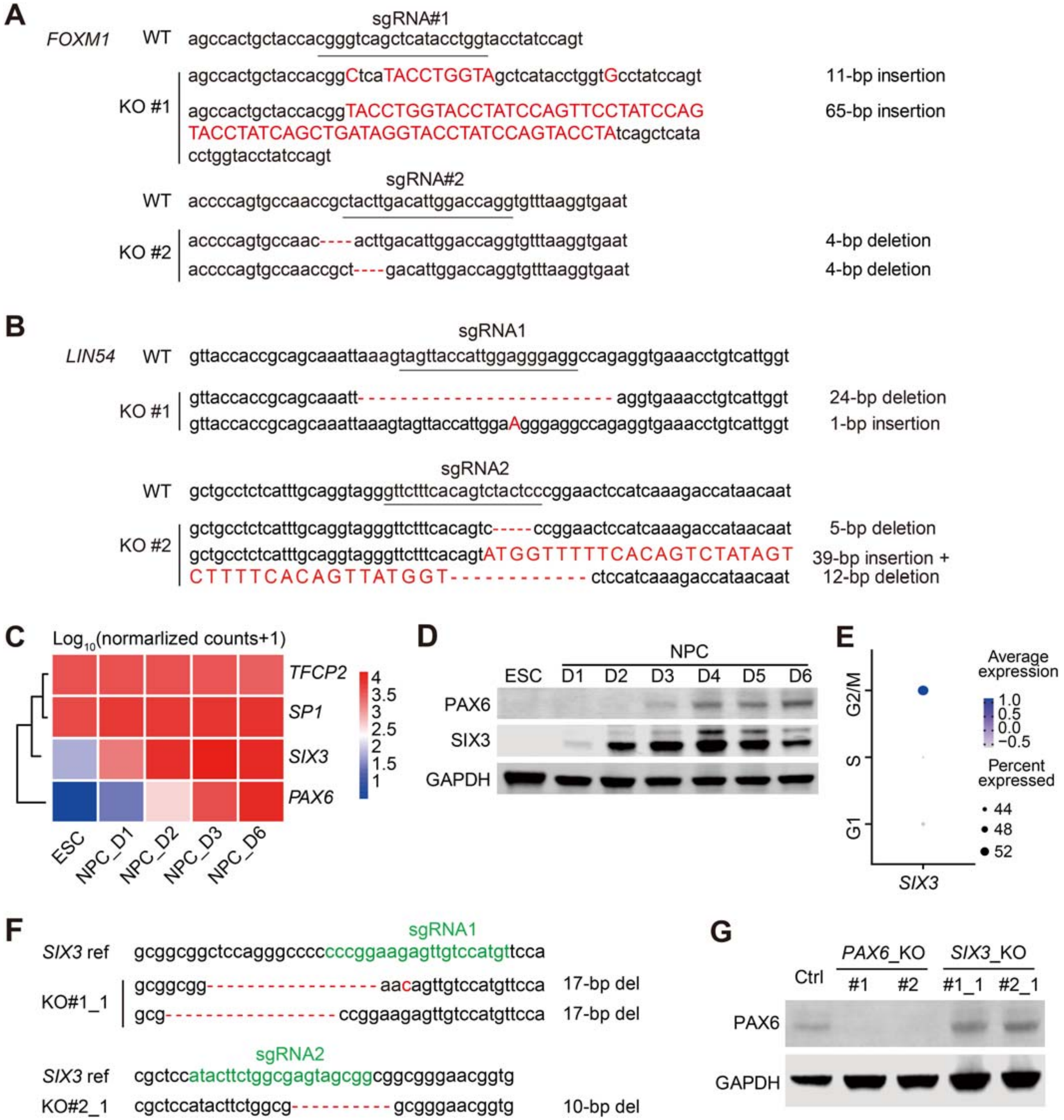

Figure EV3. FOXM1, LIN54, and SIX3 and EGR1 are not required for *PAX6* expression during ESC–NPC transition.

(**A**, **B**) Sanger sequencing of the *FOXM1* (**A**) and *LIN54* (**B**) genes in different ESC knockout (KO) clones. The sgRNA sequences used for the genome editing were underlined in the wild-type (WT) reference sequence. Indels and insertions in the genome were shown in red font. (**C**) Heatmap showing the expression levels of putative *PAX6* upstream regulators, such as *TFCP2*, *SP1*, and *SIX3* in ESCs and NPCs at different times of neural induction. *PAX6* expression was included as a positive control. The color bar represented values calculated as log10 (normalized counts + 1). (**D**) Western blots showing the protein levels of SIX3 and PAX6 in ESCs and NPCs. (**E**) Dot plot showing the expression levels of *SIX3* in different cell cycle phases from NPC_24h to NPC_52h based on the integrated scRNA-seq data. (**F**) Sanger sequencing of the *SIX3* gene in two ESC KO clones. The sgRNA sequences were highlighted in green, and indels in the genome were indicated in red. (**G**) Western blots showing the protein levels of PAX6 in WT and *SIX3* KO NPCs on D3 of neural induction. Two *PAX6* KO NPC_D3 were used as the positive control.

 © The Author(s)

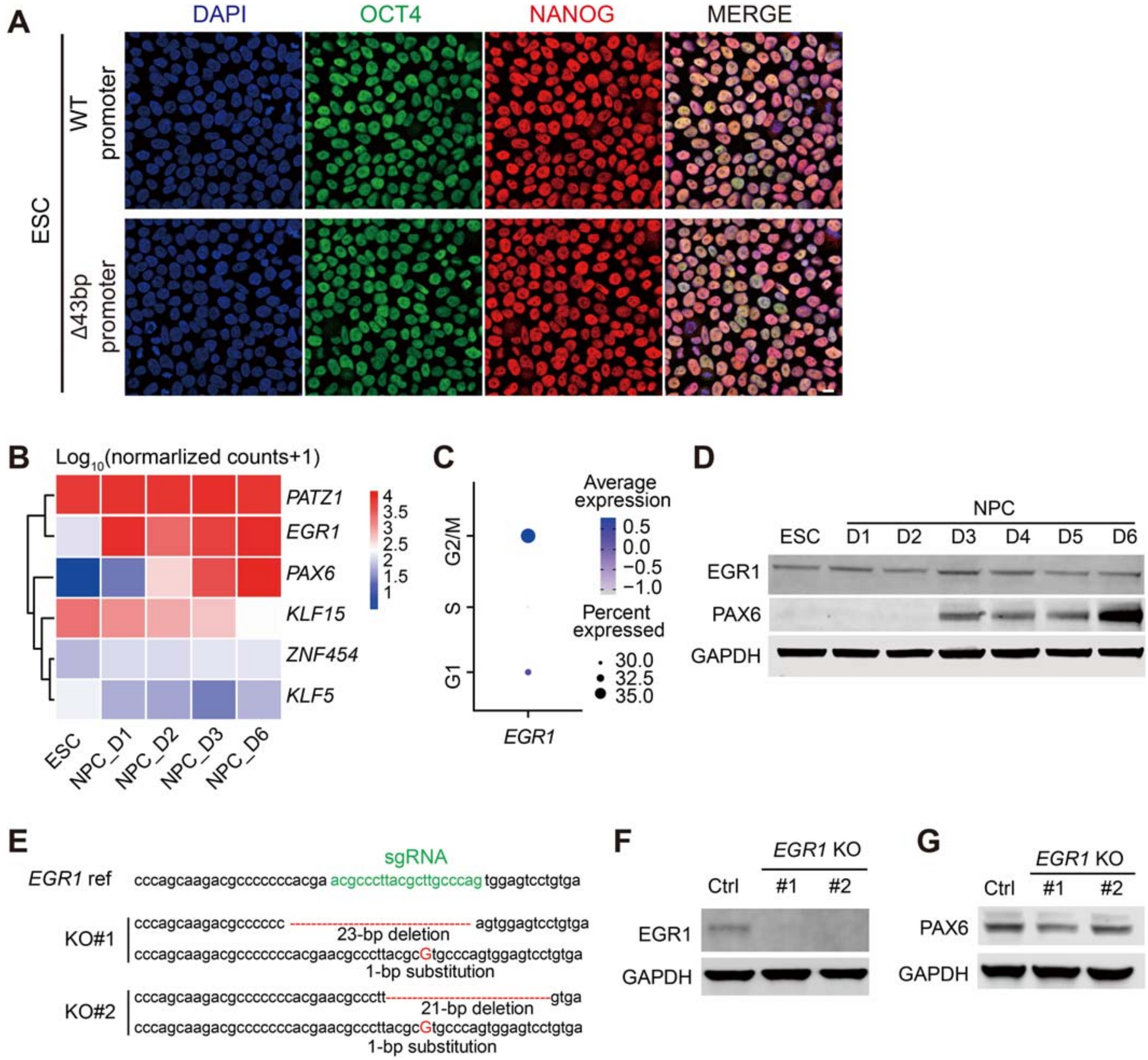

Figure EV4. SIX3 and EGR1 are not required for *PAX6* expression during ESC–NPC transition.

(**A**) Images of WT ESCs or ESCs with the Δ43 bp deletion from the *PAX6* promoter stained with DAPI and antibodies against OCT4 (green) and NANOG (red). Scale bar, 10 μm. (**B**) Heatmap showing the expression levels of predicted upstream regulators of *PAX6* in ESCs and NPCs at different times of neural induction. (**C**) Dot plot showing the expression levels of *EGR1* in different cell cycle phases from NPC_24h to NPC_52h based on the integrated scRNA-seq data. (**D**) Western blots showing the protein levels of EGR1 and PAX6 in ESCs and NPCs. (**E**) Sanger sequencing of the *EGR1* gene in two ESC knock out (KO) clones. The sgRNA sequence was highlighted in green, and indels in the genome were indicated in red. (**F**, **G**) Western blots showing the protein levels of EGR1 in WT and *EGR1* KO ESCs (**F**) and PAX6 in WT and *EGR1* KO NPCs on D3 of neural induction (**G**).

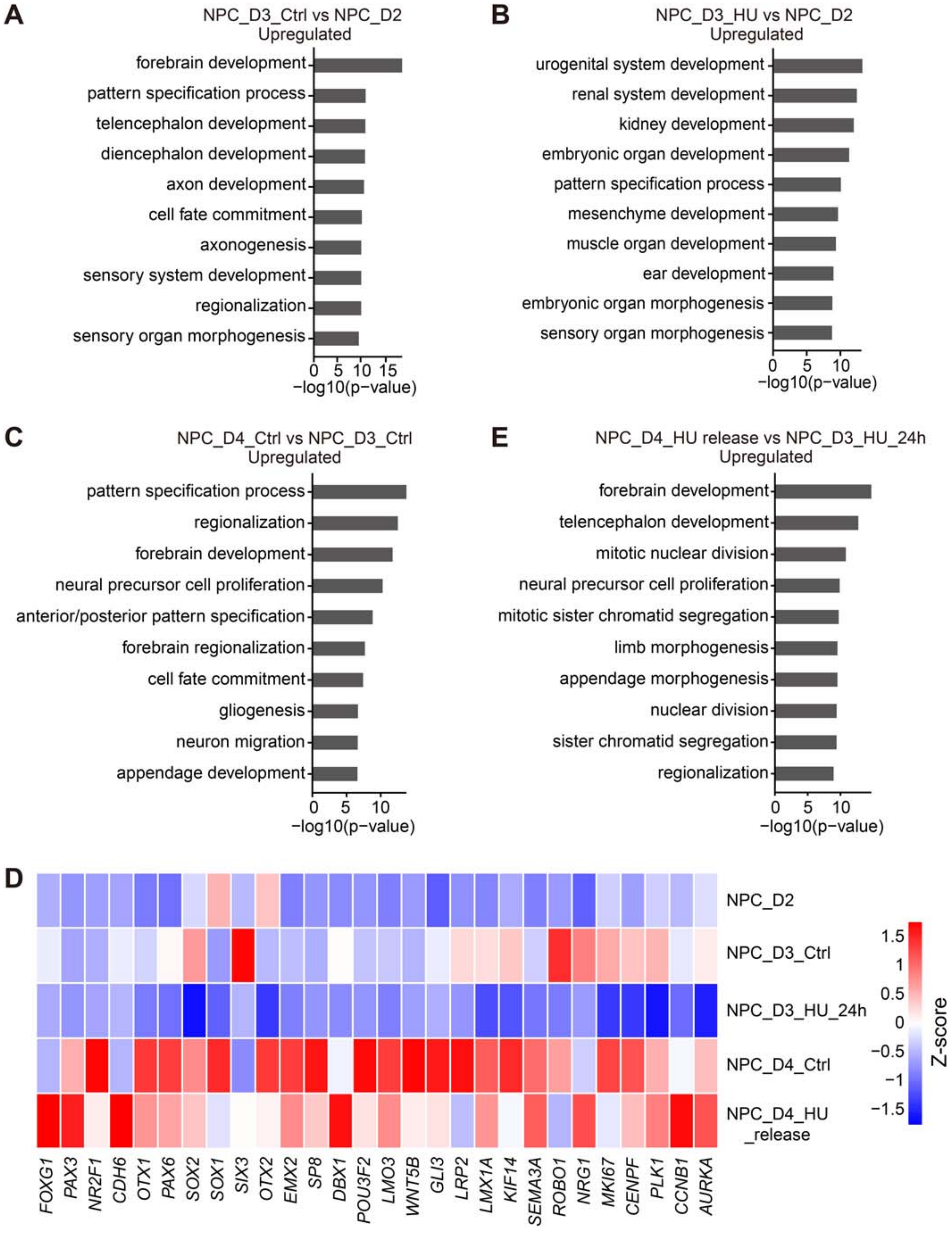
A
NPC_D3_Ctrl vs NPC_D2
Upregulated
forebrain development
pattern specification process
telencephalon development
diencephalon development
axon development
cell fate commitment
axonogenesis
sensory system development
regionalization
sensory organ morphogenesis
0 5 10 15
−log10(p−value)
B
NPC_D3_HU vs NPC_D2
Upregulated
urogenital system development
renal system development
kidney development
embryonic organ development
pattern specification process
mesenchyme development
muscle organ development
ear development
embryonic organ morphogenesis
sensory organ morphogenesis
0 5 10
−log10(p−value)
C
NPC_D4_Ctrl vs NPC_D3_Ctrl
Upregulated
pattern specification process
regionalization
forebrain development
neural precursor cell proliferation
anterior/posterior pattern specification
forebrain regionalization
cell fate commitment
gliogenesis
neuron migration
appendage development
0 5 10
−log10(p−value)
E
NPC_D4_HU release vs NPC_D3_HU_24h
Upregulated
forebrain development
telencephalon development
mitotic nuclear division
neural precursor cell proliferation
mitotic sister chromatid segregation
limb morphogenesis
appendage morphogenesis
nuclear division
sister chromatid segregation
regionalization
0 5 10
−log10(p−value)
D
NPC_D2
NPC_D3_Ctrl
NPC_D3_HU_24h
NPC_D4_Ctrl
NPC_D4_HU
_release
1.5
1
0.5
0
−0.5
−1
−1.5
Z-score
FOXG1 PAX3 NR2F1 CDH6 OTX1 PAX6 SOX2 SOX1 SIX3 OTX2 EMX2 SP8 DBX1 POU3F2 LMO3 WNT5B GLI3 LRP2 LMX1A KIF14 SEMA3A ROBO1 NRG1 MKI67 CENPF PLK1 CCNB1 AURKA

 © The Author(s)

Figure EV5. Hydroxyurea (HU) treatment impairs the ESC–NPC transition.

(**A**) Top 10 enriched Gene Ontology (GO) pathways of upregulated genes in normally differentiated NPC_D3 compared to NPC_D2. (**B**) Top 10 enriched GO pathways of upregulated genes in HU-treated NPC_D3 compared to NPC_D2. (**C**) Top 10 enriched GO pathways of upregulated genes in normally differentiated NPC_D4 compared to NPC_D3. (**D**) Heatmap showing the expression levels of neural lineage genes and cell division genes in normally differentiated NPCs, HU-arrested NPC_D3 cells, and NPC_D4 following HU release. The color bar represented Z-scores. (**E**) Top 10 enriched GO pathways of upregulated genes in NPC_D4 with HU release compared to HU-treated NPC_D3. Statistical significance of GO enrichment analysis in (**A**, **B**, **C**, **E**) is determined using the hypergeometric test and Benjamini-Hochberg false discovery rate (FDR) correction, with a significance threshold of $P < 0.05$.

© The Author(s)
